# Wireless electrostimulation implants enable sphincter neuromuscular improvement toward mixed urinary incontinence

**DOI:** 10.1038/s41467-026-71532-7

**Published:** 2026-04-15

**Authors:** Tianxiang Zheng, Li Tao, Qiuhua Gao, Zhiran Yi, Yaoxia Shao, Yu Xiao, Ning Kang, Christopher H. T. Lee, Ming Liu, Chengbin Ma, Wenming Zhang, Yuan Shao, Lei Shao, Metin Sitti

**Affiliations:** 1 Global College, Shanghai Jiao Tong University, Shanghai, China; 2 Ruijin Hospital, School of Medicine, Shanghai Jiao Tong University, Shanghai, China; 3 School of Mechanical Engineering, Shanghai Jiao Tong University, Shanghai, China; 4 School of Information and Electrical Engineering, Hangzhou City University, Hangzhou, China; 5 School of Electrical and Electronic Engineering, Nanyang Technological University, Singapore, Singapore; 6 Lee Kong Chian School of Medicine, Nanyang Technological University, Singapore, Singapore; 7 School of Electrical Engineering, Shanghai Jiao Tong University, Shanghai, China; 8 School of Medicine and College of Engineering, Koç University, Istanbul, Turkey; 9 Physical Intelligence Department, Max Planck Institute for Intelligent Systems, Stuttgart, Germany

**Keywords:** Biomedical engineering, Urological manifestations

## Abstract

Mixed urinary incontinence, involving concurrent or diagnostically unclear components of urge- and stress-related dysfunction, remains poorly addressed by current therapeutic strategies. State-of-the-art electrostimulation relies primarily on sacral neuromodulation for urge-predominant symptoms, requiring bulky (~3 cm³) and costly (>$10,000) implants, while stress incontinence is largely managed by mechanical or surgical interventions that do not restore sphincter neuromuscular function. Here, we propose wireless-powered implanted programmable electrostimulation of sphincters (WIPES) that bypasses long nerve loops and provides on-site stimulation at the urethral sphincter. The adhesive-patch-like soft implant integrates wireless power receiving and tunable pulse generation, enabling long-term controlled stimulation with reduced size (<0.3 cm³) and weight (<0.9 g). In rats with experimentally induced urge or stress incontinence (in separate rat models of urge and stress incontinence established under the same experimental conditions), WIPES delivered free-moving stimulation and achieved average alleviation rates of 90.62% (UUI) and 97.92% (SUI) in voiding frequency and volume. WIPES was associated with peri-urethral neuromuscular remodeling and improved bladder–urethral functional outcomes, supporting its potential role in coordinated continence control.

## Abstract (web summary)

Mixed urinary incontinence troubles many patients worldwide but lacks effective therapies. Here, the authors present a wireless-powered soft bioelectronic implant to provide on-demand stimulation of the urethral sphincter, leading to improvement in both urge and stress urinary incontinence.

## Introduction

Urinary incontinence, a very common condition with global prevalence around 35%-45%,^1^ is characterized by the progressive worsening of unintentional urine leakage, reduced bladder capacity, or sudden strong urges to urinate. If left untreated, it can become permanent and lead to limited work capability and social isolation in people, significantly reducing the quality of life. Among many different types of urinary incontinence, the most common pathology is often due to two reasons. Stress urinary incontinence (SUI) results from impaired urethral neuromuscular control, causing urine leakage during physical exertion when urethral closure pressure fails to counter increased abdominal pressure. In contrast, urge urinary incontinence (UUI) arises from neural dysfunction leading to involuntary over-active bladder and sudden urgency before voiding^2^.

SUI is usually caused by different factors depending on gender and is more common in women than men. Among women, ~30–50% suffer different degrees of incontinence during or after pregnancy due to pelvic floor muscle dysfunction, and the most common cause in men is surgeries for benign prostatic hyperplasia or prostate cancer, though it can also result from trauma, iatrogenic injury, and neurological disorders for both men and women^3,4^. Serious SUI, such as caused by excision of sphincter muscle, is treated by a sling surgery in which a soft strap physically shuts the urethra and could open up to urinate when needed^5^. As for mild sphincter damage, magnetic stimulation has been proven effective but only limited to those caused by pelvic floor muscle dysfunction in pregnant women, with its outcomes varying greatly, too^6,7^.

On the other hand, UUI occurs both frequently in men and women, which is more complex to tackle. It is usually treated by electrostimulation therapies through the sacral plexus S2-S4 of the spinal nerves, which can regulate the neural functions of bladder and sphincter. For instance, transcutaneous tibial nerve stimulation^8–11^ and posterior tibial nerve stimulation have shown benefits in improving UUI as the tibial nerve is a peripheral extent from the sacral plexus^12–15^, but are gradually phasing out because the efficacy is relatively low due to the distant stimulation site near the foot. Sacral nerve stimulation, the currently incumbent clinical therapy, consists of an implant with lead wires to access to the sacral plexus S2-S4, which can relieve symptoms of UUI by modulating the neural signals sent from and to the bladder and sphincter^16–19^. The state-of-the-art commercial electrostimulation implant, such as the InterStim^TM^ Micro, features a bulky volume of 2.8 cm^3^, a heavy weight of 13 grams, a high price between $20,000–30,000^20^. They maintain a therapeutic effectiveness of ~46–47% complete-dry rates at 6–36 months (a conservative endpoint), while the responder rates are typically ~76% for a reduction over 50%^21^ (Table S1 for additional commercial device comparisons). More unfortunately, it only targets UUI and yields drastically low efficacy in case that the muscular function controlling the bladder and sphincter is damaged, too. This is a combination of the two types and known as mixed urinary incontinence (MUI), which is believed to affect nearly one third of the patients in both men and women^2,22^.

The different types of urinary incontinence require an accurate diagnosis to identify the exact type in order to develop an effective treatment plan, which, however, is usually not easy to determine, while the frequent and large-scale MUI also greatly complicates this issue. As a result, patients may undergo an extensive list of steps to diagnose urinary incontinence including behavior questionnaire, bladder stress test, catheterization, urine culture, urodynamic evaluation and ultrasound. This difficulty leads to resource-consuming trial-and-error treatment plans involving numerous clinical visits for a combination of measures, and sometimes exhausted trials of medications.

Currently lacking is a means to work for SUI, UUI, and MUI as well, with efficacy well surpassing existing devices. It is thus desired that the therapy could not only regulate the neural signaling of bladder and sphincter, but also repair the damaged muscular function. In addition, the biological mechanism of current methods for relieving urinary incontinence is largely unknown or not well explored, even in rat models, which needs to be uncovered during experiment, too. In terms of device engineering, it is expected that the device would be soft and small for minimal invasion, customizable for individual level of severity, easy to operate in both outpatient clinics and at home, and staying inside the human body for a long term without inflammation which could be a result of direct contact with the nerves^23–28^. A wireless power without onboard batteries is also favored due to reduced volume of implants and enhanced long-term safety^29–33^.

Here, we propose wireless-powered implanted programmable electrostimulation of sphincters (WIPES) to provide direct stimulation at the nerves and smooth muscles of the urethral sphincter. A soft, wireless-powered device is implanted in the lower abdomen to deliver conditioned and tunable pulse patterns to the sphincter with drastically reduced size (<0.3 cm^3^), weight (<0.9 g) and cost. This implant could enable long-term controlled tetherless electrostimulation at any point-of-care facility equipped with an external emitting coil, which could be easily positioned near the lower abdomen with a distance extending to about 20 cm. In this study, the WIPES device was successfully validated in numerous rats and they have been staying healthy for at least 1 year till now. Bidirectional & biperiodic pulses are optimized to produce the highest and most sustained contraction pressure in rat urethral sphincter. After stimulation in rats with experimentally induced UUI or SUI (modeled in two separate cohorts because true MUI cannot currently be created in a single rat), the improvement rates reached 90.62% (UUI) and 97.92% (SUI), as evidenced by reduced urination frequency and increased average urination volume. Furthermore, cell culture and tissue analysis revealed marked nerve regeneration and enhanced muscle activity of the rat urethral sphincter, which well targets the cause of both UUI and SUI. Compared with prior preclinical approaches in rat models, our work provides an integrated therapeutic platform supported by multi-level functional and biological evaluations.

## Results

### System overview of WIPES

**Fig. 1:**
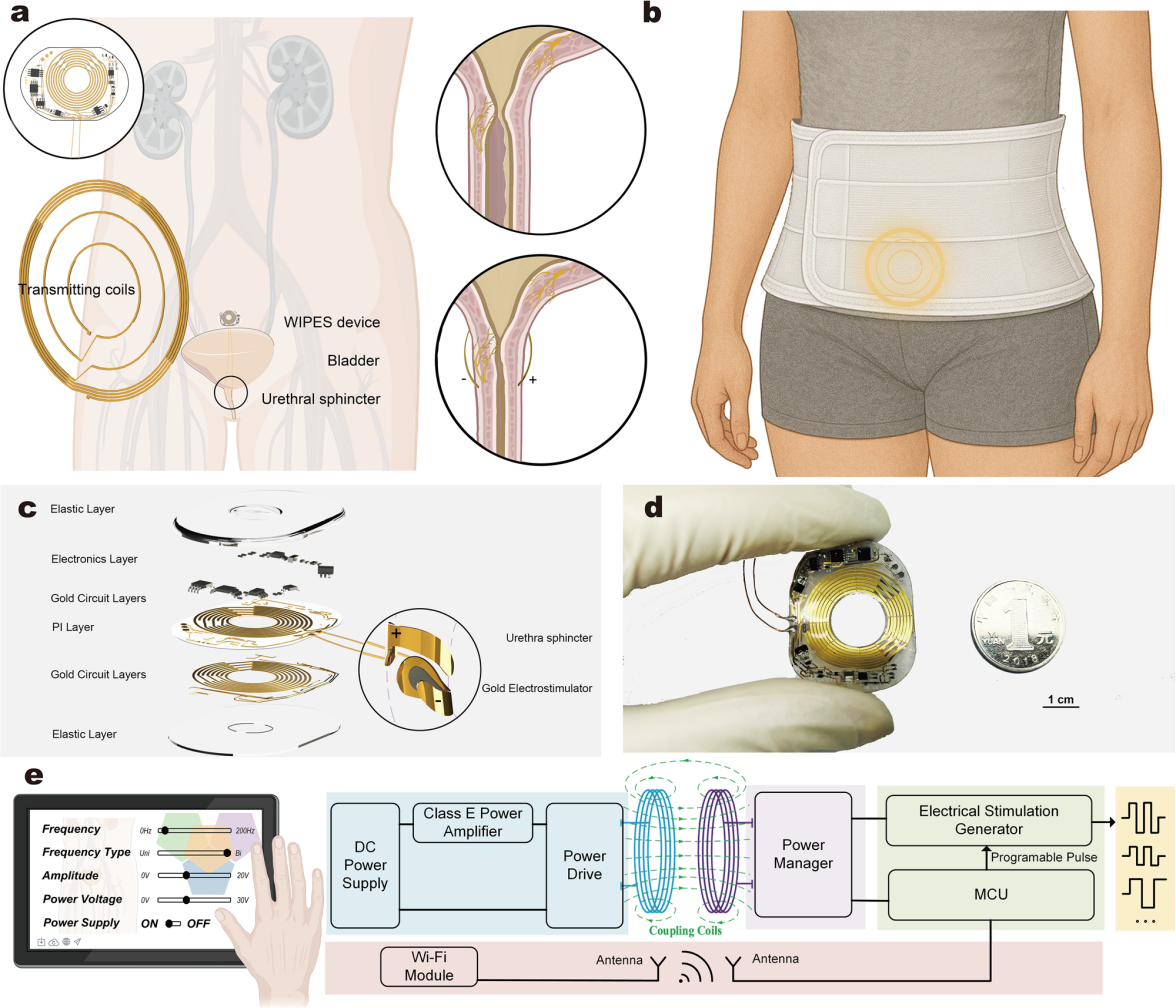
Overview and design of the WIPES. **a** Working principle of the WIPES, consisting of an external Tx coil and an implanted device housing the Rx coil. Two wires extend from the implant to deliver electrostimulation pulses to the urinary sphincter, enhancing neuromuscular function. **b** Wearable transmitting belt enabling wireless power transfer to the implanted WIPES device. **c** Exploded view of the adhesive-patch-like implant, featuring an elliptical racetrack design consisting of six functional layers, with specially designed gold electrodes, which wrap around the urethral sphincter to deliver pulses at the surface of contact with the sphincter. **d** The fabricated implant's size and flexibility. **e** Control strategy of the WIPES system including 1) a mobile APP that controls the stimulation frequency, type, amplitude and voltage, 2) Tx coil with power supply and amplification, 3) Rx coil cascaded with functional modules including power management, MCU and stimulation pulse generator, and 4) Wi-Fi module enabling tetherless customized pulse tuning. Graphical elements in Fig. 1a, e are created in BioRender. Dwad, D. (2026) [https://biorender.com/eknsmzf].

The overall working principle and key components of WIPES are shown in Fig. 1a. The WIPES system is mainly composed of an external transmission coil (Tx), and an adhesive-patch-like soft implant accommodating a receiving coil (Rx) for wireless power transfer (WPT) followed by sophisticated circuits with a user interface. The implant is designed to be positioned at lower abdomen near the bladder and delivers electrostimulation via two electrons softly fixed on the urethral sphincter, which is the primary site for stimulation. In clinical application, the device is expected to be surgically implanted at the extraperitoneal fascia above the pubic bone by a minimally invasive surgery. Patients can receive electrostimulation treatment at customized frequencies and voltages for a designed period in a clinic or at home using the belt-integrated Tx coil (Fig. 1b), which is worn around the waist to provide portable, continuous power to WIPES. The implanted device is designed to be an elliptical racetrack shape and consists of six thin functional layers, as shown in Fig. 1c and d, including the outermost elastic layers, inner electronics layer, gold coil layers with extended wires serving as stimulation probes. The top and bottom elastomer layers ensure excellent flexibility and biocompatibility within human body, and protect the internal electronics and gold Rx coils from contamination^34–37^. The electrodes are designed as flexible C-shaped gold patches that wrap gently around the sphincter, ensuring stable contact without exerting constrictive pressure (Fig. S27). The inner surface serves as the active interface, while the outer surface and connecting leads are fully encapsulated to maintain insulation. The operation of the WIPES system is depicted in Fig. 1e. The induced AC electrical current in the Rx coil is rectified and regulated through an on-board power management module, which supplies a DC power to the on-board microcontroller unit (MCU) and the electrostimulation delivery module. In addition, the on-board Wi-Fi allows tetherless, real-time adjustment of the pattern, frequency and intensity of the electrical stimuli for different patients and treatment stages.

### Wireless powered circuit design

**Fig. 2:**
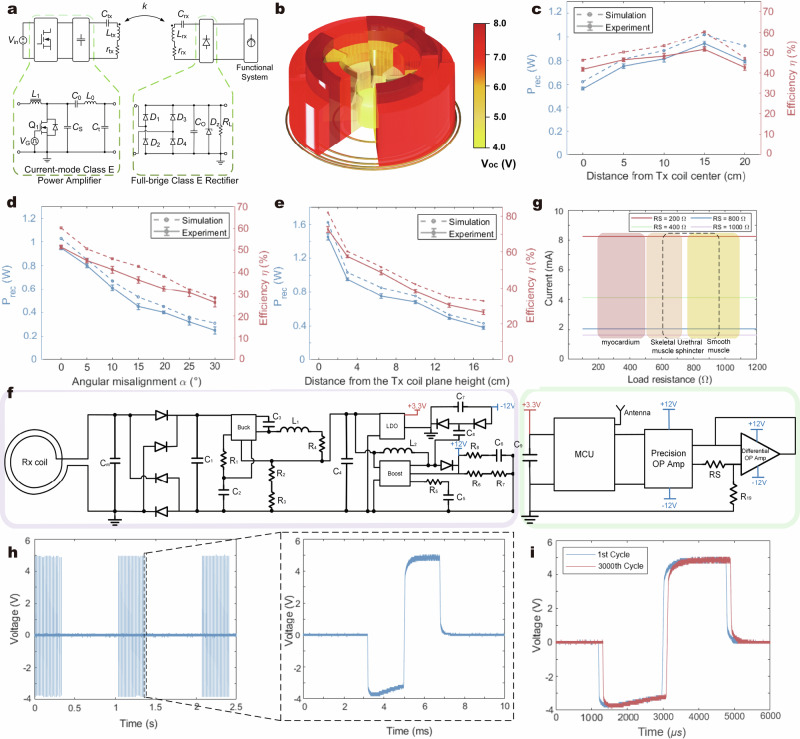
Wireless power transfer (WPT) characteristics of the WIPES. **a** Schematic of the WIPES's power transfer system. **b** 3D representation of the open-circuit voltage (${V}_{{{{\rm{oc}}}}}$) received by the Rx coil at various positions relative to the Tx coil, demonstrating that it has a relatively constant voltage distribution. **c** Variation of the Rx power received (${P}_{{{{\rm{rec}}}}}$, left axis) and transmission efficiency (${\eta }_{{coil}}$, right axis) as a function of horizontal distance from the Tx coil center at a gap of 3 cm. **d** Effect of angular misalignment between the Tx and Rx coils on ${P}_{{{{\rm{rec}}}}}$ and ${\eta }_{{coil}}$, with measurements taken at a horizontal distance of 15 cm from the Tx coil center and a gap of 3 cm. **e** Variation of ${P}_{{{{\rm{rec}}}}}$ (left axis) and ${\eta }_{{coil}}$ (right axis) as a function of gap from the Tx coil plane up to nearly 20 cm at a horizontal distance of 15 cm from the Tx center. For experiments, lines show the mean ± s.d.; *n* = 6 independent technical replicates per x-axis condition, obtained by repeating the WIPES wireless power-transfer measurement (unit of study: one independent WPT measurement run). Simulation results are deterministic (single values). **f** Circuit diagram of the WIPES's implant. **g** Load resistance values for various muscle types, with indicated stable current ranges achieved by using shunt resistance (*R*_s_) in the circuit of the implant. The red, orange, and yellow blocks represent the resistance values of myocardium, skeletal muscle, and smooth muscle, respectively, while the black dashed box represents the resistance values of the urethral sphincter. **h** Measured biphasic pulses with a dual-period configuration, showing the inner (60 Hz) and the envelope (1 Hz) frequencies. **i** Stability assessment of the electrical pulses over 2000 cyclesshowed an average delay of 120 μs.

WPT is fundamental to the operation of the WIPES system, providing a reliable and tetherless power source for its communication and electrostimulation. The WPT is operated at 6.78 MHz and designed for optimal energy transfer (as shown in Figs. 2a and S1), including a Class E power amplifier and a uniform magnetic field generated by a specially designed unequal-gap arrangement of the Tx coil. It ensures stable and continuous power transfer across varying horizontal positions and angles of the Rx coil^38,39^ This is demonstrated by a consistent open-circuit voltage (${V}_{{{{\rm{oc}}}}}$) of the Rx coil at a series of horizontal positions and a gap of 3 cm (Fig. 2b). The impact of the horizontal distance from the Tx center on the received power (${P}_{{{{\rm{rec}}}}}$) and transmission efficiency (${\eta }_{{coil}}$) was measured at a fixed gap of 3 cm, as shown by the blue and red curves in Fig. 2c, respectively. Additionally, the ${P}_{{{{\rm{rec}}}}}$ and ${\eta }_{{coil}}$ were measured when the angular misalignment between the Rx and Tx coils was varied by 30° at a horizontal distance of 15 cm from the center of the Tx coil and at a fixed gap of 3 cm (Fig. 2d). It is also important to analyze ${P}_{{{{\rm{rec}}}}}$ and ${\eta }_{{coil}}$ under a series of gaps from the Tx coil up to near 20 cm at a 15 cm-horizontal distance from the Tx center (Fig. 2e). All these results show that the coupling coefficient remains relatively stable across these displacements of the implant. The activation power threshold of the implantable device is 0.15 W, which could be always satisfied in conditions of various misalignment characterized above.

Figure 2f presents the complete circuit diagram of the implant of the WIPES system, including power management in the purple box and the MCU tunable electrostimulation generator in the green box (refer to the section of “Implantable Device Design” in the “Methods” and Fig. S2 for more details). Given that the impedance of biological tissues can vary, with the typical range for the urethral sphincter between 600 and 950 ohms, the circuit employs a dual op-amp circuit design to ensure stable stimulation current, even with changes in the load resistance (Fig. 2g)^40,41^ The device generates biphasic constant-current pulses with a dual-period configuration (Fig. 2h) including an inner cycle and an envelope cycle. Finally, to demonstrate the system's reliability in maintaining precise timing and consistent pulse delivery over extended periods, the long-term stability of the electrical pulses was evaluated over 2000 stimulation cycles with an average pulse delay of only 120 μs (Fig. 2i). In addition, mechanical reliability tests including cyclic bending, torsion, stretching and interlayer adhesion strength test for WIPES device were conducted to evaluate its durability under deformation (refer to the section of “Mechanical reliability testing of WIPES” in the “Methods” and Figs. S19 and S20). The results confirmed excellent mechanical stability and insulation integrity of the encapsulated electrodes during repeated flexural and tensile loading. Thermal-safety analysis based on coupled HFSS–Icepak simulations (Fig. S22) further confirmed that the device operates within safe temperature limits under its rated power, ensuring stable and biocompatible performance.

### Biosafety verification and ex vivo electrostimulation

**Fig. 3:**
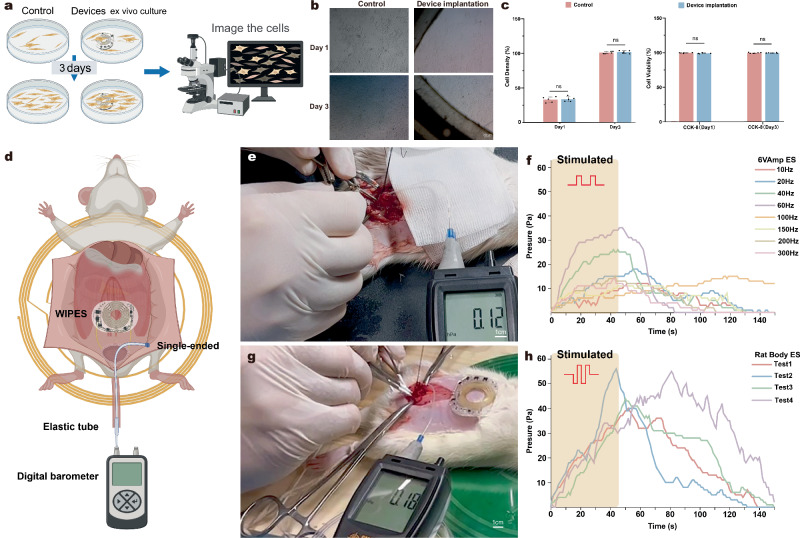
Biocompatibility evaluation of the WIPES and its effectiveness in ex vivo stimulation. **a** Schematic diagram of the experimental setup for assessing biocompatibility with SV-HUC-1 cells for a 3-day co-culture. **b** Microscopic images showing cell density on Day 1 and Day 3 for the WIPES device co-culture group with the control group. **c** Cell density and viability (CCK-8) of Control and Device implantation groups at Day 1 and Day 3. Dots indicate biologically independent cultures (*n* = 5 per group; each point is the mean of 2–3 technical replicate wells); bars show mean ± s.d. Two-sided unpaired t-test; ns (*P* ≥ 0.05). **d** Schematic of the ex vivo experimental setup in anesthetized rats for measuring the pressure of urethral sphincter with a pneumatic manometer. **e** Stimulation setup using monophasic pulses by a function generator. **f** Pressure response of urethral sphincter when stimulated by different frequencies of monophasic pulses. **g** Stimulation setup using biphasic pulses by WIPES. **h** Pressure responses of urethral sphincter when stimulated by biphasic pulses for four continuous trials. Graphical elements in Fig. 3a, d are created in BioRender. Dwad, D. (2026) [https://biorender.com/eknsmzf].

Before proceeding with animal experiments, it is essential to ensure the biosafety of the device and conduct toxicity tests. The device was co-cultured with SV-HUC-1 cells for three days to assess its impact on cell viability and proliferation, as shown in Fig. 3a. Upon microscopy analysis, no notable differences were observed in cell density or morphology between the control and experimental groups (Fig. 3b). The results of the CCK-8 cell viability assay (Fig. 3c) further confirmed that there were no significant changes in cell viability after both 24 h and 3 days of co-culture. These findings indicate that the WIPES implant exhibits excellent biocompatibility, with no adverse effects on cell growth or survival in vitro. Furthermore, electrochemical impedance spectroscopy (EIS) of the WIPES electrodes demonstrated stable interfacial impedance for at least 45 days under physiological conditions, confirming reliable and low-resistance electrode–tissue coupling during long-term operation (refer to the section of “Electrochemical impedance spectroscopy (EIS) of WIPES electrodes” in the “Methods” and Fig. S21).

To explore the optimal parameters for electrostimulation pulses, a series of ex vivo experiments were conducted using anesthetized rats. Following the surgical exposure of the bladder and urethra, a single-blind pneumatic manometer was employed to measure the sphincter pressure in response to varying stimulation conditions. Specifically, electrical pulses were applied at different frequencies and voltages to the region 5 mm below the urethra-bladder junction, with the corresponding changes in pressure being recorded, as schematically illustrated in Fig. 3d. The recorded manometer readings reflect the strength of muscle contractions induced by the applied electrostimulation, allowing indirect measurement of the generated muscle force. The actual pressure generated only by the muscle contraction had to be calculated by a physical model of the elastic tube (refer to the section of “Sphincter Pressure Measurement During Electrostimulation” in the “Methods”). Figures 3e-g and S10 depict two different stimulation modalities for comparison, one is a function generator applying monophasic pulses, and the other is WIPES delivering biphasic pulses. In Fig. 3f, the pressure response to monophasic pulses for a duration of 45 s and an amplitude of 6 V is shown for a series of inner frequencies, with the 60-Hz pulses producing the most pronounced and sustained contraction pressure during the stimulation period (shaded in yellow), highlighting its potential as the optimal stimulation frequency (more data available in Fig. S11). With a 45- s duration, a 1-Hz envelope frequency, a selected optimal 60-Hzinner frequency and a biocompatible biphasic pulse, the pressure curves of four different sets of stimulation trials using WIPES show a continuous increase in urethral sphincter pressure during the electrostimulation period and a gradual reduction after stimulation, as shown in Fig. 3h (45–150 s). This optimized stimulation scheme was adopted for in vivo experiments in the next section.

### In vivo behavioral experiments targeting urinary incontinence in rats

**Fig. 4:**
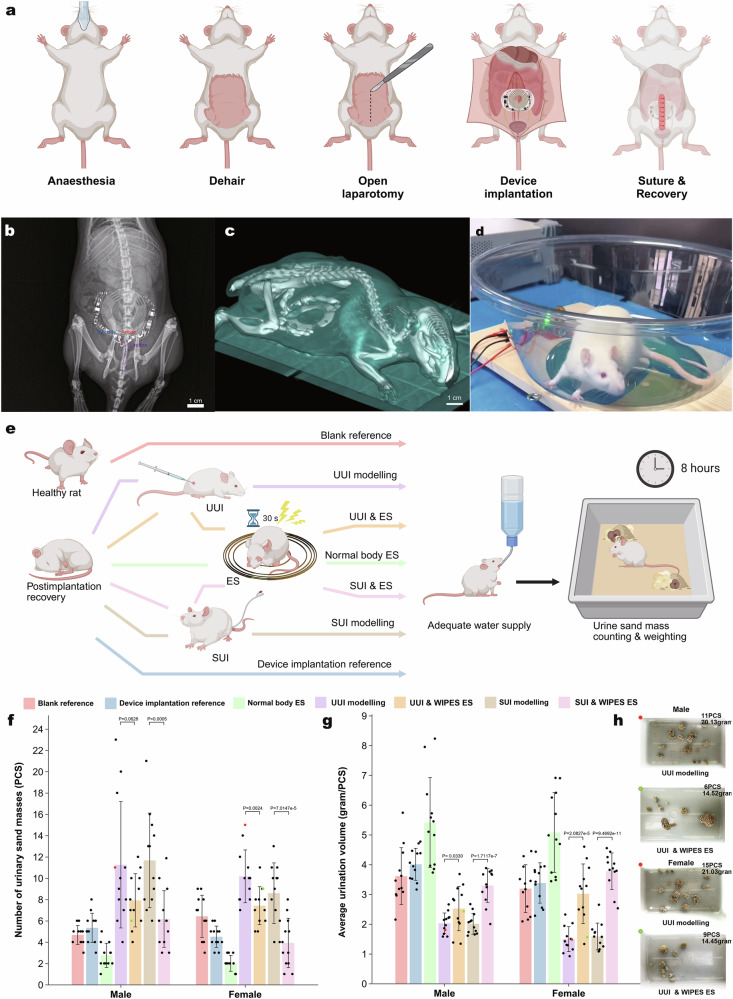
In vivo implantation and efficacy of WIPES for urinary incontinence. **a** Steps of implantation in rats. **b** X-ray image shows the implantable device intact post-implantation. **c** 3D CT reconstruction confirming proper device positioning after recovery. **d** Rat in working Tx coil system during WIPES operation. **e** Experimental design with seven groups: Blank reference, Device implantation reference, Normal body ES, UUI modelling, UUI & WIPES ES, SUI modelling, and SUI & WIPES ES. **f** Number of urinary sand masses (PCS), indicating urination frequency, measured by repeated urine-sand collection (male and female shown separately). **g** Average urination volume (g/PCS), indicating effective bladder capacity, measured by repeated urine-sand collection (male and female shown separately). For **f**, **g**, dots represent individual urine-sand collection measurements (*n* = 12 per group per sex), and multiple measurements may originate from the same rat. To avoid pseudo-replication, repeated measurements were averaged within each rat to yield one value per animal (unit of study: rat; *N* = 8 rats per group per sex) for statistical testing. Bars show mean ± s.d. Statistical analysis was performed within each sex using one-way ANOVA with Tukey's multiple-comparison test; exact Tukey-adjusted P values are indicated above brackets. **h** Urine sand mass samples. The marks of red and green dots represent UUI modelling and UUI & ES groups in Fig. 4f and g, respectively. Graphical elements in Fig. 4a, e are created in BioRender. Dwad, D. (2026) [https://biorender.com/eknsmzf].

The implantation procedure of the WIPES devices followed a clear protocol as shown in Fig. 4a, including anesthesia, hair removal, laparotomy, device placement, closure and recovery in sequential steps. Postoperative care was carefully administered to ensure the animals recover well over 2–3 weeks after the wounds had completely healed and normal activity was resumed (refer to the section of “Surgical Procedure and Device Implantation” in the “Methods”, and Fig. S5 for surgical environment, Fig. S6 for detailed procedures). The rats continued to live and behave normally, with no significant deviations in weight, activity level, or food and water consumption, indicating that the WIPES was well-tolerated. X-ray imaging and computed tomography (CT) with 3D reconstruction confirmed the successful implantation, with all electronic components remaining intact and stable at their designated positions (refer to the section of “Post-implantation X-ray and CT imaging” in the “Methods”; Figs. 4b, c and S7). Figures 4d and S15 illustrate a free-moving healthy rat with an implanted device, which is under electrostimulation therapy above the Tx coil. Additionally, the detailed photograph showing the gold electrodes of WIPES wrapped around the external urethral sphincter is provided in Fig. S27.

To study the therapeutic potential of WIPES-administered electrostimulation in treating urinary incontinence, particularly mixed conditions, a comprehensive behavior-based in vivo evaluation was conducted involving 48 rats (Fig. 4e). The study divided the rats into seven groups, according to categories as healthy rats with no incontinence (1st, Blank reference), device implanted but no stimulation (2nd, Device implantation reference), healthy rats with implanted device followed by electrostimulation (3rd, Normal body ES), urge urinary incontinence with no device implantation (4th, UUI modelling), UUI modelling followed by device implantation and electrostimulation (5th, UUI & WIPES ES), stress urinary incontinence with no device implantation (6th, SUI modelling), and SUI modelling followed by device implantation and electrostimulation (7th, SUI & WIPES ES). Each group comprised eight male rats and eight female rats. After recovery for those with implants, all rats were provided with unrestricted access to drinking water and urination throughout the experiments. The incontinence models were established to separately simulate SUI and UUI, enabling symptom-specific evaluation of WIPES electrostimulation relevant to MUI. Detailed procedures for model establishment are described in the Methods section (“Rat models of stress urinary incontinence” and “Rat models of urge urinary incontinence”). We observed that those rats with induced UI tended to drink more water as they urinate more frequently due to incontinence, which is distinctly opposite to humans as patients with urinary incontinence would consciously limit daily water consumption. In the three groups involving electrostimulation, Normal body ES (3rd), UUI & WIPES ES (5th) and SUI & WIPES ES (7th), rats were subjected to three 30 min daily stimulation sessions, with each consisting of three cycles, with each cycle being 30 s of stimulation followed by a 9.5 min-rest interval. The 30 s stimulation ensures enough stimulation time but no causing damage, while the 9.5 min rest period allows the muscle to recover, ensuring sustained stimulation and consistent therapeutic outcomes at every single session.

Throughout the treatment, urination-related behavior was quantified using the urine sand mass assay, where urine masses were analyzed by collecting and weighing sand clumps every 8 h to assess urination frequency and volume (refer to the section of “Urine Collection Protocol” in the Methods for the special sand used in this work). This behavioral assay enables non-invasive, continuous monitoring of voiding patterns in freely moving animals. Figure 4f shows that the UUI and SUI modelling groups (4th and 6th) exhibited significantly higher urination frequencies, with an increase of 93.21% and 82.71% averaged across male and female groups compared to the Blank reference group (1st), indicating the successful induction of urinary incontinence. Notably, the UUI & WIPES ES group (5th) demonstrated a marked reduction in urination frequency of 28.40% compared to the UUI modelling group (4th), and SUI & WIPES ES group (7th) showed a 50.21% decrease compared to the SUI modelling group (6th). Furthermore, A study on the average urination volume (Fig. 4g, weight of urine mass divided by number of sand clumps), reflects that the UUI & WIPES ES group (5th) and SUI & WIPES ES group (7th), particularly among female rats, showed significant improvements as they reflect clearly increased effective bladder capacity. We note that the improvement observed in male rats in the UUI & WIPES ES group (5th) is less pronounced, likely due to anatomical differences. The male urethra is longer and lies adjacent to the prostate and seminal vesicles, which reduces wrap conformity and stimulation penetration to peri-urethral afferents. Additionally, thicker connective tissue and pelvic geometry may attenuate current density at the target, resulting in lower and more variable therapeutic response compared with females. Figure 4h visually shows representative sand clumps collected from male and female rats in the UUI modelling group (4th) and the UUI & WIPES ES group (5th), respectively. Based on statistics comparing WIPES-treated individuals to average UI modeled behavior, the UUI & WIPES ES group (5th) showed reduced urination frequency and increased average urination volume compared to the UUI modelling group (4th) with an average alleviation rate of 90.62%. Similarly, the SUI & WIPES ES group (7th) exhibited comparable improvements compared to the SUI modelling group (6th), achieving an average therapeutic efficacy of 97.92%, clearly showing a significant advantage over the ~50% efficacy in existing methods (refer to the section of “Data Analysis” in the Methods for a detailed calculation procedure). From a behavioral perspective using the urine-sand collection assay, the therapeutic effects of traditional sacral nerve stimulation (SNS) on both UUI and SUI were evaluated, as shown in Fig. S26. Representative urine-sand samples from female and male rats in the UUI modelling group (4th) and the UUI & WIPES ES group (5th) are shown in Fig. 4h, with additional samples provided in Fig. S16.

### Evaluation of WIPES therapy in urge urinary incontinence models

**Fig. 5:**
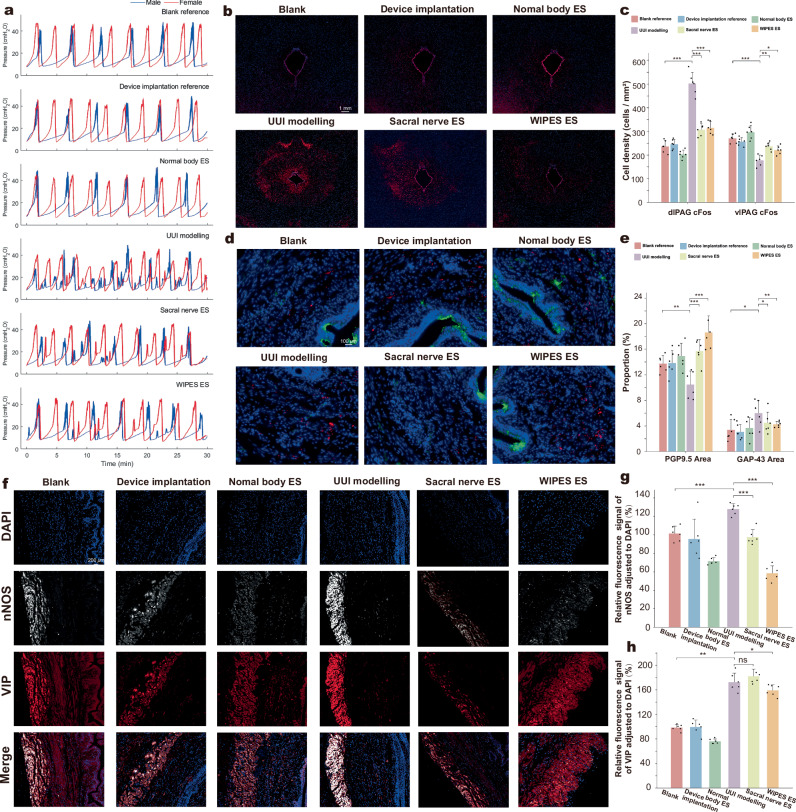
WIPES improves bladder function and is associated with neural remodeling in rats with urge urinary incontinence. **a** Representative cystometric traces of male and female rats under different conditions, including Blank reference (1st), Device implantation reference (2nd), Normal body ES (3rd), UUI modelling (4th), Sacral nerve ES (5th), and WIPES ES (6th) groups. **b** c-Fos immunostaining images of the dorsal and ventrolateral periaqueductal gray (dPAG and vlPAG) regions in rats from each group. **c** Statistical analysis of c-Fos–positive cell densities in dPAG and vlPAG regions. **d** Immunofluorescence staining images showing PGP9.5 (green) and GAP-43 (red) expression in urethral sphincter tissues of rats in each group, the green fluorescence indicates the expression of PGP9.5, and red fluorescence indicates the expression of GAP-43. **e** Statistical analysis of PGP9.5-positive area and GAP-43 area in rats of all groups. **f** Immunofluorescence staining of nNOS and VIP neurotransmitters in urethral sphincter tissues of rats in each group. **g** Quantitative comparison of nNOS fluorescence intensity normalized to DAPI for all groups. **h** Quantitative comparison of VIP fluorescence intensity normalized to DAPI for all groups. For (**c**, **e**, **g** and **h)**, dots represent biologically independent rats (*n* = 6 per group); for each rat, measurements from multiple sections were averaged to yield one data point (unit of study: rat). Data are shown as mean ± s.d. Statistical analysis was performed using one-way ANOVA with Tukey's multiple-comparison test (two-sided); significance is indicated by brackets (ns, *P* ≥ 0.05; *P < 0.05; **P < 0.01; ***P < 0.001).

This section focuses on the results of UUI validation, as summarized in Fig. 5. The cystometric analysis (Fig. 5a) provides a quantitative assessment of bladder overactivity and storage dysfunction in the UUI model by measuring intravesical pressure changes over time (refer to the section of “Urodynamic Evaluation” in the “Methods”, and Fig. S28). Three key parameters—non-voiding contraction (NVC) frequency, inter-contraction interval (ICI), and bladder capacity—were analyzed to characterize detrusor muscle function^42,43^ Rats in the UUI modelling group exhibited abnormally frequent NVCs and markedly shortened ICIs, indicating excessive and unstable detrusor activity associated with urge incontinence. The reduced bladder capacity further confirmed the loss of coordinated neural control and premature triggering of voiding reflexes. In contrast, WIPES electrostimulation (WIPES ES) effectively normalized these parameters. The frequency of NVCs was significantly reduced, while the ICI was prolonged to levels comparable to healthy controls, reflecting improved detrusor stability and improved bladder compliance. Meanwhile, the measured bladder capacity increased markedly, suggesting that the WIPES stimulation enabled fuller bladder filling before the initiation of micturition, consistent with improved regulation of bladder storage function. The SNS group also showed comparable improvement, with slightly more irregular NVCs. Additionally, representative, time-aligned cystometry and EUS-EMG recordings across both UUI and SUI models, are provided in Supplementary Fig. S32 for reference.

To further investigate how WIPES modulates central neural circuits involved in micturition control, c-Fos immunofluorescence staining was performed in the dorsal periaqueductal gray (dlPAG) and ventrolateral periaqueductal gray (vlPAG) regions of the midbrain (Fig. 5b and c) (refer to the section of “Quantification of c-Fos for urge-suppression assessment” in the “Methods”). The PAG is a pivotal integrative hub linking bladder sensory input and pontine motor output. Specifically, the dlPAG primarily participates in urine storage and inhibitory control, suppressing the activation of the micturition reflex, whereas the vlPAG is associated with voiding facilitation, relaying excitatory signals to the pontine micturition center (PMC)^44,45^ Thus, the spatial distribution and intensity of c-Fos expression in these two regions provide insight into the excitatory–inhibitory balance within the central micturition network.^43^ In the UUI modelling group (4th), excessive bladder afferent input caused by detrusor overactivity aberrantly activated the dlPAG, leading to an overcompensated inhibitory response attempting to suppress involuntary voiding. Quantitatively, c-Fos expression in the dlPAG increased by 112.81% compared to the blank reference (1st), confirming pronounced hyperactivation of this inhibitory center. In contrast, the vlPAG showed a 34.5% reduction in c-Fos activity, indicating suppression of the voiding-facilitatory pathway and disruption of the normal excitatory relay to the PMC. Such imbalance between facilitation and inhibition underlies the detrusor overactivity characteristic of urge incontinence. Following WIPES ES group (6th), this imbalance was markedly corrected. c-Fos expression in the dlPAG decreased by 37.16%, while the vlPAG exhibited a 33.77% increase, indicating a bidirectional normalization of inhibitory and excitatory activities within the central micturition network. Similarly, SNS ES group (5th) produced comparable effects, with dlPAG activity reduced by 38.86% and vlPAG elevated by 33.78%. These results suggest that both WIPES and SNS treatments effectively rebalanced the dlPAG–vlPAG circuitry, and from the perspective of impulse inhibition, their restorative efficacy showed no significant difference. (Mean ± SD for N = 8 rats; ***P < 0.001, **P < 0.01, *P < 0.05).

At the central level, c-Fos mapping revealed altered activity within brainstem regions associated with urination following WIPES electrostimulation, supporting central engagement in response to peripheral stimulation. To further examine the peripheral outcomes of this neuromuscular interface effect, we evaluated nerve regeneration within the urethral sphincter region and synaptic recovery using PGP9.5 and GAP-43 immunofluorescence markers (green and red, Fig. 5d and e). PGP9.5 serves as a pan-neuronal marker reflecting the overall density and integrity of peripheral nerve fibers, whereas GAP-43 is a growth-associated protein expressed during axonal regeneration, indicating active neural remodeling and repair (refer to the section of “Neural Immunostaining & Quantification” in the “Methods”)^46,47^ As shown in Fig. 5e, the PGP9.5 Area represents the proportion of PGP9.5-positive regions within the total tissue area, quantifying the nerve fiber density. Compared with the blank reference (1st), the UUI modelling group (4th) showed a 23.92% reduction, indicating marked nerve degeneration and fiber loss. Following treatment, the SNS ES group (5th) exhibited a 50.53% increase relative to the model group, reflecting partial recovery characterized by discontinuous and loosely organized fibers. In contrast, the WIPES ES group (6th) demonstrated a 78.43% increase in PGP9.5 Area, showing densely packed and continuous neural structures, indicative of robust peripheral nerve restoration. To further assess regenerative activity, GAP-43—an established marker of axonal sprouting and early-stage neural remodeling—was quantified across all groups. The UUI modelling group (4th) exhibited a markedly elevated GAP-43 signal, showing a 77.54% increase compared with the blank reference, indicating extensive but disorganized compensatory sprouting of immature axons following nerve injury. In contrast, the SNS ES group (5th) reduced GAP-43 expression by 25.06%, and WIPES ES group (6th) by 28.99%, relative to the UUI model, suggesting that electrical stimulation helped suppress excessive, non-functional sprouting while promoting a more stabilized and orderly regeneration environment. These results support that WIPES facilitates structured neural recovery rather than chaotic axonal overgrowth. Notably, WIPES treatment normalized the GAP-43 Index to a moderate yet orderly level while significantly increasing the PGP9.5 Area, suggesting that WIPES promoted organized axonal remodeling and markers associated with reinnervation, rather than chaotic overgrowth. This coordinated pattern suggests an association between WIPES stimulation and increased nerve density with improved morphological organization of regenerated fibers (Mean ± SD for *N* = 8 rats; ***P < 0.001, **P < 0.01, *P < 0.05).

Furthermore, to elucidate the neuroregulatory mechanisms responsible for the functional recovery observed in cystometric profiles, we  selected two markers, nNOS and VIP, to evaluate the growth and function of nerve cells around the urethra (refer to the section of “Immunofluorescence Staining“ in the Methods). nNOS is a neurotransmitter in urethral nerve cells that induces smooth muscle relaxation and mediates nerve injury and inflammatory pain hypersensitivity^48,49^ while VIP reduces neuroinflammation and promotes early myelination and growth of regenerative axons in the nervous system (Fig. 5f and h)^50^. In the Blank reference (1st), nNOS and VIP were distinctly expressed in organized nerve fibers surrounding the urethral sphincter and bladder wall, ensuring synchronized activation of excitatory and inhibitory pathways that maintain stable micturition cycles. In contrast, rats subjected to UUI modelling (4th) exhibited diffuse and disordered nNOS overexpression, accompanied by irregular VIP distribution and reduced spatial colocalization, with increases of 26.73% (nNOS) and 75.13% (VIP) (Mean ± SD for *N* = 8 rats; ***P < 0.001, **P < 0.01). Such aberrant patterns indicate a breakdown in inhibitory control and overactivation of parasympathetic efferents, consistent with the frequent, high-amplitude NVCs observed in cystometric recordings. Following SNS ES (5th) and WIPES ES (6th) electrostimulations, nNOS signals recovered toward physiologically balanced patterns, with a decrease of 23.67% and 54.30%, and VIP levels have different expressions, with a slight increase (Mean ± SD for *N* = 8 rats; ns, not significant) and a decrease of 8.03%. The normalized nNOS distribution suggests recovery of nitric oxide–related inhibitory signaling associated with suppression of premature detrusor contractions, while the orderly re-expression of VIP is consistent with improved neurovascular and mucosal relaxation–related signaling. These features collectively reflect the functional reconnection of afferent–efferent feedback loops rebuilt through WIPES-induced peripheral reinnervation. By contrast, SNS treatment led to partial normalization, with residual nNOS hyperexpression and sparse VIP regions, suggesting incomplete restoration of inhibitory control.

In summary, cystometric and neurobiological analyses collectively suggest an association between WIPES stimulation and altered peripheral–central signaling relevant to bladder control. The system is associated with altered activity within brainstem regions implicated in micturition, alongside orderly peripheral nerve remodeling, as evidenced by increased PGP9.5 density and normalized GAP-43 expression. Concurrent changes in nNOS and VIP expression further support modulation of excitatory–inhibitory signaling relevant to bladder–sphincter coordination.

### Evaluation of WIPES therapy in stress urinary incontinence models

**Fig. 6:**
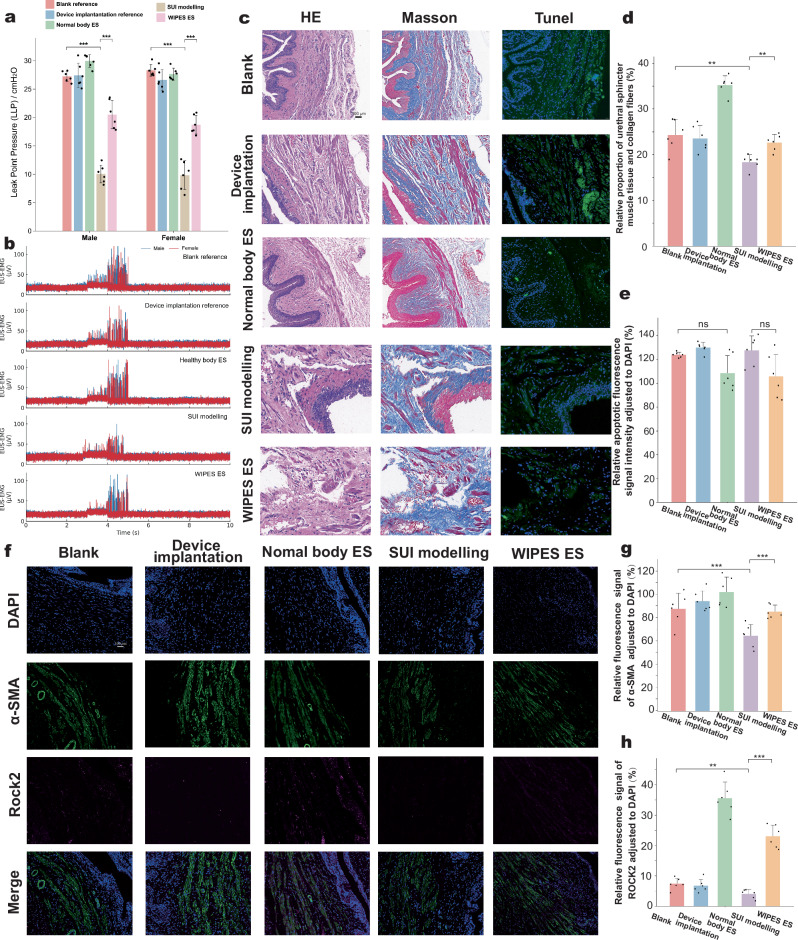
WIPES Promotes Functional and Morphological Recovery of the Urethral Sphincter in Stress Urinary Incontinence Rats. **a** Leak point pressure results for five groups—Blank reference (1st), Device implantation reference (2nd), Normal body ES (3rd), SUI modelling (4th), and WIPES ES (5th)—showing significant LPP recovery after WIPES stimulation in both male and female rats. **b** Representative external urethral sphincter electromyography (EUS-EMG) recordings of all groups. **c** Histological evaluation of urethral sphincter tissues using H&E, Masson, and TUNEL staining for all groups. **d** Quantitative analysis of the relative proportion of urethral sphincter muscle to collagen area for all groups. **e** Quantification of TUNEL fluorescence signal intensity the in five groups. **f** Immunofluorescence staining of α-SMA (green) and ROCK2 (magenta) in urethral sphincter tissues, with DAPI (blue) indicating nuclei for all groups. **g** Quantitative analysis of α-SMA signal intensity normalized to DAPI for all groups. **h** Quantitative comparison of ROCK2 fluorescence intensity normalized to DAPI for all groups. For (**a**, **d**, **e**, **g** and **h**), dots represent biologically independent rats (*n* = 6 per group); for each rat, measurements from multiple sections were averaged to yield one data point (unit of study: rat). Data are shown as mean ± s.d. Statistical analysis was performed using the one-way ANOVA with Tukey's multiple-comparison test (two-sided); significance is indicated by brackets (ns, *P* ≥ 0.05; *P < 0.05; **P < 0.01; ***P < 0.001).

We next validated the performance in SUI rats to further assess their broad neuromuscular restoration potential. Cystometric measurements and leak point pressure (LPP) testing (Fig. 6a; refer to the section of “Urodynamic Evaluation” in the Methods) revealed a pronounced reduction in LPP in rats subjected to SUI modelling (4th) compared with the Blank group (1st), with a decrease of 64.24%, indicative of compromised urethral closure competence and weakened sphincteric resistance to bladder pressure^51^.^52^ The decreased LPP reflects impaired transmission of contractile force within the external urethral sphincter (EUS), resulting in premature urine leakage upon elevated intravesical pressure. Following WIPES ES group (5th), both male and female rats exhibited a substantial recovery of LPP values, with an increase of 97.90%, approaching those of the healthy baseline, demonstrating the efficacy of electrical stimulation in restoring urethral outlet resistance (Mean ± SD for *N* = 8 rats. ***P < 0.001). Concurrently, electromyographic recordings from the EUS (EUS-EMG, Fig. 6b) revealed characteristic alterations in neuromuscular activity patterns.^53^ In SUI-modelled rats, the EMG traces showed sparse, low-amplitude, and poorly coordinated bursts during voiding, reflecting disrupted neuromuscular communication and reduced sphincter excitability. In contrast, WIPES-treated animals exhibited rhythmic, high-frequency spike bursts synchronized with the micturition cycle, indicative of reinstated reflex control and functional re-engagement of motor units within the sphincter. These findings collectively support that WIPES stimulation enhances urethral closure pressure mechanically, as evidenced by LPP improvement, and is associated with improved neuromuscular coordination relevant to continence regulation.

In this study, H&E staining was used to verify the integrity of the sphincter tissue, and Masson staining was used to assess the musculature-to-collagen ratio, and lastly these data were combined to comprehensively evaluate the health status of the urethral musculature and the therapeutic effect, as shown in Fig. 6c. Compared to the Blank reference group (1st), the smooth muscle-to-collagen ratio showed a significant decrease of 24.28% in the SUI modelling group (4th) (Mean ± SD for *N* = 8 rats. ***P < 0.001). Interestingly, the smooth muscle volume in the WIPES ES group (5th) showed significant improvement, with an increase of 23.14%, compared to the SUI modelling group (4th) (Mean ± SD for *N* = 8 rats. *P < 0.05) as shown in Fig. 6d. These results suggest that WIPES stimulation is associated with enhanced smooth muscle proliferation and improved functional outcomes, approaching levels observed in healthy controls. The histological findings align with the functional evaluations described earlier, supporting the potential of WIPES to ameliorate SUI-related functional deficits. In addition, TUNEL staining was employed as an effective method to demonstrate apoptosis in vivo^54^. The green fluorescence intensity indicated the distribution of DNA associated with programmed cell death in tissues (Fig. 6c). There were no significant differences in green fluorescence intensity among all groups, indicating that the experiments did not induce apoptosis in urethral tissues.

To further investigate the molecular mechanisms underlying tissue recovery, immunofluorescence staining of α-SMA and ROCK2 was conducted (Fig. 6f–h). α-SMA is an actin protein specifically expressed in smooth muscle, forming the core part of muscle cells and maintaining contractile capacity^55,56^. ROCK2, a downstream effector of Rho kinase, regulates the proliferation and differentiation of cytoskeletal proteins in smooth muscle cells and is a critical factor in promoting smooth muscle contraction^57,58^ (refer to the section of “Immunofluorescence Staining“ in the Methods). It showed a clear distribution of α-SMA in all groups, indicating the structural and functional integrity of the urethral smooth muscle. Compared with SUI modelling group (4th), the WIPES ES group (5th) showed a 32.63% increase in α-SMA signals  (Fig. 6f). On the other hand, for ROCK2, stimulation significantly enhanced the ROCK2 signals in the WIPES ES group (5th) compared with the SUI modelling group (4th), with an increase of 471.46%, with ROCK2 primarily localized around the urethral smooth muscle cells (Mean ± SD for *N* = 8 rats; **P < 0.01, ***P < 0.001), as shown in Fig. 6h. We note that the induced incontinence results from nerve inflammation and damage, along with the overexpression of relaxant neurotransmitters. Based on the observed molecular and histological changes, WIPES stimulation may influence urethral tissue remodeling through modulation of neurotransmitter-associated markers, axonal remodeling, and ROCK2-related signaling pathways to enhance the proliferation and differentiation of smooth muscle cells, thereby contributing to functional improvement relevant to SUI.

In summary, WIPES stimulation effectively improves urethral function in SUI models by reinforcing  the sphincteric contractility and supporting improved coordination between detrusor and sphincter activity. Histological and molecular analyses further support enhanced smooth muscle regeneration, improved muscle-to-collagen balance, and activation of the ROCK2 pathway, indicating both mechanical strengthening and cellular remodeling of the urethral musculature toward full continence recovery.

## Discussion

We have shown that the WIPES system enables wireless, implantable electrostimulation that is associated with significant improvement of urinary incontinence–related functional outcomes in freely moving rat models. Comprehensive urodynamic assessments revealed functional improvements in bladder and urethral performance consistent with enhanced lower urinary tract control during micturition. Ex vivo experiments were conducted to survey optimal pulse parameters that generated the highest and most sustained urethral pressure. The efficacy of WIPES stimulation was further evaluated in vivo, where treated rats exhibited reduced urination frequency and increased voided volume. Histological and immunofluorescence analyses further revealed enhanced smooth muscle organization and neural remodeling within the urethral sphincter, accompanied by altered expression of neurotransmission-related markers. Together, these findings support an association between WIPES stimulation and concurrent functional improvement and tissue-level remodeling of the urethral sphincter, highlighting its potential as a neuromodulation-based platform for treating urge, stress, and mixed urinary incontinence.

When viewed in the context of existing state-of-the-art neuromodulation strategies for urinary incontinence, these findings help clarify the distinct positioning of WIPES. Established implantable neuromodulation systems and miniaturized wireless stimulators typically derive benefits from neural modulation or acute increases in urethral pressure, whereas non-invasive approaches modulate bladder activity indirectly through distal neural pathways. In contrast, the present results demonstrate that localized, sphincter-level electrostimulation can simultaneously yield sustained functional improvements and tissue-level remodeling at the urethral outlet. This combined functional and structural response differentiates WIPES from approaches that primarily target neural signaling or short-term functional endpoints alone. By employing a conformal, battery-free interface directly at the peri-urethral neuromuscular junction, WIPES addresses both outlet competence and urge-related dysfunction within a single local site, thereby complementing existing implantable and non-invasive therapies rather than replacing them.

Application in humans requires engineering enhancements to ensure lifelong reliability and safety, such as capping the highest possible currents in the Rx coil and the stimulation pulses. The long-term stability of the elastic protection layer and the electronics is subject to further analysis, particularly their lifetime as an implant inside the human body. In addition, the clamping of the pulse delivery wires around the urinary sphincter needs rigorous engineering design to ensure that they are always at the correct location while not compressing the sphincter, even under intense body motions. In future translational studies, extended-duration implantation and large-animal models will be essential to systematically evaluate long-term detrusor function, upper urinary tract safety, and the physiological consequences of sustained modulation of urethral outlet resistance. Another critical issue is the safety of body tissues subject to magnetic field of WPT, which has been analyzed by a preliminary simulation study as provided in Table 1 and the section of Specific Absorption Rate of Electromagnetic Power in the Methods. Finally, the differences in human urinary anatomy compared with those in rats and between the two sexes also requires comprehensive research to ensure proper implantation and suitable stimulation plan. Although these extensive engineering topics are required, WIPES provides a promising ease-of-use value in relieving mixed or unclear types of urinary incontinence.

**Table 1:** Main tissue properties for the HUGO model at 6.78 MHz

Tissue	Relative permittivity	Conductivity (s/m)	Weight density (kg/m)
Brain (grey matter)	208.76	0.385	1035.5
Heart Muscle	231.48	0.664	1059
kidney	336.39	0.637	1147
Liver	204.94	0.372	1151
Lung	103.63	0.207	563
Spleen	628.03	0.367	1059
Muscle (Across)	194.49	0.631	1059
Muscle (Along)	120.92	0.553	1059
Uterus	331.91	0.593	1057
Skin	228.16	0.368	1125
Thyroid	246.49	1.118	1035.5
Testis	387.67	0.929	1059
Ovary	356.65	0.461	1050
Bladder	74.46	0.25	1120
Tongue	222.07	0.501	1050
Adipose Tissue	9.608	0.0234	916
cartilage	266.64	0.338	1115
Bone	42.48	0.046	1080

## Methods

### Implantable device design

The implant of the WIPES system is designed with a multilayer structure, including an elastic packaging layer, an electronics layer, a polyimide (PI) layer with gold coils, and electrostimulation wire electrodes. The receiving coil (Rx) picks up energy from alternating magnetic flux due to mutual induction. We have selected an 8-turn circular coil with a 1.5:1 wire width-to-spacing ratio as the Rx coil, which is based on several design considerations, optimized through High Frequency Structure Simulator (HFSS) simulations. Initially, the coil configuration was chosen to balance a compact form factor with sufficient induced current, ensuring stable wireless power reception. Through a series of HFSS simulations, the optimal number of turns and wire width-to-spacing ratio were iteratively refined. The 8-turn configuration was found to provide an ideal balance between size and energy efficiency, while the 1.5:1 wire width-to-spacing ratio optimized the magnetic field distribution and minimized electromagnetic interference. The simulations also indicated that the circular shape enhances the uniformity and symmetry of the magnetic field, allowing the coil to maintain high energy reception across different orientations. This detailed design and optimization process can be included in Figs. S3 and S4.

The energy reception by Rx is followed by the power management subsystem (PMS) in the electronics layer, which includes a Buck converter, an LDO regulator, and a BOOST converter. The Buck converter stabilizes the DC output power received from the Rx coil and rectifier circuit. The LDO regulator steps down the voltage to 3.3 V to power the microcontroller unit (MCU). The BOOST converter increases the voltage to ±12 V to provide the operating voltage for the subsequent dual operational amplifier (op-amp) stage. After conditioning the received power by the above electronic components, it is used to provide energy for the MCU, stimulation generator module, and Wi-Fi module. When receiving external execution commands transmitted via Wi-Fi, the MCU sends a signal to the stimulation generator to produce customized electrostimulation pulse patterns. The MCU coordinates the operation of dual op-amps to adjust the frequency and amplitude of the bidirectional pulses, enabling the desired variation. The dual op-amps are critical for maintaining consistent and stable stimulation pulse waveforms, compensating for the varying electrical resistances encountered when interfacing with different muscle tissues or when muscle morphology changes. Despite resistance fluctuations, the op-amps ensure that the output current remains constant, preserving the effectiveness and consistency of the stimulation.

### Device fabrication process

The process begins by selecting a 75 μm-thick transparent polyimide (PI) film, with 35 μm copper foil attached on both sides. A 13 μm layer of photoresist (AZ-5214E) is applied to the copper foil and soft-baked at 75 °C. The surface of the photoresist is then exposed to ultraviolet (UV) light. After exposure, the photoresist is developed in solution (AZ-300MIF), removing the unexposed portions and revealing the circuit pattern on the copper foil. The exposed copper areas are then etched using ferric chloride (FeCl₃), which dissolves the unprotected copper, leaving behind the desired circuit pattern. After etching, the flexible circuit board is thoroughly rinsed with deionized water to remove any remaining etchant. The surface is then activated using a gold chloride solution (HAuCl₄) and immersed in a cyanide gold electroplating bath (KAu(CN)₂), where plating occurs at a current density of 1 A/dm² to achieve a gold deposition thickness of 4 μm. Once the gold plating is complete, electronic components are carefully placed onto the circuit traces using component leads and soldered in place with low-temperature solder. Finally, 35 μm gold-plated wire electrodes are connected to the flexible circuit board, and the entire circuit is encapsulated using PDMS (Sylgard 184 with a 10:1 base-to-curing agent ratio) to ensure that all surfaces are sealed and protected except for the section contacting the urethral sphincter.

### Electrochemical impedance spectroscopy (EIS) of WIPES electrodes

EIS was performed to characterize the electrode–tissue interface and evaluate the long-term electrochemical stability of the WIPES electrodes during implantation. Measurements were carried out at different time points (1, 7, 14, 30, and 45 days) using a potentiostat/impedance analyzer (Gamry Reference 600 + , USA). A three-electrode setup was adopted, with the WIPES gold electrode as the working electrode, a Pt wire as the counter electrode, and an Ag/AgCl electrode as the reference. At the same time, a partial segment of the rat urethral sphincter was excised, wrapped by the gold electrode, and maintained in culture medium (DMEM/F-12) as an ex vivo construct to mimic the implanted configuration. At each time point, the electrode–tissue construct was connected to the measurement system for EIS. The sinusoidal excitation signal was 10 mV rms, and the frequency range was 0.1–10,000 Hz. The frequency range of 0.1 Hz–10 kHz was chosen to comprehensively evaluate electrode–tissue interactions. Low frequencies assess interfacial charge transfer and encapsulation, mid frequencies (including the 60 Hz operating point of WIPES) capture therapeutic impedance, and high frequencies reflect  the bulk solution resistance.

The obtained impedance spectra were fitted with a standard Randles-type equivalent circuit consisting of: ${R}_{{{{\rm{ct}}}}}$ (charge transfer resistance) reflects the electron-transfer kinetics at the gold–tissue interface. A significant reduction of ${R}_{{{{\rm{ct}}}}}$ may indicate increased electrochemical reactions, possible electrode degradation, or tissue inflammatory response, while stable ${R}_{{{{\rm{ct}}}}}$ suggests preserved electrode integrity. CPE (constant phase element): models the non-ideal capacitive behavior of the electrode–tissue interface, accounting for surface roughness and heterogeneity. A decreasing CPE exponent (n) over time suggests interface irregularity or fibrotic encapsulation. Stable ${R}_{{{{\rm{ct}}}}}$ and CPE parameters indicated minimal gold electrode corrosion and a robust electrode–tissue coupling over the chronic implantation period.

### Mechanical reliability testing of WIPES

To validate the long-term mechanical stability of WIPES under repetitive deformations resembling physiological motion, two types of tests were performed. Cyclical bending and twisting tests: The device was mounted on a custom motorized stage capable of applying controlled forward–reverse angular displacements. For bending, the device was fixed at both ends and subjected to repeated deflections along its long axis, while for twisting, one end of the device was fixed and the opposite end was rotated alternately in clockwise and counterclockwise directions. Each test was performed for 1000 continuous cycles at a frequency of 0.2 Hz. During deformation, the electrical stimulation output of WIPES was monitored in real time using an oscilloscope to record pulse amplitude and waveform fidelity. Variations in pulse amplitude after 1000 cycles were analyzed to quantify electrical stability under mechanical strain (As shown in Fig. S19).

Interlayer adhesion strength test: To assess the bonding robustness between the PDMS adhesive layer and PI substrate, a 180° peel test was performed according to ASTM D903 standard. The PI layer was clamped to a motorized pulling stage and stretched at a constant rate of 30 mm/min, while the PDMS layer was secured to a digital force gauge oriented in the opposite direction. The force–time curve was continuously recorded during peeling. Transient decreases in force corresponded to regions of reduced adhesion width due to the central hollow geometry of the device, followed by recovery as the peel front passed the narrower sections. The maximum and average peel forces were extracted to characterize interfacial adhesion energy and structural integrity (As shown in Fig. S20).

### Biphasic pulse design

The WIPES delivers biphasic pulses with an inner frequency of 60 Hz and an outer frequency of 1 Hz. This dual-period stimulation design ensures effective muscle contraction and relaxation cycles, optimizing therapeutic outcomes. The rationale for this dual-period system stems from extensive experimental observations. First, for the inner Frequency of 60 Hz, this frequency is chosen to match the natural contraction frequency of muscle fibers, ensuring that each pulse can effectively stimulate the muscle. It provides a rapid series of contractions that help strengthen the muscle fibers over time (as shown in Figs. 3f and S11). Second, for the outer Frequency of 1 Hz, this slower frequency controls the overall pacing of the stimulation sessions. It allows for sufficient relaxation periods between the faster inner pulses (as shown in Fig. S13). The dual-period system has been shown in experiments to balance efficacy and safety. By providing high-frequency pulses in short bursts followed by longer intervals of rest, the system can maximize muscle training benefits while minimizing adverse effects such as muscle soreness or damage. In rat models, this approach has effectively improved muscle strength and function without causing undue stress or pain, indicating its potential for safe and effective therapeutic use in humans. The stimulation was validated in an ex vivo gastrocnemius preparation, as shown in Fig. S8.

### Sphincter pressure measurement during electrostimulation

To measure the pressure exerted by the urethral sphincter during stimulation, a manometer is utilized, which operates with a sealed volume of air inside a silicone tube. This tube is inserted through the rat's urethra. When the urethral sphincter contracts due to electrostimulation, it compresses the silicone tube and the enclosed air, leading to changes in air pressure that the manometer detects. This setup allows for the calculation of the pressure changes in the urethral sphincter. The pressure generated by the urethral sphincter is measured using a differential pressure manometer connected to a single-ended plastic catheter. The catheter has a length of *l* = 13 cm, an outer diameter ${d}_{{od}}$=1 mm and an inner diameter ${d}_{{id}}=$ 0.5 mm. The catheter, sealed with air, is inserted through the rat's urethra into the bladder. During electrostimulation, the urethral sphincter contracts and compresses the catheter, increasing the internal pressure, as shown in Fig. S12. To calculate the initial elastic resistance pressure ${P}_{r}$ of the silicone tube, the following formula is used

1 $${{{\rm{P}}}}_{r}=\frac{E}{1-{v}^{2}}\left(\frac{{d}_{{od}}-{d}_{{id}}}{{d}_{{od}}+{d}_{{id}}}\right).\,{\Delta}$$

where *E* = 12 *MPa* (Young's modulus), v = 0.49 (Poisson's ratio), and Δ = 0.001% (radial deformation, which is depended on the length deformation and radial deformation of the elastic silicone tube).

Given that the deformed section length $\Delta \,l=1.3{cm}$ is 10% of the total length, and the radial deformation is about 1%, the elastic resistance ${P}_{r}$ is calculated to be 52.64 Pa. When the urethral sphincter contracts, it exerts a pressure $\Delta {P}_{1}$ overcoming the elastic resistance ${P}_{r}$, leading to an increase in the internal air pressure $\Delta {P}_{2}$. Assuming negligible volume change due to the small deformation, Boyle's Law (${P}_{1}{V}_{1}={P}_{2}{V}_{2}$) for ideal gases can be applied. Given that the initial conditions are at one standard atmosphere, $\Delta {P}_{1}=\Delta {P}_{2}$. Therefore, the pressure generated by the urethral sphincter after stimulation, ${P}_{A}$, is:

2 $${P}_{A}={P}_{r}+{\Delta P}_{1}.$$

This setup and calculation method effectively validate the pressure changes in the urethral sphincter induced by stimulation during open abdominal experiments on rats (Fig. S9). Additionally, histological examination confirmed that external urethral intubation did not cause detectable structural damage to the urethral sphincter, as evidenced by the intact cross-sectional morphology shown in Fig. S31.

### WPT simulation and efficiency calculation

Ansys HFSS simulation for optimizing the transmission efficiency involved key steps, including material selection, coil design, and excitation settings. Copper was used as the coil material due to its high conductivity. The Tx and Rx coil geometries were defined in HFSS, considering parameters such as coil diameter, number of turns, and wire width-to-spacing ratio. An a.c. excitation at 6.78 MHz was applied to the Tx coil to simulate real-world operation. The surrounding biological tissue environment was modeled as a dielectric with typical tissue permittivity and conductivity to reflect in vivo conditions. Multiple simulation scenarios were tested, varying coil alignment, spacing and tissue effects. The results guided optimization of coil configuration and placement to improve transfer efficiency while minimizing losses and interference.

The WPT transmission efficiency is calculated using the following formula.^59^. The coupling coefficient (*k*) is an important parameter in the transmission process of WPT. Impedance variations resulting from alterations in the *k* detune the resonance of the coupling coil and impair the performance of the power amplifier, so it will greatly affect the transmission efficiency between WPT systems:

3 $$k=\frac{M}{\sqrt{{L}_{{tx}}{L}_{{rx}}}}$$

where *M* is the mutual inductance between the coils, and ${L}_{{tx}}$ and ${L}_{{rx}}$ are the inductance of the Tx and Rx coil (Figure S17). For coupling coils, the power loss occurs on the parasitic resistances, i.e. ${r}_{{tx}}$ and ${r}_{{rx}}$. A T-shaped matching network is used to maintain a high mutual inductance between Tx and Rx as positions and orientations change. Thus, the efficiency of a pair of Tx and Rx coils, ${\eta }_{{coil}}$, can be expressed as

4 $${\eta }_{{coil}}=\frac{{R}_{{rec}}}{(1+\frac{{r}_{{tx}}{r}_{{rx}}+{r}_{{tx}}{R}_{{rec}}}{{\omega }^{2}{k}^{2}{L}_{{tx}}{L}_{{rx}}})({r}_{{rx}}+{R}_{{rec}})}$$

where $\omega=2\pi \cdot 6.78{MHz}$ and ${R}_{{rec}}$ is the resistance of the receiving's impedance.

### Specific absorption rate of electromagnetic power

We employed HFSS to simulate the specific absorption rate (SAR) of the WIPES after implantation to assess its safety. SAR quantifies the rate of electromagnetic energy absorption by biological tissues and reflects potential thermal effects. A simplified human body model including skin, fat, muscle, and internal organs was built, with conductivity and permittivity set based on standard tissue parameters. The WIPES was implanted near the urethral sphincter, in the lower abdomen and coupled with an external electromagnetic field via a transmitting coil. The operating frequency was set at 6.78 MHz to match the actual working conditions. HFSS calculated the electromagnetic field distribution and energy absorption. Open boundary conditions were used to avoid reflection artifacts. SAR was calculated using the following formula:

5 $${SAR}=\frac{\sigma }{\rho }\cdot {{{{\rm{| }}}}E{{{\rm{| }}}}}^{2}$$

where $\sigma$ is the tissue conductivity, $| E|$ is the electric field strength, and$\,\rho$ is the tissue density. SAR is expressed in watts per kilogram (W/kg). According to IEEE C95.1 and ICNIRP, SAR should not exceed 10 W/kg for the head and limbs, and 20 W/kg for the torso. The simulated peak SAR at the implantation site was 3.951 W/kg, well below the 20 W/kg threshold, indicating compliance with safety standards. (Fig. S18)^60^.

### Post-implantation X-ray and CT imaging

After wound healing, radiography and computed tomography (CT) were performed to verify the structural integrity and in vivo positioning of the implanted WIPES device before subsequent functional experiments. Animals were imaged under inhalation anesthesia, and CT volumes were reconstructed to obtain 3D renderings of the implant. Imaging was used for qualitative confirmation of implant placement, consistent with Figs. 4b–c and S7, S25.

### Animal euthanasia protocol

Post-experiment, the animals were humanely euthanized following standard protocols to minimize suffering. Euthanasia was performed using an overdose of isoflurane, a commonly used anesthetic agent, administered in a closed chamber. The rats were first placed in the chamber, and isoflurane was delivered at a concentration sufficient to induce deep anesthesia rapidly. The animals were monitored closely to ensure the complete loss of consciousness, confirmed by the absence of reflexes and a lack of response to stimuli. Once deep anesthesia was confirmed, euthanasia was completed by cervical dislocation to ensure a swift and painless death. This method adheres to the ethical guidelines for the humane treatment of laboratory animals, ensuring that the process is both quick and effective. Following euthanasia, death was confirmed by the cessation of breathing and the absence of a heartbeat, verified through palpation and visual inspection. The entire procedure was conducted under the supervision of a trained technician, ensuring compliance with institutional and national guidelines for animal care and use. The remains were then handled according to biohazard disposal protocols to ensure safe and respectful disposal.

### Rat models of stress urinary incontinence

To establish stress urinary incontinence (SUI) in female and male rats, two complementary approaches were employed. In females, a vaginal distension (VD) procedure was performed to mimic birth-related pelvic floor injury (Fig. S23). Under isoflurane anesthesia delivered via a precision vaporizer (isoflurane vaporizer, nose cone), the animal was placed supine with hindlimbs abducted and the vaginal introitus gently dilated using lubricated cotton swabs. A lubricated 6 Fr balloon catheter (rodent Foley catheter, 6 Fr) was inserted fully into the vaginal canal, then inflated with 2.5–3.5 mL of warm saline. During balloon inflation, the rat frequently exhibited reflexive urine leakage due to compressive pressure on the urethra. To apply axial stretch, the catheter hub was connected to a 20–30 g weight (elastic traction line with weight), and distension was maintained for 2–4 h with brief deflations every 30 min to prevent ischemia. After removal, the introitus was inspected and covered with topical antibiotic ointment (cefazolin, 25 mg/kg, s.c.).

In males, a post-prostatectomy-like model was developed by surgically removing the prostate and then applying targeted electrocautery to the external urethral sphincter (EUS) to simulate sphincteric injury following radical prostatectomy (Fig. S24). After midline laparotomy under isoflurane anesthesia, the bladder neck, prostate, and membranous urethra were exposed using fine scissors and forceps (9 microsurgical scissors, Adson forceps, retractors). The bilateral prostate lobes were carefully dissected free and excised (micro-scissors, bipolar cautery for hemostasis). Following prostate removal, the puboprostatic ligaments and adjacent peri-urethral adhesions were divided to increase urethral mobility. A micro-bipolar electrosurgical unit (bipolar generator with micro-forceps, 3–5 W) was then applied at the 3 o'clock and 9 o'clock positions of the membranous urethra for 1 s each, producing focal blanching without penetrating the urethral lumen. Irrigation was performed with warm saline, and hemostasis was assisted by gelatin sponge. The abdominal wall was closed in two layers (5-0 absorbable sutures). All animals received prophylactic antibiotics (cefazolin, 25 mg/kg, s.c.) and analgesia (meloxicam 1–2 mg/kg, s.c.) post-operatively.

### Rat models of urgency urinary incontinence

Urgency urinary incontinence (UUI) was pharmacologically induced in both female and male Sprague–Dawley rats through intraperitoneal injection of cyclophosphamide (CTX, 80 mg/kg), a well-established rodent model of cystitis and detrusor overactivity^61–64^ Systemically administered CTX is metabolized into the urotoxic compound acrolein, which damages the urothelium and lamina propria, triggers inflammatory cascades, and sensitizes bladder afferents^65,66^ These processes collectively lead to bladder overactivity, characterized by increased micturition frequency, reduced capacity, and secondary urethral sphincter dysfunction, thereby reproducing the urge component of urinary incontinence^67,68^ CTX was prepared fresh on the day of use by dissolving powder in sterile 0.9% NaCl to 20 mg/mL, vortexing until clear, and passing through a 0.22 µm syringe filter to ensure sterility (powder CTX, sterile saline, 0.22 µm filter). After brief restraint without anesthesia, the dose was delivered i.p. in the lower right abdominal quadrant using a 27–29 G needle at ~4 mL/kg injection volume. Animals were returned to warmed cages with ad libitum water and wetted chow and were clinically monitored for hematuria, reduced activity, and dysuria. Subcutaneous isotonic saline (5–10 mL/kg, s.c.) was provided as needed to support hydration, while analgesics known to alter bladder reflexes were avoided or minimized per IACUC guidance. Functional assessments for UUI were performed in the acute window 24–48 h post-CTX, encompassing awake cystometry (intercontraction interval, non-voiding contractions), voiding Spot Assay/metabolic cage readouts (voiding frequency and per-void volume), with the same timing applied to both sexes. Consistent with these functional abnormalities, H&E staining revealed CTX-induced urothelial thickening and inflammatory remodeling of the bladder wall, further validating successful model establishment (Fig. S30).

### Spinal nerve stimulation for UUI Ttreatment

To simulate the conventional sacral nerve stimulation (SNS) treatment used clinically for urge urinary incontinence (UUI), we performed transcutaneous needle-based electrical stimulation targeting the rat sacral nerve plexus. Two sterile acupuncture needles (diameter 0.3 mm, length 25 mm) were inserted percutaneously at the bilateral posterior superior iliac spine region (approximating the S1–S2 level in rats), under brief isoflurane anesthesia (Fig. S25a). The insertion site and angle were guided by anatomical landmarks and confirmed via lateral X-ray imaging (Fig. S25b), showing needle tips adjacent to the sacral nerve foramina. The external stimulation was delivered using a programmable pulse generator (DF-301, Suzhou Dongfang Electronics, China), connected to the needles via alligator clips. Electrical stimulation was applied with monophasic square pulses (frequency: 60 Hz, pulse width: 0.3 ms, amplitude: 4 V) continuously for 30 min per day, over a period of 7 consecutive days. The parameters were selected based on prior literature to ensure effective nerve activation while avoiding tissue damage. Throughout the stimulation, the animals remained lightly anesthetized to minimize stress and motion artifacts. This setup was used as a control treatment group to evaluate the efficacy of the WIPES device in modulating urinary continence–related functions, enabling direct comparison between conventional sacral nerve modulation and targeted peripheral muscle stimulation.

### Urodynamic evaluation

To further analyze the therapeutic effects of stimulation on stress urinary incontinence (SUI), external urethral sphincter electromyography (EUS-EMG) and leak point pressure (LPP) tests were performed under inhalational anesthesia with isoflurane (isoflurane vaporizer). For EUS-EMG, through a perineal approach, bipolar fine-wire hook electrodes (50–100 µm, 1–2 mm inter-electrode distance) were inserted 0.5–1 mm into the EUS. Signals were amplified (gain 1000–5000×), band-pass filtered at 300–3000 Hz, and digitized at ≥10 kHz. Raw traces were full-wave rectified and a 50–100 ms sliding RMS envelope was computed. Outcomes included basal activity, guarding-burst and voiding-burst RMS amplitudes, and their ratios to baseline. For LPP testing, an inflatable vaginal balloon catheter was positioned adjacent to the bladder neck and urethra. After the bladder was filled to a standardized volume, external pressure was gradually applied by inflating the vaginal balloon. Urine leakage from the urethral meatus was visually observed, and the bladder pressure at the moment of the first leakage was recorded. Pressure was referenced against the water column height of a connected three-way stopcock system and expressed in cmH₂O. Each measurement was repeated three times, and the average value was used for analysis.

To evaluate detrusor overactivity in urge urinary incontinence (UUI), continuous cystometry (CMG) was performed. A small puncture was made at the bladder dome, and a PE-50 catheter was carefully inserted into the bladder lumen. The external end of the catheter was connected via a three-way stopcock to an infusion pump and a pressure transducer (MLT844 Physiological Pressure Transducer, ADInstruments). The pressure signal was digitized and recorded using a PowerLab data acquisition system with LabChart software. Warm sterile saline (37 °C) was infused at a constant rate of 0.08–0.10 mL/min, and intravesical pressure was continuously monitored to evaluate bladder filling, voiding contractions, intercontraction interval, and baseline pressure.

Because cystometry and EUS-EMG recordings were performed under isoflurane anesthesia, these data were interpreted as functional outcome measures rather than as indicators of physiological bladder–sphincter coordination. Consistently, LPP measurements were used as a functional endpoint to assess urethral outlet competence under identical anesthetic conditions across experimental groups, enabling valid relative comparisons between treatments.

### Surgical procedure and device implantation

Sprague-Dawley (SD) rats were anesthetized with isoflurane gas. Following skin disinfection and preparation, a 3 cm midline incision was made in the abdomen to expose the subcutaneous and muscular connective tissues, providing adequate space for the implantation of the WIPES implant. A 1 cm incision was then made along the linea alba of the rectus abdominis muscle, allowing access to the bladder within the abdominal cavity. The connective tissue surrounding the urethra was carefully dissected, and adjacent organs were bluntly separated to fully expose the urethral tissue. The positive and negative electrodes of the conductive component of the WIPES implant were subsequently wrapped around the urethral sphincter with appropriate tension, after which the bladder and urethra were repositioned. The abdominal wall muscles were sutured using 5-0 absorbable sutures, and the WIPES implant was securely placed between the subcutaneous layer and the abdominal wall. The epidermis was then closed using 3-0 non-absorbable sutures.

### Postoperative care and anti-inflammatory treatment

After the wound was closed, penicillin ointment was applied to prevent infection, and the area was covered with sterile gauze. The gauze was changed daily, and the wound was checked for signs of bleeding, infection, or other issues. Ibuprofen was given for the first three days to manage pain and reduce inflammation. It was mixed into the rats' drinking water to make it easy for them to consume.

The rats were kept in individual cages to avoid disturbing their wounds, with the temperature set at 22–24 °C to keep them comfortable. Soft bedding was used in the cages to prevent irritation, and nesting materials were provided to help reduce stress. The surgical site was cleaned daily with sterile saline, followed by reapplying the penicillin ointment. The rats' overall health was monitored closely, including their weight and how much they were eating and drinking. If extra pain relief was needed, buprenorphine was given as an injection under the skin. The rats were observed regularly, especially during the first 72 h after surgery, to make sure they were recovering well.

### Urine collection protocol

Special sodium-based mineral sand designed for rodents is widely used to absorb any moisture produced during the urination and defecation processes of rats, clumping together to keep the environment dry and odor-free. A 5 cm thick layer of sterile sodium-based mineral sand is spread at the bottom of a standard-sized sterile housing cage for SD rats, with no other absorbent material added. The cage is equipped with sufficient food and water. Once the surgical wounds of all rats have fully healed, they are transferred to individual cages to initiate the urine collection experiment. At 12:00 AM, 8:00 AM, and 4:00 PM each day, all clumps in the cage are separated using a slotted scoop. Clumps containing feces and those located directly under the water dispenser (to prevent counting moisture from spilled drinking water) are manually removed. The clumps are then placed in a container, with the number recorded, and the total weight measured using an electronic scale to calculate the rats' urination frequency and average urine volume (the linear relationship between the weight of the urine sand clumps and the volume of urine has been proven, as shown in Fig. S14).

### Toxicity analysis

The Simian Virus 40-transformed Human Uroepithelial Cells line (SV-HUC-1) was obtained from the American Type Culture Collection (ATCC, USA) and maintained in an incubator with 5% CO_2_ at 37 °C. The cells were cultured in high-glucose Dulbecco's Modified Eagle Medium (high-glucose DMEM, Gibco, USA) as the basic culture medium, supplemented with 10% fetal bovine serum (FBS, Gibco, USA), 100 U/ml streptomycin, and 100 µg/ml penicillin (Gibco, USA). After the third passage, the cells reached stable growth. The sterilized WIPES device was introduced into the culture dishes of the experimental group. The cell density and growth status in the culture dishes were observed and photographed at 24 and 72 h, respectively, to confirm that the device itself was non-toxic and harmless to the tissue. Furthermore, systemic safety was evaluated by H&E staining of major organs (Fig. S29) showed no pathological alterations, indicating that WIPES implantation and stimulation caused no detectable organ toxicity.

### Biological tissue sample preparation

Following the completion of the experimental procedures, the rats were humanely euthanized using an overdose of isoflurane, followed by cervical dislocation to ensure a rapid and painless death. After confirming the cessation of vital signs, the abdominal cavity was carefully opened through a midline incision. The bladder and urethral tissues were identified and meticulously excised using sterile surgical instruments.

The excised tissues were immediately rinsed in cold phosphate-buffered saline (PBS) to remove any blood or debris. The tissues were then placed in 10% neutral buffered formalin for 24 h to achieve fixation, ensuring the preservation of cellular and tissue structures. Following fixation, the tissues underwent a graded series of ethanol solutions (70%, 80%, 90% and 100%) for dehydration, with each step lasting 30 min. This dehydration process was critical for preparing the tissues for subsequent embedding in paraffin. The fixed and dehydrated tissues were then stored in 100% ethanol until they were ready for embedding and further histological analysis. All samples were labeled appropriately and stored at 4 °C to maintain their integrity until processing.

### Urethral sphincter muscle tissue staining

Paraffin-embedded bladder and urethral sphincter tissues were sectioned at a thickness of 4 µm using a microtome, mounted on glass slides, and dried overnight at room temperature. Before staining, sections were deparaffinized in xylene (3 × 5 min), rehydrated through a graded ethanol series (100%, 95%, 80%, 70%), and rinsed in distilled water.

Hematoxylin and Eosin (H&E) Staining. For general histological examination, tissue sections were stained with hematoxylin for 5 min, rinsed in tap water, and differentiated in 1% acid alcohol. Bluing was performed using lithium carbonate or Scott's tap water substitute for 2 min. Slides were then stained with eosin for 2 min to visualize cytoplasm and extracellular matrix components. After removing excess dye in 95% ethanol, sections were dehydrated, cleared in xylene, and coverslipped. The stained tissues were observed under a light microscope to evaluate overall morphology and pathological alterations.

Masson's Trichrome Staining. To assess fibrosis and collagen deposition, sections were stained with Weigert's iron hematoxylin for 10 min to visualize nuclei, followed by Biebrich scarlet–acid fuchsin for 10–15 min to stain cytoplasm and muscle fibers. After differentiation in phosphomolybdic–phosphotungstic acid until collagen fibers were clearly distinguished, slides were counterstained with aniline blue for 5–10 min and briefly rinsed in distilled water. Sections were then treated with 1% acetic acid for 2–5 min, dehydrated, cleared, and coverslipped. Collagen deposition and fibrotic changes were examined microscopically.

TUNEL Staining. Apoptosis in bladder and urethral tissues was evaluated using a commercial TUNEL (Terminal deoxynucleotidyl transferase dUTP Nick-End Labeling) kit following the manufacturer's protocol. After deparaffinization and rehydration as described above, tissue sections were treated with proteinase K (20 µg/mL, 15 min, room temperature) for permeabilization, washed with PBS, and incubated with the TUNEL reaction mixture (TdT and fluorescein-labeled dUTP) at 37 °C for 60 min in a humidified chamber. After washing, nuclei were counterstained with DAPI (5 min), and slides were mounted with anti-fade medium. Apoptotic cells displaying green nuclear fluorescence were visualized and quantified using a fluorescence microscope.

### Quantification of c-Fos for urge-suppression assessment

To assess neuronal activation associated with bladder filling and urge control, c-Fos expression was evaluated within the dorsolateral (dlPAG) and ventrolateral (vlPAG) regions of the periaqueductal gray (PAG). Following standardized cystometric stimulation to evoke the desired neural response, animals were maintained at the threshold filling state for 10–15 min and euthanized 90 min later to capture the c-Fos peak expression window (60–120 min). Brains were perfusion-fixed sequentially with PBS and 4% paraformaldehyde, post-fixed overnight, cryoprotected in 30% sucrose, and coronally sectioned at 30 µm thickness at 120 µm intervals covering the PAG along the rostrocaudal axis. Free-floating sections were processed for c-Fos immunofluorescence using rabbit anti-c-Fos (1:500) and species-appropriate Alexa-conjugated secondary antibody (1:1000), followed by DAPI counterstaining.

Confocal imaging was performed under identical acquisition parameters (10×/20× objective, 0.5 µm/pixel, fixed laser power and gain, 16-bit TIFF export). Sections were aligned to the Paxinos & Watson atlas (bregma −7.3 to −8.3 mm) to confirm PAG levels. Within each PAG section, bilateral sampling frames were defined relative to the aqueduct contour, and dlPAG and vlPAG regions of interests (ROIs) were delineated according to atlas-based anatomical landmarks, while avoiding vessels and artifacts. Image processing in FIJI/ImageJ or CellProfiler used standardized parameters: background subtraction (40–60 px), optional CLAHE (clip-limit 0.01), Otsu thresholding, watershed separation, and particle analysis (50–400 µm², circularity 0.3–1.0). c-Fos–positive cell density (cells/mm²) was calculated as nucleus count per sampled area, averaged across 2–3 non-overlapping sections per animal. Thresholds were fixed across batches, and segmentation was verified by blinded assessors. Statistical comparisons used one-way ANOVA with Tukey's post hoc test or Kruskal–Wallis with Dunn–Bonferroni correction.

### Neural immunostaining and quantification

To assess peri-urethral and pudendal neural changes with pathway-agnostic structural readouts, paraffin-embedded 4-µm sections of urethra (including the peri-urethral plexus surrounding the external urethral sphincter, EUS) and proximal pudendal nerve were mounted, deparaffinized in xylene (3 × 5 min), and rehydrated through graded ethanol. Antigen retrieval was performed in citrate buffer (pH 6.0, microwave 10 min), followed by cooling and PBS rinse. Sections were permeabilized with 0.1% Triton X-100 (10 min) and blocked with 5% BSA in PBS (1 h, RT). Primary antibodies (overnight, 4 °C; 1:100–1:500) included PGP9.5 (pan-neuronal fibers) and GAP-43 (axonal growth/sprouting), after PBS washes, sections were incubated with species-appropriate fluorophore-conjugated secondaries (1 h, RT, dark) and counterstained with DAPI. Slides were mounted in anti-fade medium and imaged on the same epifluorescence/confocal platform under identical exposure and gain settings within each batch; pixel size was recorded for conversion to physical units.

For quantification, anatomically defined ROIs were delineated for each tissue type prior to analysis. For urethral sections, the ROI encompassed the peri-urethral neural plexus circumferentially surrounding the EUS, identified based on smooth muscle morphology and lumen position. ROIs were manually traced under consistent magnification using DAPI and bright-field references, following the same anatomical criteria across all groups. Images were analyzed in FIJI/ImageJ following a fixed, blinded pipeline: background subtraction (rolling-ball 40–60px), global thresholding of each marker (Otsu), binary mask cleanup, and skeletonization. For PGP9.5, we quantified the percent positive area (%Area) and fiber length density (skeleton length per unit area, converted to mm/mm²), representing total neuronal fiber coverage and trajectory continuity. For GAP-43, to avoid intensity-related bias, we quantified a normalized GAP-43 regeneration index defined as the GAP-43–positive signal length within the peri-urethral region divided by the total analyzed tissue length, providing a robust measure of axonal sprouting independent of staining intensity or fiber overlap. All threshold and filter parameters were fixed across groups, and assessors were blinded to experimental allocation. Multiple sampled fields per animal were averaged to yield one biological replicate for statistical comparison.

### Immunofluorescence staining

To investigate the co-expression of target proteins in bladder and urethral tissues, dual-labeling three-color immunofluorescence staining was performed. Primary antibodies (Abcam, 1:50–1:500) included: nNOS (ab76067), VIP (ab272726), α-SMA (ab7817), ROCK2 (ab125025). Paraffin-embedded sections were deparaffinized in xylene and rehydrated through a graded ethanol. Antigen retrieval was then performed by heating in a citrate buffer (pH 6.0) via microwave (10 min). After cooling, sections were washed with PBS and blocked with 5% bovine serum albumin (BSA) in PBS (1-hour) to prevent non-specific binding. Sections were incubated overnight at 4 °C with two primary antibodies diluted appropriately in 1% BSA/PBS. Two antibody combinations were used: α-SMA + ROCK2, nNOS + VIP. After incubation, slides were washed with PBS and incubated for 1 h at room temperature (in the dark) with fluorescently labeled secondary antibodies, each conjugated to distinct fluorophores for three-color detection. After PBS washing, nuclei were counterstained with DAPI. Slides were mounted using anti-fade medium and examined under a fluorescence microscope. Images were captured, and co-localization was analyzed based on the distinct fluorescence signals.

### Data analysis

The sample data obtained by the urine sand collection method is analyzed to determine the extent to which electrostimulation therapy can improve urinary incontinence symptoms. If the average urination volume in the UI & ES group exceeds the average value in the Incontinence modelling group for either males or females, it is considered a sample of symptom improvement. Similarly, if the urinary frequency (number of urinary sand masses) of a sample in the UI & ES group exceeds the average value in the Incontinence modelling group for either males or females, it is considered a sample of symptom improvement.

The symptom improvement rate for each metric is then calculated as follows:

5 $${R}_{{volume}}=\frac{{N}_{{volume}\,}}{{N}_{{total}}}$$

6 $${R}_{{{{\rm{frequency}}}}}=\frac{{N}_{{{{\rm{frequency}}}}}}{{N}_{{total}}}$$

where ${R}_{{volume}}$ and ${R}_{{{{\rm{frequency}}}}}$ are the improvement rates for average urination volume and urinary frequency, respectively, ${N}_{{volume}}$ and ${N}_{{{{\rm{frequency}}}}\,}$ are the number of samples showing improvement in each metric, and ${N}_{{total}}$ is the total number of samples in each metric.

To reduce statistical error from using single-sample data for each metric, the adjusted symptom improvement rate is calculated by averaging the improvement rates from both metrics as:

7 $${R}_{{{{\rm{adjusted}}}}}=\frac{{R}_{{volume}}+{R}_{{{{\rm{frequency}}}}}}{2}$$

which represents the adjusted symptom improvement rate. Additionally, all statistical analyses and data processing in this assay were performed in MATLAB for accuracy and visualized in OriginLab for high-quality presentation. Group comparisons were performed using the Wilcoxon rank sum test or t-test for continuous variables, with statistical significance set at *P* < 0.05. Asterisks (*) denote statistical significance as * (*P* < 0.05), ** (*P* < 0.01) and *** (*P* < 0.001), while “ns” indicates no significant difference between the groups.

### Reporting summary

Further information on research design is available in the Nature Portfolio Reporting Summary linked to this article.

### Ethics

This study did not involve human participants, human data, or clinical samples. All animal experiments were conducted in accordance with the Guide for the Care and Use of Laboratory Animals (NIH Publication No. 85-23, revised 1996) and were approved by the Animal Ethics Committee of Ruijin Hospital, School of Medicine, Shanghai Jiao Tong University, Shanghai, China (Ethics No. RJ2024051). Rats were handled humanely, with appropriate anesthesia and surgical procedures to minimize pain and distress.

## Supplementary information

Supplementary Information

Reporting Summary

Transparent Peer Review file

## Source data

Source Data

## Data Availability

All data supporting the findings of this study are available within the article and its supplementary files. Any additional requests for information can be directed to and will be fulfilled by the corresponding authors. Source data are provided with this paper.

## Code availability

Data analysis made use of inbuilt functions in MATLAB and OriginLab. All parameters used for analysis are available in Methods and Supplementary Information. All codes for the wireless-powered implanted programmable electrostimulation of sphincters used in this study are available from the corresponding authors on reasonable request.
