# Supplementary material for: Wireless electrostimulation implants enable sphincter neuromuscular improvement toward mixed urinary incontinence
Source: Nat Commun. 2026 Apr 15;17:5226. doi: 10.1038/s41467-026-71532-7 (PMC13260462; doi:10.1038/s41467-026-71532-7)
Supplement: Supplementary file 1 — Supplementary Information [file 41467_2026_71532_MOESM1_ESM.pdf]

Supplementary Information for

Wireless electrostimulation implants enable sphincter neuromuscular improvement toward mixed urinary incontinence

Tianxiang Zheng,[1,†] Li Tao,[2,†] Qiuhua Gao,[3] Zhiran Yi,[3] Yaoxia Shao,[4] Yu Xiao,[1] Ning Kang,[5] Christopher H. T. Lee,[5,6] Ming Liu,[7] Chengbin Ma,[1,*] Wenming Zhang,[3,*] Yuan Shao,[2,*] Lei Shao,[1,*] and Metin Sitti [8,9*]

[1] University of Michigan-Shanghai Jiao Tong University Joint Institute, Shanghai Jiao Tong University, Shanghai, China 200240

[2] Ruijin Hospital, School of Medicine, Shanghai Jiao Tong University, Shanghai, China 200025

[3] School of Mechanical Engineering, Shanghai Jiao Tong University, Shanghai, China 200240

[4] School of Information and Electrical Engineering, Hangzhou City University, Hangzhou, China 310015

[5] School of Electrical and Electronic Engineering, Nanyang Technological University, Singapore 639798

[6] Lee Kong Chian School of Medicine, Nanyang Technological University, Singapore 308232

[7] School of Electrical Engineering, Shanghai Jiao Tong University, Shanghai, China 200240

[8] School of Medicine and College of Engineering, Koç University, Istanbul, Turkey 34450

[9] Physical Intelligence Department, Max Planck Institute for Intelligent Systems, Stuttgart, Germany 70569

[†] Equal contribution

* Correspondence to: chbma@sjtu.edu.cn, wenmingz@sjtu.edu.cn, shaoyuan15@hotmail.com, lei.shao@sjtu.edu.cn, sitti@is.mpg.de

Table S1. Quantitative comparison of WIPES vs. commercial sacral neuromodulation systems

System	WIPES	Medtronic InterStim™ II	Medtronic InterStim™ Micro	Axonics R20
**Implant type**	Thin, flexible patch directly on urethral sphincter	Primary-cell IPG + S3 tined lead (Indirect SNS)	Rechargeable IPG in gluteal pocket + S3 tined lead (indirect SNS)	Rechargeable IPG + S3 tined lead (indirect SNS)
**Volume**	0.3 cm^3	14 cm^3	2.8 cm^3	5 cm^3
**Power source**	Wireless power	Primary-cell lithium battery	Rechargeable battery (transcutaneous energy transfer)	Rechargeable battery (transcutaneous energy transfer)
**Recharge interval**	N/A	N/A	~20 min weekly	6–10 months
**Targeting**	Direct anatomical placement on sphincter	S3	S3	S3
**Effective rate of treatment**	UUI:90.62%<br>SUI: 97.92%	UUI~76%<br>(⩾50% reduction)	UUI:79-89%<br>(⩾50% reduction)	UUI:70-90%<br>(⩾50% reduction)
**Device price**	$120	>$10,000	>$10,000	>$10,000
**Portability notes**	Fully implanted and external flexible belt Tx supports daily/home use	Fully implanted	Fully implanted	Fully implanted

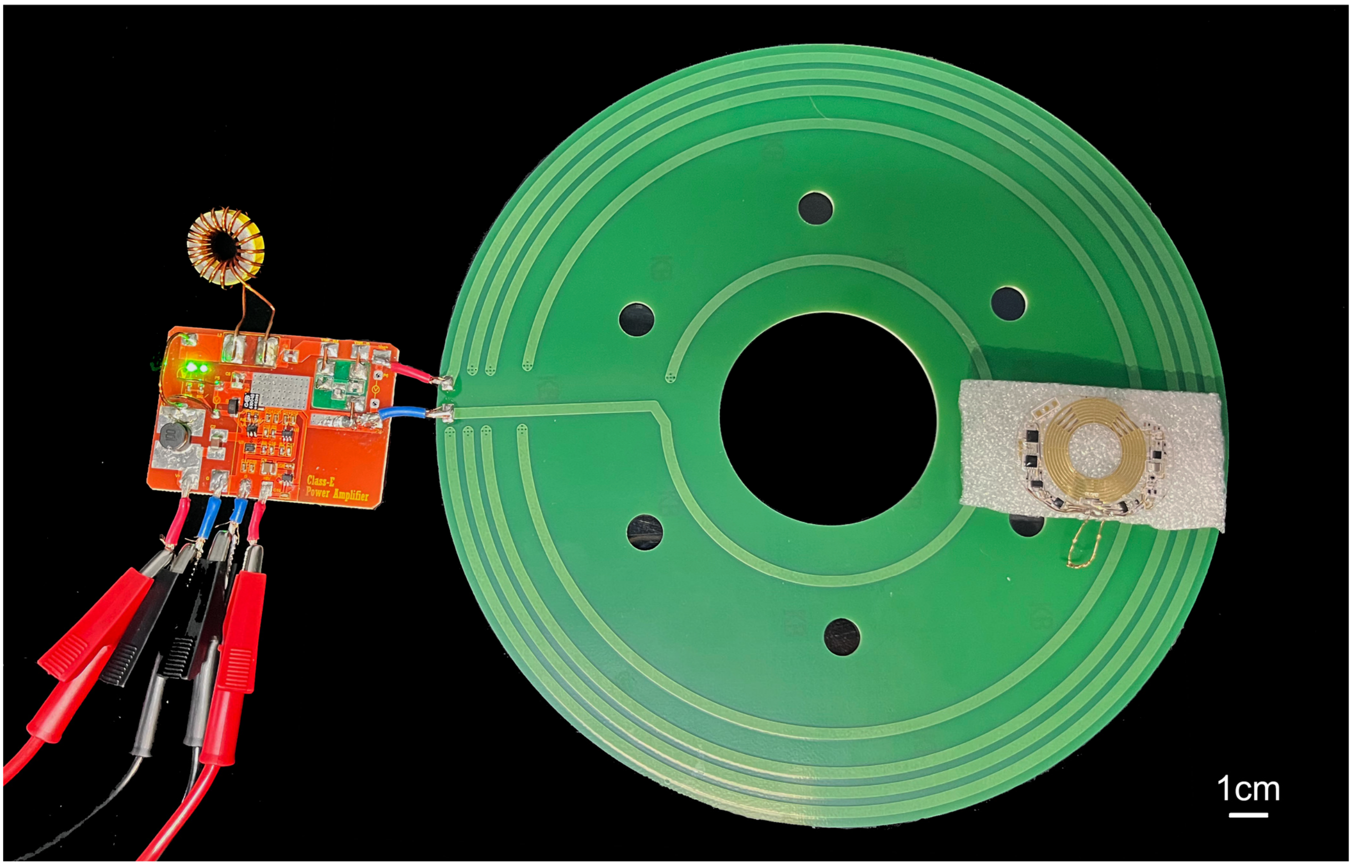

Fig. S1. The WIPES operating system consists of a uniform transmitting coil (Tx coil) (green) and a Class E power amplifier (red). The WIPES implant consists of a receiving coil (Rx) (gold coil wrapped in a PDMS transparent gel) and an electrostimulation circuit. The Tx coil's size is a disk with a diameter of 20 cm. The WIPES implant is a hollow elliptical ring with a long axis of 44 mm, a short axis of 32 mm, a center circular void of 14 mm, and a thickness of 2 mm. The void center of the implanted device not only contributes to the overall light weight but also allows it to withstand bending and twisting stress, reducing the risk of mechanical failure after implantation.

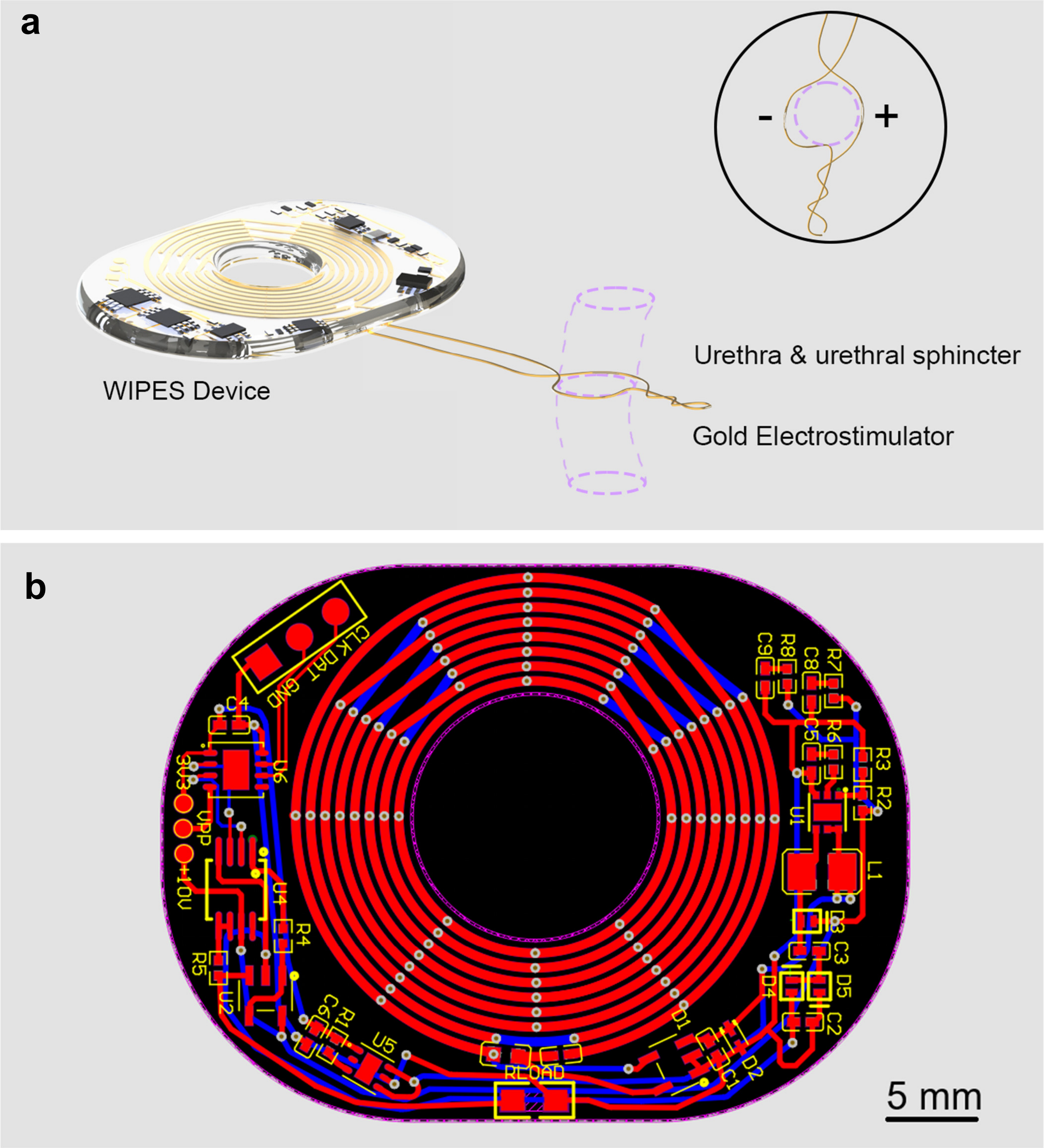

Fig. S2. Special implantable structures were designed in order to be able to implant into rats. **(a)** The special wire electrode winding method can effectively wrap around the urethral sphincter to achieve electrostimulation without creating initial pressure on urethra. **(b)** WIPES implant's circuit design (by Altium Designer software).

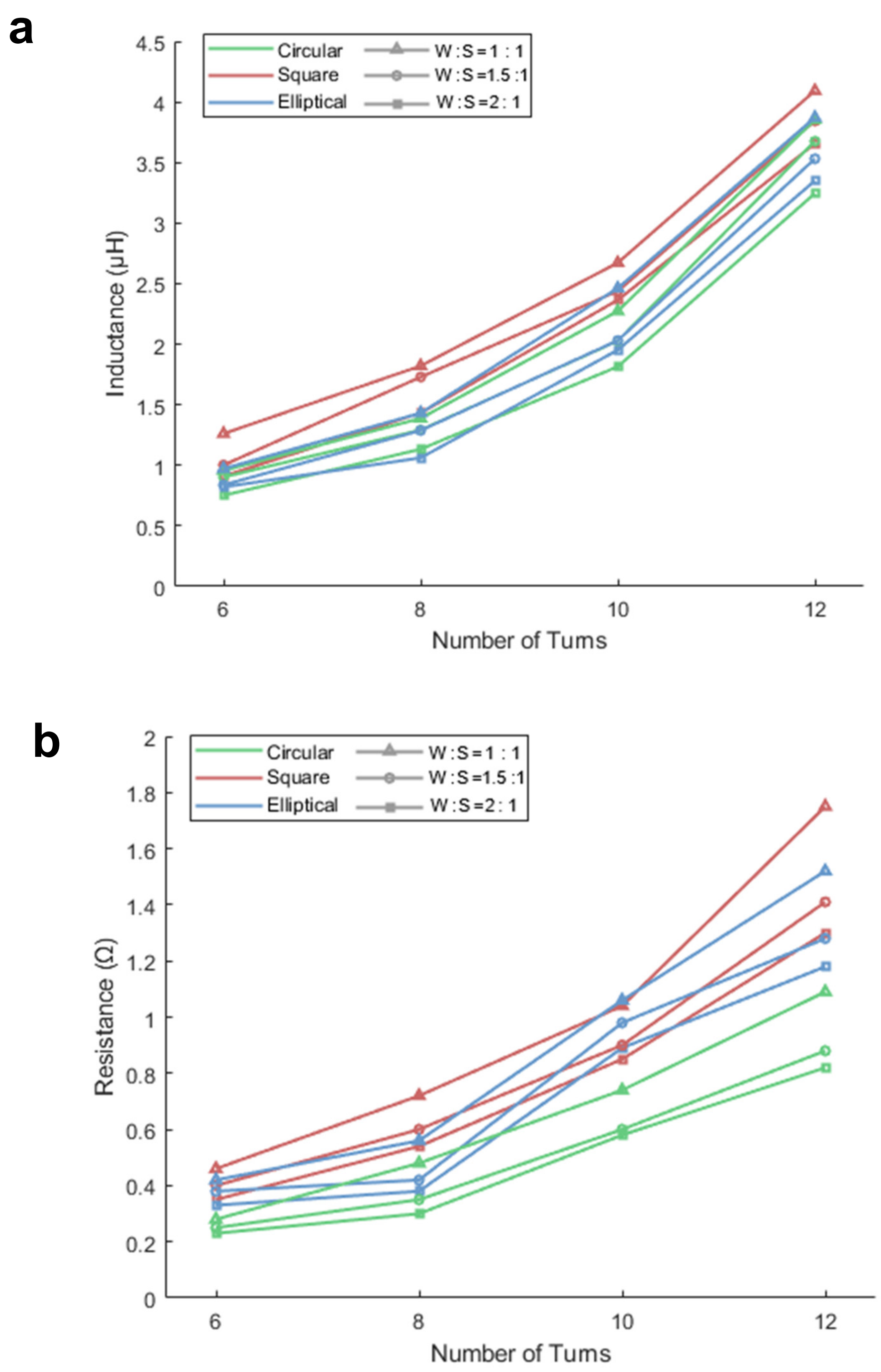

Fig. S3. To optimize the Rx coil design for WIPES and ensure efficient power transmission, simulations were conducted comparing different shapes, turns, and wire width-to-spacing ratio. The effective wiring area was constrained to 0.85 πcm^2 and the effective flux area to 0.5 πcm^2, based on the device's layout and biological compatibility requirements. Three Rx coil shapes were considered: Circular (outer diameter 2.3 cm, inner diameter 1.4 cm), Square (outer side 2.1 cm, inner side 1.25 cm), and Elliptical (outer semi-circle diameter 2 cm, inner diameter 1 cm, and connecting length 0.8 cm). The next step was optimizing the number of turns and the wire width-to-spacing ratio through HFSS simulations. The coil turns were varied from 6 to 12, and the wire width-to-spacing ratios were set to 2:1, 1.5:1, and 1:1 for all three shapes. The resulting self-inductance (L_{rx}) (Fig. S3a) and internal resistance (r_{tx}) (Fig. S3b) were simulated with different coil parameters. All data points are deterministic outputs from HFSS simulations; therefore, no sample size (n) or error bars apply.

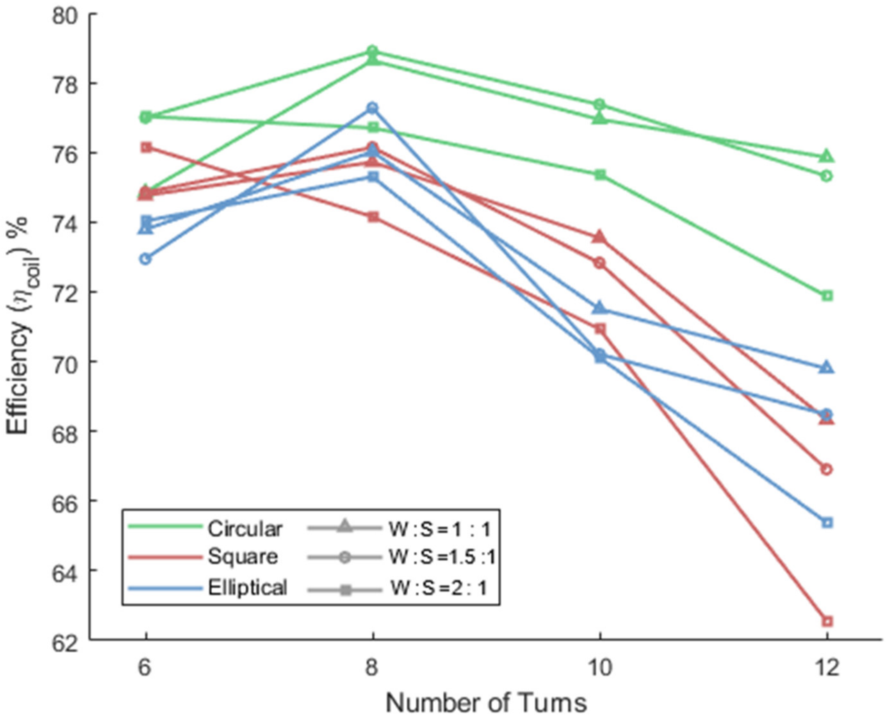

Fig. S4. When L_{tx} r_{tx}, k, and R_{rec} remain constant, as $L_{tx} = 7.85\ uH$ $r_{tx} = 1.82\ \Omega$, $k = 0.055$ (1 cm gap from the Tx coil plane simulated by HFSS), and $R_{rec} = 3.5\ \Omega$, the η_{coil} is directly related to both L_{rx} and r_{rx}. To further analyze this relationship, efficiency was computed for different shapes, number of turns and wire width-to-spacing ratio (W:S). It is proved that the optimized design is 8-turn circular coil with a W:S = 1.5:1. All data points are deterministic outputs from HFSS simulations; therefore, no sample size (n) or error bars apply.

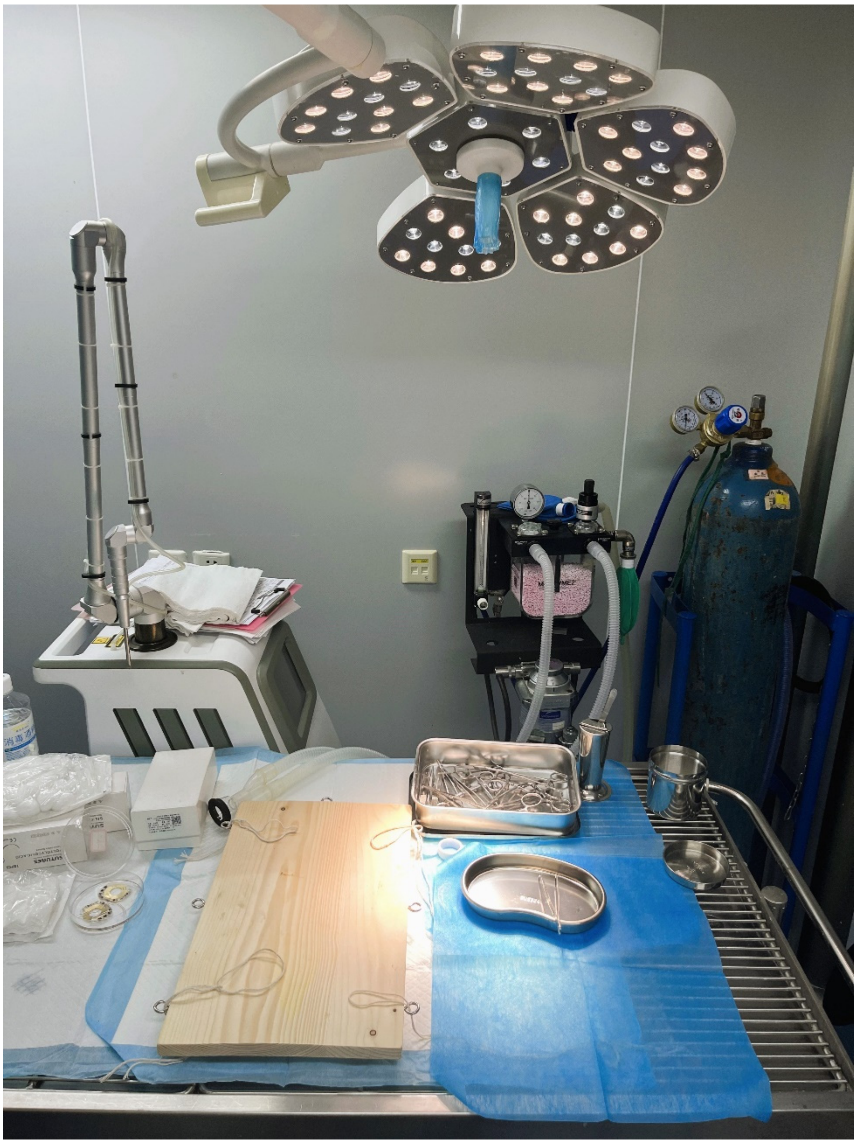

Fig. S5. Overall layout of the surgical environment, including the operating table, medical equipment, and anesthesia instrument.

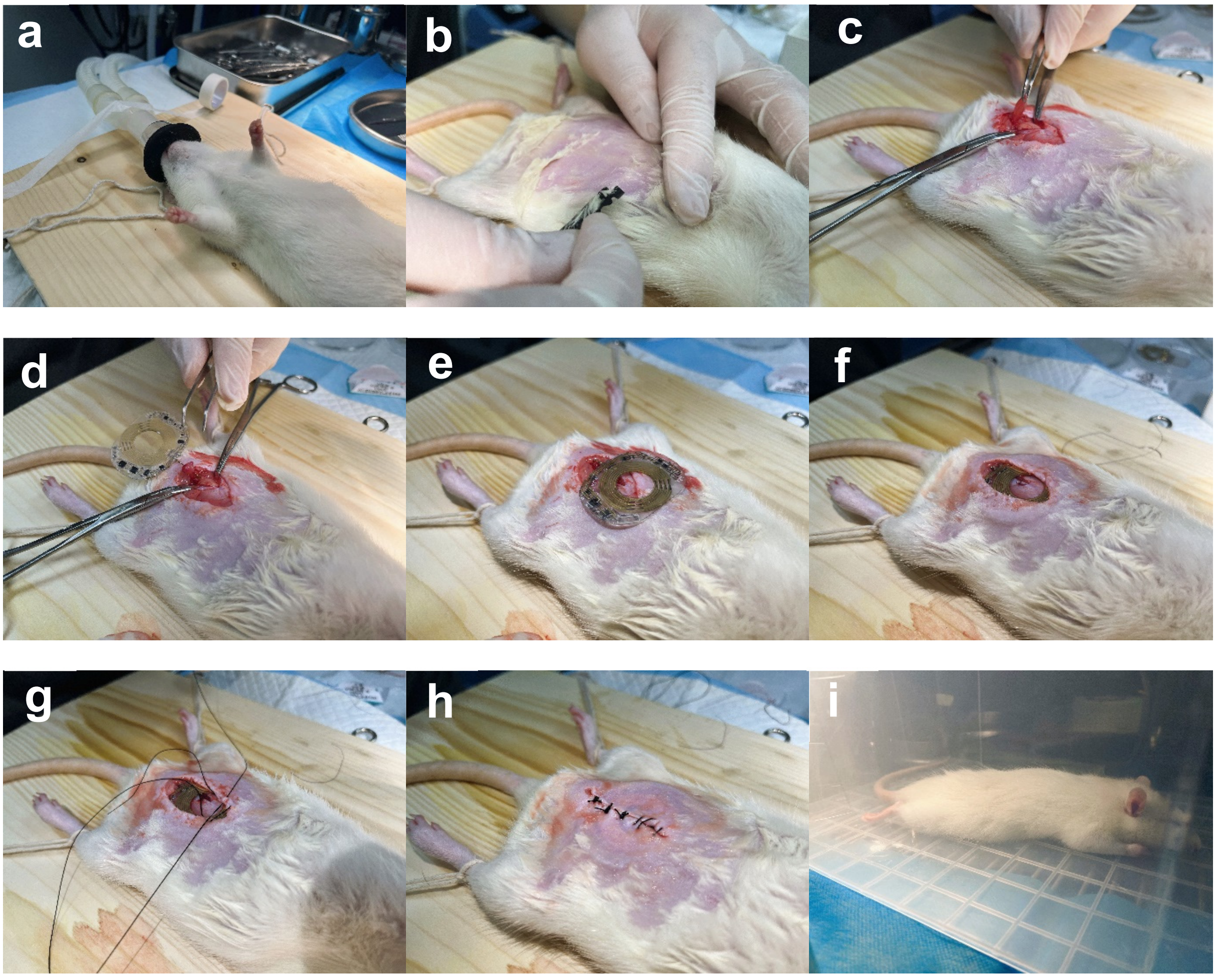

Fig. S6. Details the implantation process of the WIPES implant in rats. **(a)** Anesthesia of the rat using a gas anesthesia machine, **(b)** Shaving of the rat's abdomen, **(c)** Opening the abdomen and incising the muscle layers to locate the urethral sphincter, **(d)** Wrapping the WIPES electrodes around the urethral sphincter, e. Suturing the muscle layers, **(f)** Implanting WIPES, **(g)** Suturing the outer skin layer, h. Postoperative cleaning and disinfection, and **(i)** Waiting for postoperative recovery.

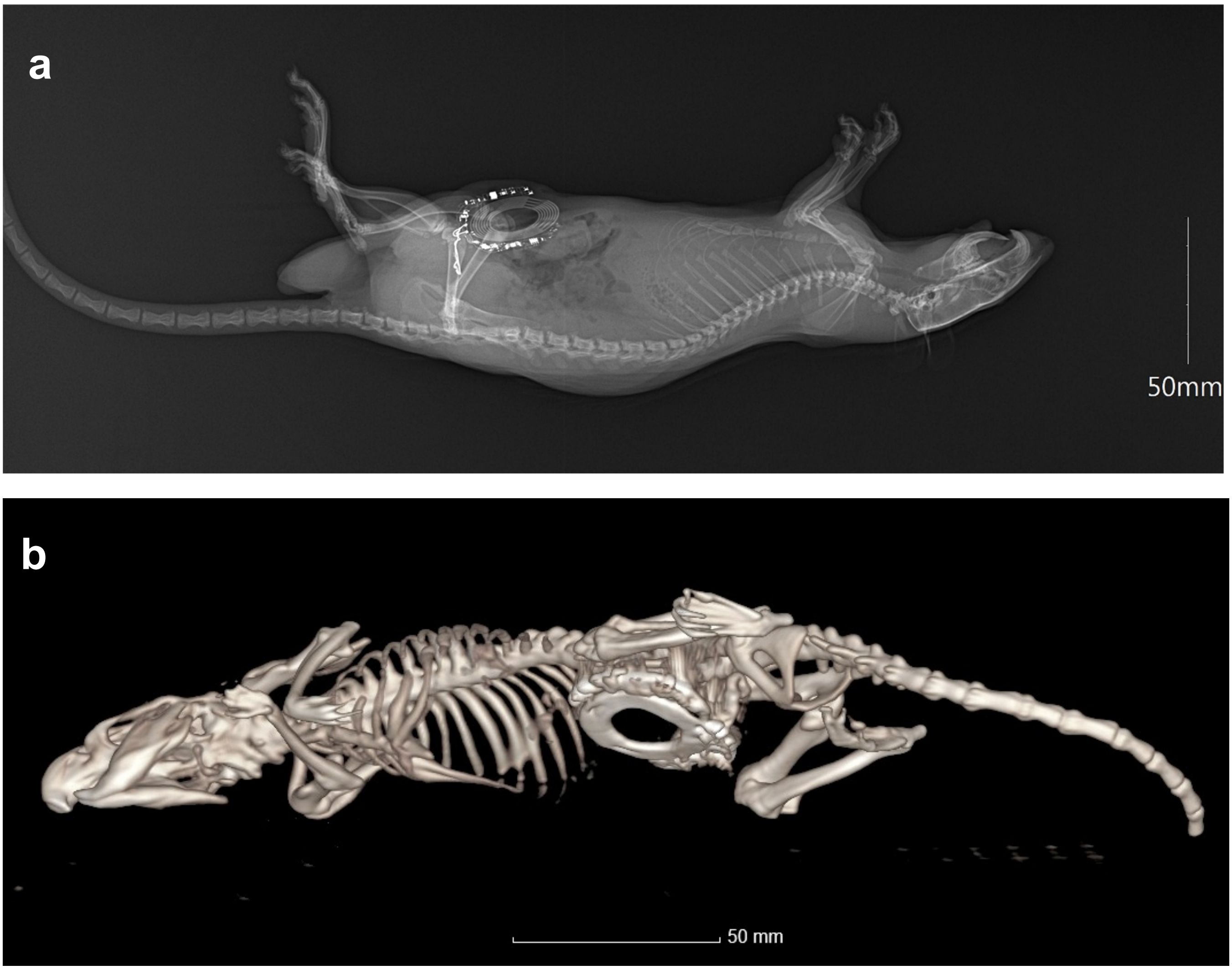

Fig. S7. Medical images of the WIPES device, composed of Rx coils with different turns, implanted in rats, with **(a)** showing X-ray images, and **(b)** showing CT modeling images.

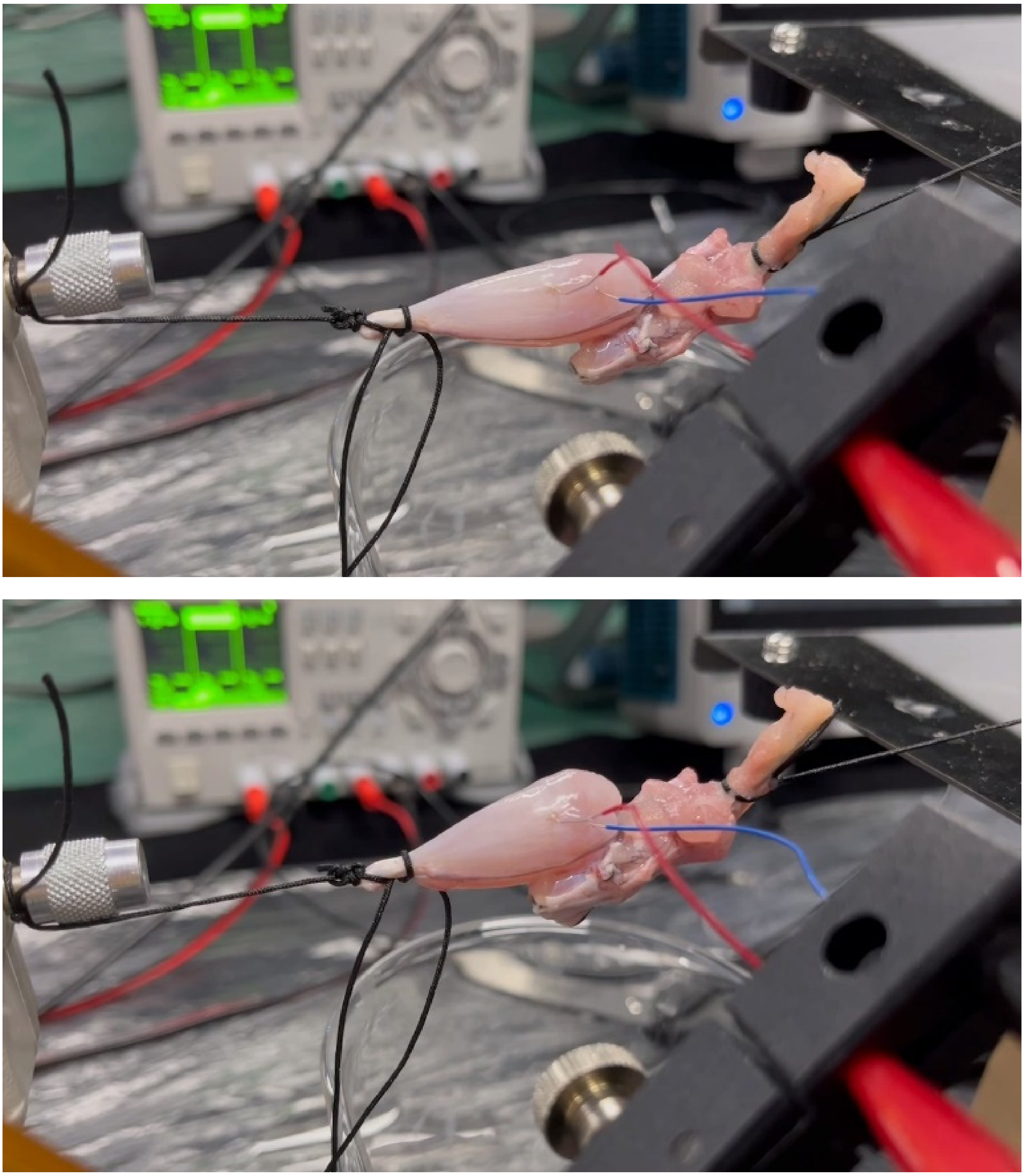

Fig. S8. Preliminary experiment of electrically stimulating the gastrocnemius muscle of a bullfrog using the WIPES device. **(a)** Showing the relaxed state of the gastrocnemius muscle when the WIPES device is inactive. **(b)** Showing the contracting state of the gastrocnemius muscle when the WIPES device is active.

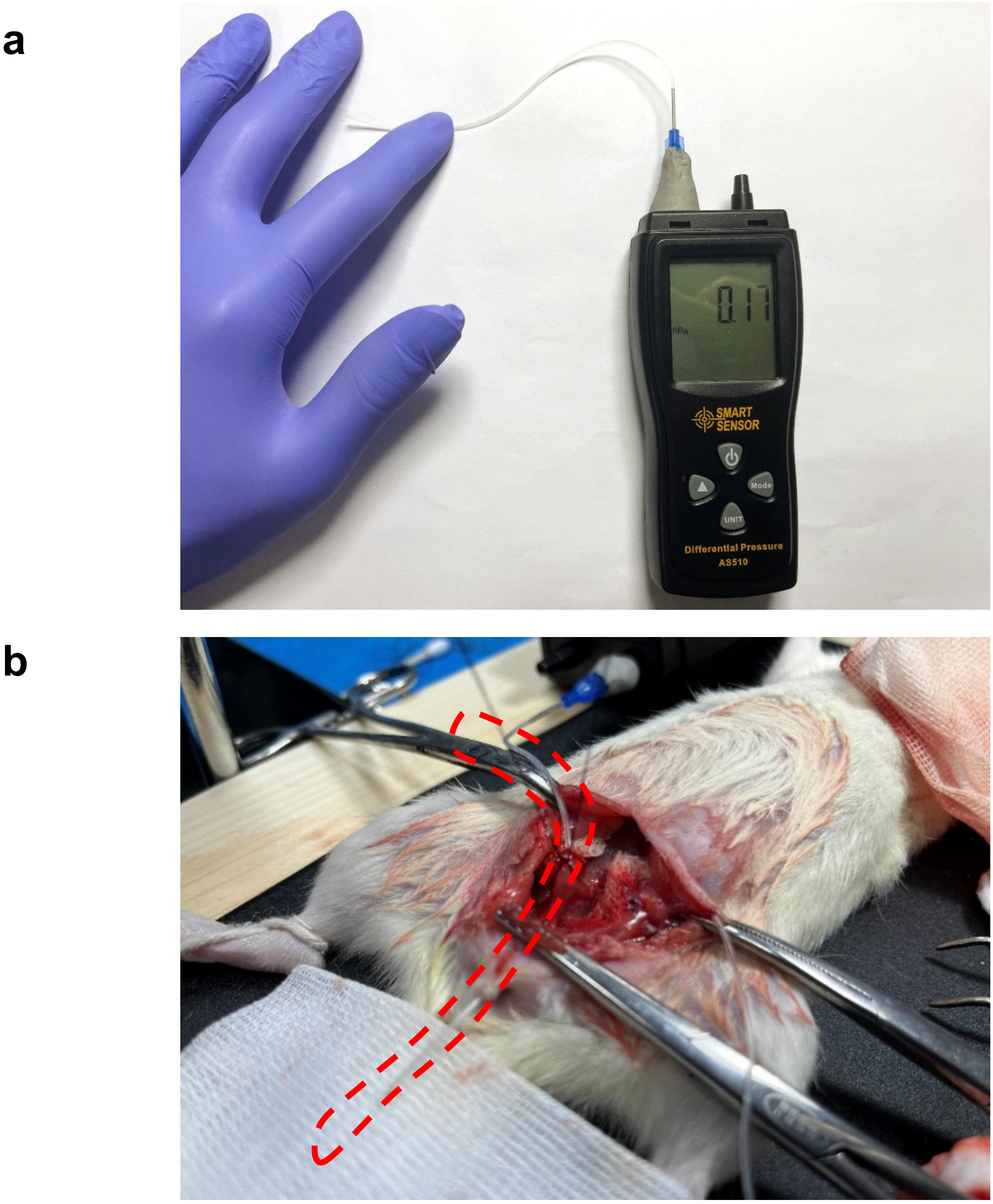

Fig. S9. The pressure measurement process using manometer with a single-ended silicone tube, sealed at the left end with clay. **(a)** The manometer shows a significant pressure change when an external pressure is applied to the tube by manual operation. **(b)** The tube is inserted into the rat's urethra and bladder for ex vivo pressure measurement.

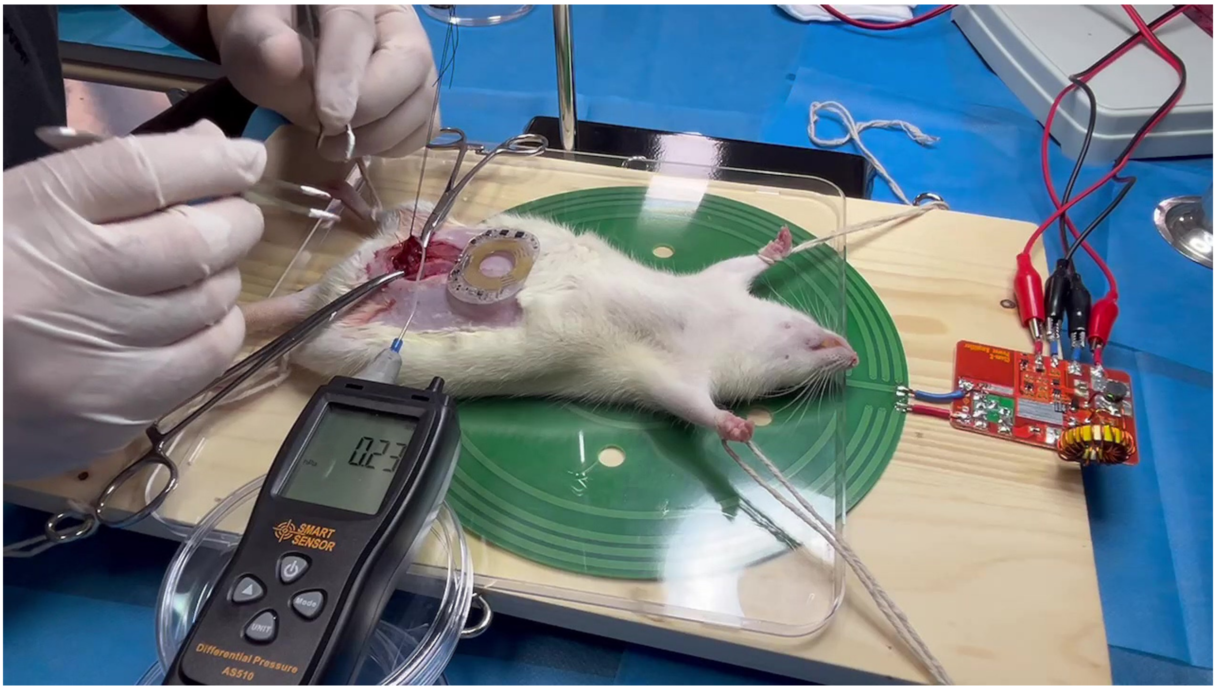

Fig. S10. A rat undergoing external electrostimulation using the WIPES implant.

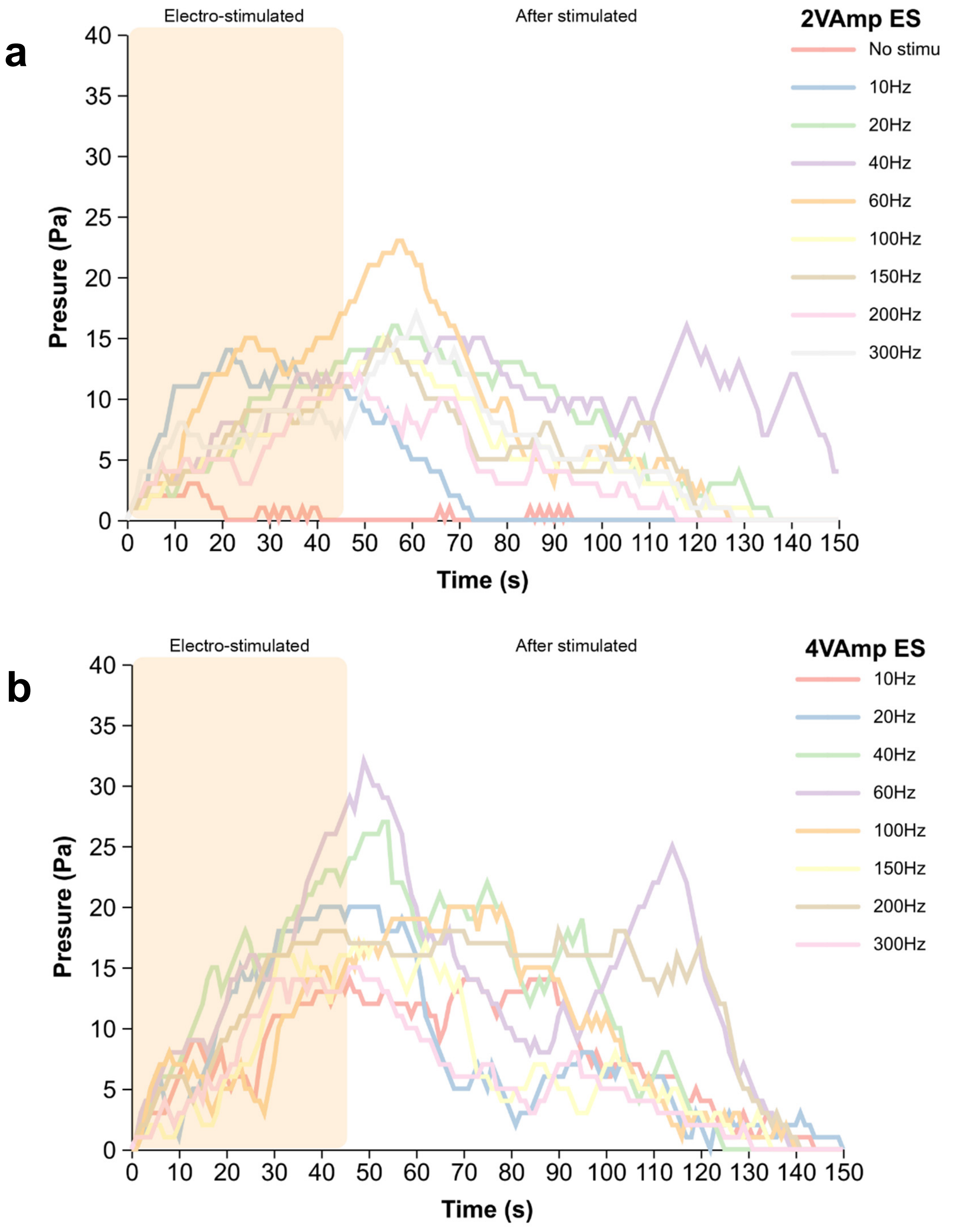

Fig. S11. The effects of varying electrostimulation frequencies and amplitudes, generated by a stable pulse output device, on the contraction pressure of the rat urethral sphincter. **(a)** Showing the stimulation condition at a 2-V amplitude, and **(b)** Showing the stimulation condition at a 4-V amplitude.

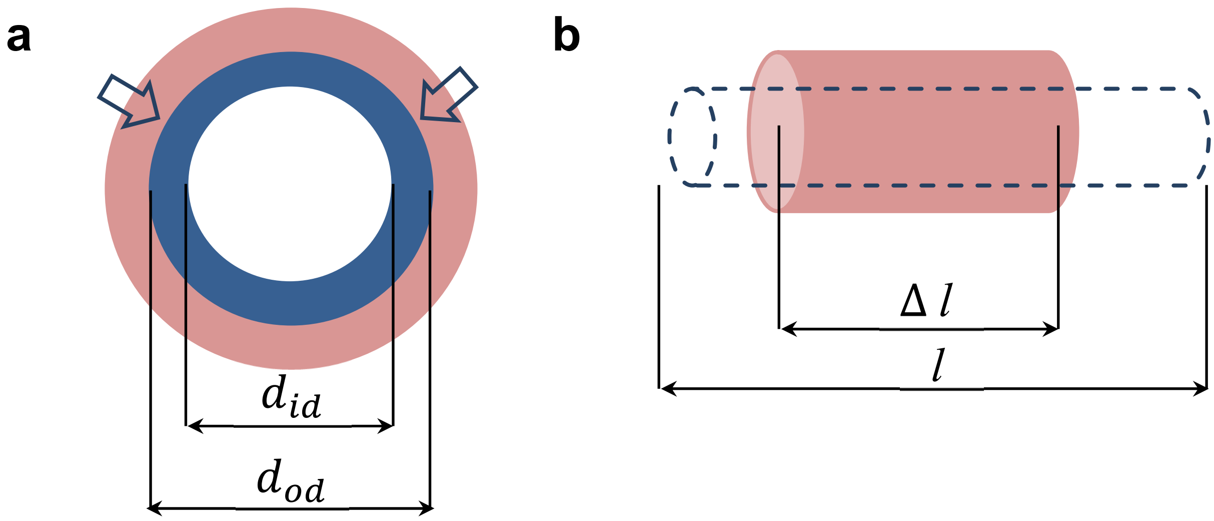

Fig. S12. Schematic diagram of the silicone catheter in the rat urethra. **(a)** The cross-sectional view, and **(b)** The longitudinal view.

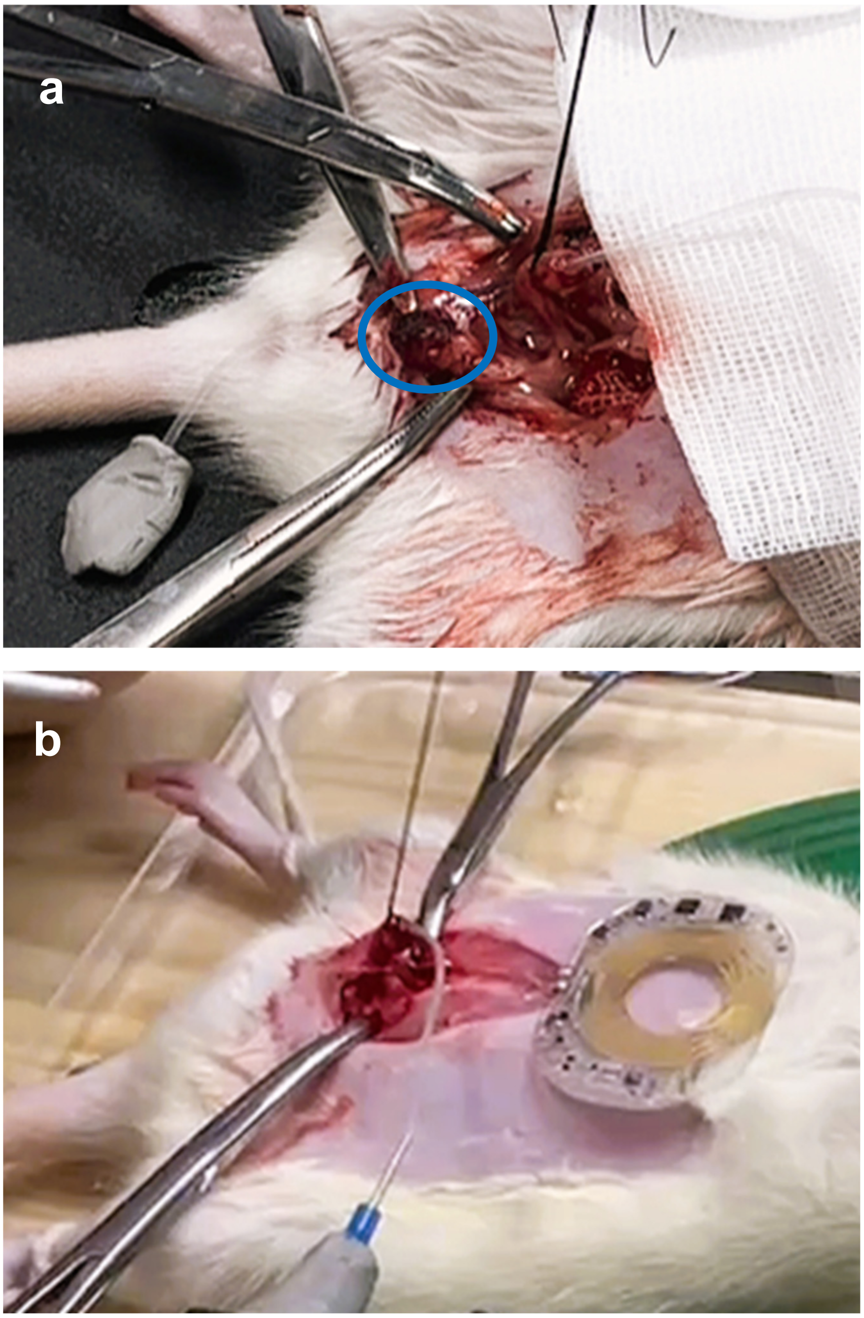

Fig. S13. The benefit of using an outer envelope frequency (1 Hz) in electrostimulation on maintaining the viability of biological tissues. **(a)** Without the use of an outer envelope frequency, continuous electrostimulation causes damage to the rat's urethral sphincter muscle (indicated by the blue circle, where muscle burns are visible). **(b)** When the outer envelope frequency is incorporated into the design, it helps maintain the muscle's viability during prolonged electrostimulation therapy, preventing tissue damage.

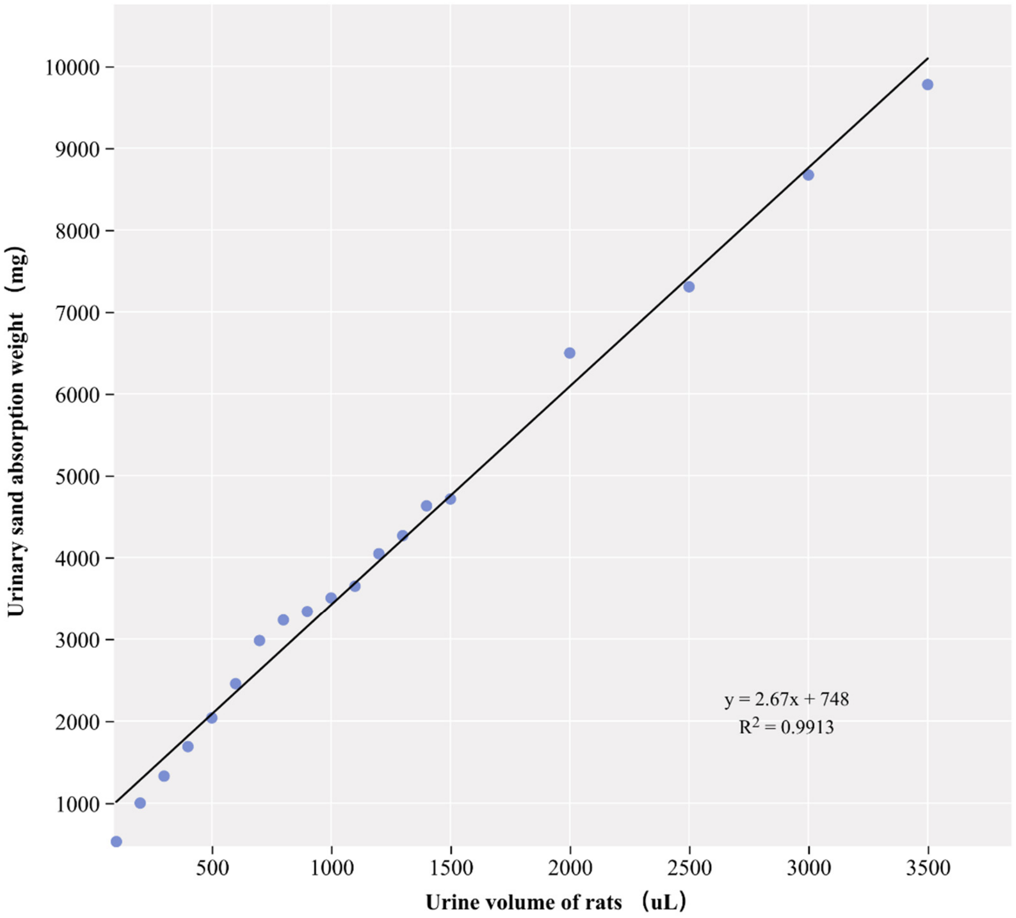

Fig. S14. Calibration curve relating absorbent sand aggregate weight to added water volume. Each dot represents one independent calibration measurement (n = 19) obtained by dispensing a known volume of water onto the absorbent sand and weighing the resulting aggregate. The solid line shows an ordinary least-squares linear fit ($y = 2.67x + 748$; $R^2 = 0.991$). This calibration was used to convert sand aggregate weight to liquid volume in subsequent urine-sand assays.

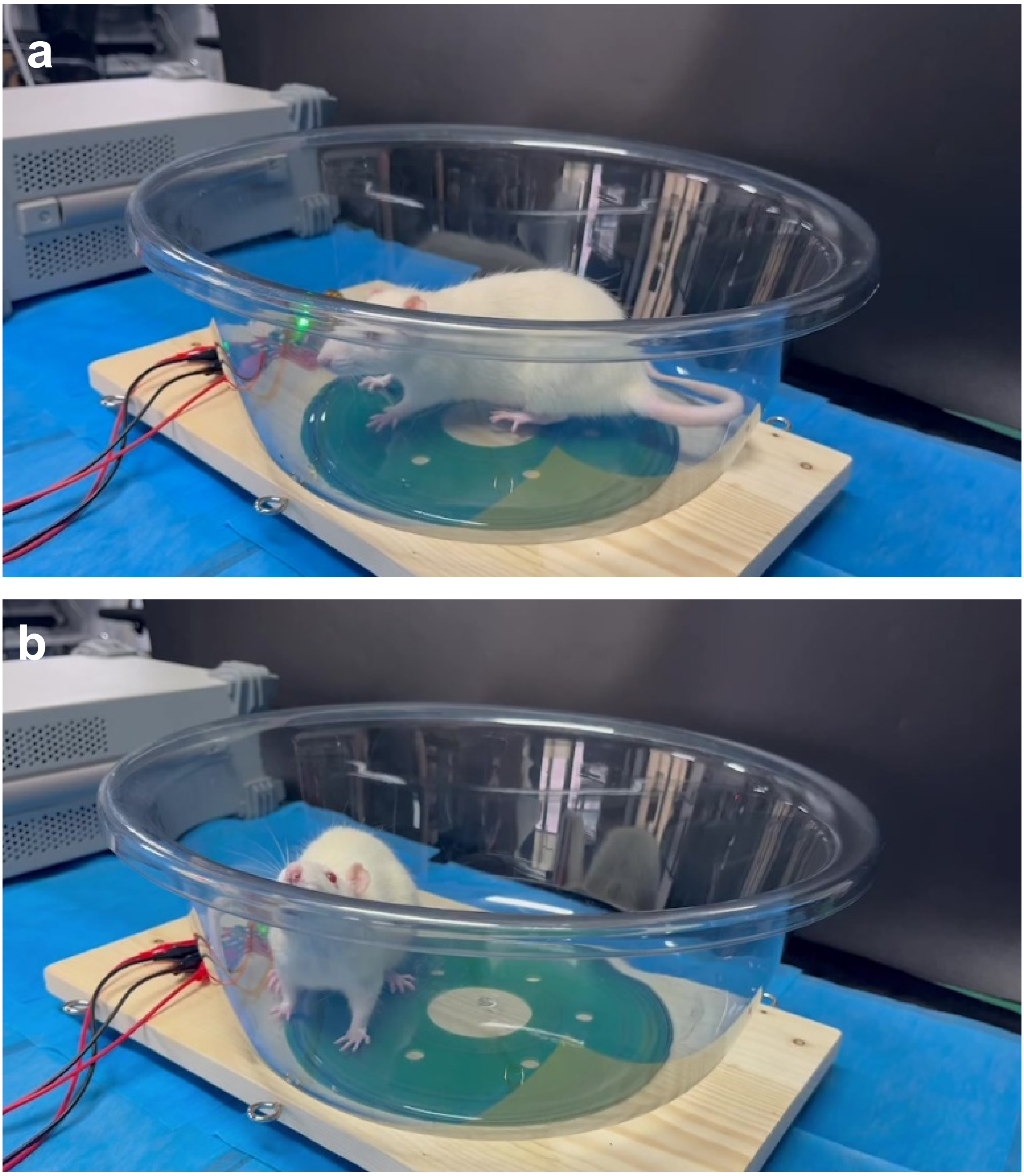

Fig. S15. The conditions of rats with and without the implanted WIPES device on the alternating magnetic field Tx coil. **(a)** Showing a rat without the implanted device on the active Tx coil, and **(b)** Showing a rat with an implanted device on the active Tx coil, where the WIPES device is under operation.

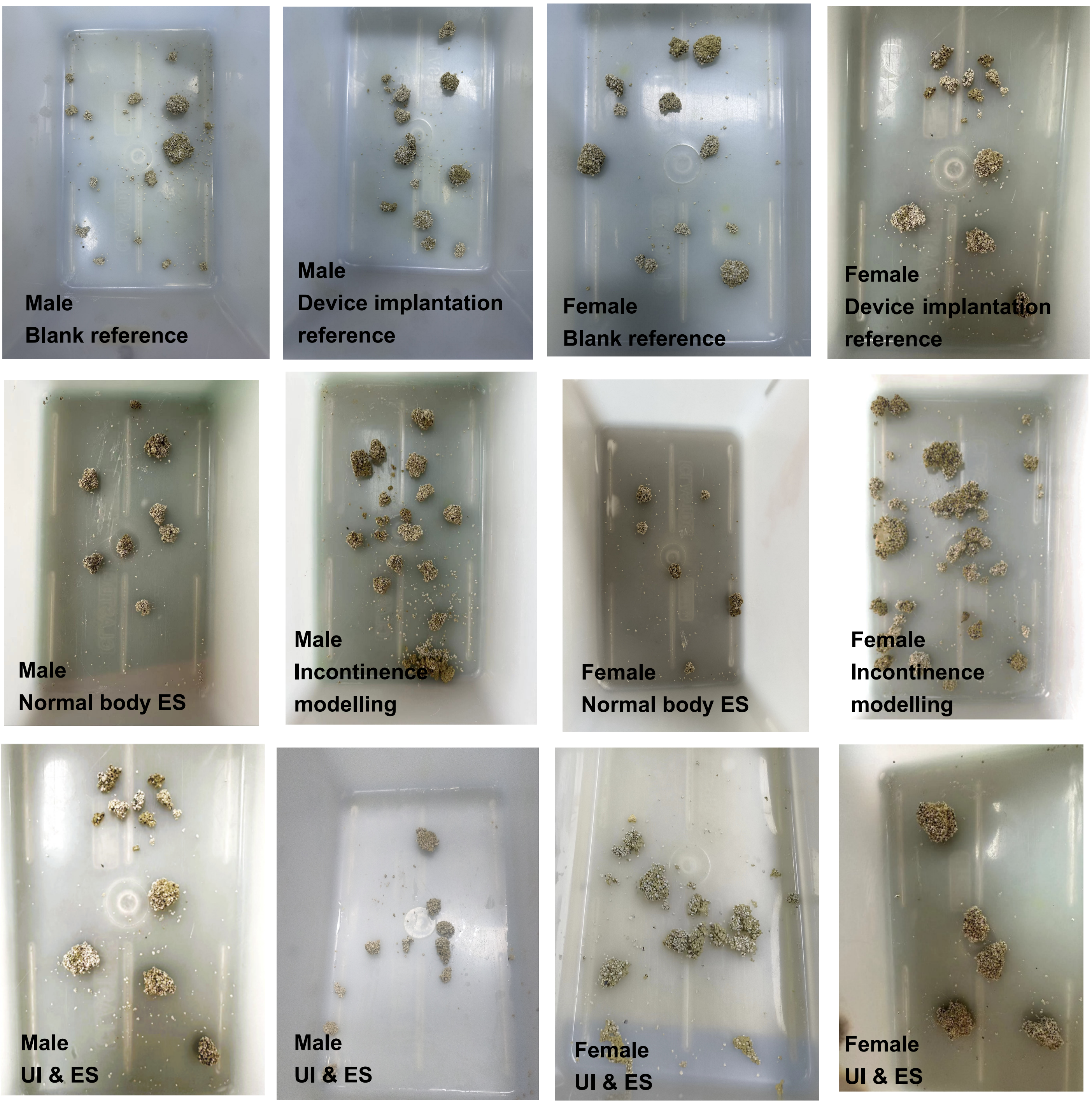

Fig. S16. Images of partially collected urinary sand masses.

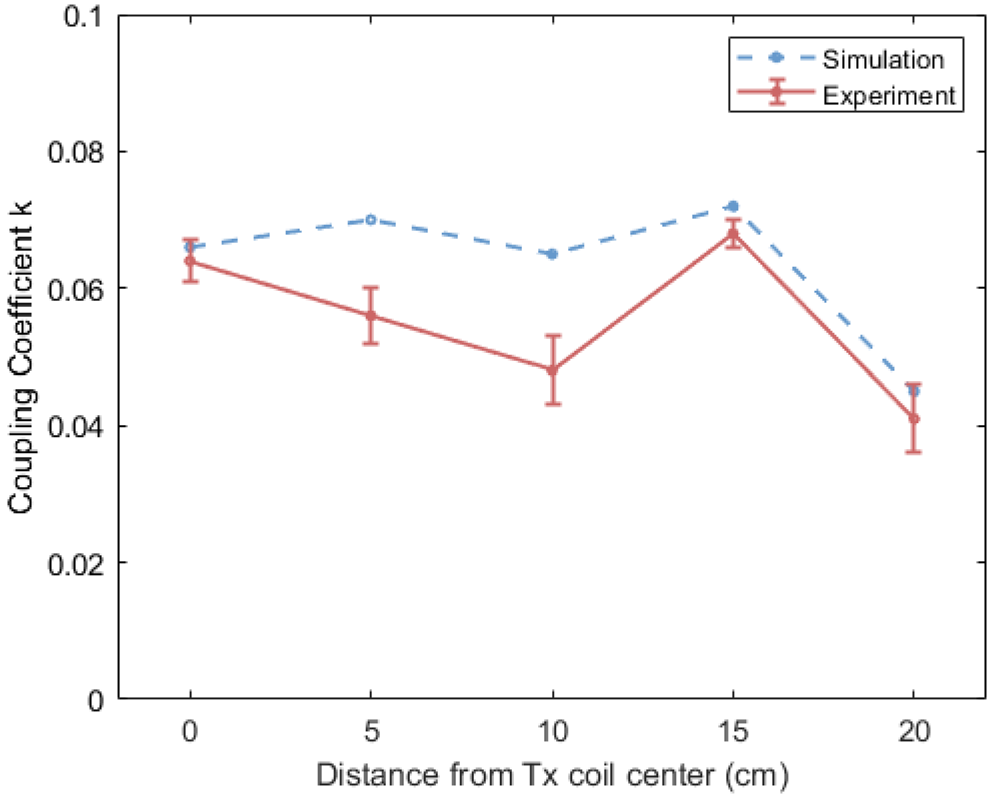

Fig. S17. The mutual inductive coupling coefficient (*k*) is one of the most critical parameters affecting the energy transfer efficiency between the Tx and Rx coils in the WPT system. To further characterize the stability of transmission efficiency at different positions, the horizontal displacement of the Rx coil from the center of the Tx coil was varied while maintaining a fixed vertical distance of 3 cm from the Tx coil plane. For experiments, lines show the mean ± s.d.; n = 6 independent technical replicates per x-axis condition, obtained by repeating the WIPES wireless power-transfer measurement (unit of study: one independent WPT measurement run). Simulation results are deterministic (single values).

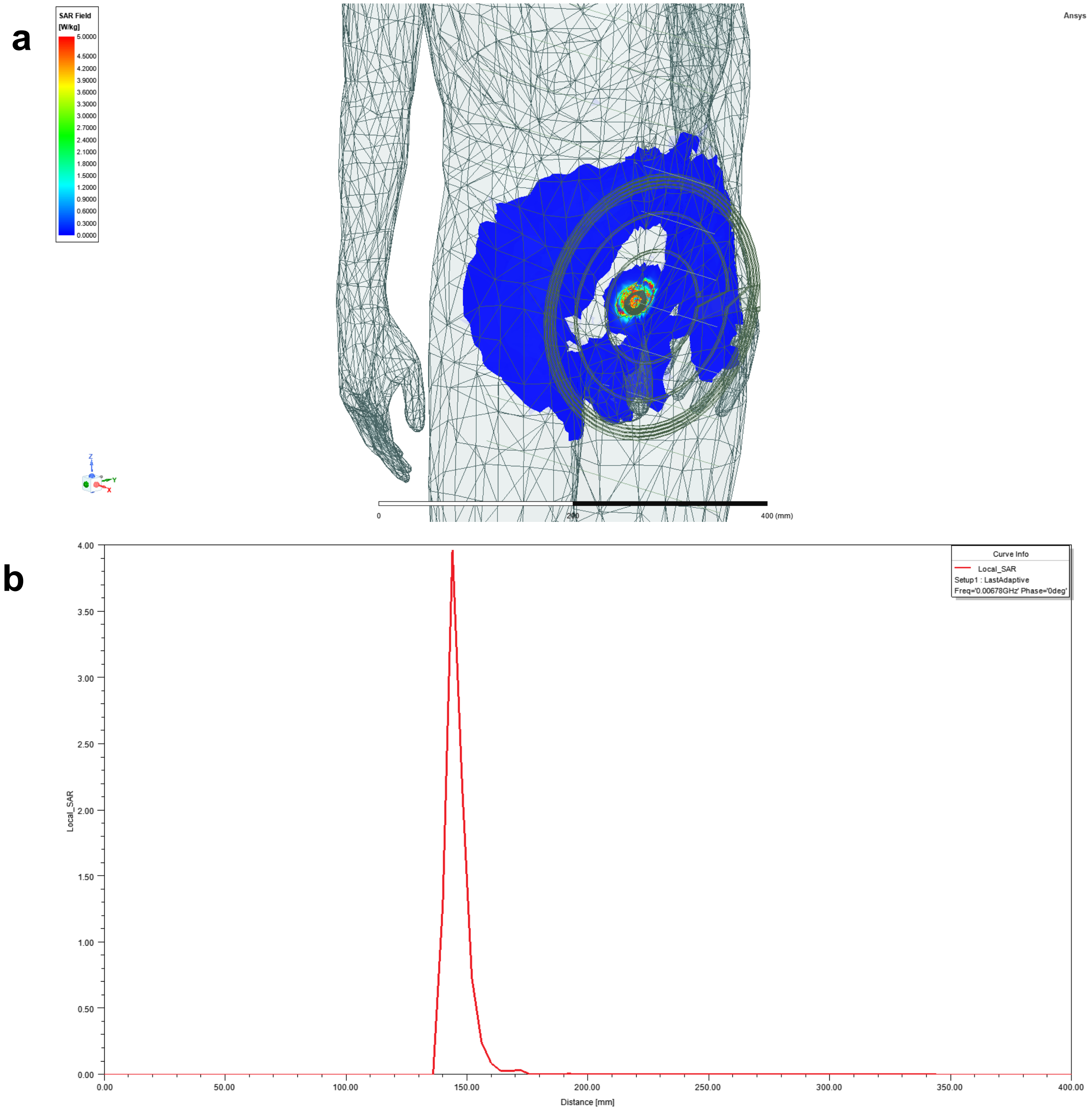

Fig. S18. SAR simulation of the WIPES device after implantation in the human body using HFSS. **(a)** Showing the SAR distribution within the human body, and **(b)** Showing the peak SAR inside the body.

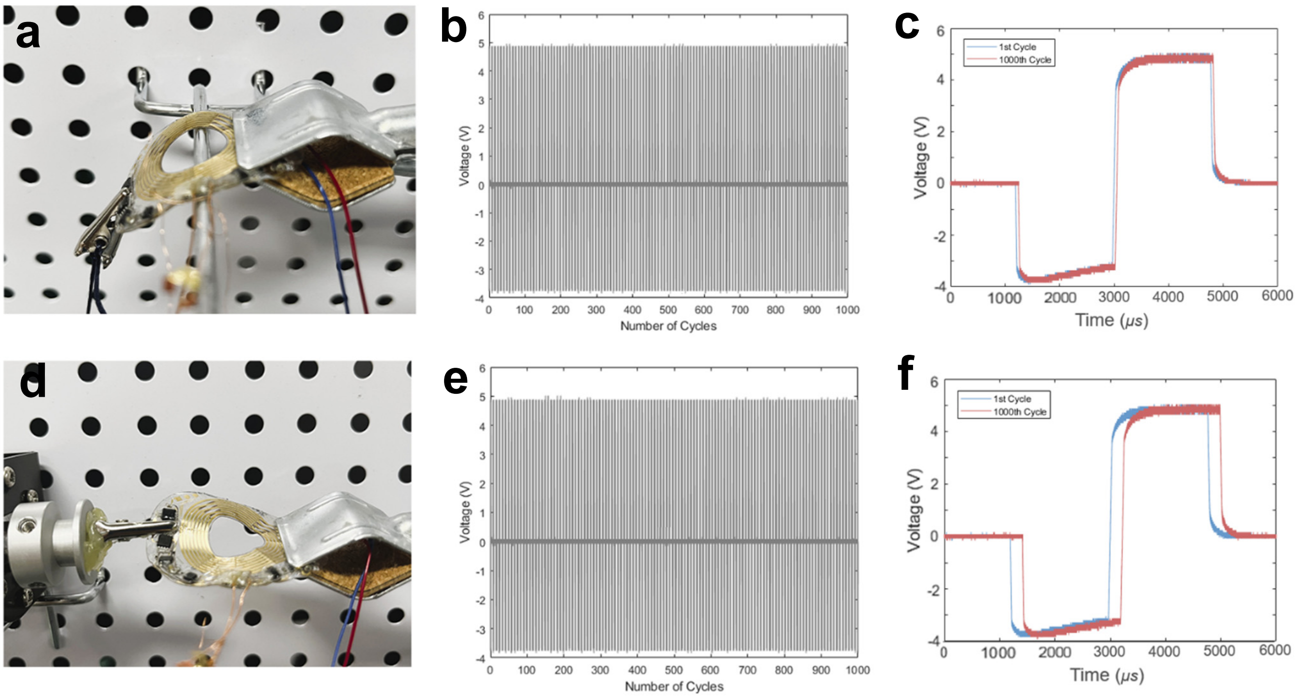

Fig. S19. Cyclical bending and twisting test. **(a)** Setup of the bending experiment, **(b)** Stimulation pulse during 1000 bending cycles, **(c)** Variation of stimulation pulse after 1000 bending cycles, **(d)** Setup of the twisting experiment, **(e)** Stimulation pulse during 1000 twisting cycles, **(f)** Variation of stimulation pulse after 1000 twisting cycles.

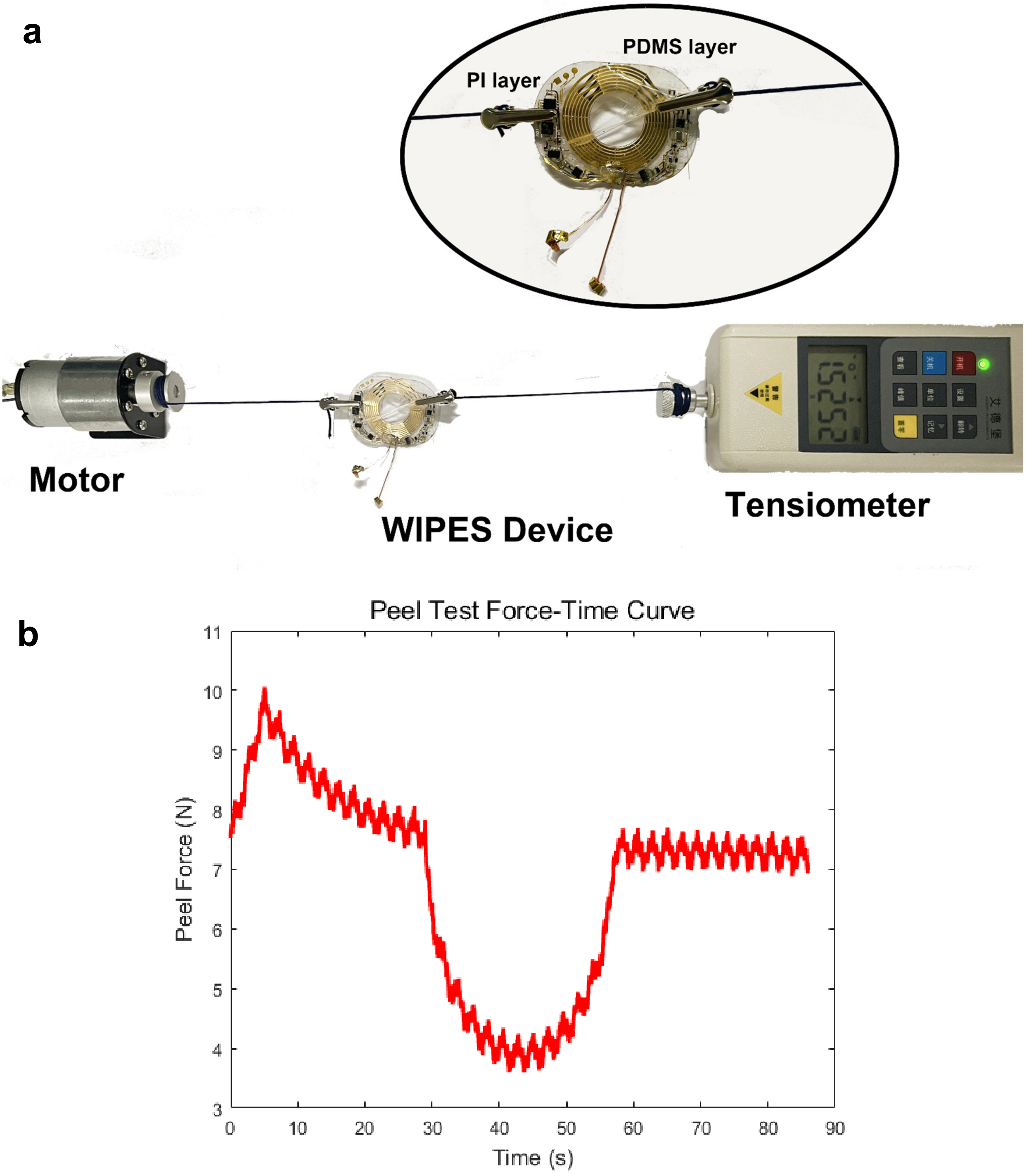

Fig. S20. Interlayer adhesion strength test of WIPES. **(a)** 180° peel test setup separating PDMS and PI layers. **(b)** Representative force–time curve showing fluctuations associated with the central hollow structure.

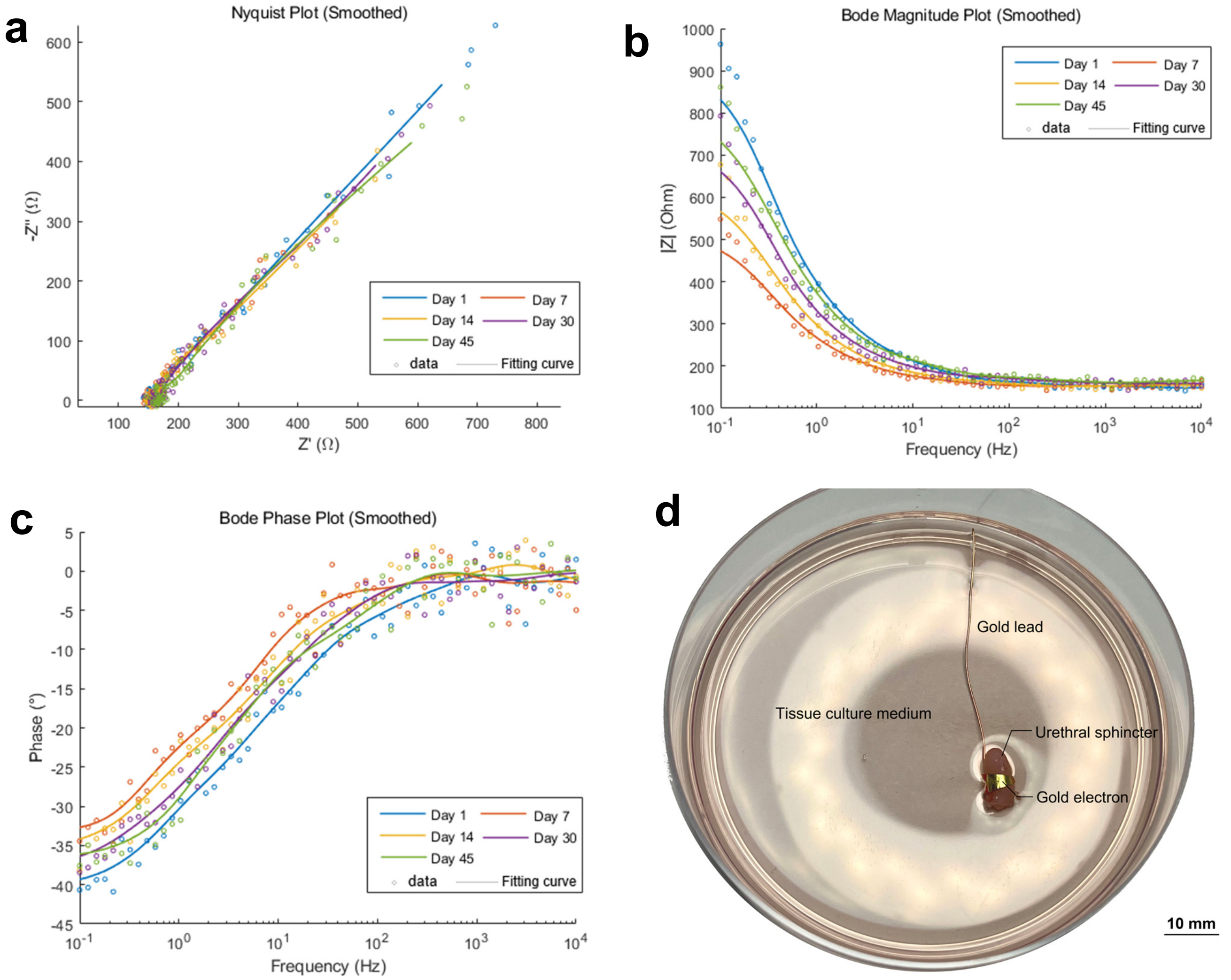

Fig. S21. Electrochemical impedance spectroscopy (EIS) analysis of the WIPES at different time points post-implantation in PBS. **(a)** Nyquist plot: Real versus imaginary impedance components were measured at Days 1, 7, 14, 30, and 45. The raw data are presented as scatter points, and the smoothed curves were obtained using Gaussian filtering. The impedance arc decreases progressively over time, indicating reduced polarization resistance and enhanced interfacial charge transfer. The Day 1 profile shows the highest impedance, suggesting significant interfacial resistance in the initial stage, while the Day 45 curve exhibits a much flatter profile, demonstrating long-term electrochemical stability. **(b)** Bode magnitude plot: The magnitude of the complex impedance $|Z|$ is plotted against frequency. All curves show a typical profile with high impedance at low frequencies and plateauing at high frequencies, reflecting capacitive and resistive behavior, respectively. As time progresses, the overall impedance values, particularly in the low-frequency region, decrease significantly. This trend indicates a gradual reduction in interfacial resistance, likely due to the stabilization of the electrochemical environment around the implant. **(c)** Bode phase plot: The phase angle of the impedance is shown across frequency. All time points demonstrate a capacitive response at low frequencies (with more negative phase angles) and trend toward 0°at higher frequencies. With increasing implantation duration, the phase angle slightly increases, suggesting a transition toward more resistive and stable interfacial characteristics. These findings confirm improved interface stability and sustained electrical functionality of the WIPES during long-term implantation. **(d)** Ex vivo culture of a rat urethral sphincter immersed in cell medium and circumferentially wrapped by the gold electrode.

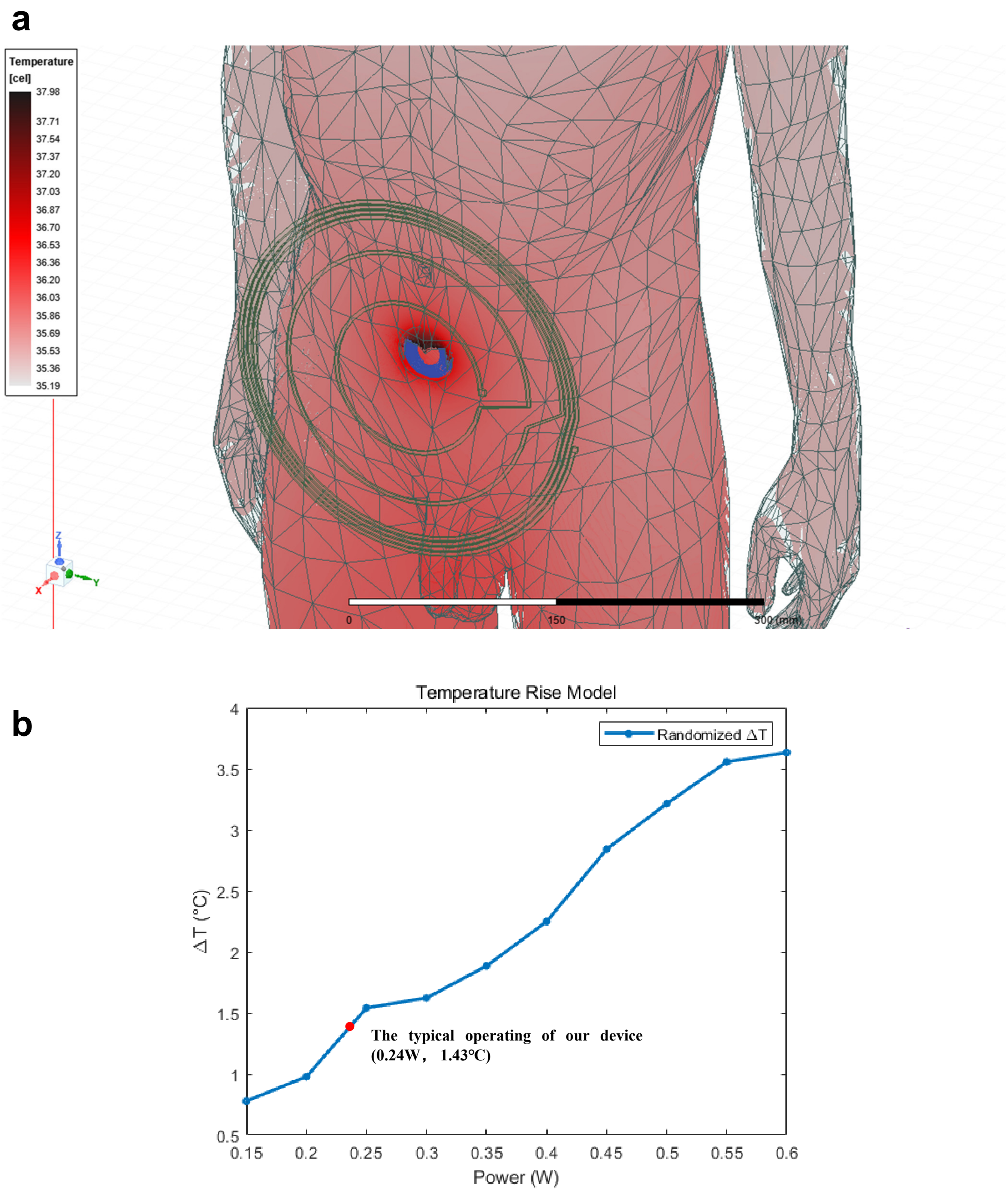

Fig. S22. Thermal simulation and power–temperature characteristics of the WIPES system. **(a)** Temperature distribution simulated using the ANSYS HFSS–Icepak coupled thermal module under 0.45 W received power. The model assumes a baseline tissue temperature of 35 °C, full PDMS encapsulation of the implant, and physiological heat dissipation within surrounding soft tissues. The localized hotspot around the implanted receiver demonstrates a maximum temperature rise of approximately 2.71 °C, remaining below commonly accepted biomedical safety thresholds. **(b)** Modeled temperature rise (ΔT) as a function of received power from 0.15–0.60 W in 0.05-W increments. Randomized variation was introduced to reflect tissue heterogeneity and implant–tissue thermal coupling variability. All data points are deterministic outputs from HFSS simulations; therefore, no sample size (n) or error bars apply.

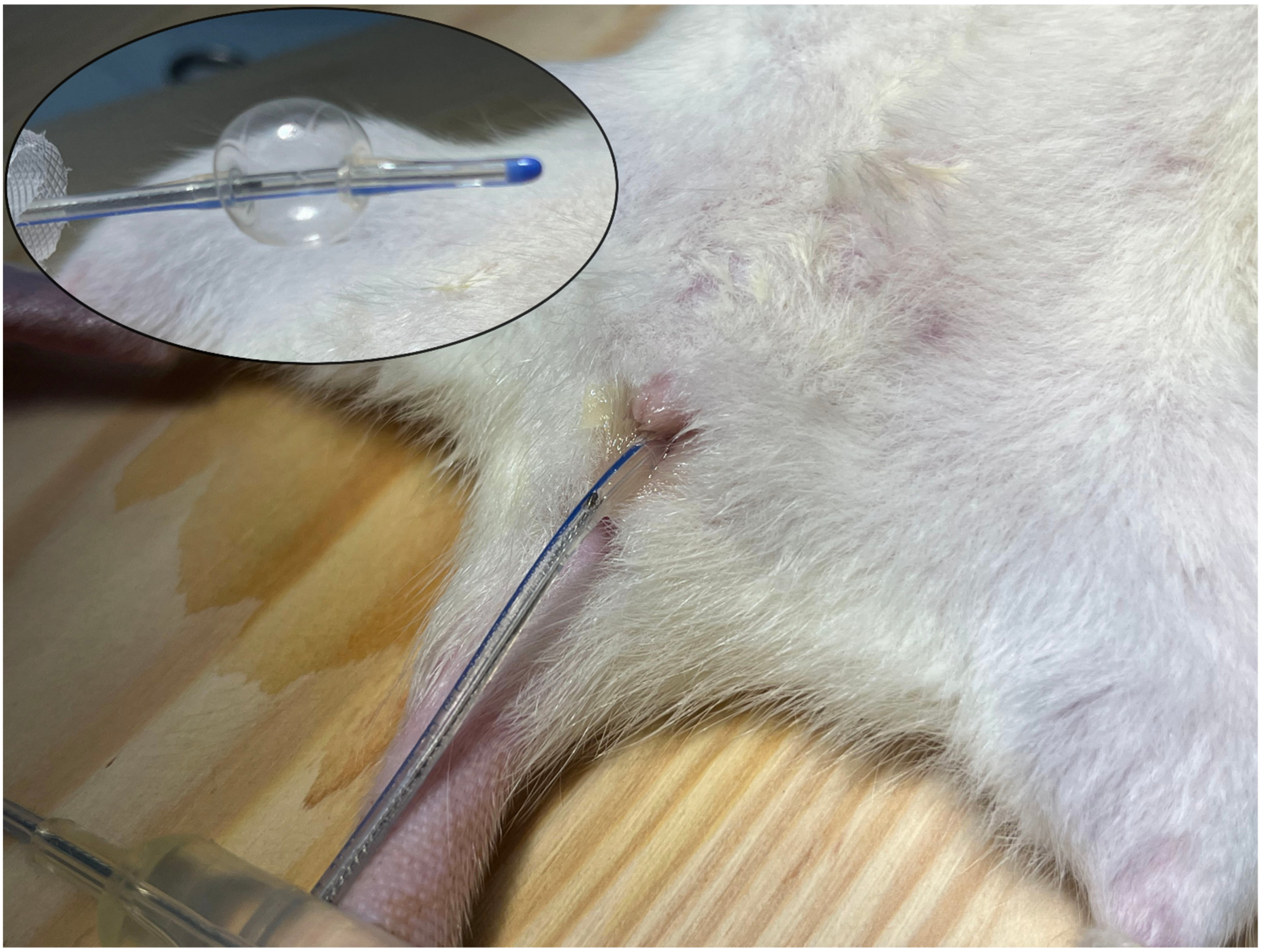

Fig. S23. Urethral balloon dilatation for establishing the SUI model in female rats.

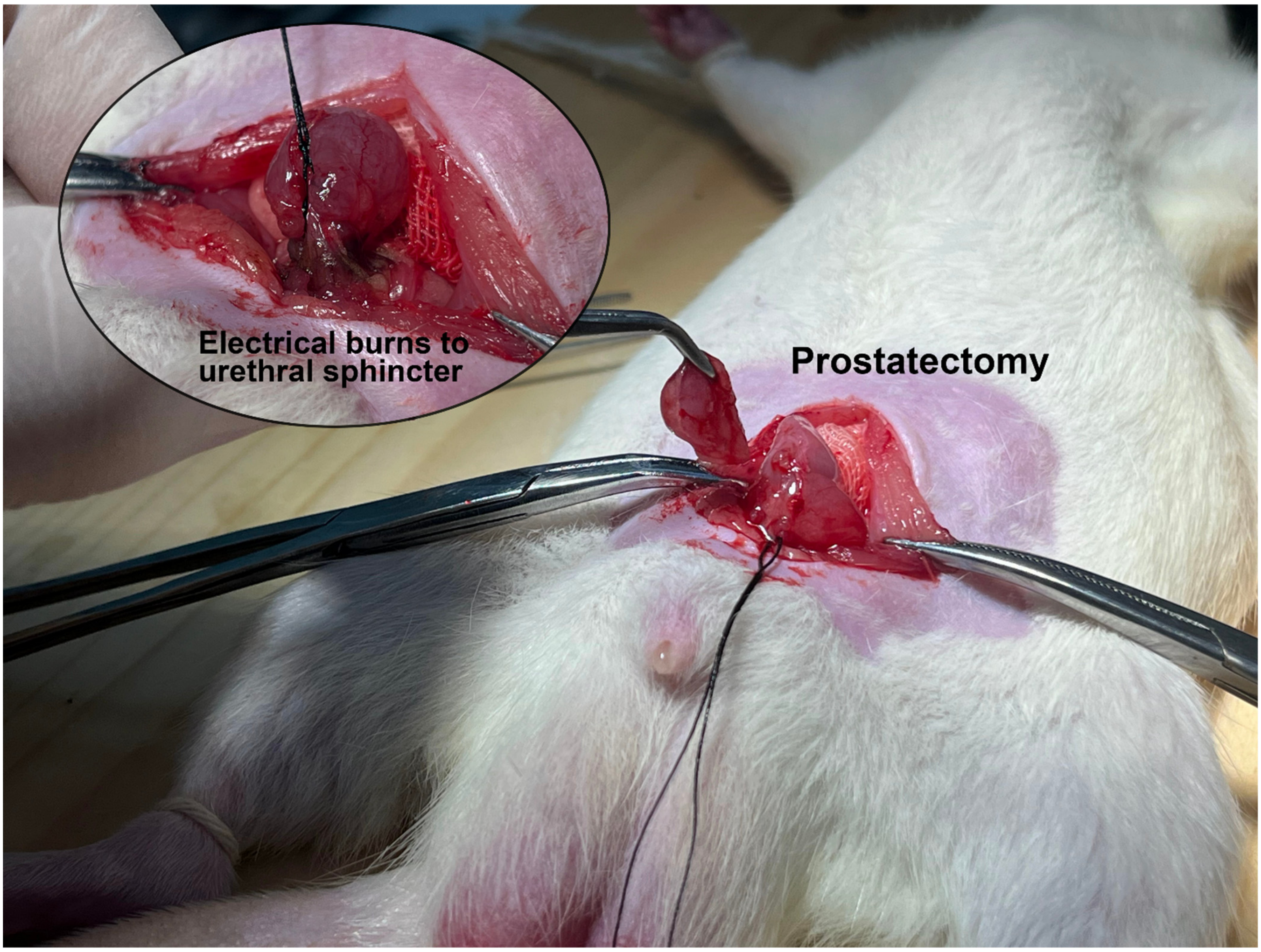

Fig. S24. Intraoperative procedure of combined SUI model induction in male rats through prostatectomy and electrical burns to the urethral sphincter and associated nerves.

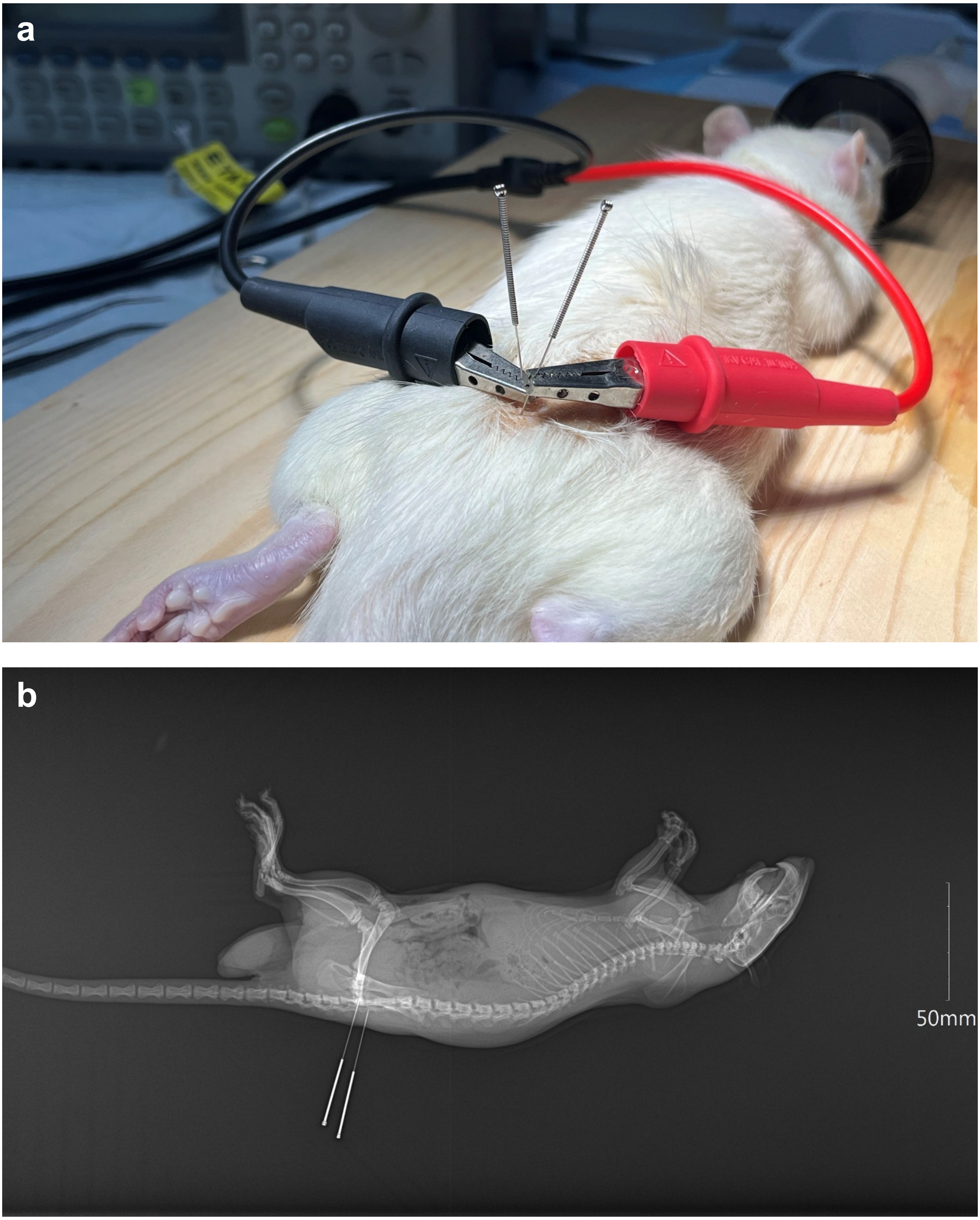

Fig. S25. Simulation of sacral nerve stimulation (SNS) in rats. **(a)** Experimental setup showing transcutaneous sacral nerve stimulation via bilateral needle insertion and external pulse connection. **(b)** X-ray image confirming needle positioning adjacent to the sacral nerve plexus at the S1–S2 level.

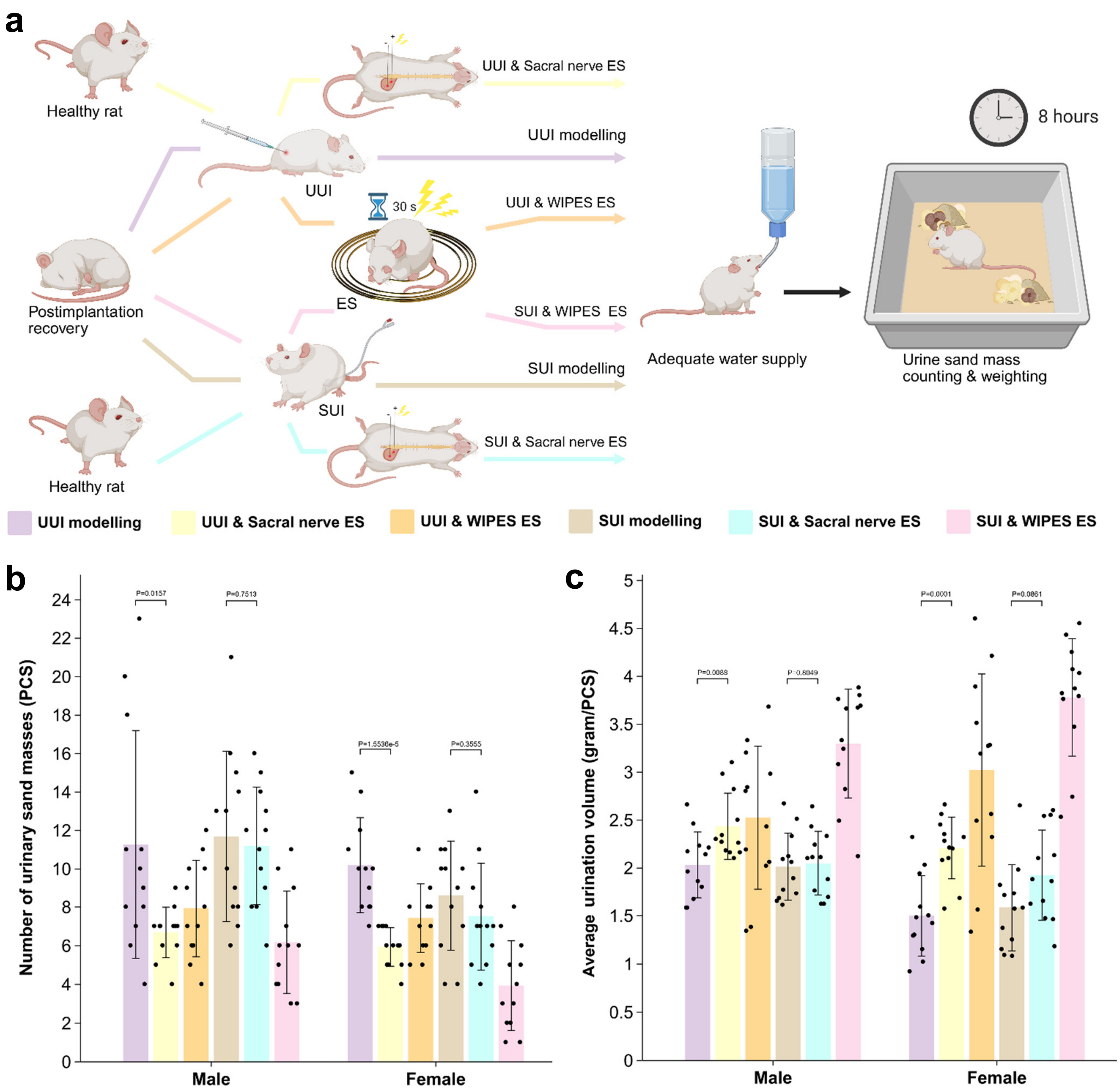

Fig. S26. Comparison with WIPES and traditional SNS for UUI or SUI. **(a)** Workflow, experimental design with six groups: UUI modelling, UUI & Sacral nerve ES, UUI & WIPES ES, SUI modelling, SUI & Sacral nerve ES, SUI & WIPES ES. **(b)** Number of urinary sand masses (PCS), indicating urination frequency, for male and female rats. **(c)** Average urination volume (g/PCS), indicating effective bladder capacity, for male and female rats. For (b) and (c), dots represent individual urine-sand collection measurements (n = 12 measurements per group per sex) obtained from N = 8 biologically independent rats per group per sex; multiple measurements may originate from the same rat. To avoid pseudo-replication, repeated measurements were averaged within each rat to yield one value per animal (unit of study: rat) for statistical testing. Bars show mean ± s.d. Statistical analysis was performed within each sex using one-way ANOVA with Tukey's multiple-comparison test; exact Tukey-adjusted P values are indicated above brackets. Graphical elements in Fig. S26 is created in BioRender. Dwad, D. (2026) [https://biorender.com/eknsmzf]

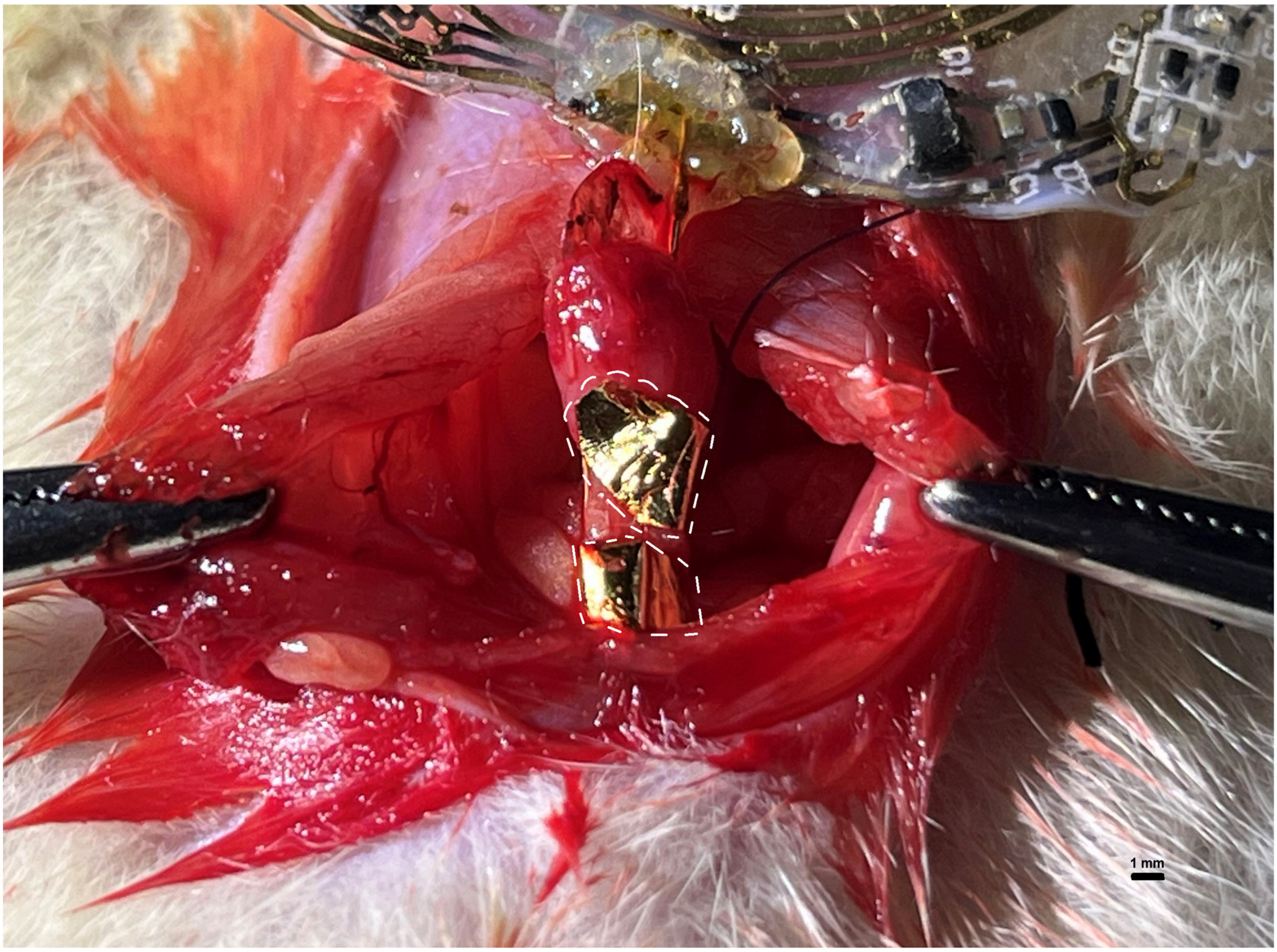

Fig. S27. Intraoperative view of WIPES electrode wrapping the external urethral sphincter (EUS). Open perineal exposure showing the C-shaped gold stimulator conformally wrapped around the EUS (non-penetrating surface contact). The white dashed outline delineates the gold electrode area. The flexible main module is visible superiorly. This image illustrates the intended circumferential apposition without constrictive ligation.

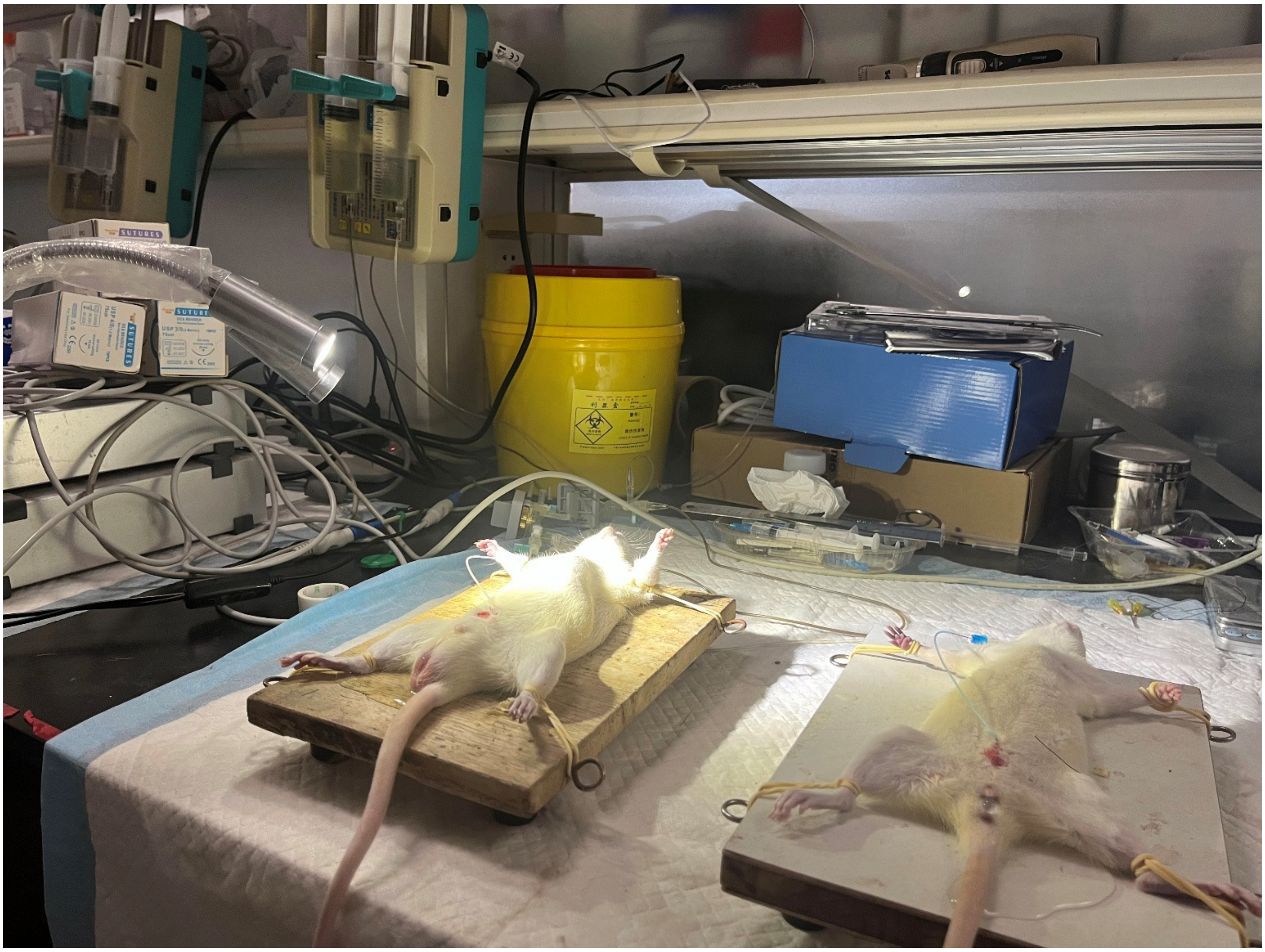

Fig. S28. Experimental setup for cystometry in male and female rats. The animals were anesthetized and fixed in a supine position during bladder infusion with physiological saline at a constant rate of 5 mL/h to record cystometric pressure responses under controlled laboratory conditions.

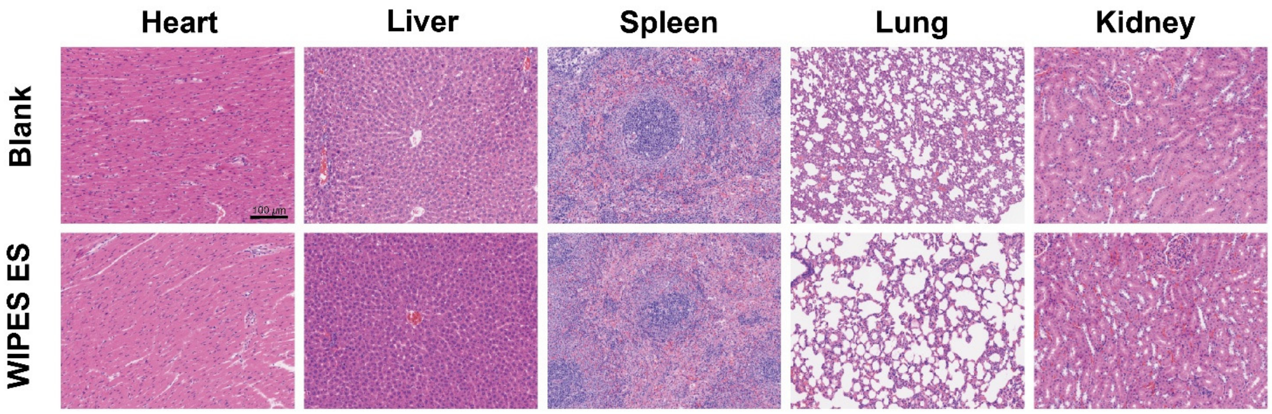

Fig. S29. Organ histological analysis. Post-experiment to evaluate potential systemic effects. H&E staining of major organs (heart, liver, spleen, lungs, and kidneys) revealed no pathological damage in any group.

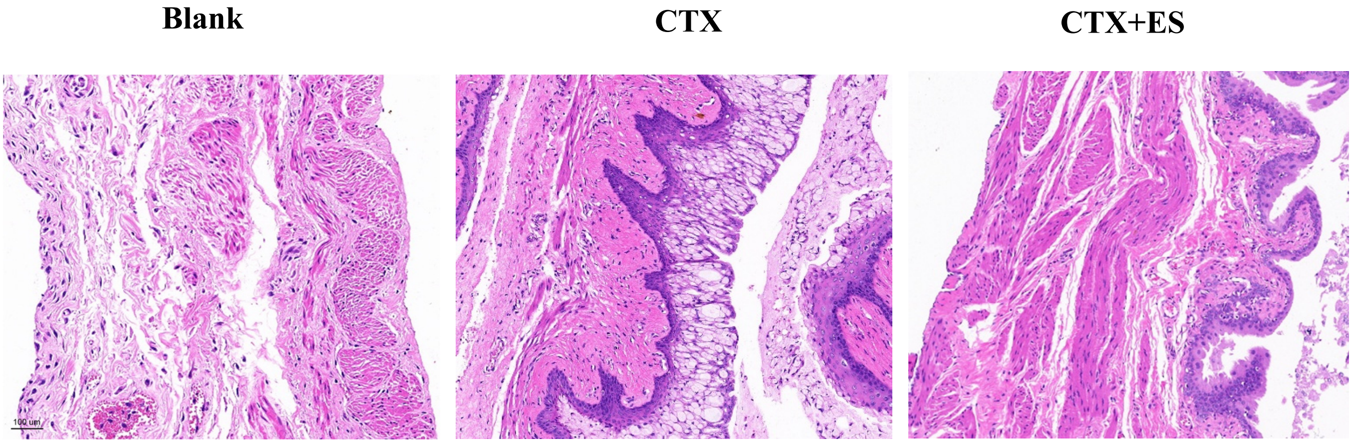

Fig. S30. Blank reference, CTX modeling, and CTX+ES groups' HE staining sections of bladder tissues.

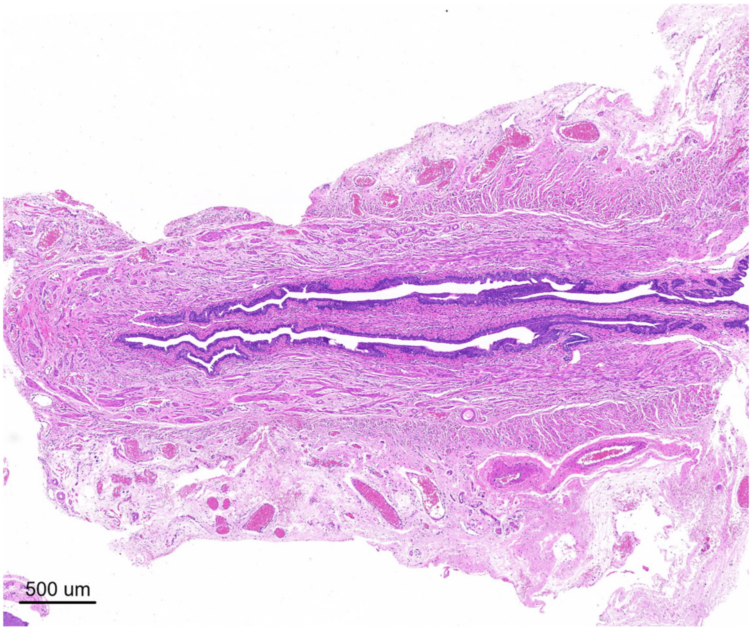

Fig. S31. HE staining of the complete urethral cross-section tissue of female rats after external urethral intubation.

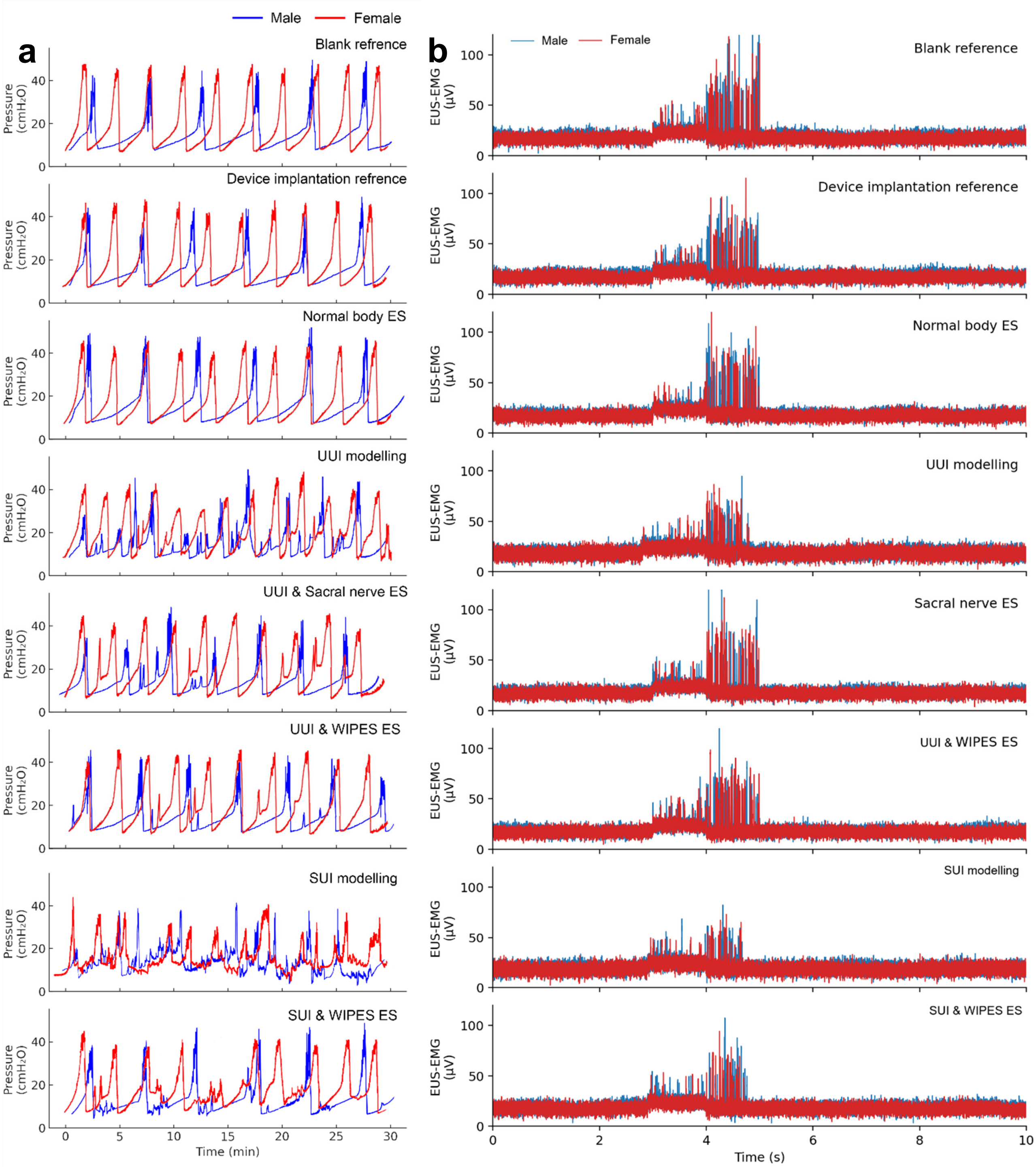

Fig. S32. CMG and EUS-EMG characteristics across UUI and SUI models with different stimulation conditions. (a) Representative CMG recordings showing intravesical pressure changes over time in male (blue) and female (red) rats under different experimental conditions, including blank reference, device implantation reference, normal body ES, UUI modelling, UUI with sacral nerve ES, UUI with WIPES stimulation, SUI modelling, and SUI with WIPES stimulation. These traces illustrate sex-dependent differences in voiding patterns and pressure dynamics, as well as condition-dependent alterations in voiding regularity and peak pressure profiles. (b) Representative external urethral sphincter electromyography (EUS-EMG) recordings from male (blue) and female (red) rats corresponding to the conditions shown in (a). The EMG traces highlight differences in burst amplitude, firing density, and temporal distribution of sphincter activity across disease models and stimulation paradigms, providing complementary neuromuscular context to the CMG measurements.
