# Supplementary material for: Wireless electrostimulation implants enable sphincter neuromuscular improvement toward mixed urinary incontinence
Source: Nat Commun. 2026 Apr 15;17:5226. doi: 10.1038/s41467-026-71532-7 (PMC13260462; doi:10.1038/s41467-026-71532-7)
Supplement: Supplementary file 2 — Reporting Summary [file 41467_2026_71532_MOESM2_ESM.pdf]

nature portfolio

Corresponding author(s): Metin Sitti

Last updated by author(s): Feb 6, 2026

Reporting Summary

Nature Portfolio wishes to improve the reproducibility of the work that we publish. This form provides structure for consistency and transparency in reporting. For further information on Nature Portfolio policies, see our Editorial Policies and the Editorial Policy Checklist.

Statistics

For all statistical analyses, confirm that the following items are present in the figure legend, table legend, main text, or Methods section.

n/a	Confirmed	
☐	☒	The exact sample size (*n*) for each experimental group/condition, given as a discrete number and unit of measurement
☐	☒	A statement on whether measurements were taken from distinct samples or whether the same sample was measured repeatedly
☐	☒	The statistical test(s) used AND whether they are one- or two-sided<br>*Only common tests should be described solely by name; describe more complex techniques in the Methods section.*
☒	☐	A description of all covariates tested
☐	☒	A description of any assumptions or corrections, such as tests of normality and adjustment for multiple comparisons
☐	☒	A full description of the statistical parameters including central tendency (e.g. means) or other basic estimates (e.g. regression coefficient) AND variation (e.g. standard deviation) or associated estimates of uncertainty (e.g. confidence intervals)
☐	☒	For null hypothesis testing, the test statistic (e.g. *F*, *t*, *r*) with confidence intervals, effect sizes, degrees of freedom and *P* value noted<br>*Give P values as exact values whenever suitable.*
☒	☐	For Bayesian analysis, information on the choice of priors and Markov chain Monte Carlo settings
☐	☒	For hierarchical and complex designs, identification of the appropriate level for tests and full reporting of outcomes
☒	☐	Estimates of effect sizes (e.g. Cohen's *d*, Pearson's *r*), indicating how they were calculated

Our web collection on statistics for biologists contains articles on many of the points above.

Software and code

Policy information about availability of computer code

Data collection	CMG and EUS-EMG signals were digitized using a PowerLab data acquisition system and recorded with LabChart (ADInstruments). Electrochemical impedance spectroscopy (EIS) data were acquired using a potentiostat/impedance analyzer (Gamry Reference 600+).
Data analysis	Data processing and statistical analyses were performed in MATLAB (MathWorks, R2021b)and visualized in OriginLab. Histology and immunofluorescence images were quantified using SlideViewer (v2.9) with standardized analysis parameters. Electromagnetic simulations (e.g., SAR) were performed using ANSYS Electronics Desktop (HFSS + Icepak). PCB/circuit layout was designed in Altium Designer (Altium, v21.0.8). Any additional requests for information (including analysis scripts) can be directed to the corresponding authors.

For manuscripts utilizing custom algorithms or software that are central to the research but not yet described in published literature, software must be made available to editors and reviewers. We strongly encourage code deposition in a community repository (e.g. GitHub). See the Nature Portfolio guidelines for submitting code & software for further information.

Data

Policy information about availability of data

All manuscripts must include a data availability statement. This statement should provide the following information, where applicable:

- Accession codes, unique identifiers, or web links for publicly available datasets
- A description of any restrictions on data availability
- For clinical datasets or third party data, please ensure that the statement adheres to our policy

All data supporting the findings of this study are available within the article and its supplementary files. Source data are provided with this paper. Any additional

requests for information can be directed to, and will be fulfilled by, the corresponding authors.

Research involving human participants, their data, or biological material

Policy information about studies with human participants or human data. See also policy information about sex, gender (identity/presentation), and sexual orientation and race, ethnicity and racism.

Reporting on sex and gender	N/A. This study did not involve human participants, human data, or human biological materials.
Reporting on race, ethnicity, or other socially relevant groupings	N/A. This study did not involve human participants, human data, or human biological materials.
Population characteristics	N/A. No human participants were included.
Recruitment	N/A. No human participants were recruited.
Ethics oversight	N/A. Animal experiments were conducted under an approved institutional animal protocol (Ruijin Hospital, SJTU School of Medicine; Approval No. RJ2024051).

Note that full information on the approval of the study protocol must also be provided in the manuscript.

Field-specific reporting

Please select the one below that is the best fit for your research. If you are not sure, read the appropriate sections before making your selection.

- [x] Life sciences
- [ ] Behavioural & social sciences
- [ ] Ecological, evolutionary & environmental sciences

For a reference copy of the document with all sections, see nature.com/documents/nr-reporting-summary-flat.pdf

Life sciences study design

All studies must disclose on these points even when the disclosure is negative.

Sample size	Sample sizes were based on prior literature, pilot experiments and the approved animal protocol; no formal a priori power calculation was performed. For animal experiments, cohorts typically included 8 rats per sex per group, unless otherwise stated. Exact n and the definition of biological vs technical replicates (and unit of study) are reported in the figure legends and Methods.
Data exclusions	No data were excluded post hoc. Exclusion criteria were predefined in the approved protocol (e.g., humane endpoints) and for technical failure (e.g., corrupted recordings, catheter displacement, or unsuccessful staining/sectioning rendering a dataset unusable). Any exclusions (if applicable) are reported in the Methods/figure legends; otherwise all collected data were included.
Replication	Key findings were reproduced across independent animals and, where applicable, independent cell cultures. For histology/ immunofluorescence quantification, multiple non-overlapping fields and ≥2 sections per animal were quantified and averaged to yield one value per animal (unit of study: rat). For in vitro assays, biologically independent cultures were used, with 2–3 technical replicate wells averaged per culture.
Randomization	Animals were allocated to predefined experimental groups according to the approved protocol, with stratification by sex and balancing for age/body weight where applicable. No additional randomization procedure beyond the protocol-defined allocation was applied.
Blinding	Blinding was not feasible for some in vivo procedures due to the nature of surgical implantation and stimulation protocols. However, image quantification used a fixed, standardized pipeline, and assessors were blinded to experimental allocation during segmentation/verification.

Reporting for specific materials, systems and methods

We require information from authors about some types of materials, experimental systems and methods used in many studies. Here, indicate whether each material, system or method listed is relevant to your study. If you are not sure if a list item applies to your research, read the appropriate section before selecting a response.

Materials & experimental systems

n/a	Involved in the study	
[ ]	[x]	Antibodies
[ ]	[x]	Eukaryotic cell lines
[x]	[ ]	Palaeontology and archaeology
[ ]	[x]	Animals and other organisms
[x]	[ ]	Clinical data
[x]	[ ]	Dual use research of concern
[x]	[ ]	Plants

Methods

n/a	Involved in the study	
[x]	[ ]	ChIP-seq
[x]	[ ]	Flow cytometry
[x]	[ ]	MRI-based neuroimaging

Antibodies

Antibodies used	Primary antibodies: c-Fos (rabbit monoclonal, CST; 1:300).<br>PGP9.5 (rabbit anti-PGP9.5, Abcam ab108986; 1:400).<br>GAP-43 (mouse anti-GAP-43, Abcam ab119973; 1:400).<br>nNOS (rabbit anti-nNOS; Abcam ab76067; 1:200).<br>VIP (rabbit anti-VIP; Abcam ab272726; 1:250).<br>ROCK2 (rabbit anti-ROCK2; Abcam ab125025; 1:250).<br>α-SMA (mouse anti-α-SMA; Abcam ab7817; 1:400).<br>Secondary antibodies: Goat anti-rabbit IgG, Alexa Fluor 488 (Invitrogen A11034; 1:1000) — used for PGP9.5 (green).<br>Goat anti-mouse IgG, Alexa Fluor 594 (Invitrogen A11005; 1:1000) — used for GAP-43 (red).<br>Goat anti-rabbit IgG, Alexa Fluor 594 (Invitrogen, [catalog #]; 1:800) — used for c-Fos (red) and other rabbit primaries as applicable.
Validation	All antibodies were commercially sourced and selected based on manufacturer validation and prior use in the literature. Specificity was assessed by expected tissue localization and negative controls (omission of primary antibody and/or secondary-only controls). Imaging and quantification were performed using standardized acquisition and analysis settings within each staining batch.

Eukaryotic cell lines

Policy information about cell lines and Sex and Gender in Research

Cell line source(s)	Simian Virus 40–transformed human uroepithelial cell line (SV-HUC-1) was obtained from ATCC (USA) and cultured at 37 °C with 5% CO_2 in high-glucose DMEM (Gibco) supplemented with 10% FBS and 1% penicillin/streptomycin. Sex of the cell line donor was not specified by the vendor and was not independently determined.
Authentication	Cells were used as provided by ATCC; no additional authentication (e.g., STR profiling) was performed. Cells were used within ≤10 passages after thawing.
Mycoplasma contamination	Mycoplasma testing was not performed.
Commonly misidentified lines<br>(See ICLAC register)	None.

Animals and other research organisms

Policy information about studies involving animals; ARRIVE guidelines recommended for reporting animal research, and Sex and Gender in Research

Laboratory animals	Specific-pathogen-free (SPF) Sprague–Dawley rats (♀24, ♂24; age 8–10 weeks; 250–400 g) were used. Animals were procured from Changzhou Cavins Laboratory Animal Co., LTD.
Wild animals	This study did not involve wild animals.
Reporting on sex	Both female and male rats were included (♀24, ♂24) and sex was recorded as part of the experimental design.
Field-collected samples	This study did not involve samples collected from the field.
Ethics oversight	All animal experiments were approved by the Animal Ethics Committee of Ruijin Hospital at School of Medicine, Shanghai Jiao Tong University (Ethics/IACUC No. RJ2024051).

Note that full information on the approval of the study protocol must also be provided in the manuscript.

Plants

Seed stocks	N/A
Novel plant genotypes	N/A
Authentication	N/A
