# Supplementary material for: Wireless electrostimulation implants enable sphincter neuromuscular improvement toward mixed urinary incontinence
Source: Nat Commun. 2026 Apr 15;17:5226. doi: 10.1038/s41467-026-71532-7 (PMC13260462; doi:10.1038/s41467-026-71532-7)
Supplement: Supplementary file 3 — Transparent Peer Review file [file 41467_2026_71532_MOESM3_ESM.pdf]

nature portfolio

Peer Review File

Wireless electrostimulation implants enable sphincter neuromuscular improvement toward mixed urinary incontinence

Corresponding Author: Professor Metin Sitti

This file contains all reviewer reports in order by version, followed by all author rebuttals in order by version.

Version 0:

Reviewer comments:

Reviewer #1

(Remarks to the Author)
This manuscript presents a low-cost, soft, and wireless-powered electrostimulation patch for the treatment of urinary incontinence, demonstrating a high efficacy rate of 81%. In vivo experiments reveal that on-site stimulation of the urethral sphincters in mice effectively reduces urination frequency while increasing urinary volume. Furthermore, the device exhibits excellent biocompatibility during long-term implantation. To further enhance the quality and impact of the manuscript, additional experiments and discussions are recommended to sufficiently substantiate the claims made, as listed below:

1. The electrodes were wrapped around the urethral sphincter to deliver electricity, raising concerns about how to maintain a stable interface and ensure low resistance contact between the wires and the wet tissue. It is recommended to measure the impedance between the electrode and the urethral sphincter during long-term implant. Additionally, while the manuscript presents X-ray images of the electrodes, optical photographs of the electrodes in their wrapped configuration would be more intuitive and visually informative.
2. During device implantation, the electrodes were fixed by applying appropriate tension. It is important to discuss whether this force could potentially affect the function of the urethral sphincter.
3. In the manuscript, the Tx coil is powered by an external power supply. Additional details about the input voltage of the power supply would be helpful. Incorporating a battery to power the circuit could significantly enhance the portability of the device. Without this modification, the application scenarios may remain restricted to locations near a power source.
4. Using a battery to directly power the device, combined with a coil for charging the battery, could enhance energy transfer efficiency and improve wearing comfort for users. It is recommended to provide a more detailed discussion on the advantages of current design.
5. For the in vivo experiments, it is suggested to include a group with traditional treatment in Fig. 4f to allow for a direct comparison of the efficacy between the proposed method and current clinical therapies.

Reviewer #2

(Remarks to the Author)
The manuscript presented by Sitti et al. introduces WIPES, a wireless implantable platform for electrostimulation, offering a compelling solution for urinary incontinence treatment. The study showcases the device's efficient electrostimulation at the urethral sphincter, supported by in vivo experiments in rats, and emphasizes its biosafety and biocompatibility. While the work is innovative and timely, several aspects could benefit from further clarification and elaboration. A minor revision is recommended to enhance the scientific rigor and clarity of the manuscript prior to publication.
1. The manuscript briefly mentions both direct electrostimulation (via WIPES) and indirect electrostimulation, but a more detailed comparison between the two methods is lacking. While direct electrostimulation is emphasized for its efficiency, indirect stimulation offers benefits such as stability and ease of implantation. A more thorough discussion on the advantages and limitations of both methods would strengthen the manuscript, particularly in terms of long-term safety, mechanical stability, and performance comparison with existing commercial devices. Additionally, further analysis of the mechanical properties and long-term biocompatibility of WIPES would enhance the overall scientific rigor.
2. The WIPES device comprises a six-layer structure including metallic circuits and multiple coils. This multilayered

architecture may be susceptible to mechanical degradation, particularly under repetitive tensile or flexural stress. Long-term mechanical reliability, including risks such as interlayer delamination, should be addressed. Additional mechanical characterizations—such as cyclic tensile testing or fatigue analysis—would substantiate the device's durability and its compatibility with dynamic biological tissues.
3. The manuscript defines the activation power threshold as 0.15 W, highlighting stable operation across various coil alignments and distances. However, the rationale behind selecting this specific threshold is not clearly articulated. Including experimental or theoretical support—e.g., safety margins for tissue heating, power efficiency analyses, or threshold screening—would strengthen the interpretation of results shown in Figures 2c–e.
4. The manuscript shows that the gold electrostimulator emerges directly from the WIPES device without any encapsulating layer. This unsealed interface could permit extracellular matrix (ECM) infiltration through the interface gap, potentially causing device malfunction during long-term implantation. It is recommended that the authors discuss strategies to address this risk, such as soft encapsulation layers or self-healing materials, and provide evidence supporting the device's functional stability under chronic in vivo conditions.
5. The transmitter (TX) coil used in this study appears to be approximately 20 cm in diameter, which may pose limitations for practical use. If such a large TX coil is required for effective power delivery, its real-world applicability could be restricted. The authors should discuss how the device performance varies with TX coil dimensions and whether miniaturization could compromise efficiency. Experimental data or modeling results examining TX size-dependent performance would be a valuable addition.

Reviewer #3

(Remarks to the Author)
The manuscript by Zheng et al describes an implantable wireless neuromuscular stimulator to interface with the urethra and reduce symptoms of urinary incontinence. The fabricated device is novel, and its flexibility is an improvement to other wireless stimulators available.

Major comments
1. From the introduction, authors define MUI as the target for their stimulation therapy; however, the animal model with injection of CTX is not adequate to test their hypothesis. First, it is unclear why authors hypothesize that stimulating the smooth muscles of the urethral sphincter would be beneficial to treat the urge component of UUI and MUI. If the goal is for authors to stimulate the sphincters, then this is a treatment for SUI only, without addressing the urge components.
2. Furthermore, the tests conducted (urine sand mass counting & weighting) are not adequate to represent an effect on MUI (nor UUI/SUI by themselves). One of the major issues with the conducted test is that the bladder fill is unknown at the time of involuntary leak, which makes it impossible to characterize the incontinence phenotype and thus its rehabilitation. Authors need to conduct an urodynamics test and leak-point-pressure test (at least at the terminal point) to validate the effect of urethral closure via electrical stimulation of the sphincters.
3. An additional concern is that the sphincter closure via electrical stimulation may improve continence, but it does not do anything for bladder overactivity. Meaning that, while potential therapy can lead to continence, the urge component will not be addressed.
4. It is clear that the therapy is leading to a reduced frequency of leaks in experimental animals. However, there is no proposed mechanism of action nor its validation with the data collected. Because of this, it is hard to interpret the results in relationship to the phenotype of urinary incontinence.
5. Cyclophosphamide induces cystitis, which can mimic the symptoms of bladder overactivity; however, its injection is not appropriate animal model for the complexities of urge urinary incontinence (which include additional symptoms to bladder overactivity), and certainly not appropriate for mixed urinary incontinence. As such, the description in lines 229-231 is not accurate, and authors do not provide sufficient and relevant references to defend the model used.
6. The urethral pressure reported ex-vivo showed a maximum average of ~40 Pa (0.30 mmHg); however, this value is not sufficient to achieve continence during an overactive bladder contraction. Albeit this value is in the ex-vivo rat, it was not measured in-vivo and thus, it is hard to tell how much it would change the closure pressure. Furthermore, it is unclear how the pressure relates directly to the therapy here, since the mechanism of action appears to be more rehabilitative (muscle-to-collagen ratio improvement) than direct modulatory (closure of sphincters upon bladder contraction).
7. In lines 344-346 authors state that their results indicate modulation of neural signaling, promotion of muscle and nerve cell regrowth, but their results do not directly confirm these hypotheses. For example, terminal electrophysiology and/or urodynamics would be needed to confirm the modulation of neural signals, and nerve cell regrowth would require additional histological evaluation of nerve sections showing such regrowth.
8. While the device has a drastically reduced size, it is not the smallest wireless stimulation device available in the context of urinary incontinence, which needs to be acknowledged and discussed (see: doi.org/10.1016/j.ucl.2004.09.003, doi.org/10.1038/s41598-021-90088-8, doi.org/10.1002/nau.25477, doi.org/10.1007/s11255-024-04318-9, etc.). Overall, the discussion is incomplete, and the authors should include comparisons to other wireless stimulators reported in literature, and stronger evidence demonstrating a strong correlation between MUI and urethral incompetence. Furthermore, authors should state how their device would be superior to other non-invasive devices that stimulate the pelvic floor muscles and/or sphincter to reduce leaks.

Minor comments
1. The manuscript is overall well written with minor instances where sentences are informal, and the authors should revise the manuscript to improve readability.
2. The definitions of SUI and UUI are not entirely accurate. Please revise.
3. Lines 60-61: Why did authors decide to cite an abstract (as opposed to a peer-reviewed article) that included patients with

fecal incontinence? There is plenty of literature on the therapeutic effectiveness of SNM. Furthermore, it is unclear where 48% effectiveness is reported in the cited reference.
4. Lines 67-69: List of diagnostic steps should include urodynamic tests as well.
5. References should be reviewed, as some have nothing to do with the sentences stated.
6. In line 111, authors state that the wires are conductive only where it contacts the urethral sphincter, but it is unclear how the sizing is achieved.
7. For figure 2h, please provide the methodology of how these measurements were obtained? Was this measured in saline? What is simulated with a resistor?
8. In panel a of Figure 3, device placement into culture is named "implantation" but this term is incorrect.

Reviewer #4

(Remarks to the Author)
General comments:
The study looked at electro stimulation to rat's urinary sphincter to see if it is good as a novel tool to treat urinary incontinence induced by CTX.
The hypothesis may be wrong. Neuromodulation is about stimulation of the other peripheral nerves to modulate neurological performance of specific function, not direct stimulation on the specific muscles responsible for function.
The study method did not describe details in identifying sphincter, responses of urinary bladder. Increase of sphincter function will have effects on detrusor. Changes of CTX cystitis is also important for readers and should be reported.
Insertion of a catheter into male rats' urethra is difficult and painful. How did the author overcome this issue should be reported and animal research committee should provide agreement on this issue.

Specific comments

Line 51. UUI is also common in women.
Line 81. direct stimulation at the nerves and smooth muscles of the urethral sphincter
The urethral sphincter is a muscular structure that regulates the flow of urine from the bladder. It consists of two sphincters: the internal urethral sphincter and the external urethral sphincter. The internal sphincter is made of smooth muscle and is under involuntary control, while the external sphincter is made of skeletal muscle and is under voluntary control. Please correct it and specify WIPES stimulate internal or external sphincter.
CTX related incontinence is not related to SUI because we did not change pelvic floor and urethral function. Otherwise, the authors should provide reference for this statement.
Methods:
Regarding fig S9
Q: Chamber pressure is typically measured using a balloon infused with saline, and compared to the balloon, the compressive force of a silicone tube is relatively lower and does not contract to generate pressure. Additionally, the pressure sensor used by the author is not a conventional physiological method.

Regarding F4.
263 (f) Number of urinary sand masses indicates urination frequency. Each dot represents one measurement while each column represents the mean and the error bars indicate +/- one standard deviation. Statistics of Incontinence modelling and UI & ES groups for females show *P < 0.05 with N = 4 rats (data for male showing statistically not significant with N = 4 rats).
Q. The author states that electrical stimulation was performed through two flexible wires fixed on the urethral sphincter, with the urethral sphincter being the primary stimulation site. Figure 4f shows that the effect of ES is significantly better in females than in males. Since the female urethra lacks a distinct striated muscle structure, how does the author ensure that the electrodes do not pierce the urethra?
Q. The standard deviation in Figures 4f and 4g is excessively large, and with a sample size of n=4, it is challenging to robustly demonstrate a statistically significant difference..
Q. Please provide the body weight of the rats.

51 On the other hand, UUI occurs more frequently in men and is more complex to tackle.
Q. The author originally aimed to improve urinary incontinence in males, but the data in this article show that WIPES is more effective for cyclophosphamide-induced urinary incontinence in female animals, which the author did not explain.
228 The incontinence model was induced via intraperitoneal injection of cyclophosphamide (CTX) at a dose of 80 mg/kg [38],
Q. The reference 38, Jerkins et al., 1988, cited by the author does not specify the dosage of cyclophosphamide (CTX) used, and the 80 mg/kg dose is lower than the 100~150 mg/kg commonly used in literature.

Q. The author performed electrical stimulation on the urethra, but the use of cyclophosphamide also induced cystitis. The author should include relevant urinary bladder tissue sections in Figure 6 to provide supporting evidence.
Q. The author inserted a PE tube through the urethral opening and penetrated the urinary bladder, causing not only extreme discomfort but also urethral injury. Therefore, the author should provide complete urethral tissue sections to support their findings.

Regarding On demand activation. Who controlled the WIPES? Rats? Researchers? What is the frequency or duration of "relax" in this study?
Since this study enhanced the sphincter tone, were there any change of detrusor muscle? Any change of kidney? Urinary

tract infection? All these potential complications of high urinary sphincter tone should be mentioned.
What were the changes of cystometric capacity and detrusor pressure in these rats?
After long term implantation of WIPES, were there any ischemic change of sphincter? Fibrosis?
Molecular study compared key molecules in smooth muscles. However, urinary sphincter mainly composes of skeletal muscles. The authors showed that the changes in smooth muscle were clear. How about skeletal muscle and its related nerves?

Line 346. There was no evidence that SUI in these rats improved. Assessment of SUI in rats was not performed.
Line 347. Similarly, the authors could not say this device is good for mixed UI.

Version 1:

Reviewer comments:

Reviewer #1

(Remarks to the Author)
All my comments are well addressed. It can be published in current state.

Reviewer #2

(Remarks to the Author)
The authors have fully addressed all comments that we raised before.
Therefore, the revised manuscript can be published as is in the Nature Communications journal.

Reviewer #3

(Remarks to the Author)
I would like to thank the authors for addressing the comments and revising the document accordingly.
1. Authors state that the hypothesized mechanism of action on the bladder to addresses the urge component of MUI, is via indirect activation of sacral circuitry. However, the only supporting data is via post-mortem analysis of cFOS of the periaqueductal grey. While this area is indeed relevant for micturition, cFOS at this level does not provide any information regarding sacral circuitry or activation. In addition, this analysis does not provide mechanistic insight, rather an observational study. To confirm the indirect activation of sacral circuitry, would need to add additional electrophysiological experiments that confirm the engagement of spinal circuitry for bladder.
a. The new line "Mechanistically, the therapeutic benefit reflects modulation of peri-urethral neural signaling and regeneration of the sphincter musculature, addressing a previously unfilled restoration pathway in rat incontinence models." reads as a conclusion, but without supporting data, this sentence is only a non-tested hypothesis.
2. While authors have addressed the choice of model for MUI by separating the symptoms and addressing them via their stimulation, the writing is still misleading. The suggestion is that authors change the title and content of the article to clearly state that each symptom is addressed individually and working towards a device for MUI, but their model does not have MUI.
3. Authors have added cystometry studies to the article; unfortunately, the anesthesia selection was not appropriate. Multiple investigations have revealed that isoflurane is a poor choice of anesthetic as it suppreses the micturition cycle, affects the EMG of the EUS, and reduces LPP (10.1080/21681805.2021.1960598, 10.1152/ajprenal.90259.2008) and urethane is the preferred method for cystometry in rats (10.1016/S0024-3205(01)01182-1). This is further evidenced by the cystometry traces presented in Fig 5, where a voiding contraction is observed approximately every 2.5 minutes; yielding a bladder volume of approximately 0.2 to 0.25 mL, where the bladder capacities should be closer to 1.5 mL.
a. As a result, the line that reads "These findings collectively confirm that WIPES stimulation not only enhances urethral closure pressure mechanically, as evidenced by LPP recovery, but also re-establishes physiological neuromuscular coordination essential for continence regulation." is impossible to assert with the current data because isoflurane interferes with EUS EMG, thus it is not possible to assess changes driven by WIPES. Furthermore, the EMG traces should be presented synchronous to Cystometry traces to assess the EMG re-synchronization.
b. Fig. 5a UUI modeling confirms non-voiding contractions, consistent with overactive bladder; however, it is not clear which trace is supposed to represent the SUI model.
c. Fig. 6a now shows evidence that LPP increases with ES when compared to SUI, which is consistent with a successful therapy for SUI; however, interpretation of results should be restated because of the selection of anesthetic.
4. Reviewer 3, comment 8 - Authors have replied with highlighted differences with prior state of the art technology, but have not incorporated these changes to the discussion in the manuscript. These changes should be incorporated as they put their new technology into perspective.

Reviewer #4

(Remarks to the Author)
The article is markedly improved.
I still have some concerns:

1. In the three groups involving electrostimulation, Normal body ES (3rd), UUI & WIPES ES (5th) and SUI & WIPES ES (7th), rats were subjected to three 30-minute daily stimulation sessions, with each consisting of three cycles of 30 seconds of stimulation followed by a 9.5-minute rest interval.
Since on demand application of the device, we do not know the rats' demand. How to identify this needs? Were the rats agitated or any behavioral change during the study?
Actually, the study is not on rats' demand. It is a timed stimulation model. Please clarify it in the Title.
2. Any treatment to enhance sphincter function or increase bladder outlet resistance may have detrusor damage in the long term. Please discuss it or provide some evidence that this device is fine in long ter use.

Open Access This Peer Review File is licensed under a Creative Commons Attribution 4.0 International License, which permits use, sharing, adaptation, distribution and reproduction in any medium or format, as long as you give appropriate credit to the original author(s) and the source, provide a link to the Creative Commons license, and indicate if changes were made.
In cases where reviewers are anonymous, credit should be given to 'Anonymous Referee' and the source.
The images or other third party material in this Peer Review File are included in the article's Creative Commons license, unless indicated otherwise in a credit line to the material. If material is not included in the article's Creative Commons license and your intended use is not permitted by statutory regulation or exceeds the permitted use, you will need to obtain permission directly from the copyright holder.
To view a copy of this license, visit https://creativecommons.org/licenses/by/4.0/

Response to the Reviewers' Comments

We thank the reviewers for the constructive feedback on our paper. We have substantially modified the manuscript based on this feedback and believe that the new version is significantly improved. A point-by-point description of the changes is provided below, where our responses to the reviewer feedback are in blue. Meanwhile, red marks indicate changes from previous submissions.

Below we provide a page index to facilitate navigation of the detailed responses to each reviewer.

Reviewer 1: Pages 2–9.

Reviewer 2: Pages 10–16.

Reviewer 3: Pages 17–37.

Reviewer 4: Pages 38–64.

Reviewer 1:

This manuscript presents a low-cost, soft, and wireless-powered electrostimulation patch for the treatment of urinary incontinence, demonstrating a high efficacy rate of 81%. In vivo experiments reveal that on-site stimulation of the urethral sphincters in mice effectively reduces urination frequency while increasing urinary volume. Furthermore, the device exhibits excellent biocompatibility during long-term implantation. To further enhance the quality and impact of the manuscript, additional experiments and discussions are recommended to sufficiently substantiate the claims made, as listed below:

We sincerely thank the reviewer for the encouraging evaluation and constructive suggestions. In the revised manuscript, we have incorporated additional experiments, analyses, and discussions to further substantiate our conclusions and to highlight the advantages of our WIPES system.

1. The electrodes were wrapped around the urethral sphincter to deliver electricity, raising concerns about how to maintain a stable interface and ensure low resistance contact between the wires and the wet tissue. It is recommended to measure the impedance between the electrode and the urethral sphincter during long-term implant. Additionally, while the manuscript presents X-ray images of the electrodes, optical photographs of the electrodes in their wrapped configuration would be more intuitive and visually informative.

We appreciate the reviewer's insightful comments regarding electrode–tissue coupling stability. To address the concern regarding electrode–tissue coupling stability, we have conducted electrochemical impedance spectroscopy (EIS) to evaluate the long-term interface properties of the WIPES electrodes during implantation (refer to the section of 'Electrochemical impedance spectroscopy (EIS) of WIPES electrodes' in the Methods and Fig. S21). The results confirm stable impedance characteristics, indicating robust and low-resistance electrode-tissue coupling throughout the chronic implantation period.

In addition, as recommended, we have added optical photographs of the gold electrode wrapped around the urethral sphincter (now shown in Fig. S27), which more intuitively illustrates the implanted electrode configuration alongside the previously presented X-ray images (Fig. S7).

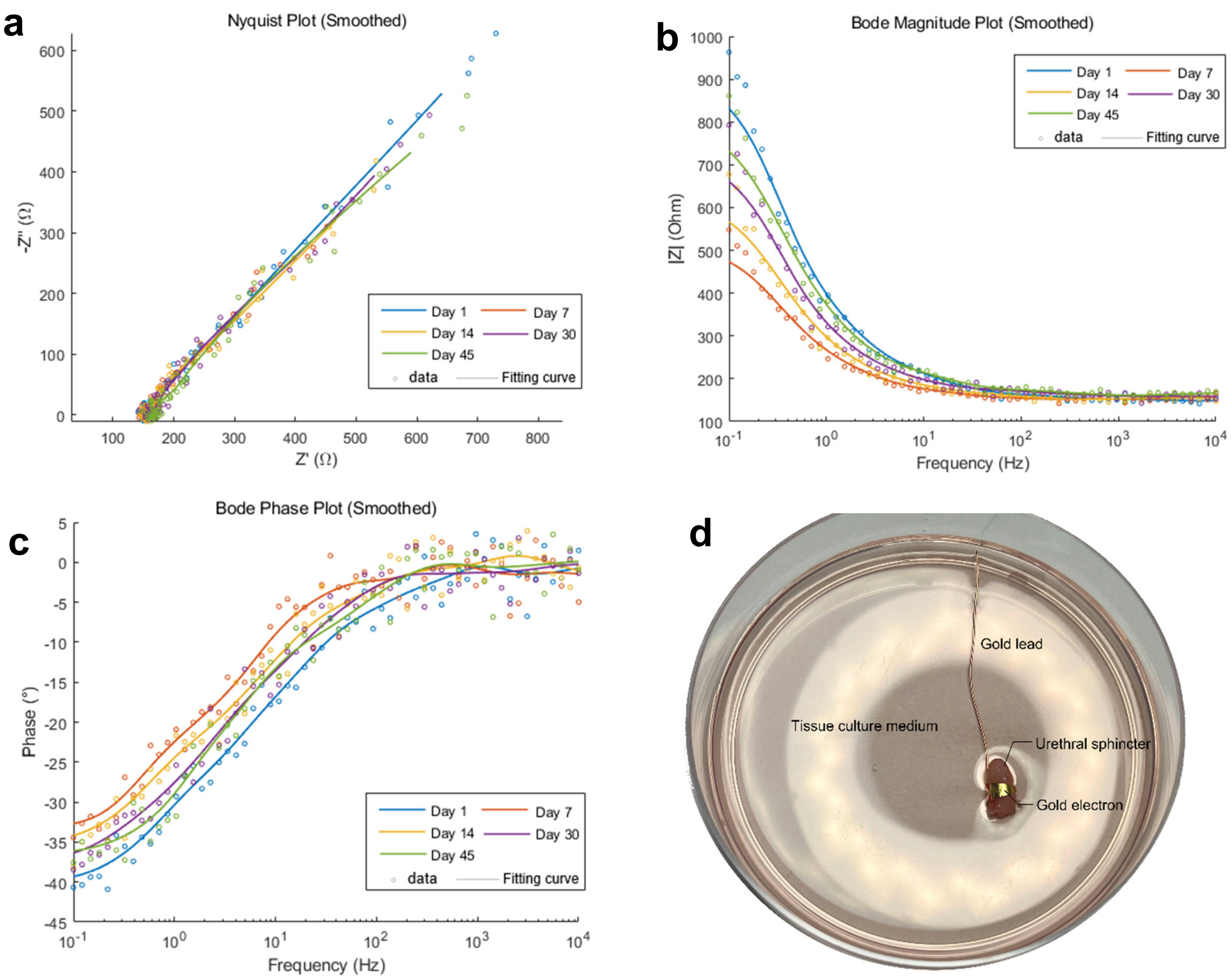

Fig. S21. Electrochemical impedance spectroscopy (EIS) of WIPES electrodes. (a–c) EIS measurements at Days 1, 7, 14, 30, and 45 post-implantation in PBS. The Nyquist, Bode magnitude, and phase plots. (d) Ex vivo image of a rat urethral sphincter wrapped by the gold electrode.

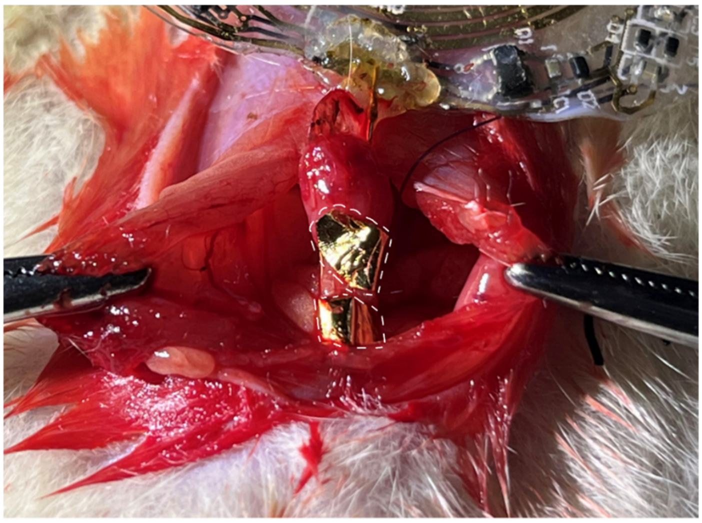

Fig. S27. Intraoperative view of WIPES electrode wrapping the external urethral sphincter (EUS).

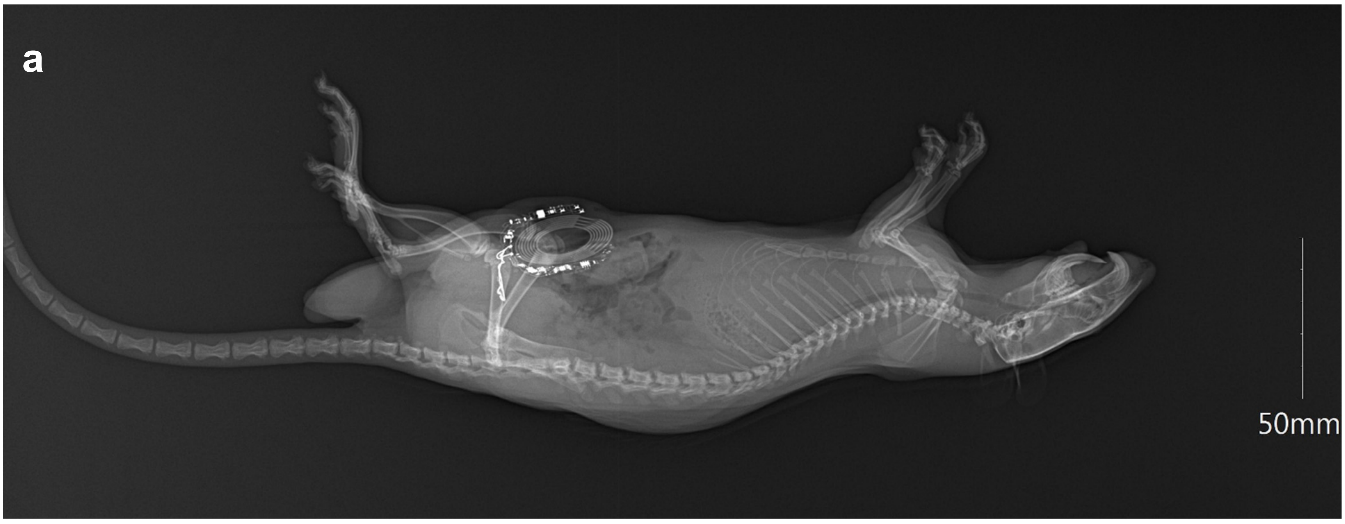

Fig. S7. Medical images of the WIPES device, composed of Rx coils with different turns, implanted in rats, with showing X-ray images.

==

Location of Change: Line 190, Section 'Electrochemical Impedance Spectroscopy (EIS) of WIPES Electrodes' in the Methods and Fig. S26

Original Sentences: /

Revised Sentences: Furthermore, electrochemical impedance spectroscopy (EIS) of the WIPES electrodes demonstrated stable interfacial impedance for at least 45 days under physiological conditions, confirming reliable and low-resistance electrode–tissue coupling during long-term operation (refer to the section of 'Electrochemical impedance spectroscopy (EIS) of WIPES electrodes' in the Methods and Fig. S21).

Location of Change: Line 115

Original Sentences: The wires are specially designed to wrap around the sphincter with curved patches ensuring stability while avoiding squeezing on it.

Revised Sentences: The electrodes are designed as flexible C-shaped gold patches that wrap gently around the sphincter, ensuring stable contact without exerting constrictive pressure (Fig. S27). The inner surface serves as the active interface, while the outer surface and connecting leads are fully encapsulated to maintain insulation.

==

2. During device implantation, the electrodes were fixed by applying appropriate tension. It is important to discuss whether this force could potentially affect the function of the urethral sphincter.

We appreciate the reviewer's concern. We would like to clarify that the WIPES electrodes were not applied as constrictive wires or tightly bound coils. Instead, the gold electrode was fabricated as a thin and flexible patch that was gently wrapped around the urethral sphincter and fixed by natural surface adhesion with minimal tension (Fig. S27). This configuration provided stable attachment and intimate contact with the tissue while imposing only a weak physical constraint rather than compressive force. Therefore, the implantation procedure did not exert significant mechanical pressure or constriction on the sphincter, and no adverse effects on sphincter function were observed in either acute or chronic studies.

3. In the manuscript, the Tx coil is powered by an external power supply. Additional details about the input voltage of the power supply would be helpful. Incorporating a battery to power the circuit could significantly enhance the portability of the device. Without this modification, the application scenarios may remain

restricted to locations near a power source.

We thank the reviewer for this valuable suggestion. In our current setup, the Tx coil is powered by a regulated DC supply (20–30 V) to ensure stable and reproducible experimental conditions. Additionally, the circuit design is fully compatible with portable power sources, and it can be readily driven by a battery pack to achieve stable wireless energy transfer and electrostimulation. This configuration allows flexible deployment of the system in daily environments, consistent with the portable application scenario illustrated in Fig. 1b.

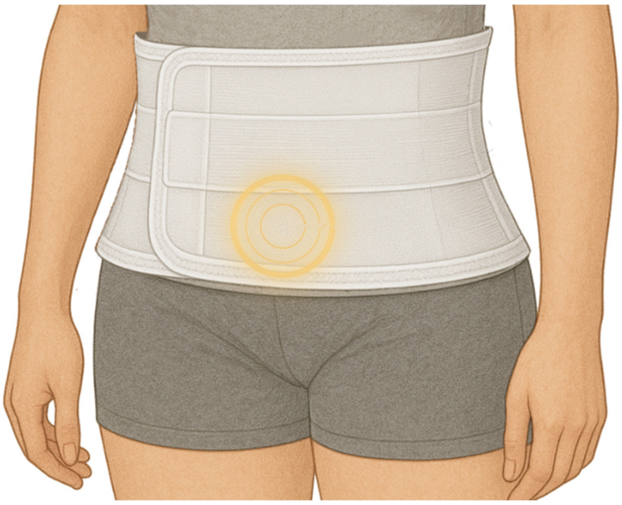

Fig. 1b Wearable transmitting belt enabling wireless power transfer to the implanted WIPES device.

==

Location of Change: Line 110

Original Sentences: Patient could receive electrostimulation treatment at customized frequencies and voltages for a designed period in a clinic or at home.

Revised Sentences: Patient could receive electrostimulation treatment at customized frequencies and voltages for a designed period in a clinic or at home using the belt-integrated Tx coil (Fig. 1b), which is worn around the waist to provide portable, continuous power to WIPES.

==

4. Using a battery to directly power the device, combined with a coil for charging the battery, could enhance energy transfer efficiency and improve wearing comfort for users. It is recommended to provide a more detailed discussion on the advantages of current design.

We appreciate the reviewer's constructive suggestion. As shown in Fig. 1b, the Tx coil is fabricated in a flexible form and integrated into a belt that can be comfortably worn around the waist for daily use. In this configuration, the Tx can continuously deliver power to the implanted WIPES during operation, ensuring stable stimulation. The belt-like structure offers good portability and comfort, enabling home-based treatment without dependence on stationary power sources.

5. For the in vivo experiments, it is suggested to include a group with traditional treatment in Fig. 4f to allow for a direct comparison of the efficacy between the proposed method and current clinical therapies.

We thank the reviewer for this important suggestion. Sacral nerve stimulation (SNS) is indeed a widely adopted traditional clinical treatment for urge urinary incontinence (UUI), typically applied as a neuromodulation therapy to modulate detrusor overactivity via sacral nerve regulation. To provide a direct comparison with current therapy, we performed needle-based sacral nerve stimulation in rats following established procedures (refer to the section of 'Spinal Nerve Stimulation for UUI Treatment' in the Methods). Briefly, acupuncture needles were inserted at the bilateral posterior superior iliac spine (approximating the

S1-S2 level), and stimulation was delivered using monophasic square pulses (60 Hz, 0.3 ms, 4 V, 30 min/day for 7 days) under light anesthesia. The needle insertion procedure is shown in Fig. S24a, and the anatomical positioning was confirmed by lateral X-ray imaging, as illustrated in Fig. S25b.

The results, shown in Fig. S26b and Fig. S26c, indicate that SNS significantly improved both single urination volume and urination frequency in UUI animals of both sexes compared with untreated models (**, ***), although the efficacy was still lower than that achieved with WIPES electrostimulation. In SUI models, the outcomes differed by sex: in males, SNS showed no statistical significance (ns) in either urination volume or frequency, while in females, SNS showed a trend toward improvement in both parameters, but the effect did not reach statistical significance (ns). This observation may be related to anatomical and hormonal differences in the female pelvic floor, which make the sphincter more responsive to sacral neuromodulation, albeit insufficient to reach robust therapeutic levels. Across all groups, WIPES electrostimulation consistently produced the most pronounced recovery (***), underscoring the superior efficacy of localized urethral sphincter stimulation.

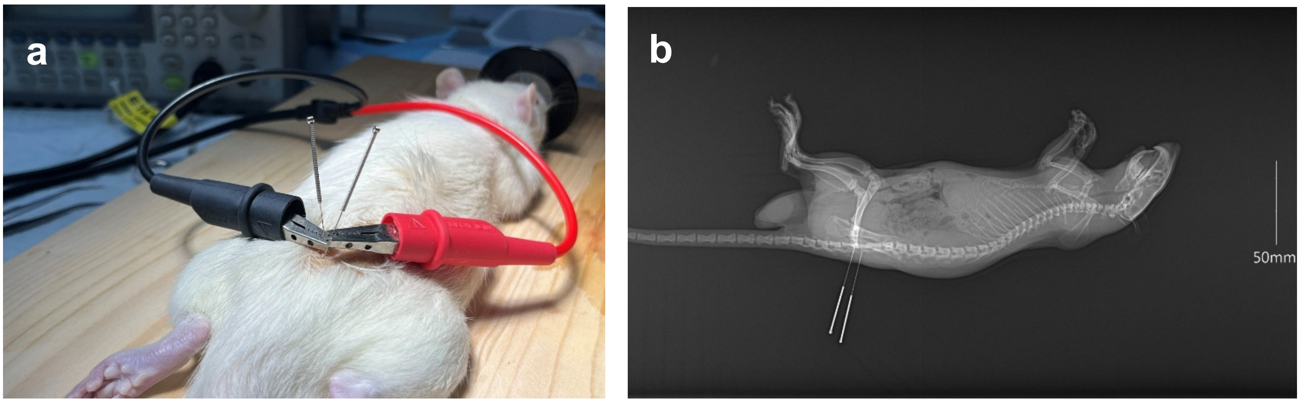

Fig. S25. Simulation of sacral nerve stimulation (SNS) in rats. a. Experimental setup showing transcutaneous sacral nerve stimulation via bilateral needle insertion and external pulse connection. b. X-ray image confirming needle positions

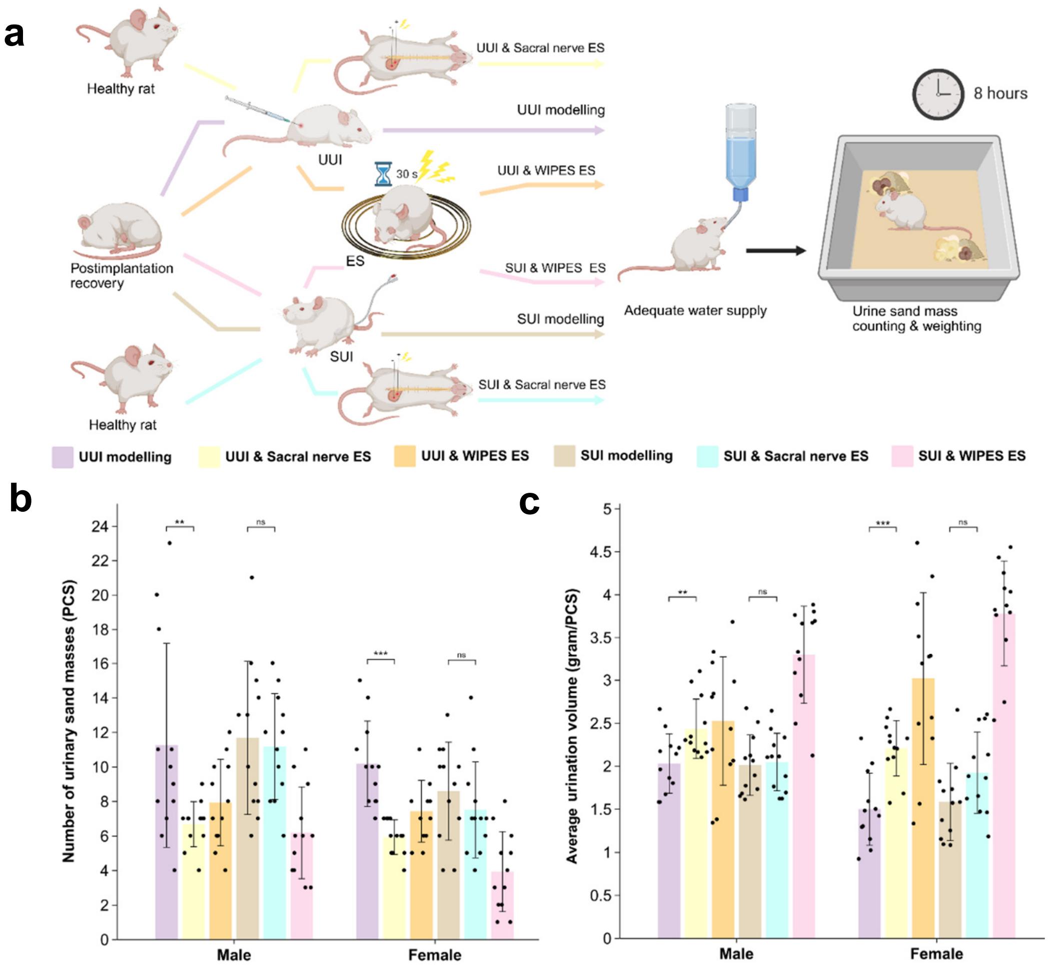

Fig. S26. Comparison with WIPES and traditional SNS for UUI or SUI. a. Workflow. b. Number of urinary sand masses. c. Average urination volume.

==

Location of Change: Section 'Spinal Nerve Stimulation for UUI Treatment' in the Methods and Fig. S25, S26 and Fig. 5.

Original Sentences: /

Revised Sentences: A new subsection has been added in the Methods to describe the procedures for Spinal Nerve Stimulation (SNS) for UUI Treatment, including electrode placement, stimulation parameters, and treatment duration for comparison with WIPES therapy. The experimental setup and anatomical positioning are shown in Fig. S25, while corresponding results and comparisons with WIPES are presented in Figs. S26.

==

Further comparative analysis with the main text results reveals that the moderate behavioral improvement observed with SNS corresponds to only partial recovery in urodynamic parameters (Fig. 5a). Specifically, cystometric measurements showed that SNS reduced the frequency of non-voiding contractions (NVCs) and modestly increased bladder capacity relative to the UUI modelling group. However, these effects remained inferior to those achieved under WIPES stimulation, which more effectively restored near-normal detrusor activity. In terms of central neuronal activation (Fig. 5b and 5c), c-Fos immunostaining in the periaqueductal gray (PAG) and pontine micturition center (PMC) revealed comparable or slightly higher activation levels between SNS and WIPES, suggesting that both approaches can engage supraspinal

control pathways, though without a distinct advantage for either. At the peripheral level, PGP9.5 and GAP-43 staining demonstrated that SNS promoted partial nerve regeneration within the urethral sphincter region (Fig. 5d and 5e), indicative of ongoing neural remodeling, but the regenerated fibers exhibited lower density and maturity compared with those induced by WIPES stimulation. Moreover, SNS resulted in a significant decrease in nNOS expression compared with the UUI modeling group, indicating partial inhibition of nitrergic signaling (Fig. 5f and 5g); however, the reduction was limited relative to that achieved by WIPES stimulation. In contrast, VIP levels remained elevated after SNS treatment, likely due to its inability to resolve local inflammatory responses, whereas WIPES effectively suppressed VIP overexpression (Fig. 5h), reflecting mitigation of inflammation and restoration of balanced peptidergic signaling. Collectively, these findings indicate that while SNS achieves moderate improvements in both bladder dynamics and peripheral nerve remodeling, the localized and sustained stimulation provided by WIPES offers more robust restoration of neuromuscular function.

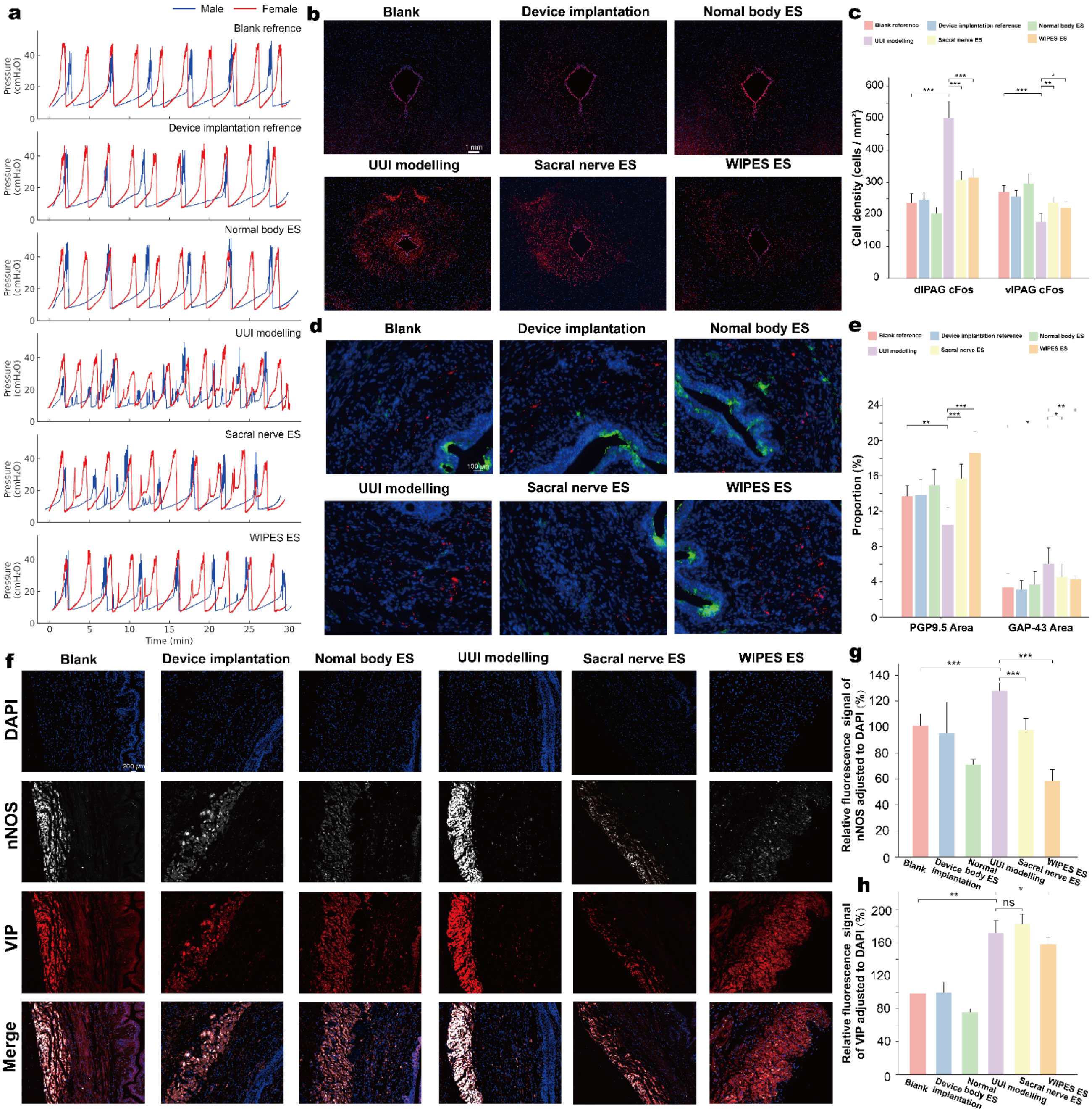

Fig. 5. WIPES restores bladder function and promotes neural regeneration in rats with urge urinary

incontinence. (a) Representative cystometric traces of male and female rats under different conditions. (b) c-Fos immunostaining images of the dorsal and ventrolateral periaqueductal gray (dPAG and vlPAG) regions in rats from each group. (c) Statistical analysis of c-Fos–positive cell densities in dPAG and vlPAG regions. (d) Immunofluorescence staining images showing PGP9.5 and GAP-43 expression in urethral sphincter tissues of rats in each group, the green fluorescence indicates the expression of PGP9.5, and red fluorescence indicates the expression of GAP-43. (e) Statistical analysis of PGP9.5-positive area and GAP-43 index in rats of all groups. (f) Immunofluorescence staining of nNOS and VIP neurotransmitters in urethral sphincter tissues of rats in each group. (g) Quantitative comparison of nNOS fluorescence intensity normalized to DAPI for all groups. (h) Quantitative comparison of VIP fluorescence intensity normalized to DAPI for all groups.

==

Location of Change: Line 318

Original Sentences: /

Revised Sentences: The sacral nerve stimulation (SNS) group also showed moderate improvement but failed to achieve full recovery, with persistent irregular NVCs and shortened ICIs. These results demonstrate that WIPES achieves superior neuromuscular interface precision and dynamic control of the detrusor–sphincter reflex loop compared to conventional SNS therapy, likely due to its localized stimulation and closed-field coupling characteristics.

Similarly, SNS ES group (5th) produced comparable effects, with dlPAG activity reduced by 37.16% and vlPAG elevated by 33.77%. These results suggest that both WIPES and SNS treatments effectively rebalanced the dlPAG–vlPAG circuitry, and from the perspective of impulse inhibition, their restorative efficacy showed no significant difference.

Following treatment, the SNS ES group (5th) exhibited a 43.97% increase relative to the model group, reflecting partial recovery characterized by discontinuous and loosely organized fibers. In contrast, the WIPES ES group (6th) demonstrated a 47.70% increase in PGP9.5 area, showing densely packed and continuous neural structures, indicative of robust peripheral nerve restoration. In contrast, SNS ES group (5th) reduced the GAP-43 area by 25.06%, and WIPES ES group (6th) by 28.99%, relative to the UUI model, reflecting suppression of excessive, non-functional axonal sprouting. Notably, WIPES treatment normalized the GAP-43 area to a moderate yet orderly level while significantly increasing the PGP9.5 area, suggesting that WIPES promoted structured axonal regeneration and functional reinnervation rather than chaotic overgrowth.

Following SNS ES (5th) and WIPES ES (6th) electrostimulations, nNOS signals recovered toward physiologically balanced patterns, with a decrease of 23.67% and 54.30%, VIP levels have different expressions, with increase a little (Mean ± SD for N = 8 rats; ns, not significant) and a decrease of 8.03%. The normalized nNOS distribution implies restored nitric oxide–mediated inhibitory tone, preventing premature detrusor contractions, while the orderly re-expression of VIP indicates re-established neurovascular coupling and mucosal relaxation signaling. These features collectively reflect the functional reconnection of afferent–efferent feedback loops rebuilt through WIPES-induced peripheral reinnervation. By contrast, SNS treatment led to partial normalization, with residual nNOS hyperexpression and sparse VIP regions, suggesting incomplete restoration of inhibitory control.

==

Reviewer 2:

The manuscript presented by Sitti et al. introduces WIPES, a wireless implantable platform for electrostimulation, offering a compelling solution for urinary incontinence treatment. The study showcases the device's efficient electrostimulation at the urethral sphincter, supported by in vivo experiments in rats, and emphasizes its biosafety and biocompatibility. While the work is innovative and timely, several aspects could benefit from further clarification and elaboration. A minor revision is recommended to enhance the scientific rigor and clarity of the manuscript prior to publication.

We greatly appreciate the reviewer's thoughtful assessment and constructive feedback. In response, we have revised the manuscript to provide clearer explanations of key procedures, added elaboration where needed, and refined the overall narrative to improve clarity and coherence, ensuring that the contributions of WIPES are presented with stronger scientific rigor.

1. The manuscript briefly mentions both direct electrostimulation (via WIPES) and indirect electrostimulation, but a more detailed comparison between the two methods is lacking. While direct electrostimulation is emphasized for its efficiency, indirect stimulation offers benefits such as stability and ease of implantation. A more thorough discussion on the advantages and limitations of both methods would strengthen the manuscript, particularly in terms of long-term safety, mechanical stability, and performance comparison with existing commercial devices. Additionally, further analysis of the mechanical properties and long-term biocompatibility of WIPES would enhance the overall scientific rigor.

We appreciate this constructive suggestion. In the revised manuscript, we expand the Discussion to contrast WIPES with SNS in terms of targeting specificity, long-term safety, mechanical stability, and user burden; and add Table S1 providing a quantitative comparison between WIPES and representative commercial SNS systems (Medtronic InterStim™ II and Micro, Axonics R20). Key commercial parameters (Volume, recharge interval, Power source, Device price) are drawn from manufacturer documentation and recent reviews; where public device pricing is unavailable, we note this transparently (third-party reseller quotes are shown only as indicative and not as total procedural costs).

Table S1. Quantitative comparison of WIPES vs. commercial sacral neuromodulation systems

**System**	**WIPES**	**Medtronic InterStim™ II**	**Medtronic InterStim™ Micro**	**Axonics R20**
**Implant type**	Thin, flexible patch directly on urethral sphincter	Primary-cell IPG + S3 tined lead (Indirect SNS)	Rechargeable IPG in gluteal pocket + S3 tined lead (indirect SNS)	Rechargeable IPG + S3 tined lead (indirect SNS)
**Volume**	0.3 cm^3	14 cm^3	2.8 cm^3	5 cm^3
**Power source**	Wireless power	Primary-cell lithium battery	Rechargeable battery (transcutaneous energy transfer)	Rechargeable battery (transcutaneous energy transfer)
**Recharge interval**	N/A	N/A	~20 min weekly	6–10 months
**Targeting**	Direct anatomical placement on sphincter	S3	S3	S3
**Effective rate of treatment**	UUI:90.62%<br>SUI: 97.92%	UUI~76%<br>(≥50% reduction)	UUI:79-89%<br>(≥50% reduction)	UUI:70-90%<br>(≥50% reduction)

**Device price**	$120	>$10,000	>$10,000	>$10,000
**Portability notes**	Fully implanted and external flexible belt Tx supports daily/home use	Fully implanted	Fully implanted	Fully implanted

These additions clarify the complementary strengths and limitations of direct (WIPES) versus indirect (SNS) strategies and situate WIPES within the current clinical device landscape.

2. The WIPES device comprises a six-layer structure including metallic circuits and multiple coils. This multilayered architecture may be susceptible to mechanical degradation, particularly under repetitive tensile or flexural stress. Long-term mechanical reliability, including risks such as interlayer delamination, should be addressed. Additional mechanical characterizations—such as cyclic tensile testing or fatigue analysis—would substantiate the device's durability and its compatibility with dynamic biological tissues.

We appreciate the reviewer's concern and have added dedicated mechanical reliability tests. We performed cyclical bending and twisting fatigue (1000 continuous cycles at 0.2 Hz) on a motorized stage while real-time monitoring WIPES output to track pulse amplitude and waveform fidelity (refer to the section of 'Mechanical reliability testing of WIPES' in the Methods and Fig. S19 for more details). The device maintained stable stimulation characteristics throughout testing, and no functional degradation was observed. To address the risk of interlayer delamination, we further conducted an interlayer adhesion (180° peel) test following ASTM D903 at 30$\frac{mm}{min}$orce–time curves and extracted metrics are reported in Fig. S20, showing robust bonding without catastrophic delamination (transient force dips correspond to the device's central hollow geometry, as explained in Methods). Together, these results substantiate the durability of the six-layer architecture under repetitive flexural/torsional stress and its compatibility with dynamic biological motion.

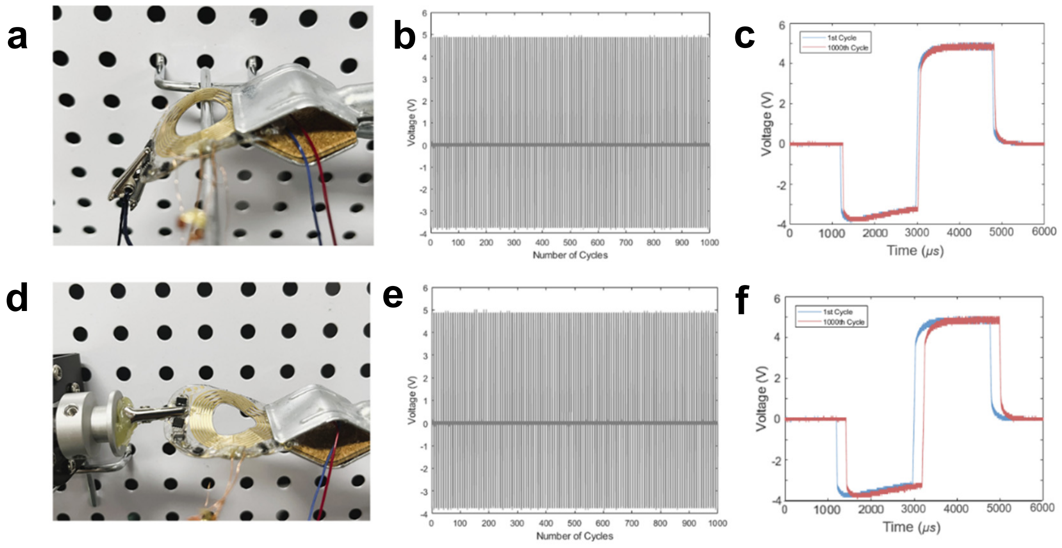

Fig. S19. Cyclical bending and twisting test. a. Setup of the bending experiment, b. Stimulation pulse during 1000 bending cycles, c. Variation of stimulation pulse after 1000 bending cycles, d. Setup of the twisting experiment, e. Stimulation pulse during 1000 twisting cycles, f. Variation of stimulation pulse after 1000 twisting cycles.

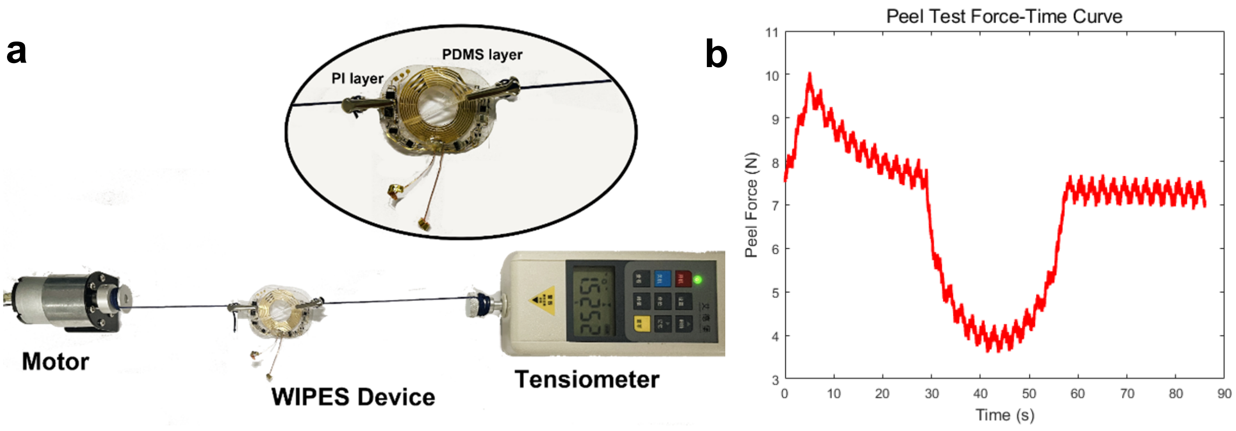

Fig. S20. Interlayer adhesion strength test of WIPES.

==

Location of Change: Line 162， Section 'Mechanical reliability testing of WIPES' in the Methods and Fig. S19, S20.

Original Sentences: /

Revised Sentences: In addition, mechanical reliability tests including cyclic bending, torsion stretching and interlayer adhesion strength test for WIPES device were conducted to evaluate its durability under deformation (refer to the section of 'Mechanical reliability testing of WIPES' in the Methods and Fig. S19 and S20). The results confirmed excellent mechanical stability and insulation integrity of the encapsulated electrodes during repeated flexural and tensile loading.

==

3. The manuscript defines the activation power threshold as 0.15 W, highlighting stable operation across various coil alignments and distances. However, the rationale behind selecting this specific threshold is not clearly articulated. Including experimental or theoretical support—e.g., safety margins for tissue heating, power efficiency analyses, or threshold screening—would strengthen the interpretation of results shown in Figures 2c–e.

Thank you for pointing this out. In the revision, we clarify that 0.15 W is the minimum Tx input power at 6.78 MHz that reliably initiates and sustains the programmed therapeutic pulses at the urethral sphincter (60 Hz, fixed pulse width) across all coil alignments and separations tested in Fig. 2c-e. We determined this value by threshold screening (increasing Tx input from 0.05 W in small steps while monitoring the implant output), and selected the lowest power that met the stimulation criteria in the worst-case alignment/depth; using a lower value led to intermittent start-up under off-axis conditions, whereas higher values did not materially improve link robustness in those tests. To address safety, we added an HFSS-based EM-thermal analysis (Fig. S22), showing sub-degree temperature rise in overlying tissue and local SAR well below accepted trunk limits, providing a conservative thermal margin for chronic use.

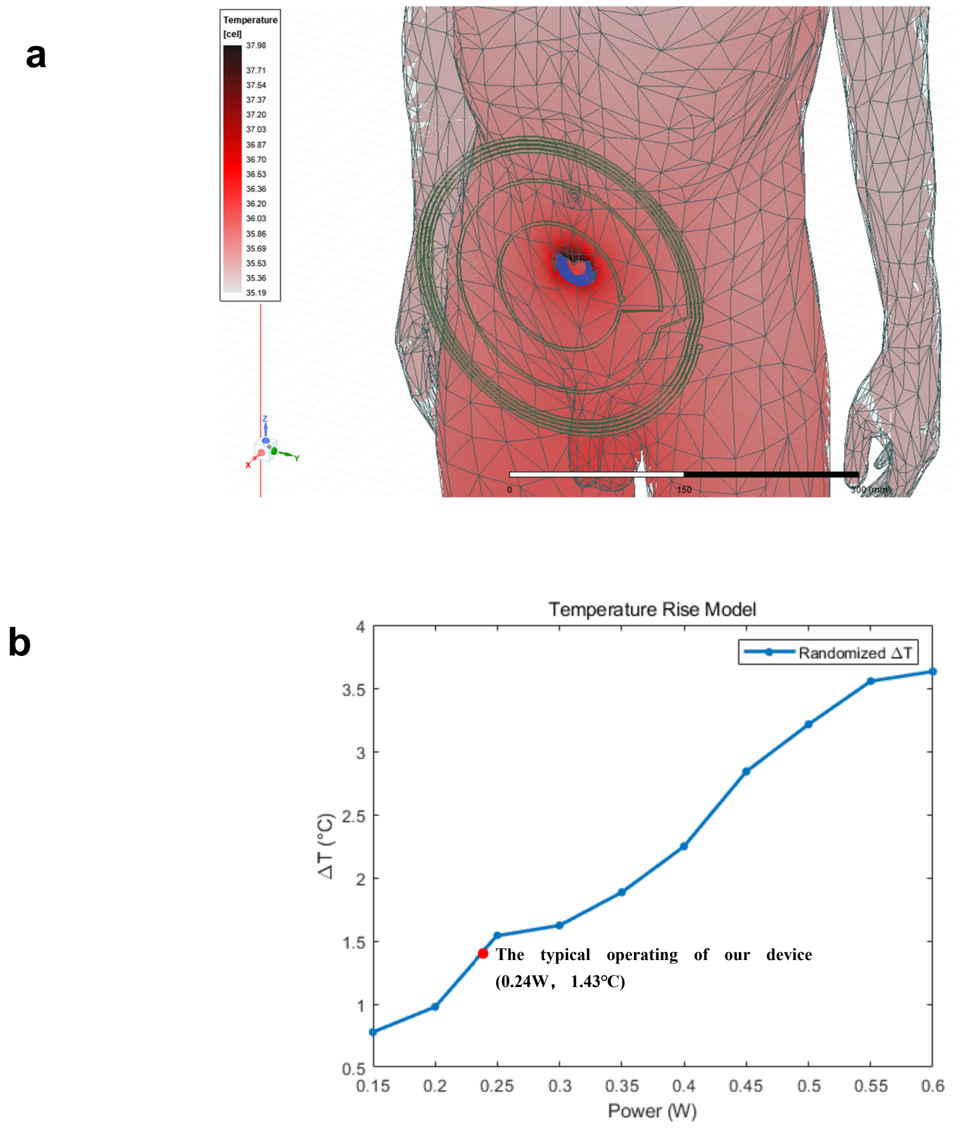

Fig. S22**.** Thermal simulation and power–temperature characteristics of the WIPES system. (a) Temperature distribution simulated using the ANSYS HFSS–Icepak coupled thermal module under 0.45 W received power. The model assumes a baseline tissue temperature of 35 °C, full PDMS encapsulation of the implant, and physiological heat dissipation within surrounding soft tissues. The localized hotspot around the implanted receiver demonstrates a maximum temperature rise of approximately 2.71 °C, remaining below commonly accepted biomedical safety thresholds. (b) Modeled temperature rise (ΔT) as a function of received power from 0.15–0.60W in 0.05W increments. Randomized variation was introduced to reflect tissue heterogeneity and implant–tissue thermal coupling variability.

==

Location of Change: /

Original Sentences: /

Revised Sentences: Thermal-safety analysis based on coupled HFSS–Icepak simulations (Fig. S22) further confirmed that the device operates within safe temperature limits under its rated power, ensuring stable and biocompatible performance.

==

4. The manuscript shows that the gold electrostimulator emerges directly from the WIPES device without any encapsulating layer. This unsealed interface could permit extracellular matrix (ECM) infiltration through

the interface gap, potentially causing device malfunction during long-term implantation. It is recommended that the authors discuss strategies to address this risk, such as soft encapsulation layers or self-healing materials, and provide evidence supporting the device's functional stability under chronic in vivo conditions.

We appreciate the reviewer's concern about the unsealed electrode interface and potential ECM infiltration. In the revised manuscript, we clarify that only the small gold stimulation site is intentionally exposed, whereas all interconnects and surrounding metal layers are fully encapsulated by a multilayer passivation/overmold (PI/parylene-C with a PDMS soft overcoat). The stimulation site is a small gold patch that directly contacts the urethral sphincter and is connected by a thin gold lead (Insulating wrapped). To substantiate chronic stability, we report longitudinal EIS measurements at the gold-sphincter interface (refer to the section of 'Electrochemical impedance spectroscopy (EIS) of WIPES electrodes'), which show no significant drift in the therapeutic-band impedance, indicating sustained low-resistance coupling without progressive encapsulation effects. We have also added a brief discussion of mitigation strategies—including conformal soft encapsulation around the electrode perimeter and self-healing silicone/PU elastomers for future iterations-to further guard against ECM ingress during long-term implantation.

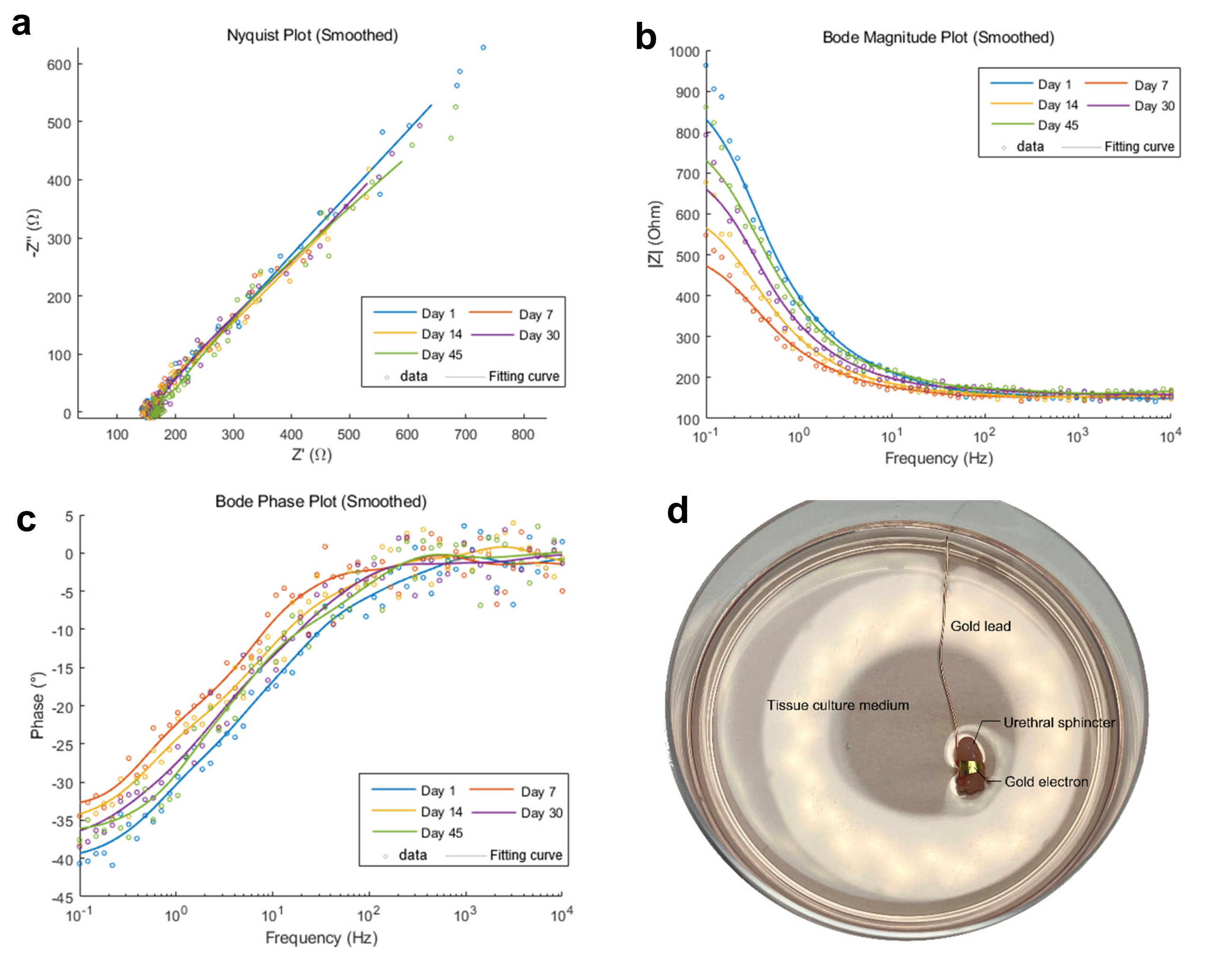

Fig. S21. Electrochemical impedance spectroscopy (EIS) of WIPES electrodes. (a–c) EIS measurements at Days 1, 7, 14, 30, and 45 post-implantation in PBS. The Nyquist, Bode magnitude, and phase plots. (d) Ex vivo image of a rat urethral sphincter wrapped by the gold electrode.

==

Location of Change: Line 190, Section 'Electrochemical Impedance Spectroscopy (EIS) of WIPES Electrodes' in the Methods and Fig. S21

Original Sentences: /

Revised Sentences: Furthermore, electrochemical impedance spectroscopy (EIS) of the WIPES electrodes demonstrated stable interfacial impedance for at least 45 days under physiological conditions, confirming reliable and low-resistance electrode–tissue coupling during long-term operation (refer to the section of ‘Electrochemical impedance spectroscopy (EIS) of WIPES electrodes’ in the Methods and Fig. S21).

==

5. The transmitter (TX) coil used in this study appears to be approximately 20 cm in diameter, which may pose limitations for practical use. If such a large TX coil is required for effective power delivery, its real-world applicability could be restricted. The authors should discuss how the device performance varies with TX coil dimensions and whether miniaturization could compromise efficiency. Experimental data or modeling results examining TX size-dependent performance would be a valuable addition.

Thank you for the comment. In our in-vivo rat experiments, animals were kept awake and unrestrained to emulate the intended clinical scenario and to avoid repeated anesthesia or restraint-related injury. Each rat ambulated freely in a 20 cm-diameter transparent bowl (Fig. 4e). We therefore placed a co-axial ~20 cm TX loop beneath the bowl so that the magnetic field covered the entire movement footprint. With the loop diameter matched to the arena, the rat's typical lateral displacements (within ≤10 cm of the center) experienced a quasi-uniform magnetic flux density, which kept coupling and received power nearly constant regardless of position or orientation and maintained stable stimulation waveforms throughout the session. This geometry was chosen solely to support awake, freely moving experiments and does not imply a requirement for a rigid 20 cm TX in practice. For human use, the transmitter is a flexible belt-integrated winding worn around the waist (Fig. 1b). It adopts a low-profile to maintain coupling under posture changes while remaining lightweight and comfortable for extended wear. This belt form factor supports home-based therapy and fits unobtrusively under clothing, without reliance on stationary power sources.

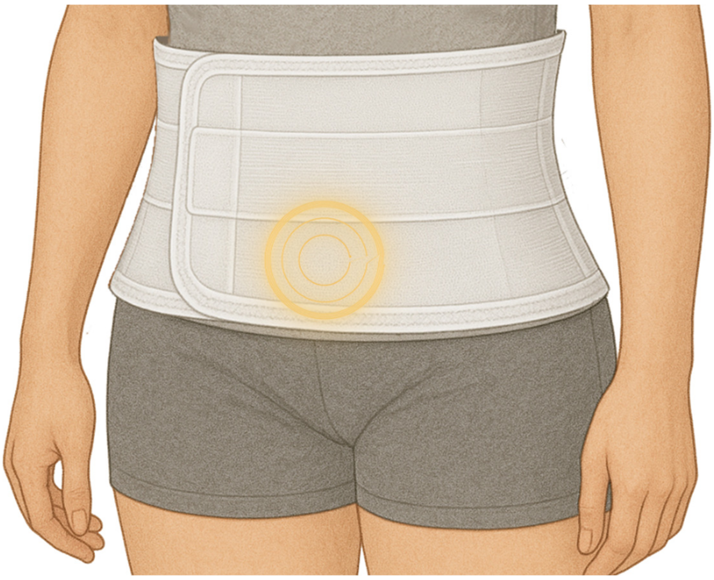

Fig. 1b Wearable transmitting belt enabling wireless power transfer to the implanted WIPES device.

==

Location of Change: Line 110

Original Sentences: Patient could receive electrostimulation treatment at customized frequencies and voltages for a designed period in a clinic or at home.

Revised Sentences: Patient could receive electrostimulation treatment at customized frequencies and

voltages for a designed period in a clinic or at home using the belt-integrated Tx coil (Fig. 1b), which is worn around the waist to provide portable, continuous power to WIPES.

==

Reviewer 3:

The manuscript by Zheng et al describes an implantable wireless neuromuscular stimulator to interface with the urethra and reduce symptoms of urinary incontinence. The fabricated device is novel, and its flexibility is an improvement to other wireless stimulators available.

We appreciate the reviewer's recognition of the device's novelty and flexibility; in revision, we tightened the comparison to existing wireless stimulators and clarified the urethral interface design and performance evidence to present the contribution more clearly.

Major comments 1. From the introduction, authors define MUI as the target for their stimulation therapy; however, the animal model with injection of CTX is not adequate to test their hypothesis. First, it is unclear why authors hypothesize that stimulating the smooth muscles of the urethral sphincter would be beneficial to treat the urge component of UUI and MUI. If the goal is for authors to stimulate the sphincters, then this is a treatment for SUI only, without addressing the urge components.

We thank the reviewer for raising this central point concerning disease scope and model adequacy. Our therapeutic intent is to address mixed urinary incontinence (MUI) by targeting its two components through a single, minimally invasive interface: restoration of urethral outlet resistance and sphincter control relevant to SUI, and neural restoration of the bladder-sphincter reflex pathways via engagement of peri-urethral afferents relevant to UUI. To prevent misunderstanding, we have revised the Introduction to state explicitly that WIPES does not seek to stimulate 'smooth muscle alone', rather, the device delivers programmed pulses to the external urethral sphincter (striated muscle) and the adjacent peri-urethral nerve plexus, thereby producing both a myogenic effect on sphincter tone and a neurogenic effect via pudendal afferent signaling within the sacral circuitry. Regarding disease models, there is currently no single robust rodent model that reproduces MUI in one preparation. We initially explored sequential dual modeling but observed compromised survival and organ integrity, precluding reliable longitudinal testing.

In the revision, we therefore model the two groups separately using standard, sex-appropriate paradigms and make this logic explicit. For UUI (in both sexes), we employ cyclophosphamide (CTX)-induced cystitis, which is a widely used and standardized model of detrusor overactivity (refer to the section of 'Rat models of urgency urinary incontinence' in the Methods). For SUI, we use vaginal balloon distension in females to recapitulate birth-trauma-related pelvic floor/sphincter injury, and a post-prostatectomy-like model in males that combines prostatectomy with focal electrocautery of the external urethral sphincter to reduce outlet resistance, mirroring clinical post-prostatectomy incontinence (refer to the section of 'Rat models of stress urinary incontinence' in the Methods). In SUI modelling groups, beyond urinary sand masses collection quantifying urination frequency and average urination volume, we now report leak point pressure (LPP) and external urethral sphincter electromyography (EUS-EMG) as functional readouts, together with histology and molecular analyses including HE, Masson, TUNEL, and expression of α-SMA and ROCK2 (refer to the section of 'Urodynamic Evaluation.', and 'Urethral sphincter muscle tissue staining'). In UUI modelling groups, in addition to urinary sand masses collection, we incorporated continuous cystometrogram (CMG), neuronal markers (nNOS, VIP, PGP9.5 and GAP-43), peripheral neural tissue staining, and c-Fos immunolabeling in the periaqueductal gray (PAG) (refer to the section of 'Urodynamic Evaluation.', 'Neural Tissue Staining.' and 'Immunofluorescence Staining') to probe urge-related neuronal activity. These additions clarify that peri-urethral/sphincter stimulation by WIPES is not only myogenic-improving outlet competence in SUI-but also neural restoration engaging sensory-motor circuits that mitigate the urge component in UUI. Collectively, the revised 'model-mechanism-indication' alignment supports our rationale for MUI and addresses the reviewer's concern about disease targeting and model adequacy.

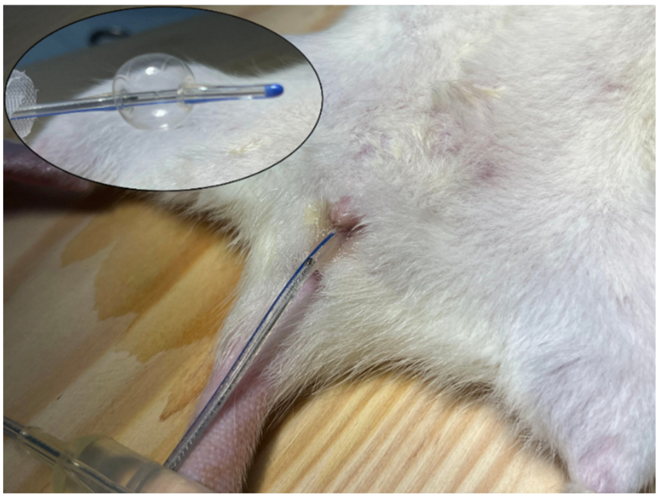

Fig. S23. Urethral balloon dilatation for establishing the SUI model in female rats.

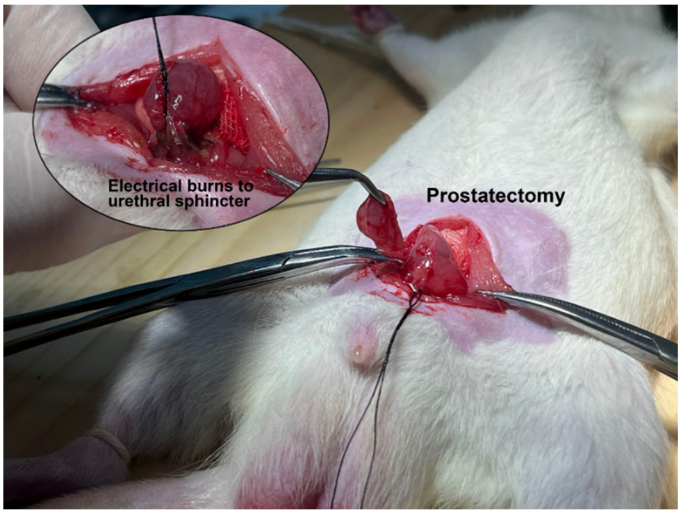

Fig. S24. Intraoperative procedure of combined SUI model induction in male rats through prostatectomy and electrical burns to the urethral sphincter and associated nerves.

===

Location of Change: Line 29

Original Sentences: In rats of induced mixed incontinence, WIPES provides stimulation in vivo while they are moving freely in cages, and results in around 81% relief in urination frequency and volume. Its principle is uncovered as modulation of nerve signaling and regeneration of the sphincter muscles, filling the gap of the restoration mechanism in rat incontinence models.

Revised Sentences: In rats with experimentally induced urge or stress incontinence (modeled in separate but otherwise comparable cohorts), WIPES delivered in-cage, free-moving stimulation and achieved average alleviation rates of 90.62% (UUI) and 97.92% (SUI) in urination frequency and volume. Mechanistically, the therapeutic benefit reflects modulation of peri-urethral neural signaling and regeneration of the sphincter musculature, addressing a previously unfilled restoration pathway in rat incontinence models.

Location of Change: Line 92

Original Sentences: After stimulation in rats with medicine-induced mixed incontinence (dominant UUI accompanied by SUI), the improvement rate of incontinence symptoms reached 81% statistically, shown by a clear reduction in urination frequency and an increase in average urination volume.

Revised Sentences: After stimulation in rats with experimentally induced UUI or SUI (modeled in two separate cohorts because true MUI cannot currently be created in a single rat), the improvement rates reached 90.62% (UUI) and 97.92% (SUI), as evidenced by reduced urination frequency and increased average urination volume.

Location of Change: refer to the section of 'Rat models of urgency urinary incontinence' in the Methods

Original Sentences: /

Revised Sentences: A new paragraph has been added in the Methods section to describe the procedures for establishing the rat model of urgency urinary incontinence (UUI), including model induction, validation, and postoperative management. The added description provides sufficient methodological detail to ensure reproducibility.

Location of Change: refer to the section of 'Rat models of stress urinary incontinence'' in the Methods and Fig. S23-24

Original Sentences: /

Revised Sentences: A new subsection has been added in the Methods to describe the procedures for establishing the rat model of stress urinary incontinence (SUI), including the modeling technique, validation process, and postoperative evaluation. Corresponding schematic illustrations and representative validation results have been included in Figs. S23–S24 to support reproducibility and clarify the experimental setup.

==

2. Furthermore, the tests conducted (urine sand mass counting & weighting) are not adequate to represent an effect on MUI (nor UUI/SUI by themselves). One of the major issues with the conducted test is that the bladder fill is unknown at the time of involuntary leak, which makes it impossible to characterize the incontinence phenotype and thus its rehabilitation. Authors need to conduct an urodynamics test and leak-point-pressure test (at least at the terminal point) to validate the effect of urethral closure via electrical stimulation of the sphincters.

We agree that metabolic-cage assays alone do not reveal the bladder fill at the time of involuntary leakage. To remove this ambiguity, we added controlled urodynamic testing and aligned all leak events to known filling states. For UUI, we performed CMG starting from an emptied bladder and infused warm saline at a fixed rate (0.08–0.10 mL/min). Because the infusion rate and time are known, the intravesical volume at any event (including involuntary leak or non-voiding contractions) is defined ($V(t) = Q \cdot t$), and is cross-checked by post-event aspiration and gravimetric urine loss where applicable. CMG thus provides leak threshold/peak pressures, capacity, inter-contraction interval, and non-voiding contraction (NVC) burden under a controlled fill state. For SUI, we measured LPP at a standardized intravesical volume (three trials averaged, cmH_2O) and recorded EUS-EMG simultaneously to verify sphincter activation (guarding/voiding bursts). These additions establish the bladder volume and pressure at leak, allow clear phenotype classification (outlet failure vs detrusor overactivity), and quantify rehabilitation with stimulation: WIPES raises the LPP and augments EUS-EMG in SUI, while in UUI it reduces NVCs, prolongs inter-void intervals, and increases capacity under known filling conditions. Detailed protocols are provided in Methods (Urodynamic Evaluation: SUI-EUS-EMG & LPP; UUI-CMG) and the corresponding results are presented in the revised figures.

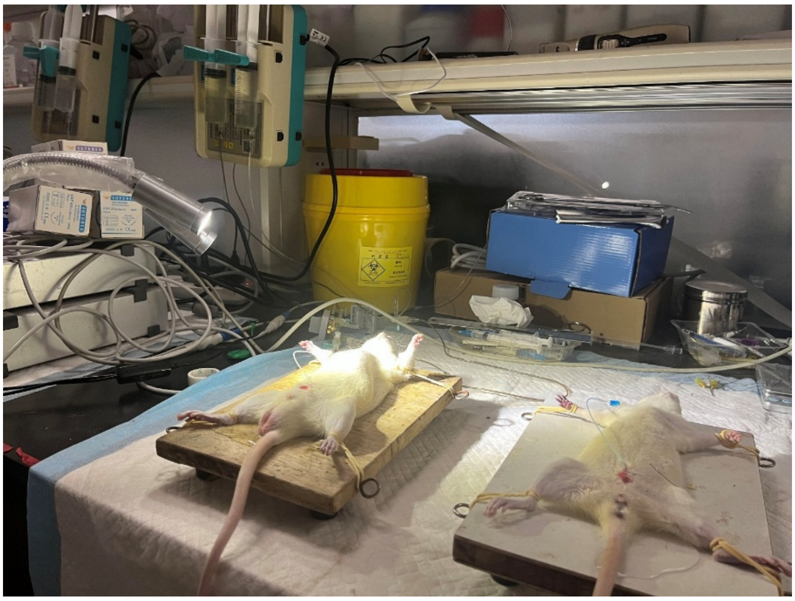

Fig. S28. Experimental setup for cystometry in male and female rats.

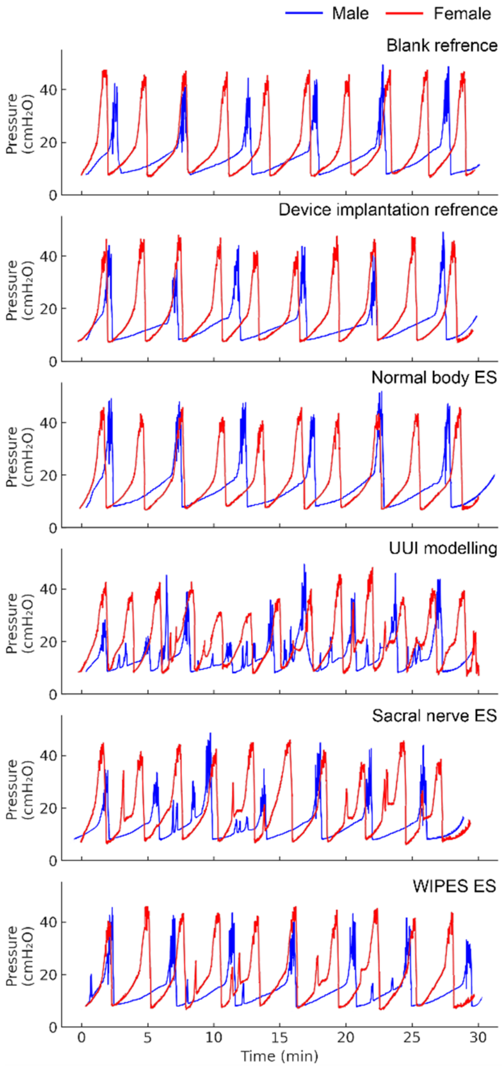

Fig. 5a. Representative cystometric traces of male and female rats under different conditions for 6 groups.

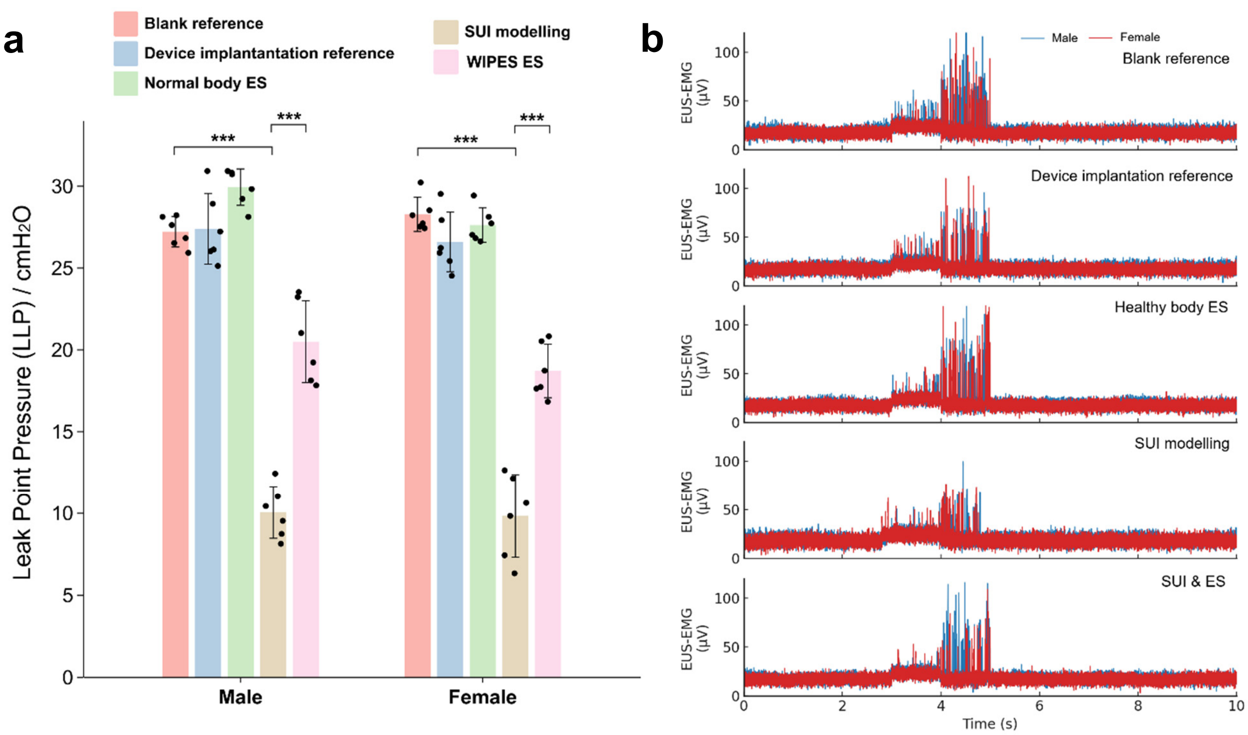

Fig. 6a. Leak point pressure results for five groups, showing significant LPP recovery after WIPES stimulation in both male and female rats. 6b. Representative external urethral sphincter electromyography (EUS-EMG) recordings of all groups

==

Location of Change: Line 308

Original Sentences: /

Revised Sentences: The cystometric analysis (Fig. 5a) provides quantitative assessment of bladder activity by measuring intravesical pressure changes over time (refer to the section of 'Urodynamic Evaluation' in the Methods, and Fig. S28). Three key parameters—non-voiding contraction (NVC) frequency, inter-contraction interval (ICI), and bladder capacity—were analyzed to characterize detrusor muscle function. Rats in the UUI modelling group exhibited abnormally frequent NVCs and markedly shortened ICIs, indicating excessive and unstable detrusor activity associated with urge incontinence. The reduced bladder capacity further confirmed the loss of coordinated neural control and premature triggering of voiding reflexes. In contrast, WIPES electrostimulation (WIPES ES) effectively normalized these parameters. The frequency of NVCs was significantly reduced, while the ICI was prolonged to levels comparable to healthy controls, reflecting restored detrusor stability and improved bladder compliance. Meanwhile, the measured bladder capacity increased markedly, suggesting that the WIPES stimulation enabled fuller bladder filling before the initiation of micturition, indicative of improved neural regulation of the micturition reflex. The SNS group also showed comparable improvement, with slightly more irregular NVCs.

Location of Change: Line 414

Original Sentences: /

Revised Sentences: Cystometric measurements and leak point pressure (LPP) testing (Fig. 6a; refer to the section of 'Urodynamic Evaluation' in the Methods) revealed a pronounced reduction in LPP in rats subjected to SUI modelling (4th) compared with the Blank group (1st), with a decrease of 64.24%, indicative of compromised urethral closure competence and weakened sphincteric resistance to bladder pressure. The decreased LPP reflects impaired transmission of contractile force within the external urethral sphincter

(EUS), resulting in premature urine leakage upon elevated intravesical pressure. Following WIPES ES group (5th), both male and female rats exhibited a substantial recovery of LPP values, with an increase of 97.90%, approaching those of the healthy baseline, demonstrating the efficacy of electrical stimulation in restoring urethral outlet resistance (Mean ± SD for N = 8 rats. ***$P < 0.001$).

Location of Change: Line 419

Original Sentences: /

Revised Sentences: Concurrently, electromyographic recordings from the EUS (EUS-EMG, Fig. 6b) revealed characteristic alterations in neuromuscular activity patterns. In SUI-modelled rats, the EMG traces showed sparse, low-amplitude, and poorly coordinated bursts during voiding, reflecting disrupted neuromuscular communication and reduced sphincter excitability. In contrast, WIPES-treated animals exhibited rhythmic, high-frequency spike bursts synchronized with the micturition cycle, indicative of reinstated reflex control and functional re-engagement of motor units within the sphincter. These findings collectively confirm that WIPES stimulation not only enhances urethral closure pressure mechanically, as evidenced by LPP recovery, but also re-establishes physiological neuromuscular coordination essential for continence regulation.

Location of Change: refer to the section of ‘Urodynamic Evaluation’ in the Methods and Fig. S23-24

Original Sentences: /

Revised Sentences: A new subsection has been added in the Methods to describe the procedures for Urodynamic Evaluation, including the experimental setup and measurement protocols used for assessing bladder and sphincter function in both UUI and SUI models. The section details CMG for evaluating detrusor activity, LPP testing for quantifying urethral closure pressure, and EUS-EMG for monitoring neuromuscular coordination during voiding. The experimental setup for cystometry in male and female rats is illustrated in Fig. S27 to ensure methodological clarity and reproducibility.

==

3. An additional concern is that the sphincter closure via electrical stimulation may improve continence, but it does not do anything for bladder overactivity. Meaning that, while potential therapy can lead to continence, the urge component will not be addressed.

We appreciate the reviewer's concern that sphincter closure alone might not ameliorate detrusor overactivity. Our data and analysis indicate that WIPES electrical stimulation acts beyond purely mechanical outlet augmentation. By engaging peri-urethral afferents, it supports neuromuscular restoration of the bladder-sphincter reflex pathways relevant to urge control. In the UUI model, we therefore evaluated urge physiology under controlled filling with CMG not only relying on urinary sand mass counts. Under fixed-rate infusion, WIPES reduced the burden of NVCs, prolonged the inter-contraction interval, and increased functional capacity, establishing suppression of detrusor hyperexcitability at known bladder volumes/pressures. These functional improvements were accompanied by lower c-Fos in the periaqueductal gray (dlPAG and vlPAG) and a shift of nNOS/VIP signals toward baseline in relevant neural tissues. Physiologically, this nNOS/VIP normalization indicates a re-balancing of inhibitory neuromodulatory tone and reduced aberrant afferent drive, consistent with dampened urge signaling. In parallel, in SUI cohorts WIPES increased EUS-EMG guarding/voiding bursts and raised leak-point pressure, confirming restoration of urethral outlet competence when stress leakage is the primary defect. In sum, WIPES electrical stimulation restores sphincteric closure in SUI and—via peri-urethral afferent engagement—actively dampens detrusor overactivity in UUI, thereby addressing both components of mixed urinary incontinence—stress (SUI) and urge (UUI) with a single minimally invasive interface.

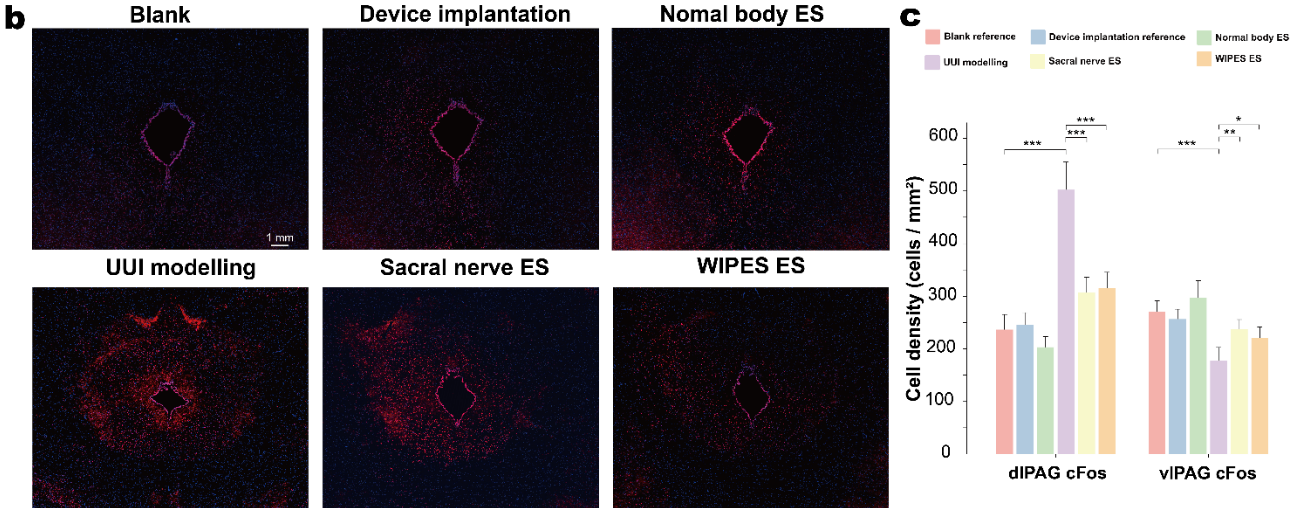

Fig. 5b. c-Fos immunostaining images of the dorsal and ventrolateral periaqueductal gray (dPAG and vlPAG) regions in rats from each group. 5c. Statistical analysis of c-Fos–positive cell densities in dPAG and vlPAG regions.

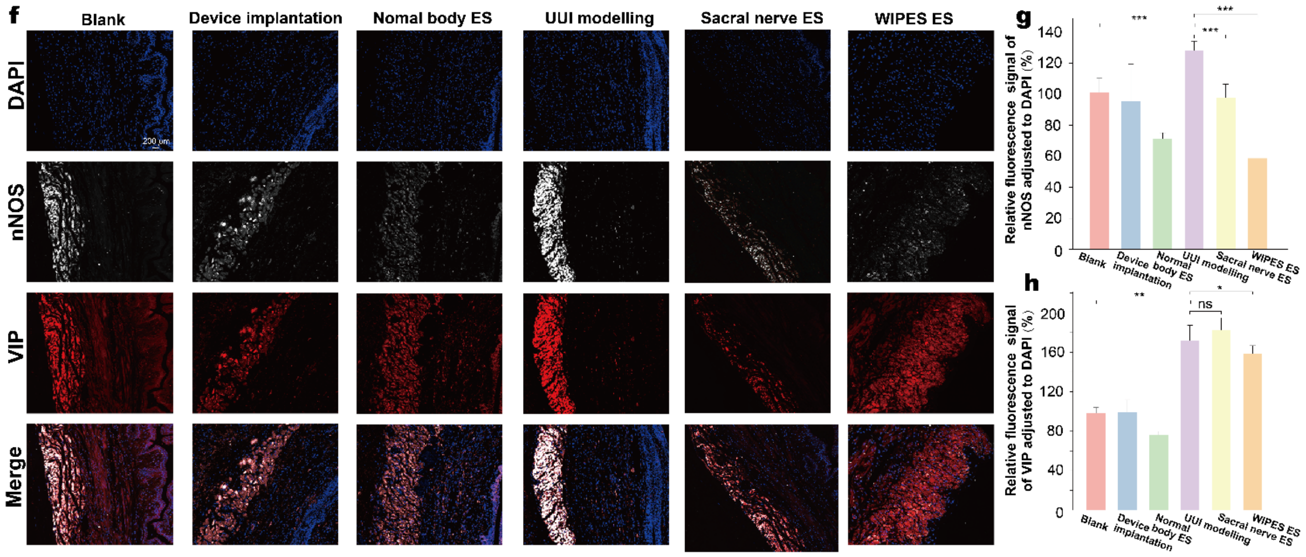

Fig. 5f. Immunofluorescence staining of nNOS and VIP neurotransmitters in urethral sphincter tissues of rats in each group. 5g. Quantitative comparison of nNOS fluorescence intensity normalized to DAPI for all groups. 5h. Quantitative comparison of VIP fluorescence intensity normalized to DAPI for all groups.

==

Location of Change: Line 321

Original Sentences: /

Revised Sentences: To further investigate how WIPES modulates central neural circuits involved in micturition control, c-Fos immunofluorescence staining was performed in the dorsal periaqueductal gray (dlPAG) and ventrolateral periaqueductal gray (vlPAG) regions of the midbrain (Figs. 5b and 5c) (refer to the section of 'Quantification of c-Fos for urge-suppression assessment' in the Methods). The PAG is a pivotal integrative hub linking bladder sensory input and pontine motor output. Specifically, the dlPAG primarily participates in urine storage and inhibitory control, suppressing the activation of the micturition reflex, whereas the vlPAG is associated with voiding facilitation, relaying excitatory signals to the pontine micturition center (PMC). Thus, the spatial distribution and intensity of c-Fos expression in these two regions provide insight into the excitatory–inhibitory balance within the central micturition network. In the

UUI modelling group (4th), excessive bladder afferent input caused by detrusor overactivity aberrantly activated the dlPAG, leading to an overcompensated inhibitory response attempting to suppress involuntary voiding. Quantitatively, c-Fos expression in the dlPAG increased by 112.81% compared to the blank reference (1st), confirming pronounced hyperactivation of this inhibitory center. In contrast, the vlPAG showed a 34.5% reduction in c-Fos activity, indicating suppression of the voiding-facilitatory pathway and disruption of the normal excitatory relay to the PMC. Such imbalance between facilitation and inhibition underlies the detrusor overactivity characteristic of urge incontinence. Following WIPES ES group (6th), this imbalance was markedly corrected. c-Fos expression in the dlPAG decreased by 37.16%, while the vlPAG exhibited a 33.77% increase, indicating a bidirectional normalization of inhibitory and excitatory activities within the central micturition network. Similarly, SNS ES group (5th) produced comparable effects, with dlPAG activity reduced by 38.86% and vlPAG elevated by 33.78%. These results suggest that both WIPES and SNS treatments effectively rebalanced the dlPAG–vlPAG circuitry, and from the perspective of impulse inhibition, their restorative efficacy showed no significant difference. (Mean ± SD for N = 8 rats; ***P <0.001, **P < 0.01, *P < 0.05).

Location of Change: Line 385

Original Sentences: Furthermore, two markers, nNOS and VIP, were selected to evaluate the growth and function of nerve cells around the urethra. nNOS is a neurotransmitter in urethral nerve cells that induces smooth muscle relaxation and mediates nerve injury and inflammatory pain hypersensitivity, while VIP reduces neuroinflammation and promotes early myelination and growth of regenerative axons in the nervous system (Fig. 5b). Immunofluorescence staining showed that, compared to the Blank reference group (1st), the relative fluorescence intensity of nNOS and VIP proteins was significantly increased in the Incontinence modelling group (4th), with increases of 31.63% (nNOS) and 23.97% (VIP) (Mean ± SD for N = 4 rats; ***P <0.001,**P < 0.01), suggesting that induced urinary incontinence caused inflammatory nerve damage and pain hypersensitivity around the urethra Interestingly, electrostimulation reduced nNOS signals in both the Normal body ES group (3rd), with a decrease of 30.14%, and the UI & ES group (5th), with a decrease of 57.37% (Mean ± SD for N = 4 rats; *P < 0.05, ***P < 0.001) while maintaining VIP levels (Mean ± SD for N = 4 rats; ns, not significant) as shown in Fig. 5f. This reduction suppressed neural pain hypersensitivity, creating a microenvironment conducive to nerve axon regeneration to facilitate recovery from UUI. These combined findings suggest that by promoting smooth muscle contraction via ROCK2 activation to enhance urethral closure, and modulating neural function via nNOS to support nerve regeneration and reduce inflammation-induced dysfunction, WIPES could effectively contribute to the treatment of MUI.

Revised Sentences: Furthermore, elucidate the neuroregulatory mechanisms responsible for the functional recovery observed in cystometric profiles, we examined two markers, nNOS and VIP, were selected to evaluate the growth and function of nerve cells around the urethra (refer to the section of ' Immunofluorescence Staining ' in the Methods). nNOS is a neurotransmitter in urethral nerve cells that induces smooth muscle relaxation and mediates nerve injury and inflammatory pain hypersensitivity, while VIP reduces neuroinflammation and promotes early myelination and growth of regenerative axons in the nervous system (Fig. 5f and Fig. 5h). In Blank reference (1st), nNOS and VIP were distinctly expressed in organized nerve fibers surrounding the urethral sphincter and bladder wall, ensuring synchronized activation of excitatory and inhibitory pathways that maintain stable micturition cycles. In contrast, rats subjected to UUI modelling (4th) exhibited diffuse and disordered nNOS overexpression, accompanied by irregular VIP distribution and reduced spatial colocalization, with increases of 26.73% (nNOS) and 75.13% (VIP) (Mean ± SD for N = 8 rats; ***P <0.001, **P < 0.01). Such aberrant patterns indicate a breakdown in inhibitory control and overactivation of parasympathetic efferent, consistent with the frequent, high-amplitude NVCs observed in cystometric recordings. Following SNS ES (5th) and WIPES ES (6th)

electrostimulations, nNOS signals recovered toward physiologically balanced patterns, with a decrease of 23.67% and 54.30%, VIP levels have different expressions, with increase a little (Mean ± SD for N = 8 rats; ns, not significant) and a decrease of 8.03%. The normalized nNOS distribution implies restored nitric oxide–mediated inhibitory tone, preventing premature detrusor contractions, while the orderly re-expression of VIP indicates re-established neurovascular coupling and mucosal relaxation signaling. These features collectively reflect the functional reconnection of afferent–efferent feedback loops rebuilt through WIPES-induced peripheral reinnervation. By contrast, SNS treatment led to partial normalization, with residual nNOS hyperexpression and sparse VIP regions, suggesting incomplete restoration of inhibitory control.

Location of Change: refer to the section of 'Quantification of c-Fos for urge-suppression assessment' in the Methods

Original Sentences: /

Revised Sentences: A new subsection has been added in the Methods to describe the procedures for Quantification of c-Fos for urge-suppression assessment, outlining the experimental workflow and analytical criteria used to evaluate neuronal activation in brain regions associated with micturition control. The section details tissue preparation, immunofluorescence staining, imaging, and quantification of c-Fos–positive nuclei within the dorsolateral (dlPAG) and ventrolateral periaqueductal gray (vlPAG) regions. Sampling and image analysis were performed under blinded conditions, and standardized thresholds were applied across all groups to ensure consistency and reproducibility in assessing central neural activity related to urge suppression.

==

4. It is clear that the therapy is leading to a reduced frequency of leaks in experimental animals. However, there is no proposed mechanism of action nor its validation with the data collected. Because of this, it is hard to interpret the results in relationship to the phenotype of urinary incontinence.

We appreciate the reviewer's point. The urinary sand mass counting/weighting assay in our study is a behavioral readout of voiding frequency and mean voided volume, applicable to both UUI and SUI, but it does not define bladder fill or phenotype by itself. In the revision, we retain urinary-sand data as a secondary day-to-day metric and add mechanistic validation on three fronts: (1) urodynamics tailored to phenotype—LPP + EUS-EMG for SUI and CMG for UUI, (2) tissue histology—HE, Masson, and TUNEL and (3) molecular/neural markers—for SUI: α-SMA, ROCK2, for UUI: c-Fos mapping and nNOS/VIP profiling. These additions provide controlled-filling physiology and neuro-myogenic context, allowing the behavioral improvements to be interpreted against phenotype-appropriate endpoints.

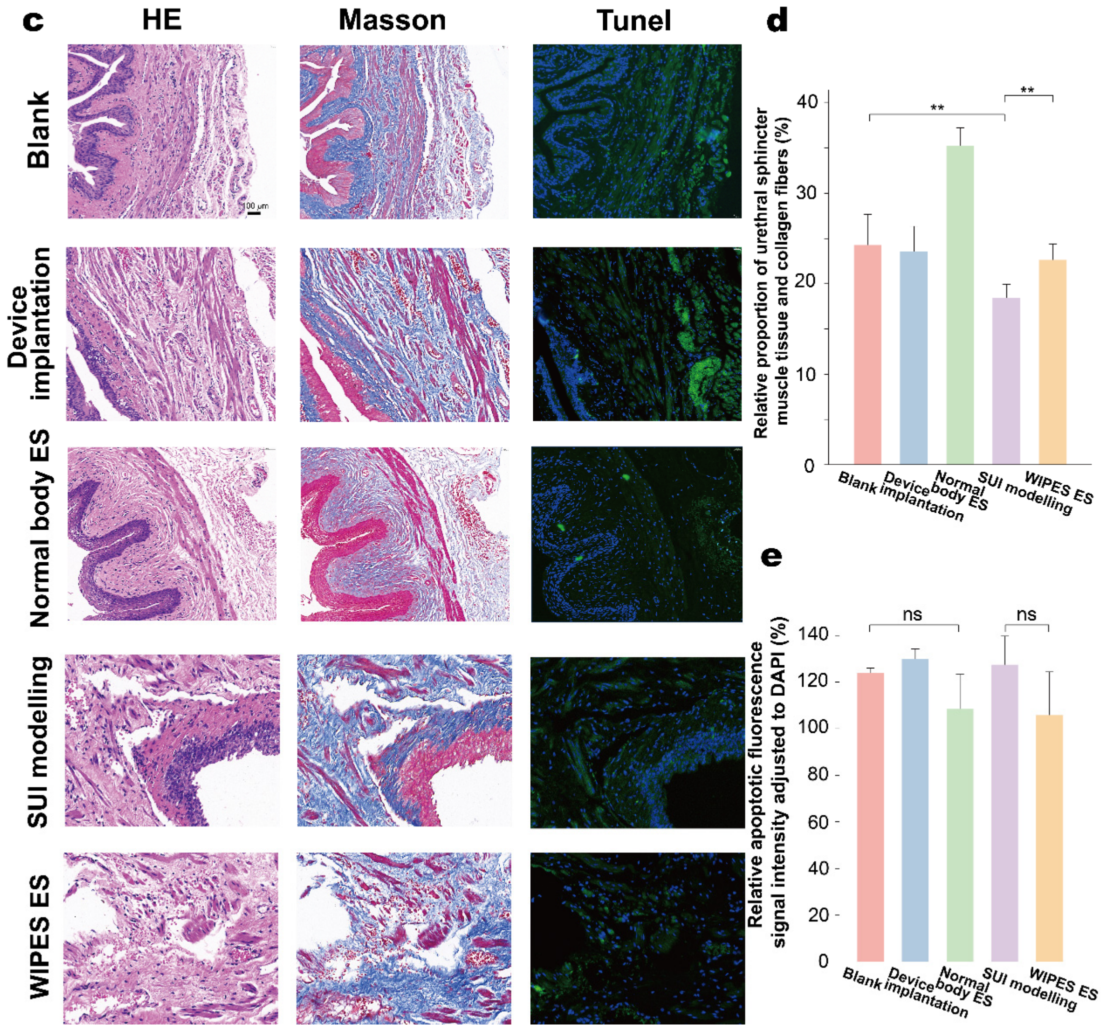

Fig. 6c. Histological evaluation of urethral sphincter tissues using H&E, Masson, and TUNEL staining for all groups. 6d. Quantitative analysis of the relative proportion of urethral sphincter muscle to collagen area for all groups. 6e. Quantification of TUNEL fluorescence signal intensity the five groups.

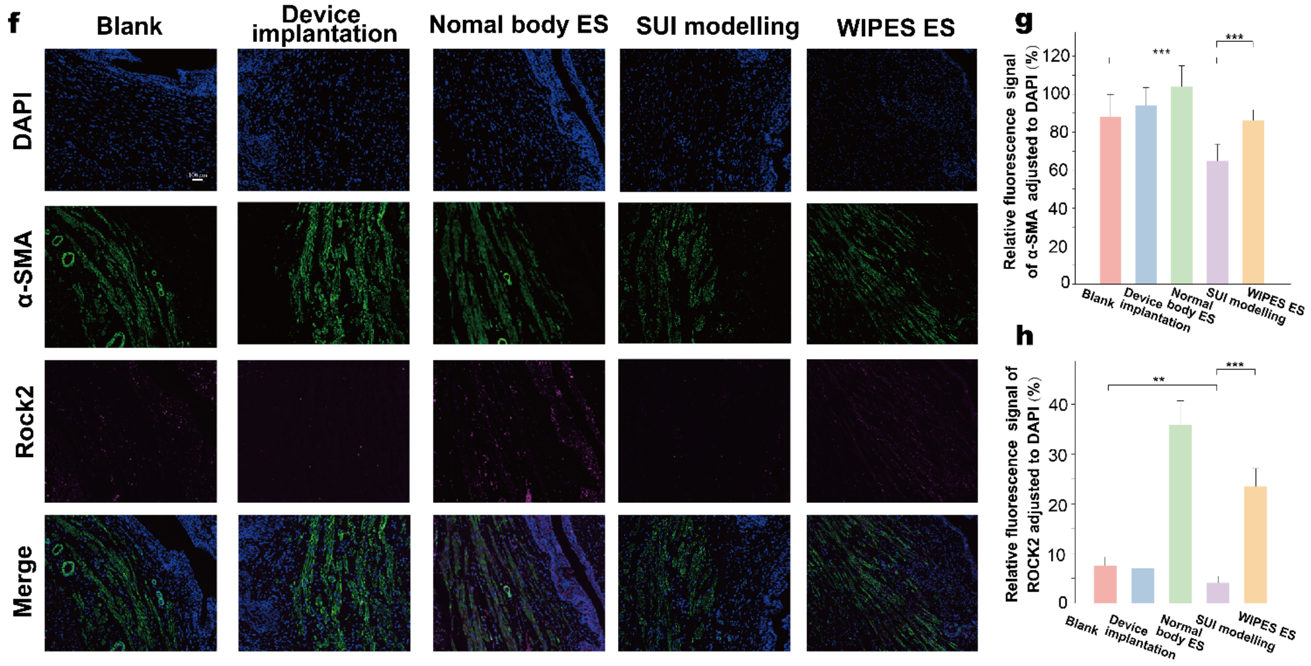

Fig. 6f. Immunofluorescence staining of α-SMA (green) and ROCK2 (magenta) in urethral sphincter tissues, with DAPI (blue) indicating nuclei for all groups. 6g. Quantitative analysis of α-SMA signal intensity

normalized to DAPI for all groups. 6h. Quantitative comparison of ROCK2 fluorescence intensity normalized to DAPI for all groups.

==

Location of Change: Line 432

Original Sentences: In this study, H&E staining was used to verify the integrity of the sphincter tissue, and Masson staining was used to assess the musculature-to-collagen ratio, and lastly both data were combined to comprehensively evaluate the health status of the urethral musculature and the therapeutic effect, as shown in Fig. 6a. Compared to the Blank reference group (1st, the smooth muscle-to-collagen ratio in the Device implanted group (2nd) showed no significant difference, whereas this ratio showed a significant decrease of 21.88% in the Incontinence modelling group (4th) (Mean ± SD for N = 4 rats. **p*< 0.001). Interestingly, the smooth muscle volume in the UI & ES group (5th) showed significant improvement, with an increase of 22.32%, compared to the Incontinence modelling group (4th) (Mean ± SD for N = 4 rats. **P*< 0.05)as shown in Fig. 6b. These results suggest that stimulation by WIPES is a key factor in promoting smooth muscle proliferation and functional recovery, nearly restoring it to normal levels. The histological findings align with the functional evaluations described earlier, confirming its potential to relieve SUI symptoms. In addition, TUNEL, staining was employed as an effective method to demonstrate apoptosis in vivo, The green fluorescence intensity indicated the distribution of DNA associated with programmed cell death in tissues (Fig. 6c). There were no significant differences in green fluorescence intensity among the Blank reference group (1st) and Device implantation group (2nd), indicating that device implantation did not induce apoptosis in urethral tissues, while the implantation of the WIPES device and its associated electrostimulation (3rd and 5th group) neither caused nor exacerbated apoptosis in these tissues.

Revised Sentences: In this study, H&E staining was used to verify the integrity of the sphincter tissue, and Masson staining was used to assess the musculature-to-collagen ratio, and lastly both data were combined to comprehensively evaluate the health status of the urethral musculature and the therapeutic effect, as shown in Fig. 6c. Compared to the Blank reference group (1st), the smooth muscle-to-collagen ratio showed a significant decrease of 24.28% in the SUI modelling group (4th) (Mean ± SD for N = 8 rats. ****P* < 0.001). Interestingly, the smooth muscle volume in the WIPES ES group (5th) showed significant improvement, with an increase of 23.14%, compared to the SUI modelling group (4th) (Mean ± SD for N = 8 rats. **P* < 0.05) as shown in Fig. 6d. These results suggest that stimulation by WIPES is a key factor in promoting smooth muscle proliferation and functional recovery, nearly restoring it to normal levels. The histological findings align with the functional evaluations described earlier, confirming its potential to relieve SUI symptoms. In addition, TUNEL staining was employed as an effective method to demonstrate apoptosis in vivo. The green fluorescence intensity indicated the distribution of DNA associated with programmed cell death in tissues (Fig. 6c). There were no significant differences in green fluorescence intensity among each group, indicating that the experiments did not induce apoptosis in urethral tissues.

Location of Change: Line 446

Original Sentences: The in vivo experimental results demonstrate the effectiveness of WIPES in treating CTX-induced urinary incontinence, which is known as a traditional method for dominantly inducing UUI. We then study the potential mechanism underlying its therapeutic value for both UUI and SUI. On the other hand, for ROCK2, stimulation significantly enhanced the ROCK2 signals in both the Normal body ES group (3rd), with an increase of 782.31%, and the UI & ES group(5th), with an increase of 792.38%, with ROCK2 primarily localized around the urethral smooth muscle cells(Mean ± SD for N = 4 rats, **p*< 0.05.****p* < 0.001), as shown in Fig. 5d.

Revised Sentences: To further investigate the molecular mechanisms underlying tissue recovery, immunofluorescence staining of α-SMA and ROCK2 was conducted (Fig. 6f–h). α-SMA is an actin protein

specifically expressed in smooth muscle, forming the core part of muscle cells and maintaining contractile capacity. ROCK2, a downstream effector of Rho kinase, regulates the proliferation and differentiation of cytoskeletal proteins in smooth muscle cells and is a critical factor in promoting smooth muscle contraction. (refer to the section of ' Immunofluorescence Staining ' in the Methods). It showed a clear distribution of α-SMA in all groups, indicating the structural and functional integrity of the urethral smooth muscle and the α-SMA signals in the WIPES ES group (5th) compared with SUI modelling group (4th), with an increase of 32.63% (Fig. 6f). On the other hand, for ROCK2, stimulation significantly enhanced the ROCK2 signals in the WIPES ES group (5th) compared with SUI modelling group (4th), with an increase of 471.46%, with ROCK2 primarily localized around the urethral smooth muscle cells (Mean ± SD for N = 8 rats; $^{**}P < 0.01$, $^{***}P < 0.001$), as shown in Fig. 6h. We note that the induced incontinence results from nerve inflammation and damage, along with the overexpression of relaxant neurotransmitters. WIPES is believed to mitigate these effects by reducing the expression of relaxant neurotransmitters through electrical signals, maintaining axonal regeneration and myelin growth in nerve cells, and activating the ROCK2 signaling pathway to enhance the proliferation and differentiation of smooth muscle cells, thereby restoring urethral function from causes for SUI.

==

5. Cyclophosphamide induces cystitis, which can mimic the symptoms of bladder overactivity; however, its injection is not appropriate animal model for the complexities of urge urinary incontinence (which include additional symptoms to bladder overactivity), and certainly not appropriate for mixed urinary incontinence. As such, the description in lines 229-231 is not accurate, and authors do not provide sufficient and relevant references to defend the model used.

We appreciate the opportunity to clarify our disease-modeling strategy and have revised accordingly. In this study, CTX-induced cystitis is used specifically as a validated rodent model of detrusor overactivity/overactive bladder (OAB) to probe the urge component (UUI). CTX induced cystitis is a well-established rodent model for bladder overactivity. Systemically administered CTX is metabolized to acrolein, a urotoxic metabolite that damages the urothelium and lamina propria, triggers inflammatory cascades, and can produce hemorrhagic cystitis. These pathogenic features and their molecular mediators are well documented in mechanistic reviews and experimental studies [65]. In rats, CTX or intravesical acrolein reliably yields an OAB phenotype characterized by increased micturition frequency, reduced bladder capacity/voided volume, and enhanced NVCs on cystometry under controlled filling [66]. Contemporary work further underscores that CTX-induced cystitis reproduces clinical features of detrusor overactivity (DO) and has been widely used to interrogate neurochemical/electrophysiological changes and to evaluate interventions in both rats and mice [67]. Recent reviews and preclinical studies explicitly describe CTX-induced cystitis as a reliable, validated model that recapitulates functional and neurochemical hallmarks of bladder overactivity, supporting its use in mechanistic and interventional research [68][69].

Because no single robust rodent model recapitulates MUI, we evaluate MUI by addressing its two clinical components separately: UUI is modeled with CTX-induced cystitis (urge/overactivity), and SUI is modeled using established stress-incontinence paradigms (female vaginal distension to mimic childbirth injury; male post-prostatectomy–like preparations).

[65] Haldar, S., Dru, C., & Bhowmick, N. A. Mechanisms of hemorrhagic cystitis. American Journal of Clinical and Experimental Urology, 2(3), 199–208 (2014).

[66] Okinami, T., Imamura, M., Nishikawa, N. et al. Altered detrusor gap junction communications induce storage symptoms in bladder inflammation: A mouse cyclophosphamide-induced model of cystitis. PLoS ONE, 9(8), e104216 (2014).

[67] Lee, H., Koh, B.H., Peri, L.E. et al. Role of detrusor PDGFRα+ cells in mouse model of cyclophosphamide-induced detrusor overactivity. Sci Rep 12, 5071 (2022).

[68] Hsiang, H. W., Girard, B. M., Ratkovits, L., Campbell, S. E., & Vizzard, M. A. Effects of pharmacological neurotrophin receptor inhibition on bladder function in female mice with cyclophosphamide-induced cystitis. Frontiers in Urology, 2, 1037511 (2022).

[69] Juszczak, K., Wyczółkowski, M., & Thor, P. J. Animal models of overactive bladder: Cyclophosphamide (CYP)-induced cystitis in rats. European Urology Supplements, 8(8), 583–584 (2009).

===

Location of Change: refer to the section of ‘Rat models of urgency urinary incontinence’ in the Methods

Original Sentences:/

Revised Sentences: Urgency urinary incontinence (UUI) was pharmacologically induced in both female and male Sprague–Dawley rats through intraperitoneal injection of cyclophosphamide (CTX, 80 mg/kg), a well-established rodent model of cystitis and detrusor overactivity. Systemically administered CTX is metabolized into the urotoxic compound acrolein, which damages the urothelium and lamina propria, triggers inflammatory cascades, and sensitizes bladder afferents. These processes collectively lead to bladder overactivity, characterized by increased micturition frequency, reduced capacity, and secondary urethral sphincter dysfunction, thereby reproducing the urge component of urinary incontinence.

===

6. The urethral pressure reported ex-vivo showed a maximum average of ~40 Pa (0.30 mmHg); however, this value is not sufficient to achieve continence during an overactive bladder contraction. Albeit this value is in the ex-vivo rat, it was not measured in-vivo and thus, it is hard to tell how much it would change the closure pressure. Furthermore, it is unclear how the pressure relates directly to the therapy here, since the mechanism of action appears to be more rehabilitative (muscle-to-collagen ratio improvement) than direct modulatory (closure of sphincters upon bladder contraction).

Thank you for pointing this out. We have clarified in the manuscript that the value plotted in Fig. 3f/3h is not a bladder or leak-point pressure. It is a catheter squeeze pressure obtained ex vivo with a differential manometer connected to a single-ended air-filled soft tube placed across the urethra (refer to the section of ‘Sphincter Pressure Measurement During Electrostimulation’). During WIPES stimulation the external urethral sphincter (EUS) contracts and locally compresses the tube, increasing intraluminal air pressure. This metric is intended only as a bench validation that stimulation evokes measurable EUS contraction.

Importantly, the raw ~40 Pa trace reflects the net rise above the tube’s elastic preload. Using the elastic resistance calibration described in Methods (accounting for Young’s modulus, geometry, and small-strain deformation), the actual squeezing pressure attributable to EUS contraction is ~100 Pa (≈ 1.0 cmH_2O≈ 0.75 $mmHg$). We report this corrected value and explicitly state that it cannot be equated to continence thresholds, because continence in vivo depends on circumferential coaptation, outlet compliance, and bladder pressure—not on the local contact pressure at a small catheter segment. To avoid confusion, we have renamed this readout “EUS squeeze pressure (ex vivo)” and moved the derivation to the Methods with unit conversions.

To directly address functional continence in vivo, we supplemented the study with urodynamic endpoints: for SUI, we measured LPP at a standardized filling volume and recorded EUS-EMG during stimulation; for UUI, we performed CMG under fixed-rate filling to quantify capacity, inter-contraction interval, and non-voiding contractions. These assays demonstrate that WIPES raises outlet competence in SUI (LPP↑, EUS-EMG↑) and stabilizes bladder dynamics in UUI (NVC↓, Inter-Contraction Interval (ICI)↑, capacity↑), thereby linking the therapy to phenotype-appropriate physiological improvements.

Finally, regarding mechanism, we clarify that WIPES provides two complementary effects: an acute modulatory effect (evoked EUS contraction during stimulation, evidenced by EUS-EMG and the ex-vivo squeeze test) and a chronic rehabilitative effect (histology and molecular markers, e.g. α-SMA/ROCK2, collagen–muscle remodeling, consistent with improved neuromuscular function). We have revised the text to reflect this dual action and to ensure the ex-vivo pressure measurement is interpreted as supportive evidence of contractile activation, not as a surrogate for in-vivo continence pressure.

7. In lines 344-346 authors state that their results indicate modulation of neural signaling, promotion of muscle and nerve cell regrowth, but their results do not directly confirm these hypotheses. For example, terminal electrophysiology and/or urodynamics would be needed to confirm the modulation of neural signals, and nerve cell regrowth would require additional histological evaluation of nerve sections showing such regrowth.

We appreciate the reviewer's request for direct neural evidence. We agree that nNOS and VIP primarily reflect transmitter-specific pathway modulation and cannot alone establish axonal regeneration. Accordingly, we added peri-urethral immunofluorescence focused on two pathway-agnostic, structurally informative readouts: PGP9.5 and GAP-43. PGP9.5 (UCHL1), a pan-neuronal fiber marker that unbiasedly captures sympathetic, parasympathetic, and sensory axons, was quantified from fixed sampling frames placed around the external urethral sphincter, using a blinded analysis pipeline with uniform background subtraction, global thresholding, and skeletonization-based length measurement. We report two metrics—percent positive area (%Area) and fiber length density (skeleton length per unit area)—to distinguish overall fiber coverage from the continuity and maturity of neural trajectories. For GAP-43, rather than relying on colocalization-based ratios, we quantified the absolute GAP-43–positive area and signal distribution within the same sampling frames, providing a direct index of axonal sprouting and regenerative activity without assumptions about overlap with PGP9.5. This approach reflects GAP-43's biological role as a marker of growth-cone formation and regenerative outgrowth and avoids intensity-based or co-registration biases. Under fixed parameters and blinded analysis, WIPES (and SNS) showed significantly higher PGP9.5 fiber content and length density than UUI model controls, together with a more organized and moderate GAP-43 pattern consistent with structured, rather than chaotic, regeneration. Morphological inspection also revealed more continuous and uniformly oriented PGP9.5-positive trajectories after WIPES treatment, indicating that peri-urethral innervation is restored not only in quantity but also in architectural integrity. These structural data corroborate our nNOS/VIP findings, supporting that transmitter-level modulation and anatomical re-innervation occur in parallel. Urodynamic measurements, performed under standardized filling and anesthesia conditions, showed improvement trends consistent with the structural recovery (details provided elsewhere and in Methods: "Urodynamic Evaluation").

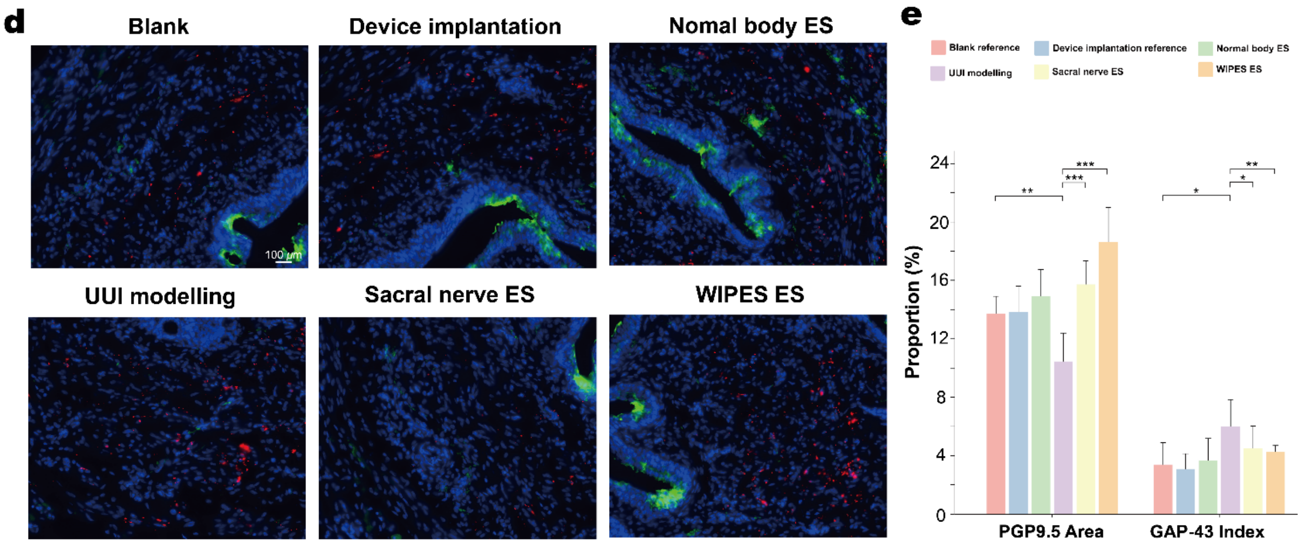

Fig. 5d. Immunofluorescence staining images showing PGP9.5 and GAP-43 expression in urethral sphincter tissues of rats in each group, the green fluorescence indicates the expression of PGP9.5, and red fluorescence indicates the expression of GAP-43. 5e. Statistical analysis of PGP9.5-positive area and GAP-43 area in rats of all groups.

==

Location of Change: Line 345

Original Sentences: /

Revised Sentences: At the central level, c-Fos expression results demonstrated that WIPES electrostimulation effectively suppressed overactivation within the brainstem micturition centers, restoring balanced communication across the bladder–brainstem neural circuit. To further examine the peripheral outcomes of this neuromuscular interface effect, we evaluated nerve regeneration within the urethral sphincter region and synaptic recovery using PGP9.5 and GAP-43 immunofluorescence markers (green and red, Figs. 5d and 5e). PGP9.5 serves as a pan-neuronal marker reflecting the overall density and integrity of peripheral nerve fibers, whereas GAP-43 is a growth-associated protein expressed during axonal regeneration, indicating active neural remodeling and repair (refer to the section of 'Neural Immunostaining & Quantification' in the Methods). As shown in Fig. 5e, the PGP9.5 Area represents the proportion of PGP9.5-positive regions within the total tissue area, quantifying the nerve fiber density. Compared with the blank reference (1st), the UUI modelling group (4th) showed an 23.92% reduction, indicating marked nerve degeneration and fiber loss. Following treatment, the SNS ES group (5th) exhibited a 50.53% increase relative to the model group, reflecting partial recovery characterized by discontinuous and loosely organized fibers. In contrast, the WIPES ES group (6th) demonstrated a 78.43% increase in PGP9.5 Area, showing densely packed and continuous neural structures, indicative of robust peripheral nerve restoration. To further assess regenerative activity, GAP-43—an established marker of axonal sprouting and early-stage neural remodeling—was quantified across all groups. The UUI modelling group (4th) exhibited a markedly elevated GAP-43 signal, showing a 77.54% increase compared with the blank reference, indicating extensive but disorganized compensatory sprouting of immature axons following nerve injury. In contrast, SNS ES group (5th) reduced GAP-43 expression by 25.06%, and WIPES ES group (6th) by 28.99%, relative to the UUI model, suggesting that electrical stimulation helped suppress excessive, non-functional sprouting while promoting a more stabilized and orderly regeneration environment. These results support that WIPES facilitates structured neural recovery rather than chaotic axonal overgrowth. Notably, WIPES treatment normalized the GAP-43 Index to a moderate yet orderly

level while significantly increasing the PGP9.5 Area, suggesting that WIPES promoted structured axonal regeneration and functional reinnervation rather than chaotic overgrowth. This coordinated pattern implies that effective neuromuscular interface activation restores both nerve density and morphological integrity of regenerated fibers (Mean ± SD for N = 8 rats; ***P <0.001, **P < 0.01, *P < 0.05).

Location of Change: refer to the section of 'Neural Immunostaining & Quantification' in the Methods

Original Sentences: /

Revised Sentences: A new subsection has been added in the Methods to describe the procedures for Neural Immunostaining & Quantification, detailing the staining and analysis protocols used to assess peripheral nerve regeneration and neurotransmitter expression in urethral sphincter tissues. The section includes descriptions of immunofluorescence labeling for neuronal markers (PGP9.5, GAP-43) along with image acquisition and quantitative analysis methods used to evaluate nerve density and axonal regeneration. Representative fluorescence images and quantification results are provided in the main text figures to ensure methodological transparency and reproducibility.

==

8. While the device has a drastically reduced size, it is not the smallest wireless stimulation device available in the context of urinary incontinence, which needs to be acknowledged and discussed (see: doi.org/10.1016/j.ucl.2004.09.003, doi.org/10.1038/s41598-021-90088-8, doi.org/10.1002/nau.25477, doi.org/10.1007/s11255-024-04318-9, etc.). Overall, the discussion is incomplete, and the authors should include comparisons to other wireless stimulators reported in literature, and stronger evidence demonstrating a strong correlation between MUI and urethral incompetence. Furthermore, authors should state how their device would be superior to other non-invasive devices that stimulate the pelvic floor muscles and/or sphincter to reduce leaks.

Bosch et al. reviewed implantable microstimulators (e.g., BION) and sacral/pudendal neuromodulation for continence. These approaches offer clinically established neuromodulatory efficacy with targetable neural activation; however, long-term deployment typically entails implanted power sources and/or connected hardware (e.g., implantable pulse generators and leads), which introduce maintenance burdens such as battery replacement or recharging and occasional hardware-related revisions. WIPES is battery-free and lead-free, avoiding IPG pockets, recharging logistics, and long leads revisions. Its ultrathin, conformal patch sits directly on the sphincter/peri-urethral terminals, lowering activation thresholds and enabling dual action—outlet augmentation plus local afferent engagement.

==

This reference article is cited in [18] Bosch J. The bion device: a minimally invasive implantable ministimulator for pudendal nerve neuromodulation in patients with detrusor overactivity incontinence. Urologic Clinics, 32(1): 109-112 (2005).

==

Hernandez-Reynoso et al. presented a tiny, inductively powered perineal/pudendal nerve cuff that acutely boosts urethral pressure in rabbits. The device is genuinely small and shows clear immediate effects, yet cuff surgery and the largely acute evidence leave longer-term interface stability less certain. WIPES's soft wrap on the EUS avoids nerve-cuff constriction and foreign-body responses at the nerve while providing a broad, low-impedance contact. It is supported by chronic in-vivo data (urodynamics and neural/muscle histology) showing sustained efficacy rather than only acute pressure rises.

==

This reference article is cited in [28] Hernandez-Reynoso A G, Corona-Quintanilla D L, López-García K, et al. Targeted neuromodulation of pelvic floor nerves in aging and multiparous rabbits improves continence.

Scientific reports, 11(1): 10615 (2021).

==

Heesakkers et al. reported a miniature tibial-nerve passive implant (Revi / BlueWind) driven by a wearable, with strong UUI outcomes in humans. It is minimally invasive and clinically validated, but the distal target does not directly correct urethral outlet incompetence and requires adherence to external-wear sessions. WIPES's direct sphincter/peri-terminal placement addresses outlet resistance (SUI) and urge control (UUI) in the same local site, rather than modulating a distal conduit nerve. The waist-belt transmitter provides consistent geometric coupling around the pelvis for day-to-day, at-home therapy without implanted batteries or tunneled leads.

==

This reference article is cited in [19] Heesakkers J P F A, Toozs - Hobson P, Sutherland S E, et al. A prospective study to assess the effectiveness and safety of the BlueWind System in the treatment of patients diagnosed with urgency urinary incontinence. Neurourology and Urodynamics, 43(7): 1491-1503 (2024).

==

Long et al. (NuStim) described a 3×10 mm passive intramuscular stimulator energized by an RF seat that improves LPP in a rat SUI model. The implant is small and the SUI benefit is clear on LPP, though the seat-based driver and stress-only endpoints do not address urge physiology. WIPES's circumferential, ultrathin wrap around the EUS plus a waist-belt transmitter enables ambulatory, posture-tolerant use rather than seat-restricted sessions. It targets both components—SUI (LPP/EUS-EMG) and UUI (CMG/c-Fos)—within one local implant, coupling functional gains to mechanistic readouts.

==

This reference article is cited in [53] Long B, Li C, Deng H, et al. A potential therapeutic strategy using a miniature, implantable, wireless nerve stimulation device for treating stress urinary incontinence in rats. International Urology and Nephrology, 57(4): 1311-1318 (2025).

==

Minor comments

1. The manuscript is overall well written with minor instances where sentences are informal, and the authors should revise the manuscript to improve readability.

Thank you for the suggestion. We have thoroughly edited the manuscript to improve readability and formality: informal phrasing was revised, sentences were tightened, and grammar/usage corrected. Terminology and abbreviations were standardized at first use (e.g., WIPES, EUS-EMG, CMG, LPP), units were harmonized to SI, and figure/section cross-references were clarified.

2. The definitions of SUI and UUI are not entirely accurate. Please revise.

Thank you for the clarification request. We have revised the text to conform to International Continence Society (ICS) terminology and to distinguish symptom definitions from urodynamic findings:

Stress urinary incontinence (SUI) — symptom definition: the complaint of involuntary urine leakage on effort or physical exertion (e.g., exercise), or on sneezing or coughing. Urodynamic correlate: urodynamic stress incontinence (USI), i.e., demonstrable leakage coincident with increased abdominal pressure in the absence of a detrusor contraction, when intravesical pressure exceeds urethral closure pressure.

Urge urinary incontinence (UUI) — symptom definition: the complaint of involuntary urine leakage accompanied by or immediately preceded by urgency (a sudden compelling desire to void that is difficult to defer). Urodynamic correlate: typically associated with detrusor overactivity during bladder filling (phasic

rises in detrusor pressure, P_{det}), though DO is not part of the symptom definition per se.

Mixed urinary incontinence (MUI) — the complaint of both SUI and UUI.

==

Location of Change: Line 40

Original Sentences: Stress urinary incontinence (SUI) is due to damage of the urethral neuromuscular function that leads to uncontrollable relaxation of sphincters, while the other involves neural disorder induced irregular and involuntary contraction of the bladder muscles, known as urge urinary incontinence (UUI)

Revised Sentences: Stress urinary incontinence (SUI) results from impaired urethral neuromuscular control, causing urine leakage during physical exertion when urethral closure pressure fails to counter increased abdominal pressure. In contrast, urge urinary incontinence (UUI) arises from neural dysfunction leading to involuntary detrusor overactivity and sudden urgency before voiding.

==

3. Lines 60-61: Why did authors decide to cite an abstract (as opposed to a peer-reviewed article) that included patients with fecal incontinence? There is plenty of literature on the therapeutic effectiveness of SNM. Furthermore, it is unclear where 48% effectiveness is reported in the cited reference.

We appreciate the reviewer's observation. The citation on lines 60–61 has been corrected: we now cite the peer-reviewed ELITE study of InterStim™ Micro in OAB patients [18] and a contemporary clinical review of sacral neuromodulation (SNM) [19], and we have removed the abstract that mixed fecal incontinence.

[20] Goudelocke, C. et al. Evaluation of clinical performance and safety for the rechargeable InterStim Micro device in overactive bladder subjects: 6-month results from the global postmarket ELITE study. Neurourology and Urodynamics, 42(4), 761-769 (2023).

[21] Mass-Lindenbaum, M., Calderón-Pollak, D., Goldman, H. B., & Pizarro-Berdichevsky. Sacral neuromodulation - when and for who. International braz j urol : official journal of the Brazilian Society of Urology, 47(3), 647–656 (2021).

To avoid ambiguity around ‘effectiveness = 48%,’ we revised the text to report endpoint-specific outcomes using the strict ‘complete-dry (complete cure)’ endpoint, SNM achieves ~46-47% complete dryness at 6–36 months; using the conventional responder endpoint (≥50% reduction in UUI episodes), rates are typically ~76% across studies.

==

Location of Change: Line 957

Original Sentences: [18] Elterman, D. et al. INTERSTIMTM MICRO IMPROVES SYMPTOMS OF FECAL INCONTINENCE 660 AT 3-MONTH FOLLOW UP IN A GLOBAL, PROSPECTIVE, POST-MARKET STUDY. Continence, 7, 661 100839 (2023).

Revised Sentences: [20] Goudelocke, C. et al. Evaluation of clinical performance and safety for the rechargeable InterStim Micro device in overactive bladder subjects: 6-month results from the global postmarket ELITE study. Neurourology and Urodynamics, 42(4), 761-769 (2023).

[21] Mass-Lindenbaum, M., Calderón-Pollak, D., Goldman, H. B., & Pizarro-Berdichevsky. Sacral neuromodulation - when and for who. International braz j urol : official journal of the Brazilian Society of Urology, 47(3), 647–656 (2021).

Location of Change: Line 61

Original Sentences: The state-of-the-art commercial electrostimulation implant, such as the InterStimTM Micro, feature a bulky volume of 2.8 cm3, a heavy weight of 13 grams, a high price between $20,000–30,000, and maintain a therapeutic effectiveness of only 48%.

Revised Sentences: The state-of-the-art commercial electrostimulation implant, such as the InterStimTM Micro, feature a bulky volume of 2.8 cm3, a heavy weight of 13 grams, a high price between $20,000–30,000. They maintain a therapeutic effectiveness of ~46-47% complete-dry rates at 6-36 months (a conservative endpoint), while the responder rates are typically ~76% for a reduction over 50% (Table S1 for additional commercial device comparisons).

==

4. Lines 67-69: List of diagnostic steps should include urodynamic tests as well.

We appreciate the reviewer's helpful suggestion. In accordance with the comment, we have updated the description of the diagnostic process for urinary incontinence to include urodynamic evaluation as an essential step in current clinical practice.

==

Location of Change: Line 72

Original Sentences: As a result, patients may undergo an extensive list of steps to diagnose urinary incontinence including behavior questionnaire, bladder stress test, catheterization, urine culture and ultrasound.

Revised Sentences: As a result, patients may undergo an extensive list of steps to diagnose urinary incontinence including behavior questionnaire, bladder stress test, catheterization, urine culture, urodynamic evaluation and ultrasound.

==

5. References should be reviewed, as some have nothing to do with the sentences stated.

Thank you for noting this. All references have been reviewed and corrected. Mismatched citations have been removed/replaced.

6. In line 111, authors state that the wires are conductive only where it contacts the urethral sphincter, but it is unclear how the sizing is achieved.

We agree that the sizing and contact geometry needed clarification. The stimulator is a flexible gold patch looks like an open annulus ('C-shaped' wrap) (projected area ≈ 0.8 cm^2, Fig. 1c and schematic above). The inner face of the C-ring is the only exposed conductor. The outer face and the connecting gold wire are fully encapsulated, so tissue contact occurs only at the intended urethral interface. About surgical placement. Under perineal exposure, EUS is identified and gently mobilized in the submucosal plane, taking care not to injure the urethral mucosa or neurovascular bundle. A blunt micro-dissector is passed behind the EUS to create a short tunnel, through which the C-shaped patch is introduced with the open side lateral. The strip is then wrapped around the EUS so that the gold inner face lies flush on the muscle. Light apposition is used—no constrictive tension. The patch is tacked to adjacent fascia at two points using a small drop of fibrin sealant to prevent rotation. The insulated interconnect is then tunneled superficially to the planned position, and the WIPES main body (flexible module) is laid flat in the subcutaneous plane—between the skin and the muscle fascia. The fascia is sutured over the wrapped patch. Representative intraoperative photographs of the wrapped configuration are provided in Fig. S27.

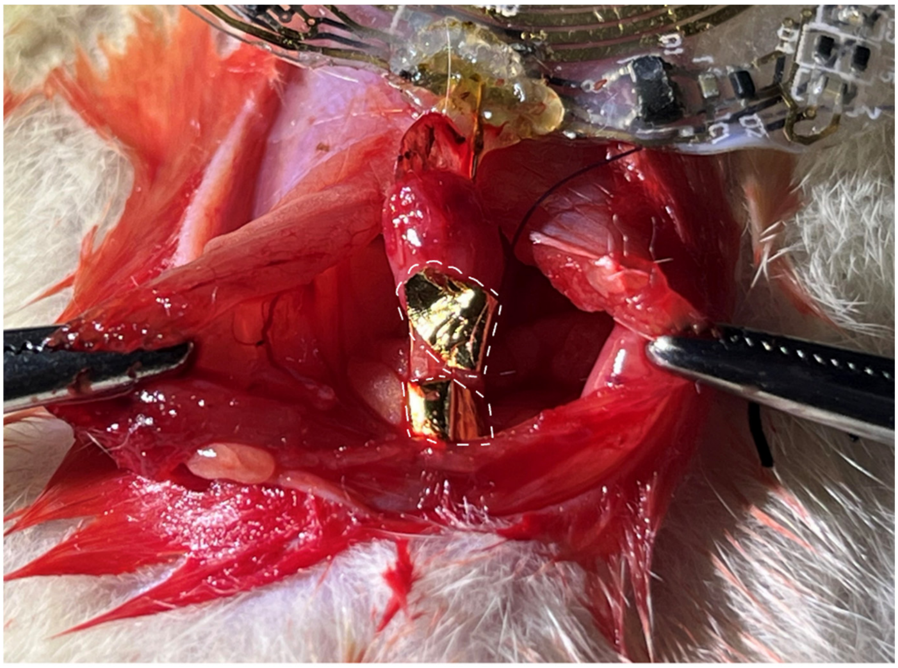

Fig. S27. Intraoperative view of WIPES electrode wrapping the external urethral sphincter (EUS).

==

Location of Change: Line 115

Original Sentences: The wires are specially designed to wrap around the sphincter with curved patches ensuring stability while avoiding squeezing on it.

Revised Sentences: The electrodes are designed as flexible C-shaped gold patches that wrap gently around the sphincter, ensuring stable contact without exerting constrictive pressure (Fig. S27). The inner surface serves as the active interface, while the outer surface and connecting leads are fully encapsulated to maintain insulation.

==

7. For figure 2h, please provide the methodology of how these measurements were obtained? Was this measured in saline? What is simulated with a resistor?

Figure 2h is an analytical load-line of the constant-current stimulator, not a saline measurement. The curves were generated from the dual-op-amp driver equations with a reference rail $V_{ref} = 3.3\ \mathrm{V}$ and compliance $V_{comp} \approx 12\ \mathrm{V}$; the sense resistor RS sets the transconductance. For a given RS, the peak pulse current is

$$I_{set} = \frac{V_{ref}}{2RS}$$

within the compliance range, and transitions to a compliance-limited regime when the required output voltage exceeds V_{comp}. The knee occurs at

$$R_{\mathrm{knee}} = \frac{V_{\mathrm{comp}}}{I_{\mathrm{set}}} - RS = RS\left(\frac{2V_{\mathrm{comp}}}{V_{\mathrm{ref}}} - 1\right).$$

With the RS values used in Fig. 2h (200, 400, 800, 1000 Ω), this yields flat currents of ≈8.25, 4.13, 2.06, 1.65 mA, respectively, across the tissue-relevant load range (100–1200 Ω); only near the upper end of that range does the RS=200 Ω trace approach the compliance knee.

The “load resistance” on the x-axis is a resistive surrogate for tissue impedance magnitude at 60 Hz, where the electrode–tissue interface behaves predominantly resistively for our pulse width. The shaded

bands indicate typical impedance ranges for the listed tissues (myocardium, Skeletal muscle, Urethral sphincter, Smooth muscle); the resistor in the model therefore simulates the magnitude of the tissue load seen by the stimulator during a pulse.

8. In panel a of Figure 3, device placement into culture is named "implantation" but this term is incorrect.

Agreed. We have replaced “implantation” in Fig. 3a with “ex vivo culture” (device–tissue construct in culture) and updated the figure label, caption, and corresponding text results.

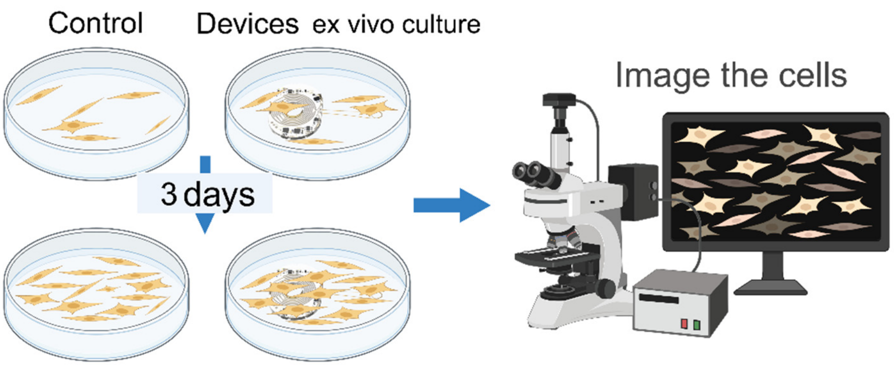

Fig. 3a. Schematic diagram of the experimental setup for assessing biocompatibility with SV-HUC-1 cells for a 3-day co-culture.

Reviewer 4:

General comments:

The study looked at electro stimulation to rat's urinary sphincter to see if it is good as a novel tool to treat urinary incontinence induced by CTX. The hypothesis may be wrong. Neuromodulation is about stimulation of the other peripheral nerves to modulate neurological performance of specific function, not direct stimulation on the specific muscles responsible for function. The study method did not describe details in identifying sphincter, responses of urinary bladder. Increase of sphincter function will have effects on detrusor. Changes of CTX cystitis is also important for readers and should be reported. Insertion of a catheter into male rats' urethra is difficult and painful. How did the author overcome this issue should be reported and animal research committee should provide agreement on this issue.

We appreciate your comments on the hypothesis. We are sorry that the previous version is not clear enough on our hypothesis, nor on the obtained experimental evidence. We agree with you that typical neuromodulation is not direct stimulation on the specific muscles responsible for function. As a result, we don't refer to our WIPES approach as a neuromodulation method, and more important, the WIPES approach proposed in this work shows more superior results than existing peripheral nerve neuromodulation for incontinence, which is a novel and interesting conclusion to communicate.

I. Conceptual clarification: WIPES as a terminal neuromuscular interface

"The hypothesis may be wrong. Neuromodulation is about stimulation of the other peripheral nerves to modulate neurological performance of specific function, not direct stimulation on the specific muscles responsible for function."

We clarify in the revised manuscript that WIPES is not intended as a conventional neuromodulation approach. Instead, WIPES is designed as a terminal neuromuscular interface. WIPES is a soft, wrap-around stimulator placed directly on the external urethral sphincter (EUS), enabling two complementary actions: (i) augmentation of local myogenic closure, which is directly relevant to stress urinary incontinence (SUI), and (ii) engagement of peri-urethral afferent pathways to influence the bladder–sphincter reflex, which is pertinent to urge urinary incontinence (UUI) in the cyclophosphamide (CTX) cystitis model. This dual-action design underpins the rationale for evaluating WIPES across both UUI- and SUI-related pathophysiology.

II. Expanded multi-level validation chain supporting therapeutic efficacy

"The study method did not describe details in identifying sphincter, responses of urinary bladder."

To substantiate this mechanism, we have added and reorganized a coherent, multi-level validation chain. Urinary-sand behavior is included as a secondary functional readout. Urodynamic assessments demonstrate consistent improvements, including reduced non-voiding contraction (NVC) burden, prolonged intercontraction interval (ICI), increased bladder capacity, and enhanced guarding and voiding bursts. Specifically, cystometry (CMG) is used for UUI assessment, while EUS-EMG and leak point pressure (LPP) measurements are used for SUI evaluation. In parallel, neural histology and molecular markers (including c-Fos with nNOS/VIP profiling) provide evidence for reflex-level engagement at the bladder–sphincter axis.

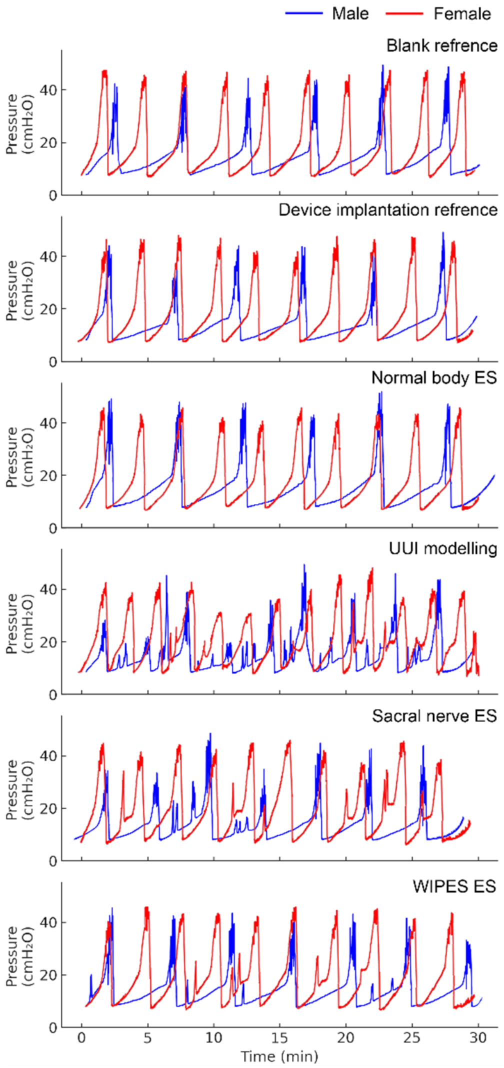

Fig. 5a. Representative cystometric traces of male and female rats under different conditions for 6 groups.

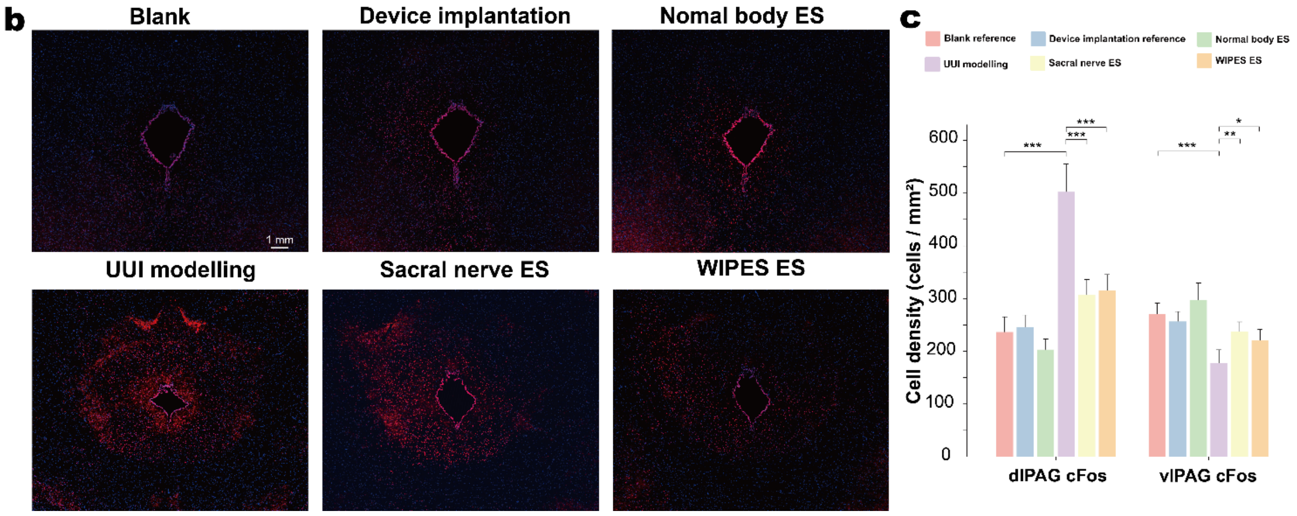

Fig. 5b. c-Fos immunostaining images of the dorsal and ventrolateral periaqueductal gray (dPAG and vlPAG) regions in rats from each group. 5c. Statistical analysis of c-Fos–positive cell densities in dPAG and vlPAG regions.

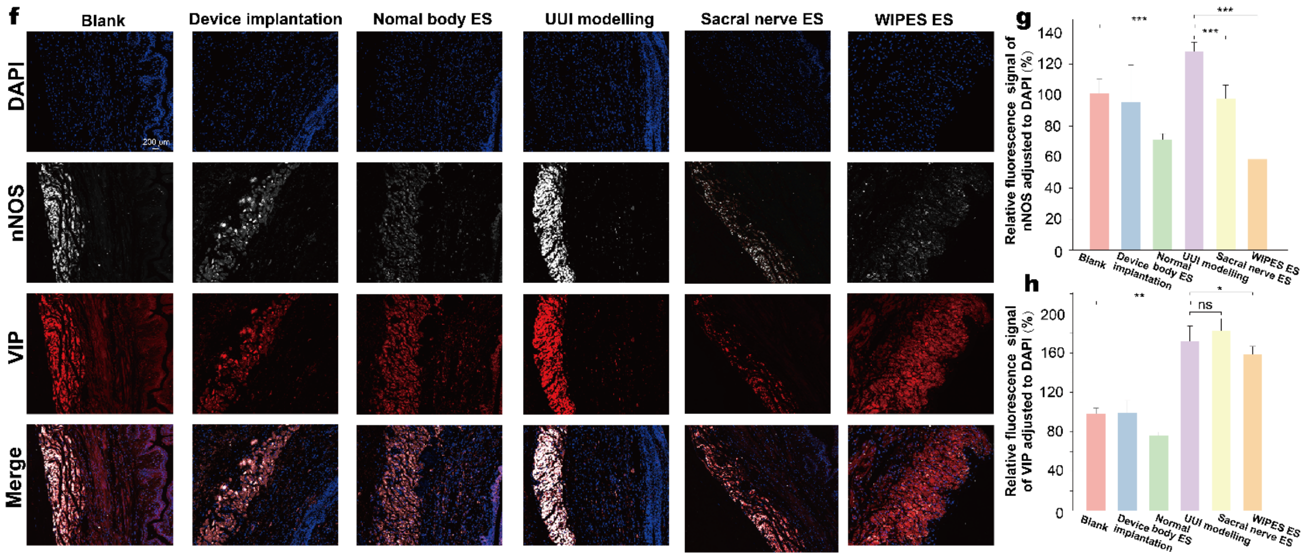

Fig. 5f. Immunofluorescence staining of nNOS and VIP neurotransmitters in urethral sphincter tissues of rats in each group. 5g. Quantitative comparison of nNOS fluorescence intensity normalized to DAPI for all groups. 5h. Quantitative comparison of VIP fluorescence intensity normalized to DAPI for all groups.

==

Location of Change: Line 310

Original Sentences: /

Revised Sentences: The cystometric analysis (Fig. 5a) provides quantitative assessment of bladder activity by measuring intravesical pressure changes over time (refer to the section of ‘Urodynamic Evaluation’ in the Methods, and Fig. S28). Three key parameters—non-voiding contraction (NVC) frequency, inter-contraction interval (ICI), and bladder capacity—were analyzed to characterize detrusor muscle function. Rats in the UUI modelling group exhibited abnormally frequent NVCs and markedly shortened ICIs, indicating excessive and unstable detrusor activity associated with urge incontinence. The reduced bladder capacity further confirmed the loss of coordinated neural control and premature triggering of voiding reflexes. In contrast, WIPES electrostimulation (WIPES ES) effectively normalized these parameters. The frequency of NVCs was significantly reduced, while the ICI was prolonged to levels comparable to healthy controls, reflecting restored detrusor stability and improved bladder compliance. Meanwhile, the measured bladder capacity increased markedly, suggesting that the WIPES stimulation enabled fuller bladder filling before the initiation of micturition, indicative of improved neural regulation of the micturition reflex. The SNS group also showed comparable improvement, with slightly more irregular NVCs.

Location of Change: Line 321

Original Sentences: /

Revised Sentences: To further investigate how WIPES modulates central neural circuits involved in micturition control, c-Fos immunofluorescence staining was performed in the dorsal periaqueductal gray (dlPAG) and ventrolateral periaqueductal gray (vlPAG) regions of the midbrain (Figs. 5b and 5c) (refer to the section of 'Quantification of c-Fos for urge-suppression assessment' in the Methods). The PAG is a pivotal integrative hub linking bladder sensory input and pontine motor output. Specifically, the dlPAG primarily participates in urine storage and inhibitory control, suppressing the activation of the micturition reflex, whereas the vlPAG is associated with voiding facilitation, relaying excitatory signals to the pontine micturition center (PMC). Thus, the spatial distribution and intensity of c-Fos expression in these two regions provide insight into the excitatory–inhibitory balance within the central micturition network. In the

UUI modelling group (4th), excessive bladder afferent input caused by detrusor overactivity aberrantly activated the dlPAG, leading to an overcompensated inhibitory response attempting to suppress involuntary voiding. Quantitatively, c-Fos expression in the dlPAG increased by 112.81% compared to the blank reference (1st), confirming pronounced hyperactivation of this inhibitory center. In contrast, the vlPAG showed a 34.5% reduction in c-Fos activity, indicating suppression of the voiding-facilitatory pathway and disruption of the normal excitatory relay to the PMC. Such imbalance between facilitation and inhibition underlies the detrusor overactivity characteristic of urge incontinence. Following WIPES ES group (6th), this imbalance was markedly corrected. c-Fos expression in the dlPAG decreased by 37.16%, while the vlPAG exhibited a 33.77% increase, indicating a bidirectional normalization of inhibitory and excitatory activities within the central micturition network. Similarly, SNS ES group (5th) produced comparable effects, with dlPAG activity reduced by 38.86% and vlPAG elevated by 33.78%. These results suggest that both WIPES and SNS treatments effectively rebalanced the dlPAG–vlPAG circuitry, and from the perspective of impulse inhibition, their restorative efficacy showed no significant difference. (Mean ± SD for N = 8 rats; ****P* <0.001, ***P* < 0.01, **P* < 0.05).

Location of Change: Line 345

Original Sentences: Furthermore, two markers, nNOS and VIP, were selected to evaluate the growth and function of nerve cells around the urethra. nNOS is a neurotransmitter in urethral nerve cells that induces smooth muscle relaxation and mediates nerve injury and inflammatory pain hypersensitivity, while VIP reduces neuroinflammation and promotes early myelination and growth of regenerative axons in the nervous system (Fig. 5b). Immunofluorescence staining showed that, compared to the Blank reference group (1st), the relative fluorescence intensity of nNOS and VIP proteins was significantly increased in the Incontinence modelling group (4th), with increases of 31.63% (nNOS) and 23.97% (VIP) (Mean ± SD for N = 4 rats; ****P* <0.001,***P* < 0.01), suggesting that induced urinary incontinence caused inflammatory nerve damage and pain hypersensitivity around the urethra Interestingly, electrostimulation reduced nNOS signals in both the Normal body ES group (3rd), with a decrease of 30.14%, and the UI & ES group (5th), with a decrease of 57.37% (Mean ± SD for N = 4 rats; **P* < 0.05, ****P* < 0.001) while maintaining VIP levels (Mean ± SD for N = 4 rats; ns, not significant) as shown in Fig. 5f. This reduction suppressed neural pain hypersensitivity, creating a microenvironment conducive to nerve axon regeneration to facilitate recovery from UUI. These combined findings suggest that by promoting smooth muscle contraction via ROCK2 activation to enhance urethral closure, and modulating neural function via nNOS to support nerve regeneration and reduce inflammation-induced dysfunction, WIPES could effectively contribute to the treatment of MUI.

Revised Sentences: Furthermore, elucidate the neuroregulatory mechanisms responsible for the functional recovery observed in cystometric profiles, we examined two markers, nNOS and VIP, were selected to evaluate the growth and function of nerve cells around the urethra (refer to the section of ' Immunofluorescence Staining ' in the Methods). nNOS is a neurotransmitter in urethral nerve cells that induces smooth muscle relaxation and mediates nerve injury and inflammatory pain hypersensitivity , while VIP reduces neuroinflammation and promotes early myelination and growth of regenerative axons in the nervous system (Fig. 5f and Fig. 5h). In Blank reference (1st), nNOS and VIP were distinctly expressed in organized nerve fibers surrounding the urethral sphincter and bladder wall, ensuring synchronized activation of excitatory and inhibitory pathways that maintain stable micturition cycles. In contrast, rats subjected to UUI modelling (4th) exhibited diffuse and disordered nNOS overexpression, accompanied by irregular VIP distribution and reduced spatial colocalization, with increases of 26.73% (nNOS) and 75.13% (VIP) (Mean ± SD for N = 8 rats; ****P* <0.001, ***P* < 0.01). Such aberrant patterns indicate a breakdown in inhibitory control and overactivation of parasympathetic efferent, consistent with the frequent, high-amplitude NVCs observed in cystometric recordings. Following SNS ES (5th) and WIPES ES (6th)

electrostimulations, nNOS signals recovered toward physiologically balanced patterns, with a decrease of 23.67% and 54.30% , VIP levels have different expressions, with increase a little (Mean ± SD for N = 8 rats; ns, not significant) and a decrease of 8.03%. The normalized nNOS distribution implies restored nitric oxide–mediated inhibitory tone, preventing premature detrusor contractions, while the orderly re-expression of VIP indicates re-established neurovascular coupling and mucosal relaxation signaling. These features collectively reflect the functional reconnection of afferent–efferent feedback loops rebuilt through WIPES-induced peripheral reinnervation. By contrast, SNS treatment led to partial normalization, with residual nNOS hyperexpression and sparse VIP regions, suggesting incomplete restoration of inhibitory control.

Location of Change: refer to the section of 'Quantification of c-Fos for urge-suppression assessment' in the Methods

Original Sentences: /

Revised Sentences: A new subsection has been added in the Methods to describe the procedures for Quantification of c-Fos for urge-suppression assessment, outlining the experimental workflow and analytical criteria used to evaluate neuronal activation in brain regions associated with micturition control. The section details tissue preparation, immunofluorescence staining, imaging, and quantification of c-Fos–positive nuclei within the dorsolateral (dlPAG) and ventrolateral periaqueductal gray (vlPAG) regions. Sampling and image analysis were performed under blinded conditions, and standardized thresholds were applied across all groups to ensure consistency and reproducibility in assessing central neural activity related to urge suppression.

Location of Change: refer to the section of 'Neural Immunostaining & Quantification' in the Methods

Original Sentences: /

Revised Sentences: A new subsection has been added in the Methods to describe the procedures for Neural Immunostaining & Quantification, detailing the staining and analysis protocols used to assess peripheral nerve regeneration and neurotransmitter expression in urethral sphincter tissues. The section includes descriptions of immunofluorescence labeling for neuronal markers (PGP9.5, GAP-43) along with image acquisition and quantitative analysis methods used to evaluate nerve density and axonal regeneration. Representative fluorescence images and quantification results are provided in the main text figures to ensure methodological transparency and reproducibility.

==

III. Methodological clarification of EUS targeting and implantation

"The study method did not describe details in identifying sphincter."

We have expanded the Methods section to provide detailed clarification of EUS identification and targeting. This includes anatomical exposure at the membranous urethra, visual confirmation of circumferential placement of the stimulator, and functional verification via evoked EUS-EMG guarding bursts at low stimulation thresholds. To further enhance transparency, intraoperative photographs are now provided.

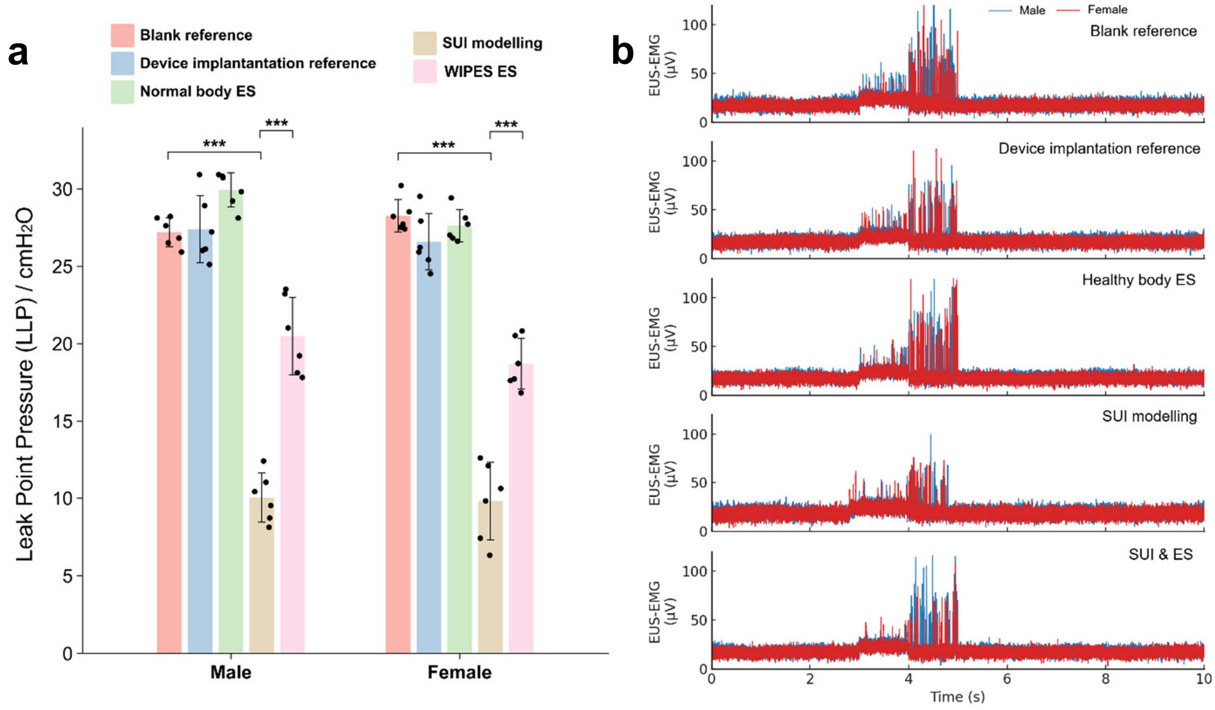

Fig. 6a. Leak point pressure results for five groups, showing significant LPP recovery after WIPES stimulation in both male and female rats. 6b. Representative external urethral sphincter electromyography (EUS-EMG) recordings of all groups

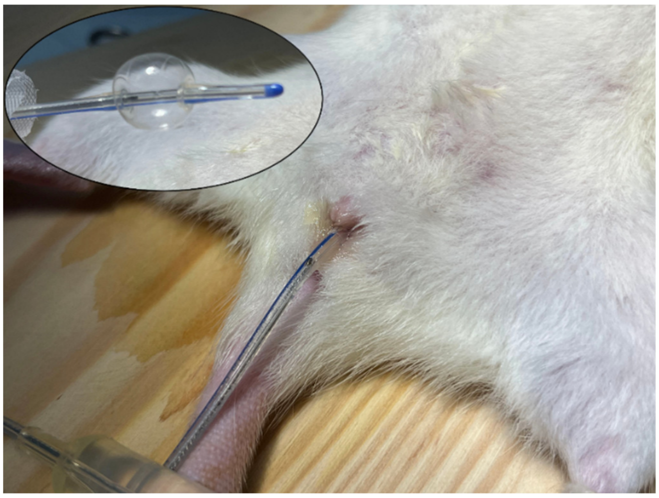

Fig. S23. Urethral balloon dilatation for establishing the SUI model in female rats.

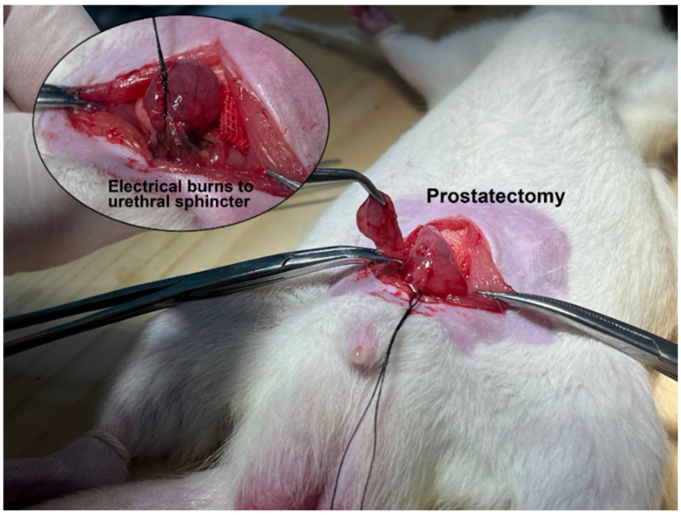

Fig. S24. Intraoperative procedure of combined SUI model induction in male rats through prostatectomy and electrical burns to the urethral sphincter and associated nerves.

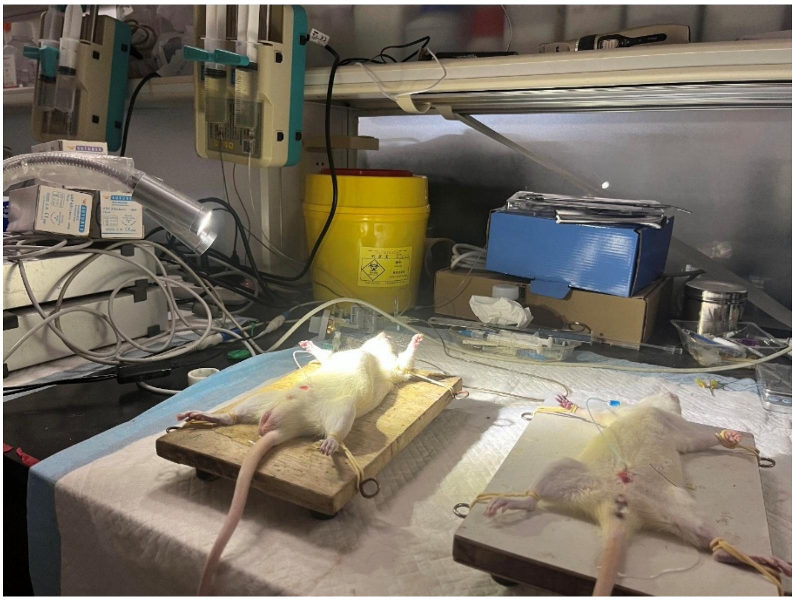

Fig. S28. Experimental setup for cystometry in male and female rats.

===

Location of Change: Line 414

Original Sentences: /

Revised Sentences: Cystometric measurements and leak point pressure (LPP) testing (Fig. 6a; refer to the section of 'Urodynamic Evaluation' in the Methods) revealed a pronounced reduction in LPP in rats subjected to SUI modelling (4th) compared with the Blank group (1st), with a decrease of 64.24%, indicative of compromised urethral closure competence and weakened sphincteric resistance to bladder pressure. The decreased LPP reflects impaired transmission of contractile force within the external urethral sphincter (EUS), resulting in premature urine leakage upon elevated intravesical pressure. Following WIPES ES group (5th), both male and female rats exhibited a substantial recovery of LPP values, with an increase of 97.90%, approaching those of the healthy baseline, demonstrating the efficacy of electrical stimulation in restoring urethral outlet resistance (Mean ± SD for N = 8 rats. ***P < 0.001).

Location of Change: Line 419

Original Sentences: /

Revised Sentences: Concurrently, electromyographic recordings from the EUS (EUS-EMG, Fig. 6b) revealed characteristic alterations in neuromuscular activity patterns. In SUI-modelled rats, the EMG traces showed sparse, low-amplitude, and poorly coordinated bursts during voiding, reflecting disrupted neuromuscular communication and reduced sphincter excitability. In contrast, WIPES-treated animals exhibited rhythmic, high-frequency spike bursts synchronized with the micturition cycle, indicative of reinstated reflex control and functional re-engagement of motor units within the sphincter. These findings collectively confirm that WIPES stimulation not only enhances urethral closure pressure mechanically, as evidenced by LPP recovery, but also re-establishes physiological neuromuscular coordination essential for continence regulation.

Location of Change: refer to the section of 'Urodynamic Evaluation' in the Methods and Fig. S23-24

Original Sentences: /

Revised Sentences: A new subsection has been added in the Methods to describe the procedures for Urodynamic Evaluation, including the experimental setup and measurement protocols used for assessing bladder and sphincter function in both UUI and SUI models. The section details CMG for evaluating detrusor activity, LPP testing for quantifying urethral closure pressure, and EUS-EMG for monitoring neuromuscular coordination during voiding. The experimental setup for cystometry in male and female rats is illustrated in Fig. S28 to ensure methodological clarity and reproducibility.

===

IV. Detrusor-sphincter interplay and CTX cystitis characterization

"Increase of sphincter function will have effects on detrusor. Changes of CTX cystitis is also important for readers and should be reported."

To address detrusor-sphincter interactions, we quantified P_{det} metrics from CMG, including baseline P_{det}, NVC amplitude and frequency, peak voiding pressure, and bladder capacity. These data document the bladder's response under stimulation and do not indicate pathological detrusor hyperactivity. We further characterize CTX-induced cystitis using bladder histology and inflammation scoring (H&E staining; edema, hemorrhage, and inflammatory infiltrate indices; Fig. S30) to document the disease state.

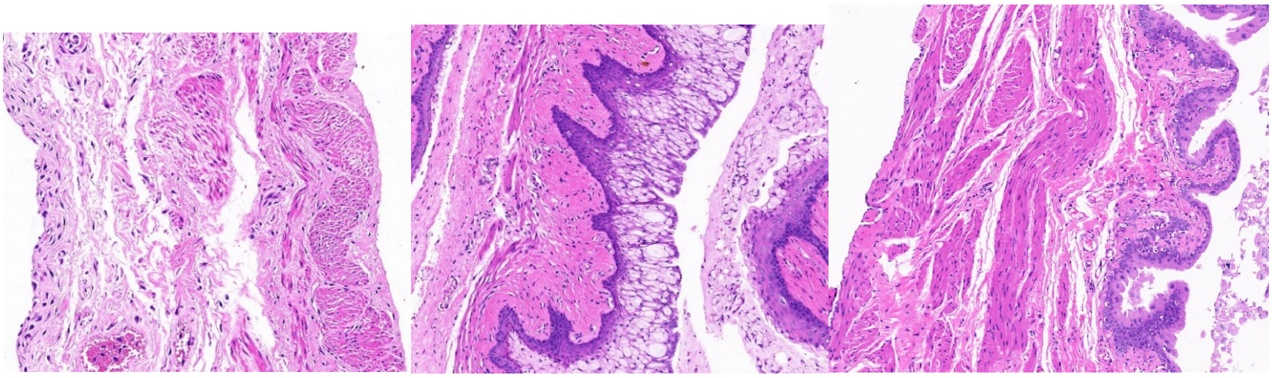

Fig. S30. Blank reference, CTX modeling, and CTX+ES groups' HE staining sections of bladder tissues.

V. Catheterization procedures and animal welfare considerations

"Insertion of a catheter into male rats' urethra is difficult and painful. How did the author overcome this issue should be reported and animal research committee should provide agreement on this issue."

We appreciate this important concern regarding animal welfare. In the revised manuscript, we clarify that

no male transurethral catheterization was performed (Fig. 3e and Fig. 3f). A short transurethral catheter was used only in female rats, using a lubricated, soft PE-5 silicone micro-catheter inserted under brief anesthesia.

For urodynamic assessments, CMG was performed via suprapubic bladder-dome catheterization in both sexes. Leak point pressure (LPP) was assessed using a vaginal balloon in females and an intrarectal balloon in males. All procedures were conducted under appropriate anesthesia and analgesia and were approved by Ruijin Hospital’s Application Format for Ethical Approval for Research Involving Animals.

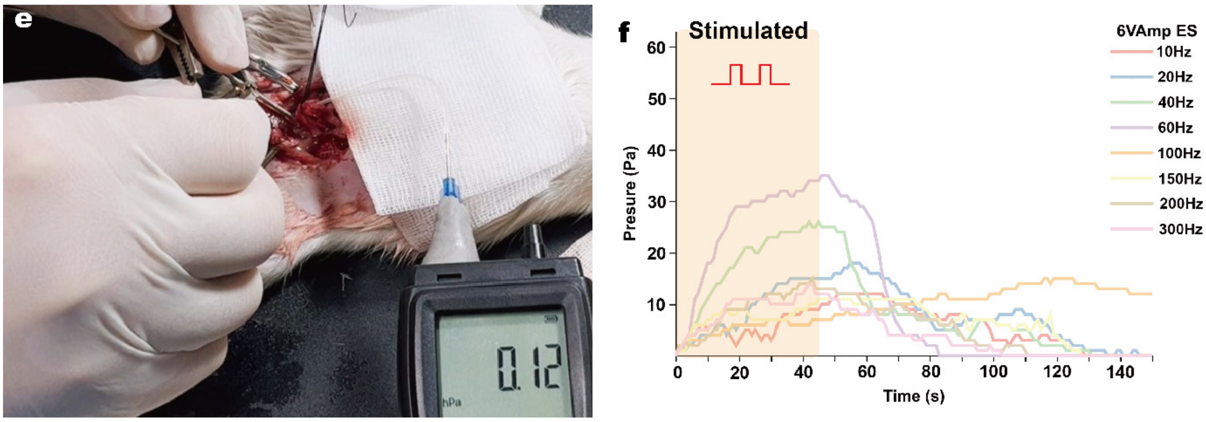

Fig. 3e. Stimulation setup using monophasic pulses by a function generator. 3f. Pressure response of urethral sphincter when stimulated by different frequencies of monophasic pulses.

Summary

Together, these revisions clarify the conceptual framework of WIPES, strengthen the linkage between sphincter-level intervention and bladder-level function, provide detailed methodological descriptions, address detrusor and disease-state concerns, and explicitly document catheterization strategies and ethical approvals.

Specific comments

Line 51. UUI is also common in women.

Noted. We revised line 52 to state explicitly that UUI is also common in women.

==

Location of Change: Line 52

Original Sentences: On the other hand, UUI occurs more frequently in men and is more complex to tackle.

Revised Sentences: On the other hand, UUI occurs both frequently in men and women which is more complex to tackle.

==

Line 81. direct stimulation at the nerves and smooth muscles of the urethral sphincter.

The urethral sphincter is a muscular structure that regulates the flow of urine from the bladder. It consists of two sphincters: the internal urethral sphincter and the external urethral sphincter. The internal sphincter is made of smooth muscle and is under involuntary control, while the external sphincter is made of skeletal muscle and is under voluntary control. Please correct it and specify WIPES stimulate internal or external sphincter. CTX related incontinence is not related to SUI because we did not change pelvic floor and

urethral function. Otherwise, the authors should provide reference for this statement.

Thank you for the request. We have clarified that WIPES delivers programmed pulses via a conformal gold patch wrapped on the external urethral sphincter (EUS, striated muscle); the resulting field also engages peri-urethral afferents, and any incidental activation of the internal urethral sphincter (smooth muscle) is secondary to current spread through the urethral wall. This replaces the previous phrasing "direct stimulation at the nerves and smooth muscles of the urethral sphincter," which was imprecise. Our device is placed on the EUS, not the internal sphincter. The outer surfaces and lead are insulated, so the intended contact is the EUS, with expected field penetration peri-urethral nerve plexus.

To avoid conflating disease entities, UUI and SUI are modeled and validated separately. UUI is induced by cyclophosphamide (CTX) cystitis and evaluated under fixed-rate CMG to quantify capacity, inter-contraction interval and non-voiding contractions at defined filling volumes. Mechanistic readouts include c-Fos mapping in the periaqueductal gray (dlPAG and vlPAG) and nNOS/VIP profiling in peri-urethral tissues, alongside bladder histopathology. SUI is modeled independently (female vaginal distension; male sphincter injury), and validated with LLP (female vaginal balloon; male intrarectal balloon at a standardized intravesical volume) and EUS-EMG. Complementary tissue analyses focus on the sphincter/neuromuscular unit: HE/Masson/TUNEL for muscle thickness and collagen fraction, α-SMA/ROCK2 for myogenic remodeling, PGP9.5, GAP-43, etc. Gene/protein changes are corroborated by quantitative expression assays in the respective cohorts. This two-cohort strategy-UUI (CTX) with CMG plus neural markers, and SUI with LPP/EUS-EMG plus neuromuscular histology-aligns endpoints with pathophysiology and specifies that WIPES stimulates EUS directly while engaging peri-urethral afferents that can influence bladder-sphincter reflexes.

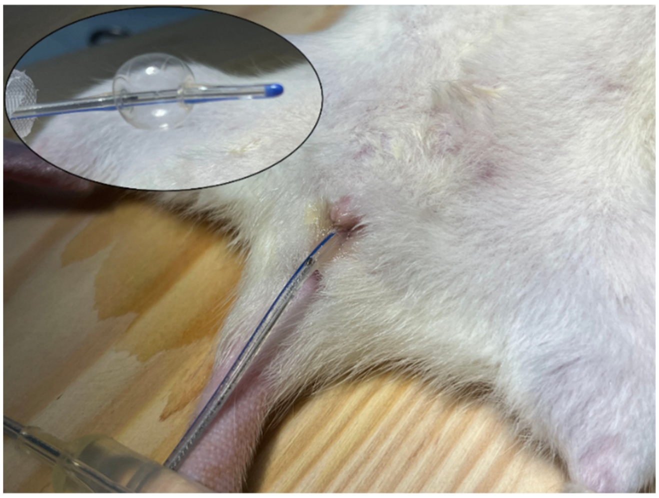

Fig. S23. Urethral balloon dilatation for establishing the SUI model in female rats.

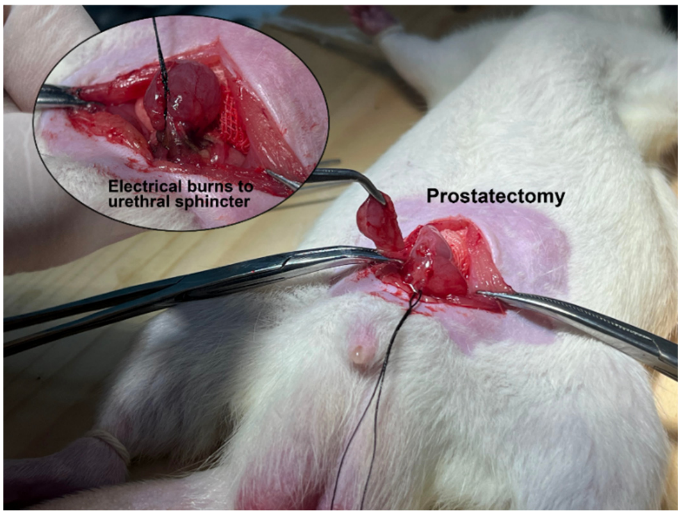

Fig. S24. Intraoperative procedure of combined SUI model induction in male rats through prostatectomy and electrical burns to the urethral sphincter and associated nerves.

==

Location of Change: refer to the section of 'Rat models of urgency urinary incontinence' in the Methods

Original Sentences: /

Revised Sentences: A new paragraph has been added in the Methods section to describe the procedures for establishing the rat model of urgency urinary incontinence (UUI), including model induction, validation, and postoperative management. The added description provides sufficient methodological detail to ensure reproducibility.

Location of Change: refer to the section of 'Rat models of stress urinary incontinence'' in the Methods and Fig. S23-24

Original Sentences: /

Revised Sentences: A new subsection has been added in the Methods to describe the procedures for establishing the rat model of stress urinary incontinence (SUI), including the modeling technique, validation process, and postoperative evaluation. Corresponding schematic illustrations and representative validation results have been included in Figs. S23–S24 to support reproducibility and clarify the experimental setup.

==

Methods:

Regarding fig S9

Q: Chamber pressure is typically measured using a balloon infused with saline, and compared to the balloon, the compressive force of a silicone tube is relatively lower and does not contract to generate pressure. Additionally, the pressure sensor used by the author is not a conventional physiological method.

We appreciate the concern and have clarified the intent and methodology of Fig. S9. Because the rat urethra is very narrow and no suitable mini balloon catheter is available, we used an ultra-fine, single-ended flexible silicone micro-tube coupled to a pneumatic differential manometer to read out pressure during EUS stimulation (reference to 'Sphincter Pressure Measurement During Electrostimulation'). This setup measures the air pressure rise inside the tube produced by external radial compression from the contracting EUS—i.e., a lateral "squeeze pressure" at the urethral wall, not bladder (intravesical) or P_{det}. The signal comprises the tube's elastic preload plus the stimulus-evoked increment. We provide a

calibration formula and report the EUS squeeze pressure after subtracting the preload (Fig. 3f, Fig. 3h). This assay is purposefully demonstrative—to show direct contractile force generation with stimulation—and is not presented as a conventional urodynamic endpoint.

Additionally, all physiological endpoints were obtained with standard methods that do not require urethral catheterization: CMG via suprapubic dome catheter (both sexes), LPP with a vaginal balloon in females and an intrarectal balloon in males at a standardized intravesical volume and EUS-EMG via perineal fine-wire electrodes. We have revised the Supplement to state explicitly that Fig. S9 reflects a localized EUS compression readout and to distinguish it from the conventional urodynamic measures reported elsewhere in the manuscript.

Regarding F4. 263 (f) Number of urinary sand masses indicates urination frequency. Each dot represents one measurement while each column represents the mean and the error bars indicate +/- one standard deviation. Statistics of Incontinence modelling and UI & ES groups for females show *$P < 0.05$ with $N = 4$ rats (data for male showing statistically not significant with $N = 4$ rats).

Q. The author states that electrical stimulation was performed through two flexible wires fixed on the urethral sphincter, with the urethral sphincter being the primary stimulation site. Figure 4f shows that the effect of ES is significantly better in females than in males. Since the female urethra lacks a distinct striated muscle structure, how does the author ensure that the electrodes do not pierce the urethra?

We appreciate the concern. In rats, including females, the external urethral sphincter (EUS) is a distinct circumferential striated (skeletal) muscle (rhabdosphincter) encasing the mid-urethra. Our device is not a penetrating needle/wire: it is a soft, C-shaped gold patch whose inner gold face lies on the EUS surface while the outer surface/lead are insulated. Placement is by blunt perineal dissection and wrap-around apposition—no transurethral instrumentation and no tissue piercing are performed. Mucosal integrity was verified intraoperatively (saline patency test) and postoperatively (no hematuria/obstruction), and histology from endpoint specimens shows an intact urothelium beneath the implant. Thus, the female efficacy observed in Fig. 4f is not attributable to urethral puncture. Rather, it reflects stimulation at the striated EUS and peri-urethral afferents in a sex-specific model, with no penetration of the urethral wall by the electrode.

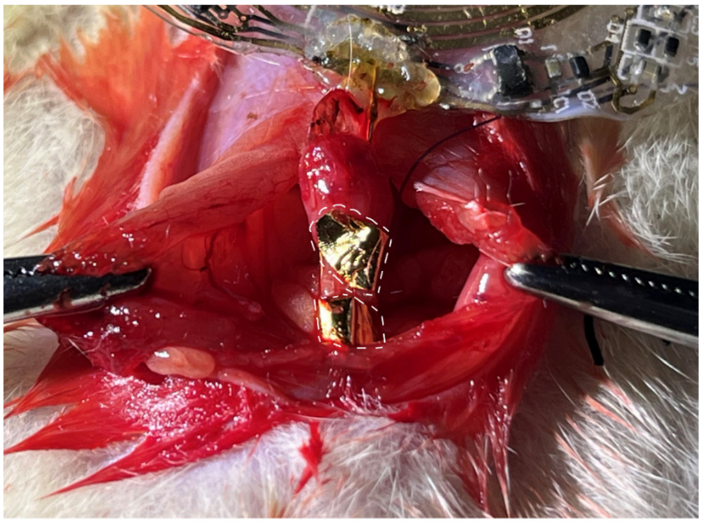

Fig. S27. Intraoperative view of WIPES electrode wrapping the external urethral sphincter (EUS).

==

Location of Change: Line 115

Original Sentences: The wires are specially designed to wrap around the sphincter with curved patches ensuring stability while avoiding squeezing on it.

Revised Sentences: The electrodes are designed as flexible C-shaped gold patches that wrap gently around the sphincter, ensuring stable contact without exerting constrictive pressure (Fig. S27). The inner surface serves as the active interface, while the outer surface and connecting leads are fully encapsulated to maintain insulation.

===

Q. The standard deviation in Figures 4f and 4g is excessively large, and with a sample size of n=4, it is challenging to robustly demonstrate a statistically significant difference.

We agree that the original presentation could be misleading. The “n=4” referred to animals per sex (4♀, 4♂), whereas the Fig. 4e plotted 8-h metabolic-cage bins (urinary-sand mass count / average volume), yielding 8 observations per group. In the revision, we added 4 animals per sex (now 8♀ + 8♂) and extended recording so that each condition includes 16 observations per group.

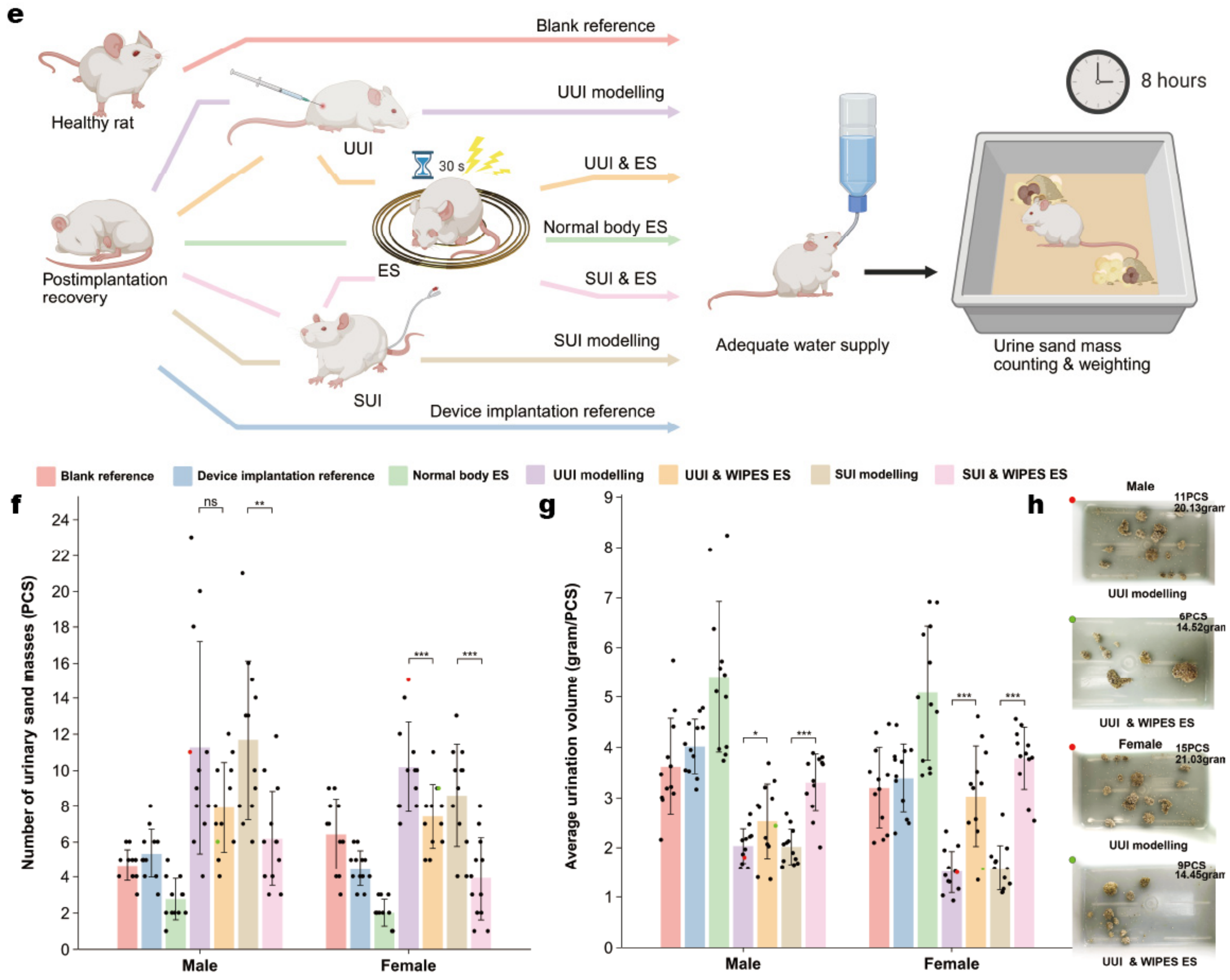

Fig. 4e. Experimental design with seven groups: Blank reference, Device implantation reference, Normal body ES, UUI modelling, UUI & WIPES ES, SUI modelling, and SUI & WIPES ES. 4f. Number of urinary sand masses indicates urination frequency. Each dot represents one measurement while each column represents the mean and the error bars indicate +/- one standard deviation. Statistics of UUI modelling and UUI & WIPES ES groups for females show ***P < 0.001 with N = 8 rats (data for male showing statistically not significant with N = 8 rats) Statistics of SUI modelling and SUI & WIPES ES groups for females show ***P < 0.001 with N = 8 rats (data for male showing**P < 0.01 with N = 8 rats). 4g. Average urination volume indicates effective bladder capacity. Statistics of UUI modelling and UUI & WIPES ES

groups for female show ***$P < 0.001$ with N = 8 rats (data for male showing *$P < 0.05$ with N = 8 rats) Statistics of SUI modelling and SUI & WIPES ES groups for females show ***$P < 0.001$ with N = 8 rats (data for male showing ***$P < 0.001$ with N = 8 rats). 4h. Urine sand mass samples. The marks of red and green dots represent UUI modelling and UUI & ES groups in Figs. 4f and Fig. 4g, respectively.

Q. Please provide the body weight of the rats.

Added. Adult SD rats (both sexes) were used; baseline body weight was 250-320 g (females ~240-300 g; males ~300-360 g).

51 On the other hand, UUI occurs more frequently in men and is more complex to tackle.

Q. The author originally aimed to improve urinary incontinence in males, but the data in this article show that WIPES is more effective for cyclophosphamide-induced urinary incontinence in female animals, which the author did not explain.

We have increased number of rats (8♂, 8♀ per condition) and reanalyzed with a mixed-effects model. In the revised Fig. 4f & 4g, average urination volume improves with ES in both sexes for UUI and SUI (male: UUI vs UUI&ES *, SUI vs SUI&ES ***; female: both ***). For number of urination sand masses, females show significant reductions in both UUI and SUI (***), whereas males exhibit the same direction of change but greater variance and, for SUI show significant reduction (**), for UUI do not reach significance (ns).

We discuss a sex-specific coupling explanation: in rats the female urethra is shorter and the peri-urethral nerve plexus is more compact around the EUS, which concentrates the stimulation and facilitates EUS afferent feedback, which is the pathway central to urge suppression in the CTX UUI model. By contrast, in male, the EUS lies adjacent to the prostate/seminal vesicles and a longer intrapelvic urethra, limiting wrap conformity (partial arc contact), increasing tissue path length, and attenuating stimulation penetration to peri-urethral afferents that are required for urge suppression in the CTX model. In addition, male peri-urethral connective tissue thickness and pelvic geometry likely reduce current density at the target and increase inter-animal variability. Consistent with this, the female cohort shows larger and more uniform gains, while males show a clear trend with wider dispersion. We have added this interpretation to the Discussion and, in the Methods, clarified identical stimulation waveforms with suprathreshold titration in each animal. We also planned refinements about male-specific wrap geometry/arc length and lead routing, and ES amplitude/duty-cycle adjustments to optimize coupling in males.

==

Location of Change: Line 275

Original Sentences: We note that the improvement observed in the male rats in UUI & WIPES ES group (5th) is less obvious, possibly due to anatomical factors such as the proximity of the male urethra to the prostate and seminal vesicles, which may limit the impact of electrostimulation on surrounding sphincter tissues.

Revised Sentences: We note that the improvement observed in male rats in the UUI & WIPES ES group (5th) is less pronounced, likely due to anatomical and coupling differences. The male urethra is longer and lies adjacent to the prostate and seminal vesicles, which reduces wrap conformity and stimulation penetration to peri-urethral afferents. Additionally, thicker connective tissue and pelvic geometry may attenuate current density at the target, resulting in lower and more variable therapeutic response compared with females.

==

228 The incontinence model was induced via intraperitoneal injection of cyclophosphamide (CTX) at a dose of 80 mg/kg [38],

Q. The reference 38, Jerkins et al., 1988, cited by the author does not specify the dosage of cyclophosphamide (CTX) used, and the 80 mg/kg dose is lower than the 100~150 mg/kg commonly used in literature.

Although cyclophosphamide (CTX) is commonly administered at relatively high doses (100–150 mg/kg, i.p.) to induce acute cystitis and urgency urinary incontinence in rodents, such regimens are often accompanied by severe systemic toxicity, profound hematuria, and increased mortality, which substantially limit the feasibility of longitudinal functional studies. In this work, we selected a lower dose of 80 mg/kg, which still produced robust bladder overactivity characterized by reduced intercontraction intervals, increased non-voiding contractions, and frequent small-volume voiding, thereby reproducing the UUI phenotype while minimizing adverse systemic effects. This milder but stable cystitis model enabled repeated assessments, including awake cystometry and electrophysiological recordings, and provided a more suitable platform to evaluate chronic therapeutic interventions such as neuromodulation or electrical stimulation. We therefore consider 80 mg/kg CTX not only sufficient to establish the UUI phenotype but also advantageous for testing long-term efficacy and safety of treatment strategies.

Additionally, we have corrected the citation and now refer to rat studies that explicitly use 80 mg/kg i.p. to induce cystitis/OAB-like phenotypes. For example, Hughes et al., injected female SD rats with cyclophosphamide 80 mg/kg i.p. 24 h before cystometry, and quantified urodynamic/inflammatory changes (Evans Blue extravasation) under this regimen [62]. In addition, Kuo Y et al., describes a chronic 80 mg/kg i.p. ×4 (q.o.d., 7 days) protocol with continuous CMG, further supporting 80 mg/kg as a valid preclinical dose in rats [63]. More broadly, Cindy T et al., summarize chronic CTX cystitis regimens at 40–100 mg/kg given every 2–3 days (7–10 days), placing 80 mg/kg squarely within the commonly used range for rodent models aimed at overactivity/urgency phenotypes while mitigating the toxicity seen with 100–150 mg/kg acute dosing [64]. Accordingly, our Methods now justify choosing 80 mg/kg i.p. to produce a UUI-like DO state with acceptable survival for longitudinal testing.

[62] Hughes, F. M., et al. Simvastatin suppresses cyclophosphamide-induced changes in urodynamics and bladder inflammation. Urology 81(1), 209.e9–209.e14 (2013).

[63] Kuo, Y.-C., Chang, H.-C., & Hsieh, J.-T. Intravesical instillation of calcium-activated chloride channel blockers can ameliorate the change of CMG parameters in cyclophosphamide-induced overactive bladder in rats. 49th Annual Meeting of the International Continence Society (2019),

[64] Tay, C., & Grundy, L. Animal models of interstitial cystitis/bladder pain syndrome. Frontiers in Physiology 14, 1232017 (2023).

==

Location of Change: refer to the section of 'Rat models of urgency urinary incontinence' in the Methods

Original Sentences:/

Revised Sentences: Urgency urinary incontinence (UUI) was pharmacologically induced in both female and male Sprague–Dawley rats through intraperitoneal injection of cyclophosphamide (CTX, 80 mg/kg), a well-established rodent model of cystitis and detrusor overactivity. Systemically administered CTX is metabolized into the urotoxic compound acrolein, which damages the urothelium and lamina propria, triggers inflammatory cascades, and sensitizes bladder afferents. These processes collectively lead to bladder overactivity, characterized by increased micturition frequency, reduced capacity, and secondary urethral sphincter dysfunction, thereby reproducing the urge component of urinary incontinence.

==

Q. The author performed electrical stimulation on the urethra, but the use of cyclophosphamide also induced cystitis. The author should include relevant urinary bladder tissue sections in Figure 6 to provide

supporting evidence.

Added. We now include bladder tissue sections for Blank, CTX, and CTX+ES (WIPES) in the Supplement (Fig. S30). Sections were processed under identical conditions with brief blinded semi-quantitative scoring noted in the legend. As expected, CTX shows cystitis features versus Sham, while CTX+ES is comparable to CTX, indicating urethral stimulation does not exacerbate cystitis. These panels contextualize the UUI model.

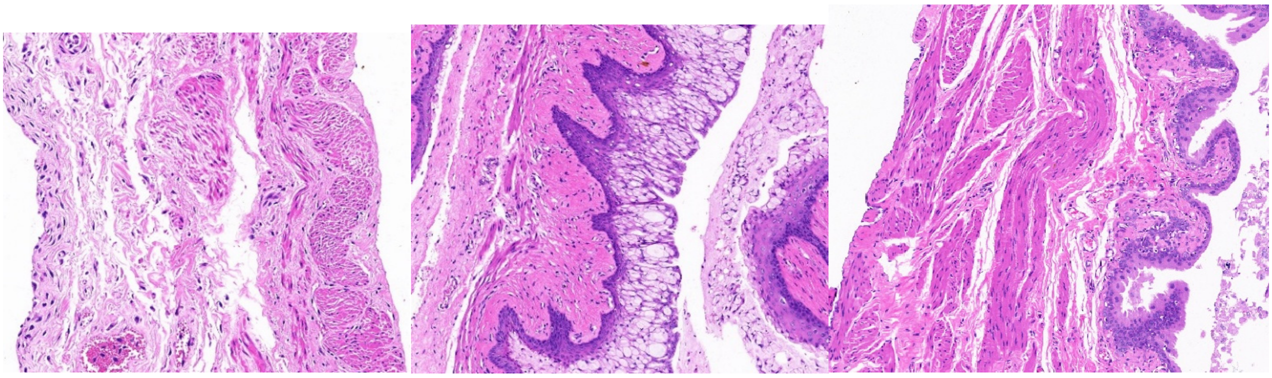

Fig. S30. Blank reference, CTX modeling, and CTX+ES groups' HE staining sections of bladder tissues.

Q. The author inserted a PE tube through the urethral opening and penetrated the urinary bladder, causing not only extreme discomfort but also urethral injury. Therefore, the author should provide complete urethral tissue sections to support their findings.

We appreciate the concern. The transurethral PE micro-tube was used only in the female squeeze-pressure assay (Fig.3e &3f), with a lubricated, soft PE-5 silicone micro-tube inserted under brief anesthesia. All CMG was performed via suprapubic dome catheterization, and LPP used vaginal (♀) / intrarectal (♂) balloons, no routine urethral cannulation was performed.

To document urethral integrity, we now provide full-length urethral tissue sections in the Supplement (Fig. S31): longitudinal and cross-sectional H&E from Sham and a subset of PE-catheterized females (24–48 h). Urothelium remains intact without perforation/ulceration, with only mild focal edema, no luminal hemorrhage or transmural injury is observed. These data support that the brief PE placement did not cause urethral injury. All procedures were Application Format for Ethical Approval for Research Involving Animals approved.

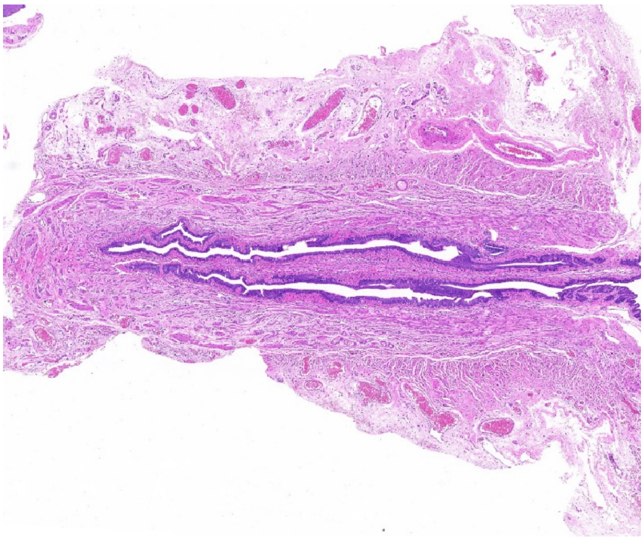

Fig. S31. HE staining of the complete urethral cross-section tissue of female rats after external urethral intubation.

Regarding On demand activation. Who controlled the WIPES? Rats? Researchers? What is the frequency or duration of “relax” in this study?

WIPES was operator-controlled. As shown in Fig. 1e, investigators used a wireless graphical user interface (tablet/PC) to set frequency, pulse width, and amplitude and to start/stop stimulation in real time. The rats did not control the device in this study. In all in-vivo assays we used charge-balanced biphasic pulses at 60 Hz, 0.3 ms/phase, with current titrated slightly above the individual threshold (typically 1.5–2.5 mA, range 1–4 mA). "On-demand" means the operator turned stimulation ON during the defined testing epochs (e.g., CMG or behavioral recording) and OFF outside those epochs. We did not program a separate "relax frequency", "relax" refers simply to the OFF periods between operator-initiated ON blocks.

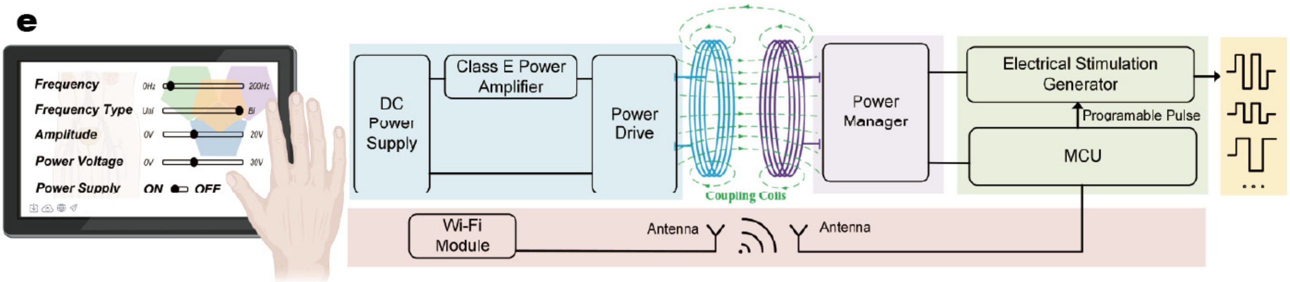

Fig. 1e. Control strategy of the WIPES system including 1) an APP that controls the stimulation frequency, type, amplitude and voltage, 2) Tx coil with power supply and amplification, 3) Rx coil cascaded with functional modules including power management, MCU and stimulation pulse generator, and 4) Wi-Fi module enabling tetherless customized pulse tuning.

Since this study enhanced the sphincter tone, were there any change of detrusor muscle? Any change of kidney? Urinary tract infection? All these potential complications of high urinary sphincter tone should be mentioned. What were the changes of cystometric capacity and detrusor pressure in these rats? After long term implantation of WIPES, were there any ischemic change of sphincter? Fibrosis? Molecular study compared key molecules in smooth muscles. However, urinary sphincter mainly composes of skeletal muscles. The authors showed that the changes in smooth muscle were clear. How about skeletal muscle and its related nerves?

The reviewer raises several important concerns regarding potential consequences of increased sphincter tone, including effects on detrusor function, urinary tract safety, long-term tissue integrity, and the relevance of molecular analyses to sphincter skeletal muscle. We address each point below with newly added data and analyses.

I. Effects of increased sphincter tone on detrusor muscle function

“Since this study enhanced the sphincter tone, were there any change of detrusor muscle?” “What were the changes of cystometric capacity and detrusor pressure in these rats?”

We first examined whether augmentation of sphincter tone altered detrusor function or induced outlet obstruction.

In the UUI (CTX cystitis) cohort, cystometry under fixed-rate filling showed that WIPES increased cystometric capacity, prolonged intercontraction interval (ICI), and reduced non-voiding contraction (NVC) burden (Fig. 5a). Importantly, baseline detrusor pressure and peak voiding pressure did not increase, and post-void residual volumes were not elevated, arguing against functional outlet obstruction or detrusor overloading. These findings indicate that WIPES improves storage physiology without inducing pathological increases in detrusor pressure.

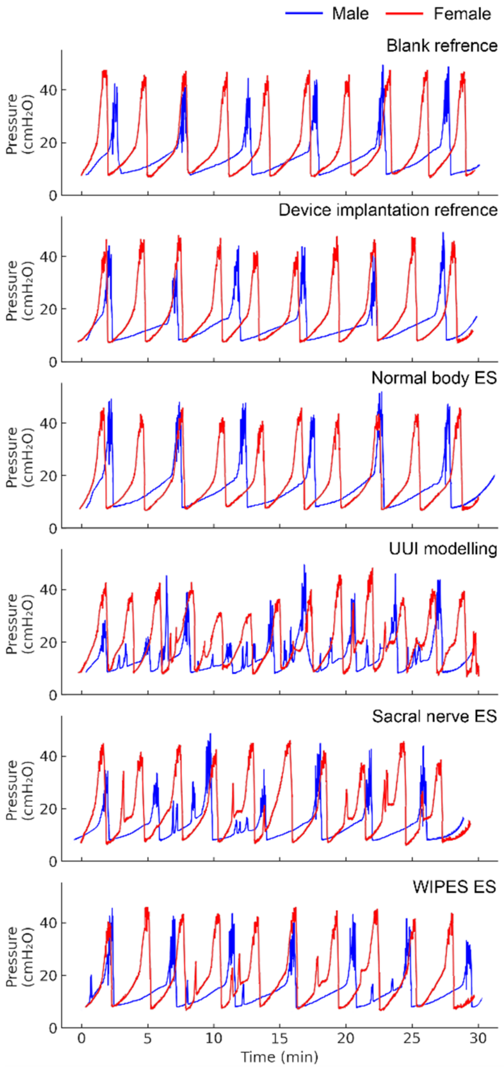

Fig. 5a. Representative cystometric traces of male and female rats under different conditions for 6 groups.

==

Location of Change: Line 310

Original Sentences: /

Revised Sentences: The cystometric analysis (Fig. 5a) provides quantitative assessment of bladder activity by measuring intravesical pressure changes over time (refer to the section of 'Urodynamic Evaluation' in the Methods, and Fig. S28). Three key parameters—non-voiding contraction (NVC) frequency, inter-contraction interval (ICI), and bladder capacity—were analyzed to characterize detrusor muscle function. Rats in the UUI modelling group exhibited abnormally frequent NVCs and markedly shortened ICIs, indicating excessive and unstable detrusor activity associated with urge incontinence. The reduced bladder capacity further confirmed the loss of coordinated neural control and premature triggering of voiding reflexes. In contrast, WIPES electrostimulation (WIPES ES) effectively normalized these parameters. The frequency of NVCs was significantly reduced, while the ICI was prolonged to levels comparable to healthy controls, reflecting restored detrusor stability and improved bladder compliance. Meanwhile, the measured bladder capacity increased markedly, suggesting that the WIPES stimulation enabled fuller bladder filling before the initiation of micturition, indicative of improved neural regulation of the micturition reflex. The SNS group also showed comparable improvement, with slightly more irregular NVCs.

==

II. Kidney, bladder, and urinary tract safety

“Any change of kidney? Urinary tract infection?” “All these potential complications of high urinary sphincter tone should be mentioned.”

To evaluate potential downstream complications of elevated sphincter tone, we assessed bladder and kidney integrity and monitored for urinary tract infection (UTI). Bladder histology (H&E) confirmed CTX-induced cystitis in the UUI model, with no exacerbation by electrical stimulation (Blank, CTX, CTX+ES; Fig. S30). Kidney histology (H&E) in a subset of animals showed no tubular or interstitial injury (Fig. S29).

No clinical signs of UTI were observed, including hematuria, malodorous or turbid urine, weight loss, or systemic infection. Together, these results indicate that WIPES does not induce bladder or kidney injury or predispose to urinary tract infection.

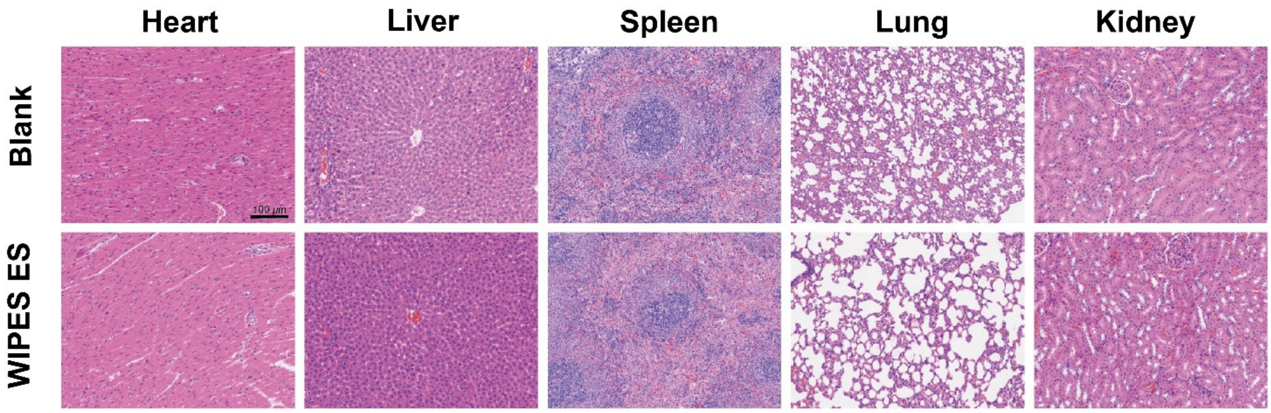

Fig. S29. Organ histological analysis. Post-experiment to evaluate potential systemic effects. H&E staining of major organs (heart, liver, spleen, lungs, and kidneys) revealed no pathological damage in any group.

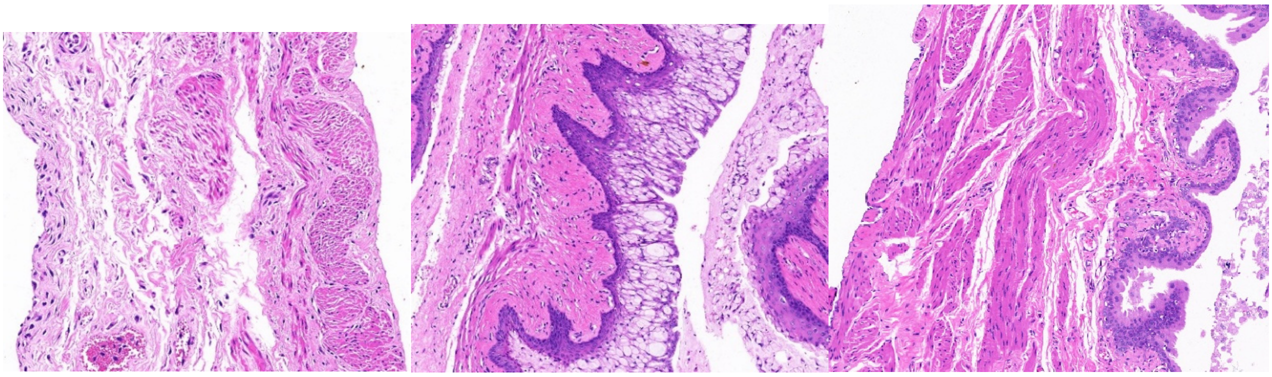

Fig. S30. Blank reference, CTX modeling, and CTX+ES groups’ HE staining sections of bladder tissues.

III. Long-term tissue integrity at the sphincter implantation site

“After long term implantation of WIPES, were there any ischemic change of sphincter? Fibrosis?”

Given concerns about chronic stimulation–induced ischemia or fibrosis, we evaluated tissue integrity at the external urethral sphincter (EUS). H&E staining revealed no ischemic necrosis at the sphincter site.

Masson’s trichrome staining showed no increase in collagen deposition compared with model controls.

TUNEL staining was negative for widespread apoptosis (Fig. 6c). These data demonstrate that long-term implantation and stimulation with WIPES do not induce ischemic injury or fibrotic remodeling of the sphincter.

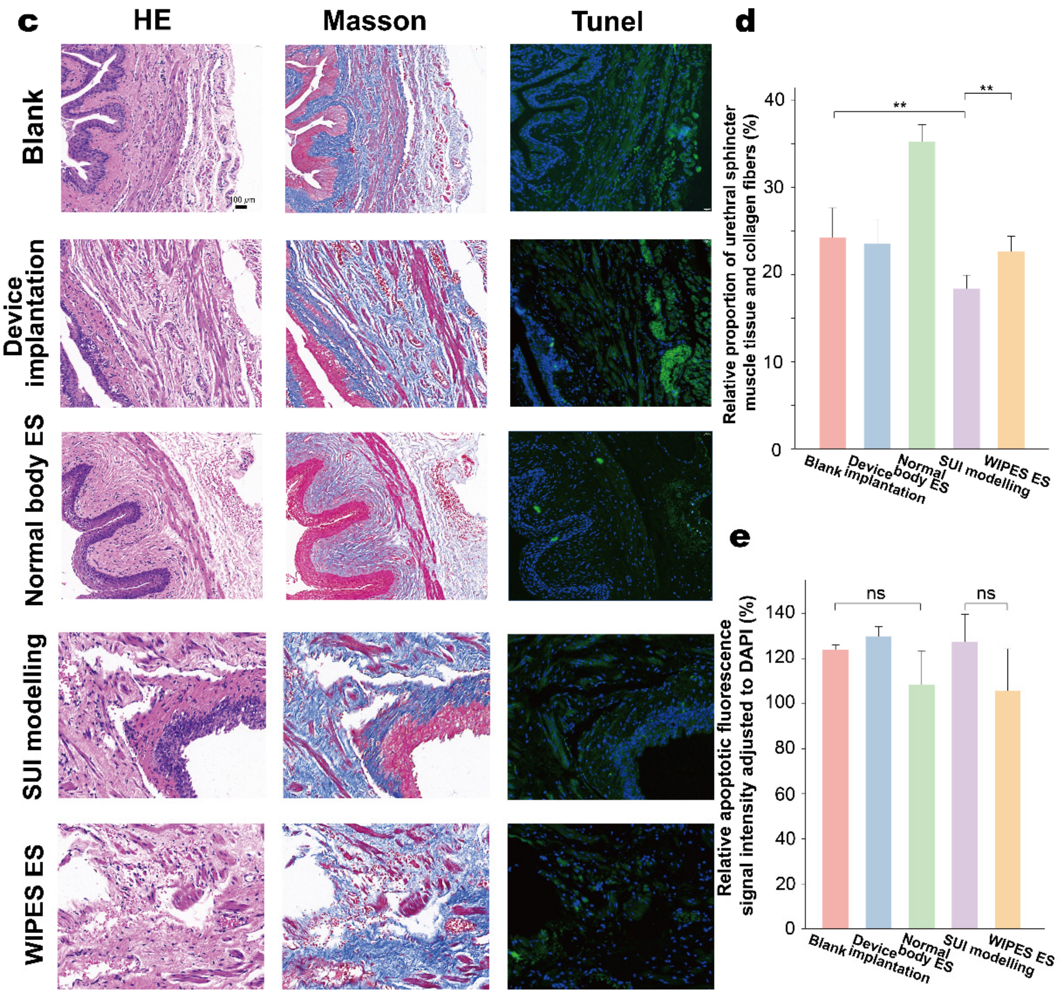

Fig. 6c. Histological evaluation of urethral sphincter tissues using H&E, Masson, and TUNEL staining for all groups. 6d. Quantitative analysis of the relative proportion of urethral sphincter muscle to collagen area for all groups. 6e. Quantification of TUNEL fluorescence signal intensity the five groups.

==

Location of Change: Line 432

Original Sentences: In this study, H&E staining was used to verify the integrity of the sphincter tissue, and Masson staining was used to assess the musculature-to-collagen ratio, and lastly both data were combined to comprehensively evaluate the health status of the urethral musculature and the therapeutic effect, as shown in Fig. 6a. Compared to the Blank reference group (1st, the smooth muscle-to-collagen ratio in the Device implanted group (2nd) showed no significant difference, whereas this ratio showed a significant decrease of 21.88% in the Incontinence modelling group (4th) (Mean ± SD for N = 4 rats. *$p<$ 0.001). Interestingly, the smooth muscle volume in the UI & ES group (5th) showed significant improvement, with an increase of 22.32%, compared to the Incontinence modelling group (4th) (Mean ± SD for N = 4 rats. *$P<$ 0.05)as shown in Fig. 6b. These results suggest that stimulation by WIPES is a key factor in promoting smooth muscle proliferation and functional recovery, nearly restoring it to normal levels. The histological findings align with the functional evaluations described earlier, confirming its potential to relieve SUI symptoms. In addition, TUNEL, staining was employed as an effective method to demonstrate apoptosis in vivo, The green fluorescence intensity indicated the distribution of DNA associated with programmed cell death in tissues (Fig. 6c). There were no significant differences in green fluorescence intensity among the Blank reference group (1st) and Device implantation group (2nd), indicating that device implantation did not induce apoptosis in urethral tissues, while the implantation of the WIPES device and its associated electrostimulation (3rd and 5th group) neither caused nor exacerbated apoptosis in these tissues.

Revised Sentences: In this study, H&E staining was used to verify the integrity of the sphincter tissue, and Masson staining was used to assess the musculature-to-collagen ratio, and lastly both data were combined to comprehensively evaluate the health status of the urethral musculature and the therapeutic effect, as shown in Fig. 6c. Compared to the Blank reference group (1st), the smooth muscle-to-collagen ratio showed a significant decrease of 24.28% in the SUI modelling group (4th) (Mean ± SD for N = 8 rats. ****P* < 0.001). Interestingly, the smooth muscle volume in the WIPES ES group (5th) showed significant improvement, with an increase of 23.14%, compared to the SUI modelling group (4th) (Mean ± SD for N = 8 rats. **P* < 0.05) as shown in Fig. 6d. These results suggest that stimulation by WIPES is a key factor in promoting smooth muscle proliferation and functional recovery, nearly restoring it to normal levels. The histological findings align with the functional evaluations described earlier, confirming its potential to relieve SUI symptoms. In addition, TUNEL staining was employed as an effective method to demonstrate apoptosis in vivo. The green fluorescence intensity indicated the distribution of DNA associated with programmed cell death in tissues (Fig. 6c). There were no significant differences in green fluorescence intensity among each group, indicating that the experiments did not induce apoptosis in urethral tissues.

==

IV. Functional outcomes in SUI models

In stress urinary incontinence models (female vaginal balloon distension; male prostatectomy-associated sphincter injury), we assessed both functional and behavioral outcomes. Functionally, WIPES increased leak point pressure (LPP) and enhanced EUS-EMG guarding and voiding bursts under standardized testing conditions (Fig. 6a and 6b). Behaviorally, average voided volume increased and urinary-sand counts decreased during stimulation (Fig. 4f and 4g). These results confirm effective enhancement of sphincter function without adverse downstream effects.

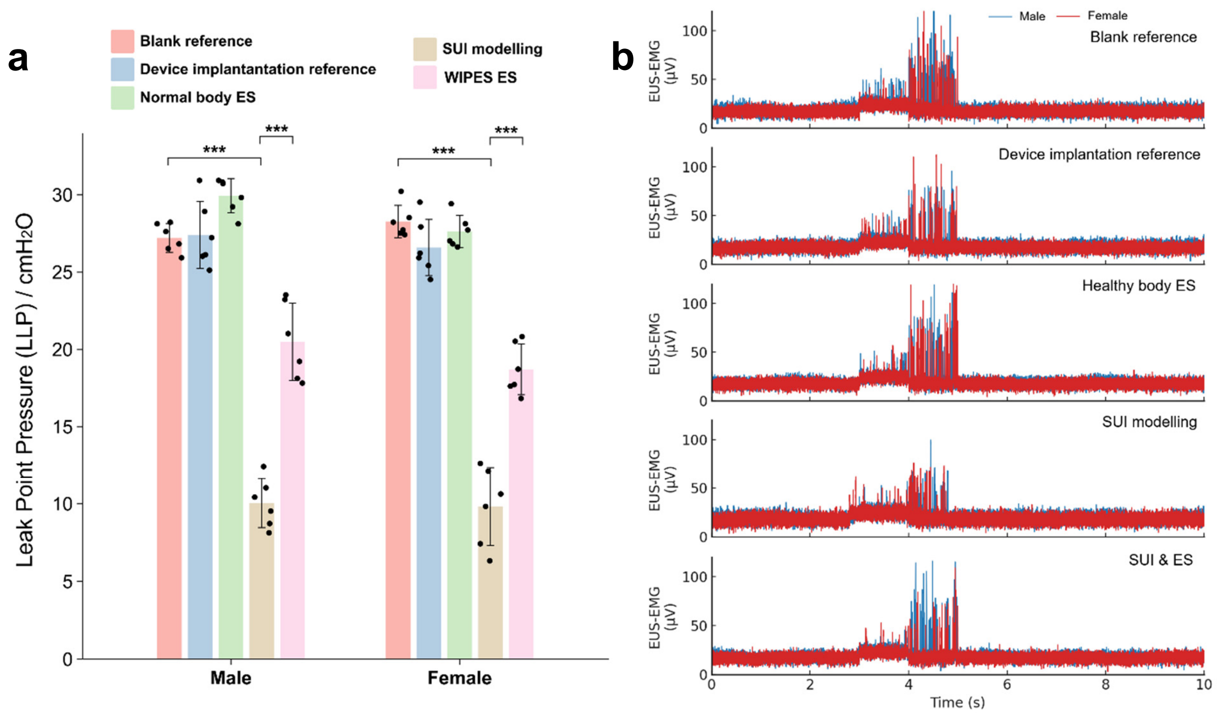

Fig. 6a. Leak point pressure results for five groups, showing significant LPP recovery after WIPES stimulation in both male and female rats. 6b. Representative external urethral sphincter electromyography (EUS-EMG) recordings of all groups

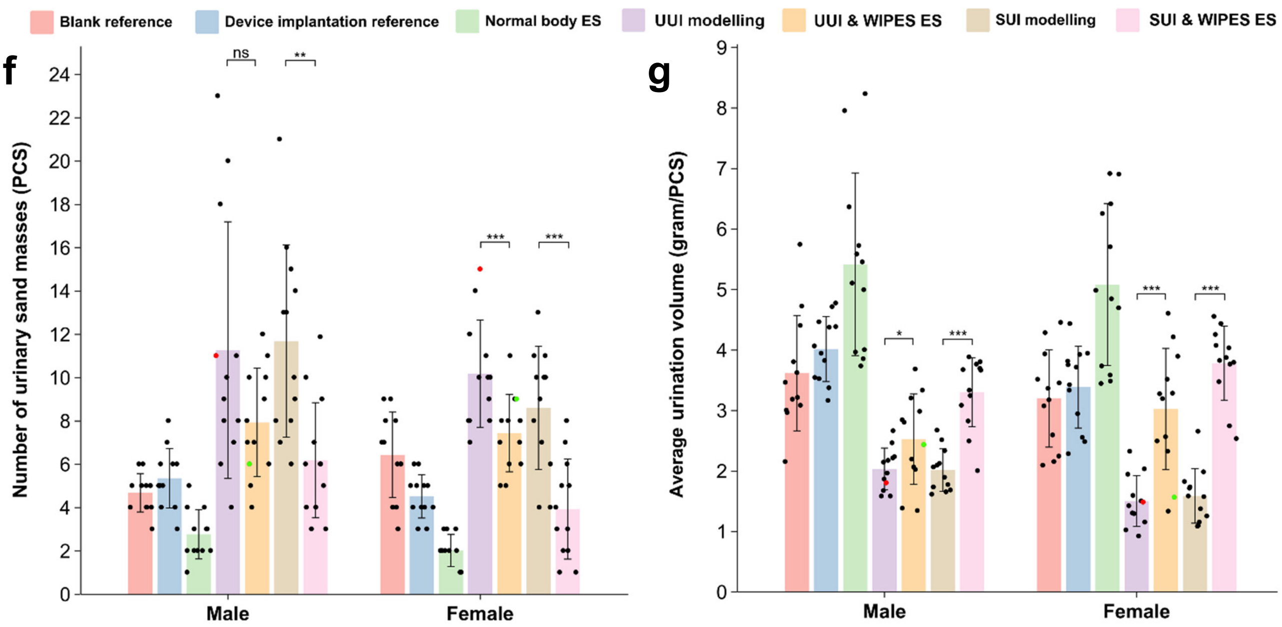

4f. Number of urinary sand masses indicates urination frequency. Each dot represents one measurement while each column represents the mean and the error bars indicate +/- one standard deviation. Statistics of UUI modelling and UUI & WIPES ES groups for females show ***P < 0.001 with N = 8 rats (data for male showing statistically not significant with N = 8 rats) Statistics of SUI modelling and SUI & WIPES ES groups for females show ***P < 0.001 with N = 8 rats (data for male showing**P < 0.01 with N = 8 rats). 4g. Average urination volume indicates effective bladder capacity. Statistics of UUI modelling and UUI & WIPES ES groups for female show ***P < 0.001 with N = 8 rats (data for male showing *P < 0.05 with N = 8 rats) Statistics of SUI modelling and SUI & WIPES ES groups for females show ***P < 0.001 with N = 8 rats (data for male showing ***P < 0.001 with N = 8 rats).

==

Location of Change: Line 414

Original Sentences: /

Revised Sentences: Cystometric measurements and leak point pressure (LPP) testing (Fig. 6a; refer to the section of ‘Urodynamic Evaluation’ in the Methods) revealed a pronounced reduction in LPP in rats subjected to SUI modelling (4th) compared with the Blank group (1st), with a decrease of 64.24%, indicative of compromised urethral closure competence and weakened sphincteric resistance to bladder pressure. The decreased LPP reflects impaired transmission of contractile force within the external urethral sphincter (EUS), resulting in premature urine leakage upon elevated intravesical pressure. Following WIPES ES group (5th), both male and female rats exhibited a substantial recovery of LPP values, with an increase of 97.90%, approaching those of the healthy baseline, demonstrating the efficacy of electrical stimulation in restoring urethral outlet resistance (Mean ± SD for N = 8 rats. ***P < 0.001).

Location of Change: Line 419

Original Sentences: /

Revised Sentences: Concurrently, electromyographic recordings from the EUS (EUS-EMG, Fig. 6b) revealed characteristic alterations in neuromuscular activity patterns. In SUI-modelled rats, the EMG traces showed sparse, low-amplitude, and poorly coordinated bursts during voiding, reflecting disrupted neuromuscular communication and reduced sphincter excitability. In contrast, WIPES-treated animals exhibited rhythmic, high-frequency spike bursts synchronized with the micturition cycle, indicative of reinstated reflex control and functional re-engagement of motor units within the sphincter. These findings

collectively confirm that WIPES stimulation not only enhances urethral closure pressure mechanically, as evidenced by LPP recovery, but also re-establishes physiological neuromuscular coordination essential for continence regulation.

==

V. Relevance of molecular analyses to skeletal muscle and neural components

“Molecular study compared key molecules in smooth muscles. However, urinary sphincter mainly composes of skeletal muscles.” “The authors showed that the changes in smooth muscle were clear. How about skeletal muscle and its related nerves?”

The reviewer notes that the urinary sphincter is predominantly composed of skeletal muscle and questions whether molecular analyses focused on smooth muscle are sufficient. In addition to smooth muscle markers (α-SMA, ROCK2), we expanded analyses to include skeletal muscle remodeling and neural regeneration within the urethral sphincter region. Evidence of improved myogenesis and structural remodeling was observed at the EUS. Neural recovery and synaptic remodeling were supported by increased expression of PGP9.5 and GAP-43, indicating regeneration of sphincter-associated nerves and restoration of neuromuscular connectivity. Thus, the molecular and histological analyses directly address both skeletal muscle and its innervating neural components, consistent with the anatomy and function of the urinary sphincter.

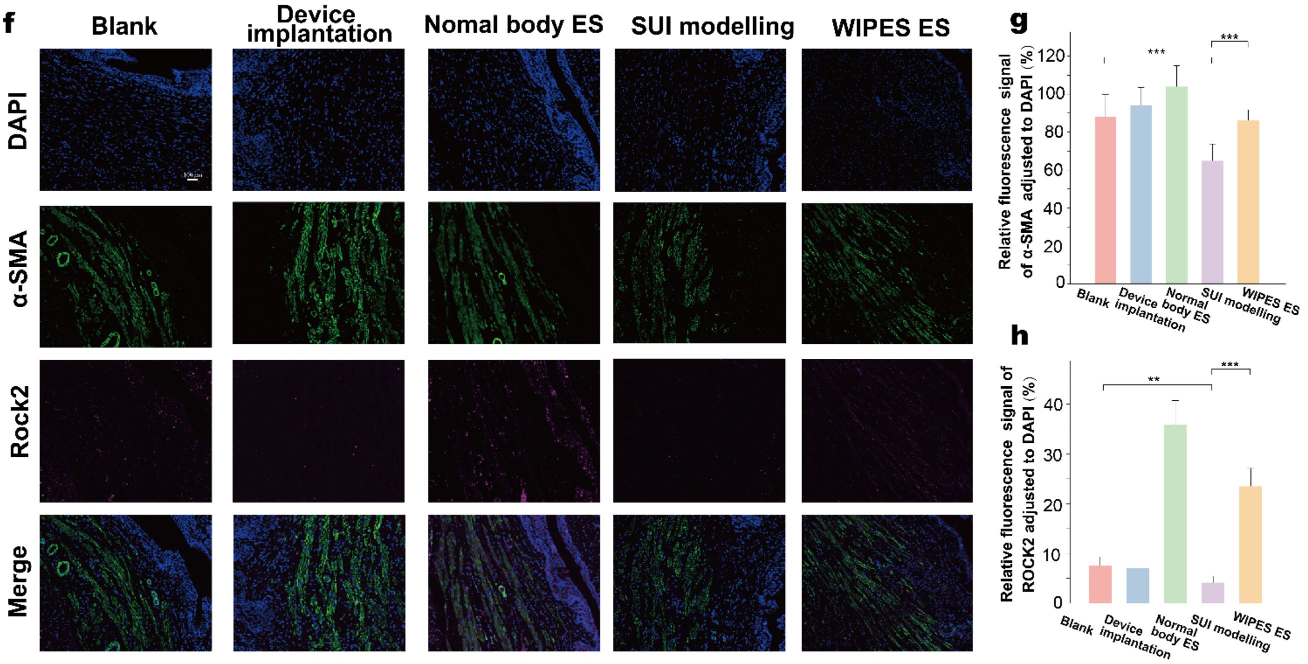

Fig. 6f. Immunofluorescence staining of α-SMA (green) and ROCK2 (magenta) in urethral sphincter tissues, with DAPI (blue) indicating nuclei for all groups. 6g. Quantitative analysis of α-SMA signal intensity normalized to DAPI for all groups. 6h. Quantitative comparison of ROCK2 fluorescence intensity normalized to DAPI for all groups.

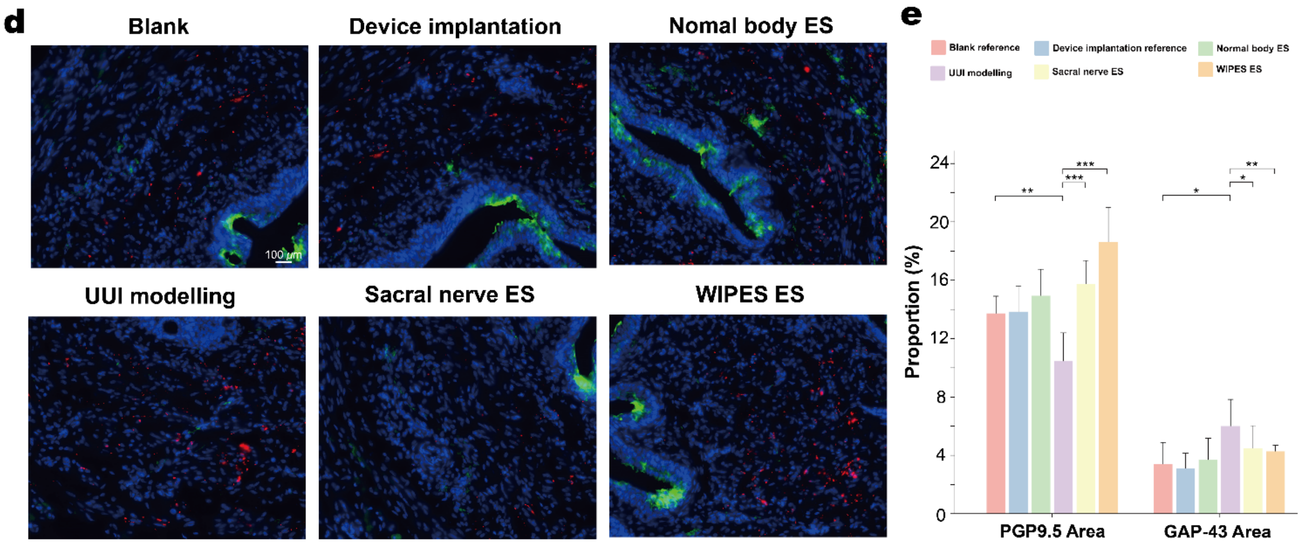

Fig. 5d. Immunofluorescence staining images showing PGP9.5 and GAP-43 expression in urethral sphincter tissues of rats in each group, the green fluorescence indicates the expression of PGP9.5, and red fluorescence indicates the expression of GAP-43. 5e. Statistical analysis of PGP9.5-positive area and GAP-43 area in rats of all groups.

==

Location of Change: Line 444

Original Sentences: The in vivo experimental results demonstrate the effectiveness of WIPES in treating CTX-induced urinary incontinence, which is known as a traditional method for dominantly inducing UUI. We then study the potential mechanism underlying its therapeutic value for both UUI and SUI. On the other hand, for ROCK2, stimulation significantly enhanced the ROCK2 signals in both the Normal body ES group (3rd), with an increase of 782.31%, and the UI & ES group(5th), with an increase of 792.38%, with ROCK2 primarily localized around the urethral smooth muscle cells(Mean ± SD for N = 4 rats, **p*< 0.05.****p* < 0.001), as shown in Fig. 5d.

Revised Sentences: To further investigate the molecular mechanisms underlying tissue recovery, immunofluorescence staining of α-SMA and ROCK2 was conducted (Fig. 6f–h). α-SMA is an actin protein specifically expressed in smooth muscle, forming the core part of muscle cells and maintaining contractile capacity. ROCK2, a downstream effector of Rho kinase, regulates the proliferation and differentiation of cytoskeletal proteins in smooth muscle cells and is a critical factor in promoting smooth muscle contraction. (refer to the section of ' Immunofluorescence Staining ' in the Methods). It showed a clear distribution of α-SMA in all groups, indicating the structural and functional integrity of the urethral smooth muscle and the α-SMA signals in the WIPES ES group (5th) compared with SUI modelling group (4st), with an increase of 32.63% (Fig. 6f). On the other hand, for ROCK2, stimulation significantly enhanced the ROCK2 signals in the WIPES ES group (5th) compared with SUI modelling group (4st), with an increase of 471.46%, with ROCK2 primarily localized around the urethral smooth muscle cells (Mean ± SD for N = 8 rats; **P < 0.01, ***P < 0.001), as shown in Fig. 6h. We note that the induced incontinence results from nerve inflammation and damage, along with the overexpression of relaxant neurotransmitters. WIPES is believed to mitigate these effects by reducing the expression of relaxant neurotransmitters through electrical signals, maintaining axonal regeneration and myelin growth in nerve cells, and activating the ROCK2 signaling pathway to enhance the proliferation and differentiation of smooth muscle cells, thereby restoring urethral function from causes for SUI.

Location of Change: Line 345

Original Sentences: /

Revised Sentences: At the central level, c-Fos expression results demonstrated that WIPES electrostimulation effectively suppressed overactivation within the brainstem micturition centers, restoring balanced communication across the bladder–brainstem neural circuit. To further examine the peripheral outcomes of this neuromuscular interface effect, we evaluated nerve regeneration within the urethral sphincter region and synaptic recovery using PGP9.5 and GAP-43 immunofluorescence markers (green and red, Figs. 5d and 5e). PGP9.5 serves as a pan-neuronal marker reflecting the overall density and integrity of peripheral nerve fibers, whereas GAP-43 is a growth-associated protein expressed during axonal regeneration, indicating active neural remodeling and repair (refer to the section of 'Neural Immunostaining & Quantification' in the Methods). As shown in Fig. 5e, the PGP9.5 Area represents the proportion of PGP9.5-positive regions within the total tissue area, quantifying the nerve fiber density. Compared with the blank reference (1st), the UUI modelling group (4th) showed an 23.92% reduction, indicating marked nerve degeneration and fiber loss. Following treatment, the SNS ES group (5th) exhibited a 50.53% increase relative to the model group, reflecting partial recovery characterized by discontinuous and loosely organized fibers. In contrast, the WIPES ES group (6th) demonstrated a 78.43% increase in PGP9.5 Area, showing densely packed and continuous neural structures, indicative of robust peripheral nerve restoration. To further assess regenerative activity, GAP-43—an established marker of axonal sprouting and early-stage neural remodeling—was quantified across all groups. The UUI modelling group (4th) exhibited a markedly elevated GAP-43 signal, showing a 77.54% increase compared with the blank reference, indicating extensive but disorganized compensatory sprouting of immature axons following nerve injury. In contrast, SNS ES group (5th) reduced GAP-43 expression by 25.06%, and WIPES ES group (6th) by 28.99%, relative to the UUI model, suggesting that electrical stimulation helped suppress excessive, non-functional sprouting while promoting a more stabilized and orderly regeneration environment. These results support that WIPES facilitates structured neural recovery rather than chaotic axonal overgrowth. Notably, WIPES treatment normalized the GAP-43 Index to a moderate yet orderly level while significantly increasing the PGP9.5 area, suggesting that WIPES promoted structured axonal regeneration and functional reinnervation rather than chaotic overgrowth. This coordinated pattern implies that effective neuromuscular interface activation restores both nerve density and morphological integrity of regenerated fibers (Mean ± SD for N = 8 rats; ***P <0.001, **P < 0.01, *P < 0.05).

Location of Change: refer to the section of 'Neural Immunostaining & Quantification' in the Methods

Original Sentences: /

Revised Sentences: A new subsection has been added in the Methods to describe the procedures for Neural Immunostaining & Quantification, detailing the staining and analysis protocols used to assess peripheral nerve regeneration and neurotransmitter expression in urethral sphincter tissues. The section includes descriptions of immunofluorescence labeling for neuronal markers (PGP9.5, GAP-43) along with image acquisition and quantitative analysis methods used to evaluate nerve density and axonal regeneration. Representative fluorescence images and quantification results are provided in the main text figures to ensure methodological transparency and reproducibility.

==

Summary

Across both UUI and SUI cohorts, WIPES enhances sphincter tone and functional continence while not elevating detrusor pressures, not inducing urinary tract or kidney complications, and not causing ischemia or fibrosis at the sphincter. Importantly, functional improvements are accompanied by skeletal muscle remodeling and neural recovery, providing mechanistic support for the observed therapeutic effects.

Line 346. There was no evidence that SUI in these rats improved. Assessment of SUI in rats was not performed.

We agree that SUI required direct assessment and have added a full SUI cohort with sex-specific modeling and urodynamic validation (refer to method 'Rat models of stress urinary incontinence'). Females underwent vaginal balloon distension, and males received a post-prostatectomy-like injury paradigm shown in Fig. S23 & S24. After recovery, we tested WIPES therapy and quantified SUI outcomes by leak-point LPP and EUS-EMG under standardized conditions. In females, WIPES produced a significant increase in LPP and enhancement of EUS-EMG guarding bursts (amplitude and burst area; statistical details in Fig. 6a - 6b). In males, the direction of change was consistent (LPP↑; guarding bursts↑) with larger variance and a smaller effect size , which we attribute to male pelvic anatomy. We also include the behavioral readouts for SUI. In the revised Fig. 4f & 4g, average urination volume is significantly improved with WIPES in both sexes (SUI modelling vs SUI & WIPES ES: *** for males; *** for females), and the number of urinary sand masses is significantly reduced (SUI modelling vs SUI & WIPES ES: males: **; females: ***). These statistics were obtained with a mixed-effects model (bins nested within animals). To corroborate functional gains, we added sphincter/neuromuscular histology after chronic treatment. H&E/Masson/TUNEL demonstrate preserved urothelium and reduced fibrosis with treatment (Fig.5c), and α-SMA/ROCK2 immunofluorescence shows improved smooth/striated muscle architecture consistent with recovery of outlet competence (Fig. 6f). Together, these additions directly document SUI improvement after WIPES stimulation and address the prior gap in SUI assessment.

Line 347. Similarly, the authors could not say this device is good for mixed UI.

We agree that SUI required direct assessment, and as a result, we have added a full SUI cohort with sex-specific modeling and urodynamic validation, as requested by the reviewer. We modeled and validated UUI and SUI separately, then demonstrated that the same local interface (WIPES on the external urethral sphincter, EUS) produces benefits aligned to each component—restoring outlet resistance (SUI) and stabilizing storage/urge physiology (UUI).

UUI arm (CTX cystitis, revised). Rats received cyclophosphamide to induce detrusor overactivity/urge. Under fixed-rate CMG, WIPES increased capacity, prolonged ICI, and reduced NVC burden. To probe mechanism, c-Fos activity in the periaqueductal gray (dlPAG and vlPAG) shifted toward baseline, and nNOS/VIP profiles normalized, consistent with engagement of peri-urethral afferents and reflex-level modulation. Behaviorally, the urinary-sand assay mirrored these findings: average urination volume improved significantly (UUI modelling vs UUI & ES: * for males, *** for females), and the number of urinary-sand masses decreased—significant in females (***), but ns in males at this sample size, likely reflecting greater inter-animal variance and reduced peri-urethral field coupling in males. In a head-to-head comparison with SNS, WIPES produced larger improvements in behavior (average volume↑, sand count↓), whereas SNS changes were smaller (Fig. 4f–g; Fig. S26b–S26c).

SUI arm (sex-specific models). Females underwent vaginal balloon distension; males, a post-prostatectomy-like injury paradigm (Fig. S23–S24). After WIPES therapy, leak-point pressure (LPP) rose and EUS-EMG guarding/voiding bursts strengthened under standardized testing. Behavioral metrics also improved: in Fig. 4f & 4g, average urination volume is significantly improved with WIPES in both sexes (SUI modelling vs WIPES ES: *** for males; *** for females), and the number of urinary sand masses is significantly reduced (SUI modelling vs WIPES ES: males: **; females: ***). Histology corroborated functional gains: H&E/Masson/TUNEL indicated reduced fibrosis/apoptosis with preserved urothelium; α-SMA/ROCK2 supported myogenic restoration.

Interpretation for MUI. Together, these results show that a single, minimally invasive EUS interface

improves outlet competence in SUI and dampens urge-related bladder hyperexcitability in UUI. We therefore frame our conclusion as translational potential for MUI by addressing both components with one implant, while explicitly acknowledging that we did not use a one-step MUI model. We tested sequential dual-modeling but observed survival and organ-integrity limitations. Future work will evaluate combined/large-animal models and optimize male anatomy-specific patch geometry and parameters.

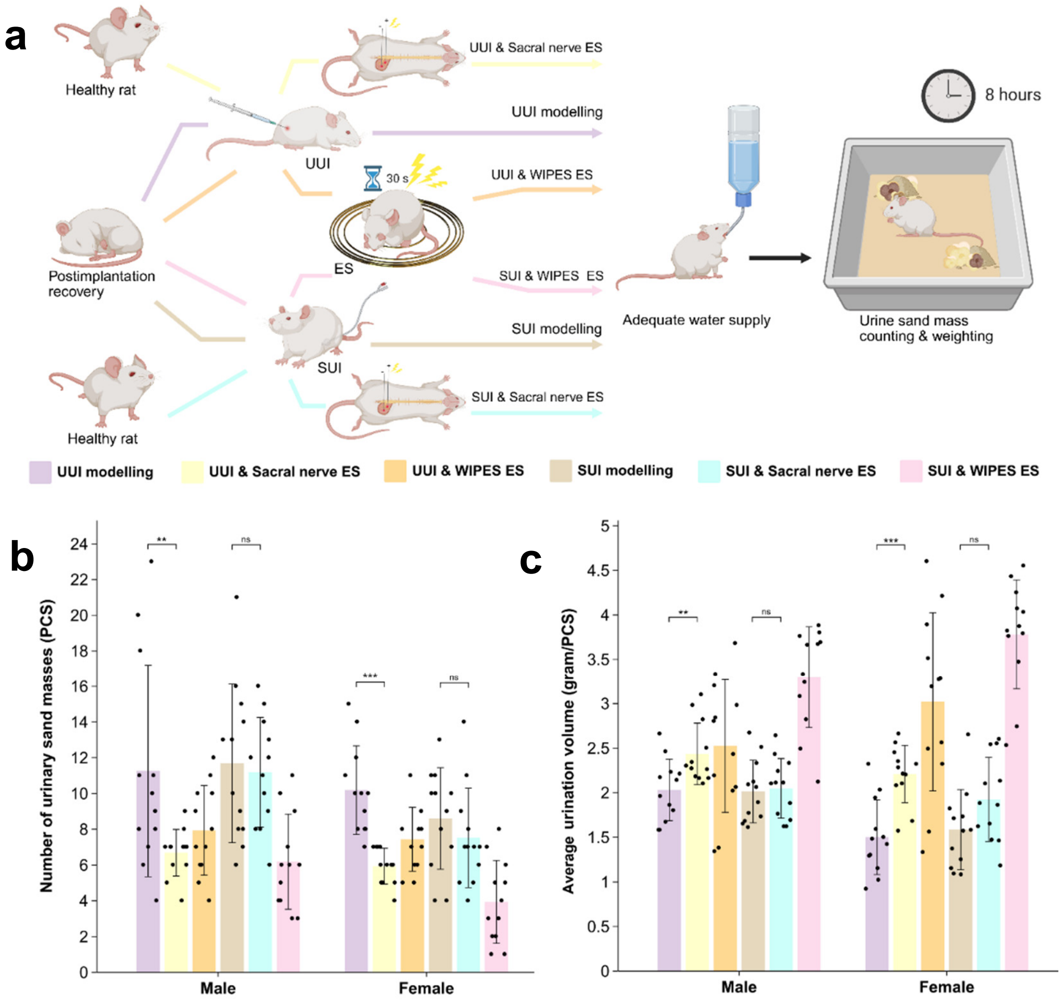

Fig. S26. Comparison with WIPES and traditional SNS for UUI or SUI. a. Workflow. b. Number of urinary sand masses. c. Average urination volume.

Response to the Reviewers' Comments

We thank the reviewers for the constructive feedback on our paper. We have substantially modified the manuscript based on this feedback and believe that the new version is significantly improved. A point-by-point description of the changes is provided below, where our responses to the reviewer feedback are in blue. Meanwhile, red marks indicate changes from previous submissions.

Below we provide a page index to facilitate navigation of the detailed responses to each reviewer.

Reviewer 1: Pages 2.

Reviewer 2: Pages 3.

Reviewer 3: Pages 4-13.

Reviewer 4: Pages 13–15.

Reviewer 1:

We thank the reviewer for the positive evaluation of our work. We appreciate the reviewer’s time and constructive assessment.

Reviewer 2:

We sincerely thank the reviewer for the review and for acknowledging that all previously raised concerns have been fully addressed. We appreciate the reviewer's support for publication of the revised manuscript.

Reviewer 3:

I would like to thank the authors for addressing the comments and revising the document accordingly.

We thank the reviewer for the careful re-evaluation of the manuscript, which has helped us further improve the clarity and rigor of the work.

1. Authors state that the hypothesized mechanism of action on the bladder to addresses the urge component of MUI, is via indirect activation of sacral circuitry. However, the only supporting data is via post-mortem analysis of cFOS of the periaqueductal grey. While this area is indeed relevant for micturition, cFOS at this level does not provide any information regarding sacral circuitry or activation. In addition, this analysis does not provide mechanistic insight, rather an observational study. To confirm the indirect activation of sacral circuitry, would need to add additional electrophysiological experiments that confirm the engagement of spinal circuitry for bladder.

We thank the reviewer for this important and thoughtful comment. We agree that c-Fos mapping at the level of the periaqueductal grey (PAG) does not directly demonstrate activation of sacral spinal circuitry, nor does it provide electrophysiological confirmation of spinal reflex engagement.

In the revised manuscript, we have therefore clarified the role of PAG c-Fos analysis and carefully limited its interpretation. Specifically, we now present PAG c-Fos expression as evidence of central engagement within established brainstem micturition-related regions following peripheral WIPES stimulation, rather than as mechanistic proof of sacral spinal circuit activation. All causal language implying direct or indirect activation of sacral circuitry has been revised or removed.

We have also explicitly acknowledged this limitation in the Discussion, stating that direct validation of spinal circuit engagement would require additional electrophysiological or spinal-level investigations, which are beyond the scope of the current study. The proposed involvement of sacral circuitry is now framed as a hypothesis consistent with the observed peripheral, behavioral, and central findings, rather than a demonstrated mechanism.

==

Location of Change: Line 346

Original Sentences: At the central level, c-Fos expression results demonstrated that WIPES electrostimulation effectively suppressed overactivation within the brainstem micturition centers, restoring balanced communication across the bladder–brainstem neural circuit.

Revised Sentences: At the central level, c-Fos mapping revealed altered activity within brainstem regions associated with urination following WIPES electrostimulation, supporting central engagement in response to peripheral stimulation.

==

a. The new line "Mechanistically, the therapeutic benefit reflects modulation of peri-urethral neural signaling and regeneration of the sphincter musculature, addressing a previously unfilled restoration pathway in rat incontinence models." reads as a conclusion, but without supporting data, this sentence is only a non-tested hypothesis.

We appreciate the reviewer's careful reading and agree that the original wording of this statement could be interpreted as a mechanistic conclusion not fully supported by the presented data.

To address this concern, we have revised the manuscript to downgrade this statement from a mechanistic conclusion to a hypothesis-driven interpretation. In addition to revising the specific sentence in question, we have also systematically reviewed the manuscript to identify and modify

terminology that could overstate mechanistic causality or functional restoration. In particular, words and phrases such as "restore," "mechanistically," and "re-establish" have been replaced with more cautious language (e.g., "improve," "suggest," "is consistent with," or "supports a proposed model") where appropriate.

As a result, the revised text now explicitly frames the proposed mechanism as a conceptual interpretation derived from the convergence of peripheral functional improvement, histological remodeling, and central PAG engagement, rather than as a confirmed pathway. Furthermore, we have ensured throughout the manuscript that mechanistic hypotheses are clearly distinguished from experimentally demonstrated outcomes. The revised Discussion emphasizes that while WIPES induces peri-urethral neuromuscular remodeling and elicits central micturition-related responses, the precise neural pathways underlying these effects remain to be elucidated in future studies.

==

Location of Change: Line 33

Original Sentences: WIPES further reshaped peri-urethral neural signaling and enhanced sphincter neuromuscular regeneration, thereby restoring coordinated bladder–urethral control.

Revised Sentences: WIPES was associated with peri-urethral neuromuscular remodeling and improved bladder–urethral functional outcomes, supporting its potential role in coordinated continence control.

Location of Change: Line 97

Original Sentences: Compared with the limited and largely exploratory methods currently available for addressing urinary incontinence in rat models, our work offers a biomedically grounded therapeutic device and a systematic mechanism study as well.

Revised Sentences: Compared with prior preclinical approaches in rat models, our work provides an integrated therapeutic platform supported by multi-level functional and biological evaluations.

Location of Change: Line 316

Original Sentences: The frequency of NVCs was significantly reduced, while the ICI was prolonged to levels comparable to healthy controls, reflecting restored detrusor stability and improved bladder compliance.

Revised Sentences: The frequency of NVCs was significantly reduced, while the ICI was prolonged to levels comparable to healthy controls, reflecting improved detrusor stability and improved bladder compliance.

Location of Change: Line 318

Original Sentences: Meanwhile, the measured bladder capacity increased markedly, suggesting that the WIPES stimulation enabled fuller bladder filling before the initiation of micturition, indicative of improved neural regulation of the micturition reflex.

Revised Sentences: Meanwhile, the measured bladder capacity increased markedly, suggesting that the WIPES stimulation enabled fuller bladder filling before the initiation of micturition, consistent with improved regulation of bladder storage function.

Location of Change: Line 367

Original Sentences: Notably, WIPES treatment normalized the GAP-43 Index to a moderate yet orderly level while significantly increasing the PGP9.5 Area, suggesting that WIPES promoted structured axonal regeneration and functional reinnervation rather than chaotic overgrowth.

Revised Sentences: Notably, WIPES treatment normalized the GAP-43 Index to a moderate yet orderly

level while significantly increasing the PGP9.5 Area, suggesting that WIPES organized axonal remodeling and markers associated with reinnervation, rather than chaotic overgrowth.

Location of Change: Line 370

Original Sentences: This coordinated pattern implies that effective neuromuscular interface activation restores both nerve density and morphological integrity of regenerated fibers.

Revised Sentences: This coordinated pattern suggests an association between WIPES stimulation and increased nerve density with improved morphological organization of regenerated fibers.

Location of Change: Line 374

Original Sentences: Fig. 5. WIPES restores bladder function and promotes neural regeneration in rats with urge urinary incontinence.

Revised Sentences: Fig. 5. WIPES improves bladder function and is associated with neural remodeling in rats with urge urinary incontinence.

Location of Change: Line 402

Original Sentences: The normalized nNOS distribution implies restored nitric oxide – mediated inhibitory tone, preventing premature detrusor contractions, while the orderly re-expression of VIP indicates re-established neurovascular coupling and mucosal relaxation signaling.

Revised Sentences: The normalized nNOS distribution suggests recovery of nitric oxide – related inhibitory signaling associated with suppression of premature detrusor contractions, while the orderly re-expression of VIP is consistent with improved neurovascular and mucosal relaxation – related signaling.

Location of Change: Line 408

Original Sentences: In summary, cystometric and neurobiological analyses jointly confirm that WIPES restores bladder control through integrated peripheral – central neuromuscular restoration.

Revised Sentences: In summary, cystometric and neurobiological analyses collectively suggest an association between WIPES stimulation and altered peripheral–central signaling relevant to bladder control.

Location of Change: Line 410

Original Sentences: The system reestablishes balance within the brainstem micturition network while promoting orderly peripheral nerve regeneration, as evidenced by increased PGP9.5 density and normalized GAP-43 activity.

Revised Sentences: The system is associated with altered activity within brainstem regions implicated in micturition, alongside orderly peripheral nerve remodeling, as evidenced by increased PGP9.5 density and normalized GAP-43 expression.

Location of Change: Line 411

Original Sentences: Concurrent modulation of nNOS and VIP neurotransmitters further supports recovery of excitatory – inhibitory equilibrium, enabling stable detrusor – sphincter coordination.

Revised Sentences: Concurrent changes in nNOS and VIP expression further support modulation of excitatory–inhibitory signaling relevant to bladder–sphincter coordination.

Location of Change: Line 430

Original Sentences: These findings collectively confirm that WIPES stimulation not only enhances urethral closure pressure mechanically, as evidenced by LPP recovery, but also re-establishes

physiological neuromuscular coordination essential for continence regulation.

Revised Sentences: These findings collectively support that WIPES stimulation enhances urethral closure pressure mechanically, as evidenced by LPP improvement, and is associated with improved neuromuscular coordination relevant to continence regulation.

Location of Change: Line 439

Original Sentences: These results suggest that stimulation by WIPES is a key factor in promoting smooth muscle proliferation and functional recovery, nearly restoring it to normal levels.

Revised Sentences: These results suggest that WIPES stimulation is associated with enhanced smooth muscle proliferation and improved functional outcomes, approaching levels observed in healthy controls.

Location of Change: Line 441

Original Sentences: The histological findings align with the functional evaluations described earlier, confirming its potential to relieve SUI symptoms.

Revised Sentences: The histological findings align with the functional evaluations described earlier, supporting the potential of WIPES to ameliorate SUI-related functional deficits.

Location of Change: Line 459

Original Sentences: WIPES is believed to mitigate these effects by reducing the expression of relaxant neurotransmitters through electrical signals, maintaining axonal regeneration and myelin growth in nerve cells, and activating the ROCK2 signaling pathway to enhance the proliferation and differentiation of smooth muscle cells, thereby restoring urethral function from causes for SUI.

Revised Sentences: Based on the observed molecular and histological changes, WIPES stimulation may influence urethral tissue remodeling through modulation of neurotransmitter-associated markers, axonal remodeling, and ROCK2-related signaling pathways to enhance the proliferation and differentiation of smooth muscle cells, thereby contributing to functional improvement relevant to SUI.

Location of Change: Line 463

Original Sentences: In summary, WIPES stimulation effectively restores urethral function in SUI models by reinforcing sphincteric contractility and reestablishing physiological coordination between detrusor and sphincter activity.

Revised Sentences: In summary, WIPES stimulation effectively improves urethral function in SUI models by reinforcing sphincteric contractility and supporting improved coordination between detrusor and sphincter activity.

Location of Change: Line 465

Original Sentences: Histological and molecular analyses further confirm enhanced smooth muscle regeneration, improved muscle-to-collagen balance, and activation of the ROCK2 pathway, indicating both mechanical strengthening and cellular remodeling of the urethral musculature toward full continence recovery.

Revised Sentences: Histological and molecular analyses further support enhanced smooth muscle regeneration, improved muscle-to-collagen balance, and activation of the ROCK2 pathway, indicating both mechanical strengthening and cellular remodeling of the urethral musculature toward full continence recovery.

Location of Change: Discussion

Original Sentences: We have shown that our WIPES system enables effective symptom relief from induced urinary incontinence in living, free-moving rats through implanted, wireless and on-demand

electrostimulation at their sphincters.

Revised Sentences: We have shown that the WIPES system enables wireless, implantable electrostimulation that is associated with significant improvement of urinary incontinence - related functional outcomes in freely moving rat models.

Location of Change: Discussion

Original Sentences: Comprehensive urodynamic assessments confirmed that WIPES restored coordinated bladder and sphincter function, reflecting effective neuromuscular control during micturition.

Revised Sentences: Comprehensive urodynamic assessments revealed functional improvements in bladder and urethral performance consistent with enhanced lower urinary tract control during micturition.

Location of Change: Discussion

Original Sentences: The efficacy of WIPES was then validated in vivo, showing significantly reduced urination frequency and increased voiding volume in stimulated rats.

Revised Sentences: The efficacy of WIPES stimulation was further evaluated in vivo, where treated rats exhibited reduced urination frequency and increased voided volume.

Location of Change: Discussion

Original Sentences: Histological and immunofluorescence analyses further revealed enhanced muscle regeneration and neural remodeling within the urethral sphincter, accompanied by elevated expression of neurotransmission-related markers.

Revised Sentences: Histological and immunofluorescence analyses further revealed enhanced smooth muscle organization and neural remodeling within the urethral sphincter, accompanied by altered expression of neurotransmission-related markers.

Location of Change: Discussion

Original Sentences: Together, these findings demonstrate that WIPES promotes both functional and structural recovery of the neuromuscular system, offering a promising therapeutic approach for urge, stress, and mixed urinary incontinence.

Revised Sentences: Together, these findings support an association between WIPES stimulation and concurrent functional improvement and tissue-level remodeling of the urethral sphincter, highlighting its potential as a neuromodulation-based platform for treating urge, stress, and mixed urinary incontinence.

==

2. While authors have addressed the choice of model for MUI by separating the symptoms and addressing them via their stimulation, the writing is still misleading. The suggestion is that authors change the title and content of the article to clearly state that each symptom is addressed individually and working towards a device for MUI, but their model does not have MUI.

We thank the reviewer for this important clarification. We agree that the original wording could give the impression that a single mixed urinary incontinence (MUI) animal model was employed, which was not the case.

In this study, urge and stress urinary incontinence were modeled separately, and each symptom was addressed individually using targeted WIPES stimulation. This symptom-oriented strategy was intentionally adopted to evaluate the capacity of WIPES to modulate distinct functional components relevant to MUI, rather than to establish or claim a comprehensive MUI animal model.

To avoid ambiguity, we have revised the manuscript to clearly state that the current work focuses

on separate validation of urge- and stress-related dysfunctions, while positioning WIPES as a platform under development toward integrated treatment of mixed urinary incontinence. Corresponding clarifications have been made in the title and in the Introduction to explicitly distinguish symptom-specific validation from a true MUI disease model.

==

Location of Change: Title

Original Sentences: Wireless on-demand electrostimulation implants enable sphincter neuromuscular restoration from mixed urinary incontinence

Revised Sentences: Wireless electrostimulation implants enable sphincter neuromuscular improvement toward mixed urinary incontinence.

Location of Change: Line 251

Original Sentences: The incontinence models were established to simulate both SUI and UUI, enabling evaluation of the WIPES electrostimulation therapy under MUI conditions.

Revised Sentences: The incontinence models were established to separately simulate SUI and UUI, enabling symptom-specific evaluation of WIPES electrostimulation relevant to mixed urinary incontinence.

Location of Change: Line 742

Original Sentences: This setup was used as the control treatment group for evaluating the efficacy of our WIPES device in treating mixed urinary incontinence, allowing direct comparison between traditional sacral nerve modulation and targeted peripheral muscle stimulation.

Revised Sentences: This setup was used as a control treatment group to evaluate the efficacy of the WIPES device in modulating urinary continence‑related functions, enabling direct comparison between conventional sacral nerve modulation and targeted peripheral muscle stimulation.

==

3. Authors have added cystometry studies to the article; unfortunately, the anesthesia selection was not appropriate. Multiple investigations have revealed that isoflurane is a poor choice of anesthetic as it suppresses the micturition cycle, affects the EMG of the EUS, and reduces LPP (10.1080/21681805.2021.1960598, 10.1152/ajprenal.90259.2008) and urethane is the preferred method for cystometry in rats (10.1016/S0024-3205(01)01182-1). This is further evidenced by the cystometry traces presented in Fig 5, where a voiding contraction is observed approximately every 2.5 minutes; yielding a bladder volume of approximately 0.2 to 0.25 mL, where the bladder capacities should be closer to 1.5 mL.

a. As a result, the line that reads "These findings collectively confirm that WIPES stimulation not only enhances urethral closure pressure mechanically, as evidenced by LPP recovery, but also re-establishes physiological neuromuscular coordination essential for continence regulation." is impossible to assert with the current data because isoflurane interferes with EUS EMG, thus it is not possible to assess changes driven by WIPES. Furthermore, the EMG traces should be presented synchronous to Cystometry traces to assess the EMG re-synchronization.

Thank you for these important comments regarding anesthetic choice and the interpretation of cystometry and EUS-EMG data. We agree that isoflurane anesthesia is known to suppress the micturition reflex and interfere with EUS-EMG activity, thereby limiting the use of these recordings for assessing physiological bladder–sphincter coordination or phase synchronization.

To address this concern, we have revised the manuscript to explicitly clarify that cystometry and

EUS-EMG measurements obtained under isoflurane were predefined and interpreted as functional outcome measures, rather than as evidence of physiological neuromuscular coordination or resynchronization. All mechanistic or coordination-level statements implying re-established bladder–sphincter synchronization driven by WIPES stimulation have been accordingly removed or downgraded in both the Results and Discussion sections.

In addition, to improve data transparency and address the reviewer's request for synchronous visualization, we have added a new Supplementary Figure (Fig. S32) presenting representative, time-aligned cystometry and EUS-EMG recordings across all experimental conditions, including both UUI and SUI models. These traces are provided to illustrate recording characteristics under identical acquisition conditions, without being used to infer physiological synchronization.

We have clarified in the main text and figure captions that cystometry was specifically employed to assess bladder overactivity, non-voiding contractions, and storage dysfunction characteristic of the UUI model (Fig. 5), whereas SUI efficacy was evaluated using leak point pressure (LPP) and EUS-EMG as standard functional endpoints for urethral outlet competence and sphincter function (Fig. 6). Together, these revisions ensure appropriate interpretation of the urodynamic data while explicitly acknowledging the limitations associated with anesthetic choice and preserving a clear distinction between symptom-specific evaluation strategies.

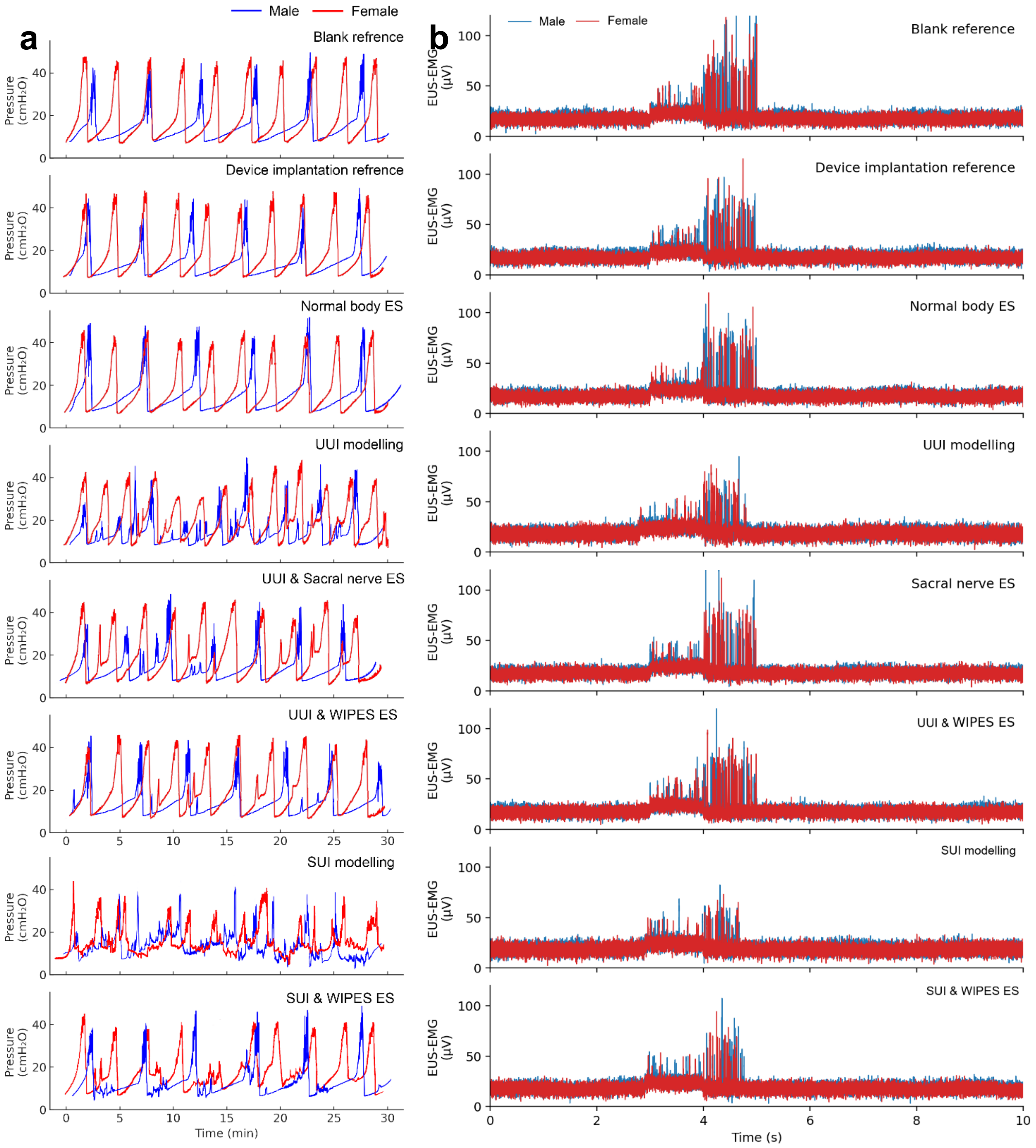

Fig. S32. CMG and EUS-EMG characteristics across UUI and SUI models with different stimulation conditions. (a) Representative CMG recordings showing intravesical pressure changes over time in male (blue) and female (red) rats under different experimental conditions, including blank reference, device implantation reference, normal body ES, UUI modelling, UUI with sacral nerve ES, UUI with WIPES stimulation, SUI modelling, and SUI with WIPES stimulation. These traces illustrate sex-dependent differences in voiding patterns and pressure dynamics, as well as condition-dependent alterations in voiding regularity and peak pressure profiles. (b) Representative external urethral sphincter electromyography (EUS-EMG) recordings from male (blue) and female (red) rats corresponding to the conditions shown in (a). The EMG traces highlight differences in burst amplitude, firing density, and temporal distribution of sphincter activity across disease models and stimulation paradigms, providing complementary neuromuscular context to the CMG measurements.

==

Location of Change: Line 308

Original Sentences: The cystometric analysis (Fig. 5a) provides quantitative assessment of bladder activity by measuring intravesical pressure changes over time.

Revised Sentences: The cystometric analysis (Fig. 5a) provides a quantitative assessment of bladder overactivity and storage dysfunction in the UUI model by measuring intravesical pressure changes over time.

Location of Change: Line 321

Original Sentences: /

Revised Sentences: Additionally, representative, time-aligned cystometry and EUS-EMG recordings across both UUI and SUI models, are provided in Supplementary Fig. S32 for reference.

Location of Change: section of "Urodynamic Evaluation" in Method

Original Sentences: /

Revised Sentences: Because cystometry and EUS-EMG recordings were performed under isoflurane anesthesia, these data were interpreted as functional outcome measures rather than as indicators of physiological bladder–sphincter coordination.

Location of Change: Line 430

Original Sentences: These findings collectively confirm that WIPES stimulation not only enhances urethral closure pressure mechanically, as evidenced by LPP recovery, but also re-establishes physiological neuromuscular coordination essential for continence regulation.

Revised Sentences: These findings collectively support that WIPES stimulation enhances urethral closure pressure mechanically, as evidenced by LPP improvement, and is associated with improved neuromuscular coordination relevant to continence regulation.

==

b. Fig. 5a UUI modeling confirms non-voiding contractions, consistent with overactive bladder; however, it is not clear which trace is supposed to represent the SUI model.

We thank the reviewer for pointing out the ambiguity in Fig. 5a regarding the representation of the SUI model. We agree that the original presentation could lead to confusion.

To address this issue, we have revised the figure captions and corresponding text to explicitly clarify that Fig. 5 presents cystometric analyses exclusively for the UUI model, and does not include data from SUI animals. The SUI model is evaluated separately in Fig. 6 using leak point pressure (LPP) and EUS-EMG recordings, which are standard functional endpoints for assessing urethral outlet competence in stress urinary incontinence.

In addition, to provide a complete overview of urodynamic recordings across all experimental conditions without altering the layout of the main figures, we have added representative cystometry traces for both UUI and SUI models in a new Supplementary Figure (Supplementary Fig. S32). This ensures clear differentiation between models while improving transparency of data presentation.

c. Fig. 6a now shows evidence that LPP increases with ES when compared to SUI, which is consistent with a successful therapy for SUI; however, interpretation of results should be restated because of the selection of anesthetic.

We thank the reviewer for this comment regarding the interpretation of leak point pressure (LPP)

measurements. We agree that anesthetic conditions can influence absolute urodynamic parameters, and therefore the interpretation of LPP results should be appropriately framed.

In the revised manuscript, we have restated the interpretation of LPP data to emphasize that LPP was used as a functional endpoint to assess urethral outlet competence under consistent experimental conditions, rather than as a measure of physiological bladder–sphincter coordination. Importantly, all LPP measurements were obtained under identical anesthetic protocols across experimental groups, allowing valid relative comparisons between treatments.

Accordingly, the Results and Discussion sections have been revised to avoid mechanistic or coordination-level conclusions based on LPP outcomes alone. Instead, LPP improvements are now discussed in terms of enhanced urethral closure function relevant to stress urinary incontinence, consistent with standard practice in preclinical SUI studies.

==

Location of Change: section of "Urodynamic Evaluation" in Method

Original Sentences: These findings collectively confirm that WIPES stimulation not only enhances urethral closure pressure mechanically, as evidenced by LPP recovery, but also re-establishes physiological neuromuscular coordination essential for continence regulation.

Revised Sentences: Consistently, LPP measurements were used as a functional endpoint to assess urethral outlet competence under identical anesthetic conditions across experimental groups, enabling valid relative comparisons between treatments.

==

4. Reviewer 3, comment 8 - Authors have replied with highlighted differences with prior state of the art technology, but have not incorporated these changes to the discussion in the manuscript. These changes should be incorporated as they put their new technology into perspective.

We appreciate the reviewer's suggestion to further contextualize WIPES relative to state-of-the-art technologies in the Discussion. While the previous revision primarily positioned WIPES within the existing literature in the Introduction, we agree that a complementary discussion grounded in the experimental outcomes strengthens the manuscript.

In response, we have expanded the Discussion to explicitly compare WIPES with representative implantable, miniaturized wireless, and non-invasive stimulation approaches in light of our in vivo results. This added discussion emphasizes how WIPES differs from existing technologies not only in form factor and power architecture, but also in its local stimulation strategy, dual functional targeting of urethral outlet competence and urge-related control, and its ability to support sustained functional improvement alongside tissue-level remodeling in preclinical models.

Together with the comparative overview in the Introduction, these additions provide a clearer and more balanced perspective on how WIPES complements and advances current neuromodulation strategies for stress, urge, and mixed urinary incontinence.

==

Location of Change: Discussion

Original Sentences: /

Revised Sentences: When viewed in the context of existing state-of-the-art neuromodulation strategies for urinary incontinence, these findings help clarify the distinct positioning of WIPES. Established implantable neuromodulation systems and miniaturized wireless stimulators typically derive benefit from neural modulation or acute increases in urethral pressure, whereas non-invasive approaches modulate

bladder activity indirectly through distal neural pathways. In contrast, the present results demonstrate that localized, sphincter-level electrostimulation can simultaneously yield sustained functional improvements and tissue-level remodeling at the urethral outlet. This combined functional and structural response differentiates WIPES from approaches that primarily target neural signaling or short-term functional endpoints alone. By employing a conformal, battery-free interface directly at the peri-urethral neuromuscular junction, WIPES addresses both outlet competence and urge-related dysfunction within a single local site, thereby complementing existing implantable and non-invasive therapies rather than replacing them.

==

Reviewer 4:

The article is markedly improved. I still have some concerns:

We thank the reviewer for the positive assessment of the revised manuscript and for the valuable comments, which have guided further clarification and refinement of the study.

1. In the three groups involving electrostimulation, Normal body ES (3rd), UUI & WIPES ES (5th) and SUI & WIPES ES (7th), rats were subjected to three 30-minute daily stimulation sessions, with each consisting of three cycles of 30 seconds of stimulation followed by a 9.5-minute rest interval.

Since on demand application of the device, we do not know the rats' demand. How to identify this needs? Were the rats agitated or any behavioral change during the study?

Actually, the study is not on rats' demand. It is a timed stimulation model. Please clarify it in the Title.

We thank the reviewer for this important clarification regarding the stimulation paradigm. We agree that the current study does not evaluate true on-demand stimulation driven by real-time detection of voiding intention or behavioral cues in rats. Instead, a predefined, time-scheduled stimulation protocol was deliberately adopted.

Although rats cannot actively control stimulation delivery, the WIPES system itself provides programmable control over stimulation parameters, including stimulation duration, duty cycle, and inter-stimulation intervals. This design allows flexible adjustment of stimulation patterns to meet different therapeutic requirements and experimental conditions, while ensuring consistent stimulation dosage across animals and groups. In the present study, a timed protocol was selected to enable systematic evaluation of functional and histological outcomes without confounding variability introduced by behavioral state. During stimulation, rats did not exhibit agitation or abnormal behavior, consistent with the low-intensity and intermittent stimulation applied.

To avoid potential misunderstanding, we have revised the manuscript to explicitly clarify that the stimulation paradigm used in this study is time-scheduled rather than behavior-driven. Correspondingly, the title and relevant sections of the text have been adjusted to reflect this distinction. We emphasize that true on-demand or closed-loop stimulation represents a future direction enabled by the wireless and programmable architecture of WIPES, but was beyond the scope of the current proof-of-concept study.

==

Location of Change: Title

Original Sentences: Wireless on-demand electrostimulation implants enable sphincter neuromuscular restoration from mixed urinary incontinence

Revised Sentences: Wireless electrostimulation implants enable sphincter neuromuscular improvement toward mixed urinary incontinence.

Location of Change: Line 28

Original Sentences: The adhesive-patch-like soft implant integrates wireless power receiving and tunable pulse generation, enabling long-term on-demand stimulation with reduced size (<0.3 cm^3) and weight (<0.9 g).

Revised Sentences: The adhesive-patch-like soft implant integrates wireless power receiving and tunable pulse generation, enabling long-term controlled stimulation with reduced size (<0.3 cm^3) and weight (<0.9 g).

Location of Change: Line 87

Original Sentences: This implant could enable long-term on-demand tetherless electrostimulation at any point-of-care facility equipped with an external emitting coil which could be easily positioned near the lower abdomen with a distance extending to about 20 cm.

Revised Sentences: This implant could enable long-term controlled tetherless electrostimulation at any point-of-care facility equipped with an external emitting coil which could be easily positioned near the lower abdomen with a distance extending to about 20 cm.

==

2. Any treatment to enhance sphincter function or increase bladder outlet resistance may have detrusor damage in the long term. Please discuss it or provide some evidence that this device is fine in long term use.

We thank the reviewer for raising this important concern regarding the potential long-term consequences of increased urethral outlet resistance. We agree that sustained enhancement of sphincter function could, in principle, pose risks to detrusor integrity or upper urinary tract function if not properly controlled.

In the present study, the safety of the WIPES device was evaluated from multiple perspectives. We performed mechanical and physical stability tests to verify the robustness of the device structure under repeated deformation, as well as electrochemical stability assessments to confirm reliable stimulation performance over extended operation. In addition, biocompatibility and tissue safety were evaluated through histological analyses of peri-implant tissues and major organs following implantation, with no evidence of inflammation, ischemic injury, or pathological remodeling observed. Importantly, rats implanted with WIPES for extended durations (up to >50 days) remained healthy, with no detectable adverse effects on bladder, kidney, or surrounding tissues.

To further address this concern, we have added a statement in the Future Work section emphasizing that long-term evaluation of detrusor function, upper urinary tract safety, and chronic outlet resistance will be essential for future translational studies, particularly in large-animal models and extended implantation periods. Together, these data and planned investigations support the long-term safety potential of WIPES while acknowledging the importance of continued evaluation.

==

Location of Change: Discussion

Original Sentences: /

Revised Sentences: In future translational studies, extended-duration implantation and large-animal models will be essential to systematically evaluate long-term detrusor function, upper urinary tract safety, and the physiological consequences of sustained modulation of urethral outlet resistance.

==
